# Intrinsically disordered signaling proteins: Essential hub players in the control of stress responses in *Saccharomyces cerevisiae*

**DOI:** 10.1371/journal.pone.0265422

**Published:** 2022-03-15

**Authors:** Leidys French-Pacheco, Omar Rosas-Bringas, Lorenzo Segovia, Alejandra A. Covarrubias

**Affiliations:** 1 Departamento de Biología Molecular de Plantas, Instituto de Biotecnología, Universidad Nacional Autónoma de México, Cuernavaca, Morelos, México; 2 Departamento de Ingeniería Celular y Biocatálisis, Instituto de Biotecnología, Universidad Nacional Autónoma de México, Cuernavaca, Morelos, México; CNR, ITALY

## Abstract

Cells have developed diverse mechanisms to monitor changes in their surroundings. This allows them to establish effective responses to cope with adverse environments. Some of these mechanisms have been well characterized in the budding yeast *Saccharomyces cerevisiae*, an excellent experimental model to explore and elucidate some of the strategies selected in eukaryotic organisms to adjust their growth and development in stressful conditions. The relevance of structural disorder in proteins and the impact on their functions has been uncovered for proteins participating in different processes. This is the case of some transcription factors (TFs) and other signaling hub proteins, where intrinsically disordered regions (IDRs) play a critical role in their function. In this work, we present a comprehensive bioinformatic analysis to evaluate the significance of structural disorder in those TFs (170) recognized in *S*. *cerevisiae*. Our findings show that 85.2% of these TFs contain at least one IDR, whereas ~30% exhibit a higher disorder level and thus were considered as intrinsically disordered proteins (IDPs). We also found that TFs contain a higher number of IDRs compared to the rest of the yeast proteins, and that intrinsically disordered TFs (IDTFs) have a higher number of protein-protein interactions than those with low structural disorder. The analysis of different stress response pathways showed a high content of structural disorder not only in TFs but also in other signaling proteins. The propensity of yeast proteome to undergo a liquid-liquid phase separation (LLPS) was also analyzed, showing that a significant proportion of IDTFs may undergo this phenomenon. Our analysis is a starting point for future research on the importance of structural disorder in yeast stress responses.

## Introduction

Stressful environments alter cellular homeostasis, leading to a diversity of adjustment mechanisms in metabolic and developmental programs. These responses allow organisms to efficiently withstand different fluctuation levels in temperature, water availability, osmolarity, reactive oxygen species, and toxic metal ions, among other stressful environments [1–4]. The unicellular eukaryote *Saccharomyces cerevisiae*, or budding yeast, has proved to be an excellent experimental model system for the study of cellular stress responses. Different mechanisms involved in yeast stress acclimation have been described, including adjustments in RNA and protein synthesis [5], chromatin remodeling by changes in histone modifications and its dynamics [6], and other epigenomic tunings, as mechanisms underlying cellular stress memory [7]. More recently, a number of reports have shown that diverse stress conditions trigger the formation of a variety of cellular granules or bodies, through the recruitment of proteins and RNAs involving liquid-liquid phase separation for their ensemble [8, 9].

Transcriptional control reprogramming is one of the most extended strategies to adapt to changing environments in organisms from the different life domains. The transcriptomic response of yeast cells to a wide range of environmental stress conditions involves a substantial number of genes that can be up- or down-regulated [2, 10]. In general, genes required for protection are up-regulated, while those related to protein biosynthesis and growth usually are down-regulated. The activation and repression of this large set of genes is the result of a complex network, interconnecting different regulatory elements that result not only in yeast tolerance to a specific stressful condition, but also in cross-tolerance to overcome associated environmental perturbations [4]. Once yeast cells perceive a stress stimulus, diverse signaling molecules transduce this signal to redirect their gene expression. Some essential elements in these stress transduction cascades are protein kinases A (PKA) and C (PKC) [11, 12] and mitogen activated protein kinases (MAPKs) [13]. These modify diverse TFs, such as Msn2/4 considered master regulators of the central stress response program [14]. The control of diverse network players leads to a transient growth arrest, given the low expression of housekeeping genes, strongly controlled by the TOR (Target Of Rapamycin) kinase complex pathway [15]. In a subsequent phase, genes encoding for protecting proteins and additional regulatory elements are induced by the action of different TFs.

Computational analyses indicated that approximately 30% of eukaryotic proteins contain considerable large disordered or unstructured regions, this set of proteins are usually called intrinsically disordered proteins (IDPs) [16]. The properties of these proteins have been broadly described; they have a characteristic amino acid composition, usually a high content of small, charged and/or polar amino acid residues, and a low abundance of order-promoting amino acids [17]. These attributes result in the lack of a unique three-dimensional structure for these proteins, which instead show highly dynamic conformational flexibility, when compared with globular or ordered proteins. This also implies that IDPs may have large interaction surfaces, and that they may adopt a diversity of conformations that might exhibit different recognition motifs, allowing their association with distinct partners. At the same time, their structural malleability gives access to alternative post-translational modifications, allowing the fine tuning of their activity depending on specific cellular states [18, 19]. The structural versatility of IDPs would be expected in proteins involved in signaling and coordination of diverse regulatory processes. The relevance of structural disordered in some of this type of proteins has been documented in several reports. This is the case of TFs, where about 82–94% of them are predicted to contain intrinsically disordered regions (IDRs) [20, 21], with variable length. TFs typically have a nuclear localization and nuclear export sequences (NLS and NES, respectively*)*, a regulatory domain, a DNA-binding domain that generally presents stable structures, surrounded by IDRs often corresponding to effector domains (activation/repression domain, AD/RD*)*, with the structural properties for a high potential of interaction with different macromolecules [22]. The flexible conformation of these domains leads to a high specificity and low affinity binding to partners, assuring an effective reversibility of their interactions. Other well-known functional characteristics of the activation domains, like post-translational modifications, are consistent with their structural nature, and expose them as the appropriate targets to sense the cellular cues selected in the different signaling pathways. Recently, the transient association of a DNA-binding domain in a TF with a distant IDR, which affects the affinity and selectivity of its binding to DNA, was reported, showing that IDR’s impact may go beyond expectations [23, 24].

To gain insight into the influence of structural disorder in the *S*. *cerevisiae* response to environmental stressful cues, we first identify IDPs in its proteome and, then analyze the prevalence of IDPs/IDRs in those sets of proteins that have been involved in yeast responses to different adverse environments, with particular emphasis on TFs and other signaling proteins. Taking advantage of the plentiful information on the function of proteins participating in different stress response pathways in this eukaryotic model organism, we analyzed yeast databases, and by using bioinformatic tools, we highlighted the role and the impact of structural flexibility of proteins on stress response pathways.

## Materials and methods

### Databases

*In silico* analyses used protein sequences (6,721) from *S*. *cerevisiae* strain ATCC 204508/Sc288c available at the Universal Protein Resource Knowledge base (UniProtKB/Swiss-Prot, UP000002311) [25]. Descriptions corresponding to TFs were complemented with annotations from YEASTRACT+ (**Yea**st **S**earch for **T**ranscriptional **R**egulators **A**nd **C**onsensus **T**racking) [26] and references elsewhere. As support to assign subcellular localization to proteins of interest, we used YEAST GFP fusion localization database [27], compiling information from proteins in different databases. Proteins with contrasting localization information were annotated as ambiguous. To categorize proteins associated to yeast stress response signaling, we consulted the stress pathway map dataset described by Kawakami et al. (2016) [28], that includes heat shock, ion homeostasis, nutrient adaptation, oxidative and osmotic stress signaling pathways. These maps contain information including genes, transcripts, proteins and protein complexes. The different pathways contain 552 unique proteins with documented functions, from which some participate in more than one pathway.

### Structural disorder and protein biochemical properties prediction

Prediction of structural disorder was performed using different meta-predictors: VSL2, based on linear support vector machines [29]; IUPred2 (long version), which considers the pairwise energy estimated from residue composition [30]; and MobiDB-Lite, a program that calculates a consensus score by considering the results from different predictors and their variants (Espritz, IUPred, DisEMBL, GlobPlot) [31]. These predictors take a single protein sequence as input and provide as output a disorder probability in the 0.0–1.0 range for each residue. VSL2 converts the residue scores into a binary system (“Ordered” and “Disordered”). A residue was considered “D” using ≥ 0.5 as threshold. To homogenize the comparative analysis, we followed the same procedure applying an identical threshold. To identify IDPs based on experimental evidence, we decided to use the disorder database MobiDB, which is manually curated and collects information from other databases; applying MobiDB-Lite algorithm, this database ponders experimental data and results from different disorder predictors (DynaMine, FeSS, DisEMBL, Espritz, IUPred, VSL2b, GlobPlot, SEG, Pfilt) [32]. The Uniprot dataset (UP000002311) from *S*. *cerevisiae* proteome (MobiDB last update 10 June 2020) was used as the source of identified proteins. Using each one of the above-described meta-predictors, the following parameters were extracted for each protein sequence: (i) average from disorder scores; (ii) “Disorder” residue proportion in a protein, applying ≥ 0.5 as threshold; and (iii) the numbers of disordered windows (regions with at least 30 consecutive disordered residues with the same threshold).

To predict the spontaneous protein capacity for liquid-liquid phase separation, we used the FuzDrop predictor from the FuzPred method, which estimates the conformational entropy in the droplet state, predicting the binding modes of proteins according to their amino acid sequences [33]. The probability of liquid-liquid phase separation (pLLPS) was determined using the complete protein sequence. A protein with an index higher than the cut off value of 0.64 is likely able to form droplets without additional components. Analyses of all data and plots were made using R. A complete scheme of the followed computational methodology and datasets are shown in S1 Fig and S1 Table.

### Gene functional enrichment analysis

To associate functional terms to the IDPs resulting from the previous evaluation, we used the functional nomenclature defined by Gene Ontology (GO) Consortium (2020-08-10 release) [34]. For GO enrichment analysis we applied hypergeometric tests, using the R clusterProfiler package [35], while the p-value was obtained using the Benjamini-Hochberg method. The GO terms with *p-*values < 0.05 were used as input for the ReviGO tool [36]; this allowed us to reduce the number of terms to just the representative ones, facilitating the interpretation of the results.

To locate IDPs in specific pathways, we used the Kyoto Encyclopedia of Genes and Genomes (KEGG) database, which has diverse maps representing molecular interactions and reaction networks from different organisms [37]. For KEGG pathway enrichment, we employed the clusterProfiler package. The KEGG pathway database was complemented with genes from stress pathways obtained from Kawakami et al. (2016) in order to get a more comprehensive pathway dataset. The disorder enriched pathways were determined by applying hypergeometric tests to the identified IDPs. The *p*-value was adjusted by multiple testing with Bonferroni-Hochberg method. The Pathview package was used to show up the position of IDPs in the enriched pathways [38]. The process followed for this analysis is shown in the flow diagram in S2 Fig.

### Construction of networks based on *S*. *cerevisiae* protein-protein interactions

*S*. *cerevisiae* protein-protein interaction (PPI) information was based on the BioGRID database (August 25, 2020 release) [39]. This database contains the PPIs detected from high and low throughput methods and from scientific literature annotations. Only those multi-validated interactions (MV), including unique interactions and self-loops, were considered for our analysis. The *S*. *cerevisiae* PPI network contains 4,126 proteins able to establish 17,771 interactions. From this network, we selected the nodes of interest.

### Statistical analysis

To evaluate the disorder groups differences in the number of Intrinsic Disorder Regions (IDR) or interactions number, a pairwise Wilcoxon test was performed, and the *p*-value was adjusted with the Bonferroni method. The statistical analysis was made using R. A *p*-value < 0.05 was considered for a statistically significant difference.

## Results and discussion

### Identification of IDPs in *S*. *cerevisiae*

**Fig 1:**
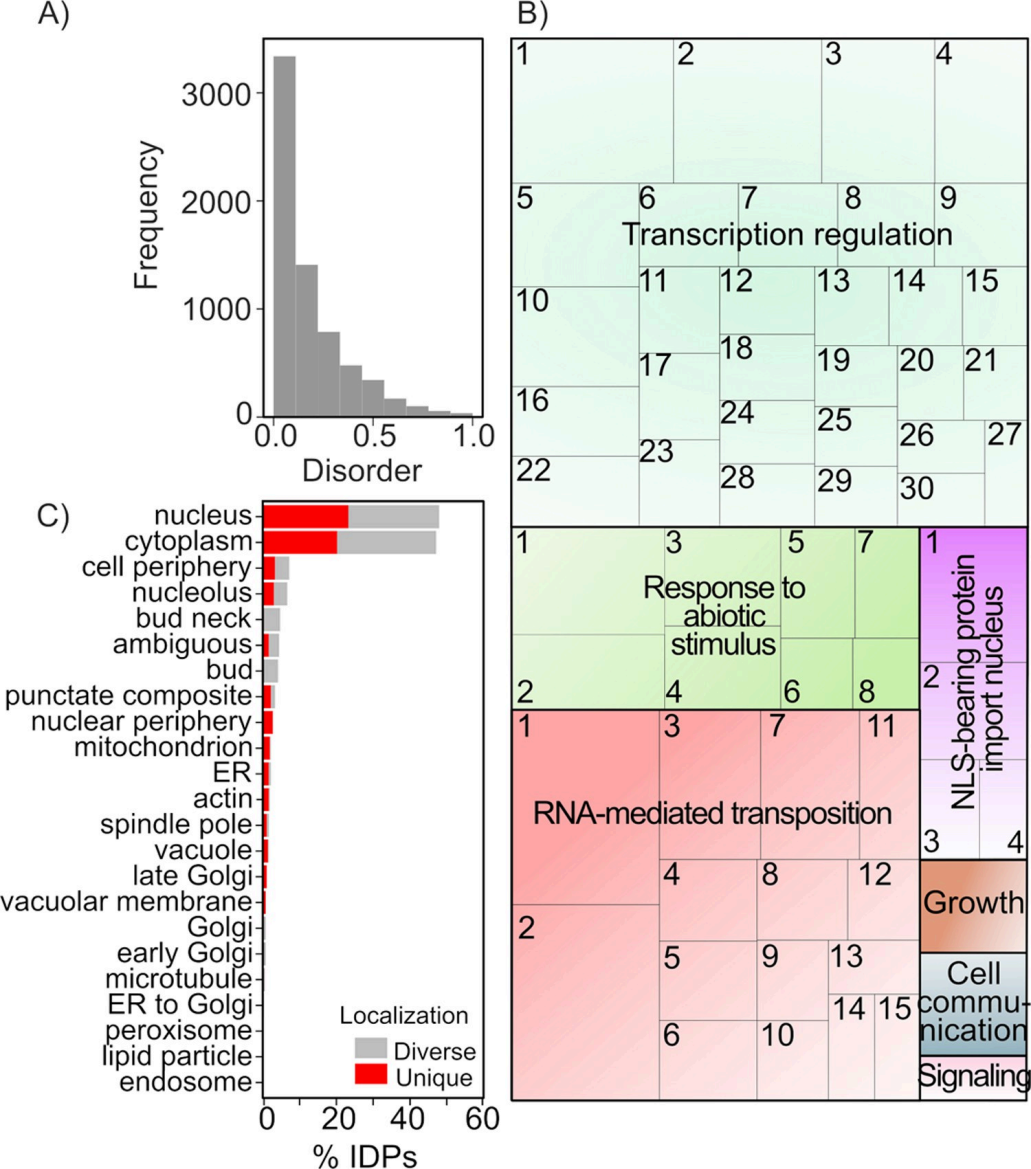
Structural disorder in *S*. *cerevisiae* proteins. Proteins with a disorder ratio ≥ 0.5 according to MobiDB were considered as intrinsically disordered proteins (IDPs). (A) Frequency of structural disorder in yeast proteins according to MobiDB disorder ratio. (B) Gene ontology (GO) analysis of S. cerevisiae IDPs represented as Treemap. The size of each box represents the dimension of the corrected *p*-value from the GO enrichment analysis. Each colored “supercluster” corresponds to common biological functions (represented with a number, S2 Table), named with the GO that showed the lower *p*-value. A full description of each GO can be found in S2 Table. (C) Cellular distribution of IDPs in yeast using YEAST GFP fusion localization database. “Unique” indicates the proteins that can only found in one compartment, meanwhile “Diverse” indicates that those proteins can be found in more than one compartment.

To explore the role of IDPs/IDRs in the signaling of stress responses, we used the model organism *S*. *cerevisiae* because of its well-known advantageous characteristics, the large amount of available information and tools, and because many key cellular processes are similar in yeast and humans, and other eukaryotic organisms. Even though, IDPs/IDRs in yeast have been previously described [21, 40–43], we started by looking for putative IDPs in its proteome, using different meta-predictors, including MobiDB. This last meta-predictor has the advantage of using the evaluation of other predictors, as well as experimental information, providing further support to its scores. In consequence, the comparison of MobiDB analysis with other predictors produced a considerably lower number of IDPs/IDRs. The disorder ratio (D_ratio) obtained by IUPred2 and VSL2 predicted 10 and 26% of IDPs for the yeast proteome, respectively, when MobiDB retrieved only 7.7% of IDPs from the 6,721 yeast proteins (Figs 1A and S3 and S1 Table). Although the different meta-predictors (MobiDB, MobiDB-Lite, IUPred2, and VSL2) use different algorithms, they yield similar D_ratio correlations (∼0.8) (S3A Fig); however, there is a clear difference in the number of IDPs retrieved (S3B Fig). As MobiDB includes experimental data to evaluate the information, we focused on its predictions to delimit the IDP set for further analyses, based on a stricter selectivity. According to this analysis, most yeast proteins (79.3%) show low levels of disorder, with D_ratios between 0–0.3. Even though, there is a set of proteins showing higher disorder scores (D_ratio > 0.3 –< 0.5), we define as IDPs those with a D_ratio ≥ 0.5, according to MobiDB, which corresponds to 7.7% of total yeast proteins (Fig 1A). Hence, our search for budding yeast IDPs involved in stress responses started from a collection of 520 IDPs, as rendered by MobiDB.

### Functional *in silico* analyses of *S*. *cerevisiae* IDPs

The identified IDPs were subjected to a GO assessment for their functional classification. For comparative purposes, the input data was filtered based on the D_ratio established by MobiDB, IUPred2 and VSL2 (D_ratio ≥ 0.5 and ≥ 0.8). Even when there is a slight difference in the significance level between the three methods, we found that, independently of the predictor, proteins with a predicted high structural disorder were functionally allocated to nearly the same biological processes (S4 Fig), despite that MobiDB selected a lower number of IDPs. This result also adds credibility to the representation of IDPs in the different processes occurring during cell life cycle. The distribution of IDPs resulting from this analysis after removal of redundancy for each GO is shown in Fig 1B (S2 Table).

Bearing in mind that one of the GO analysis constraints is its considerable dependence on the number of annotated genes and their associated functional description, this assessment indicated that the processes where IDPs are more enriched are transcriptional regulation from RNA polymerase II promoters, response to abiotic stimuli, chromatin remodeling and RNA-mediated transposition. When the input was the IDP set with a D_ratio ≥ 0.8, the highest enrichment GO is related to regulation of translation in stress responses. Regarding the distribution of IDPs in subcellular compartments, the YEASTGFP database gave similar IDPs proportion in nucleus and cytosol, independently from the origin of the IDP set used as input for this analysis (Figs 1C and S5).

Because the aim of this work is focused on disordered signaling proteins, we first pulled out from the complete yeast proteome all IDPs considered as TFs. The result showed that 170 proteins (approximately 2.5% of the proteome) correspond to all type of TFs. In this set of TFs, we did not find any that could be considered completely disordered, all of them show IDRs dispersed in different segments throughout their primary sequence. This is expected because, in general, their DNA binding domains (DBD or AD/ID) present an ordered structure.

**Fig 2:**
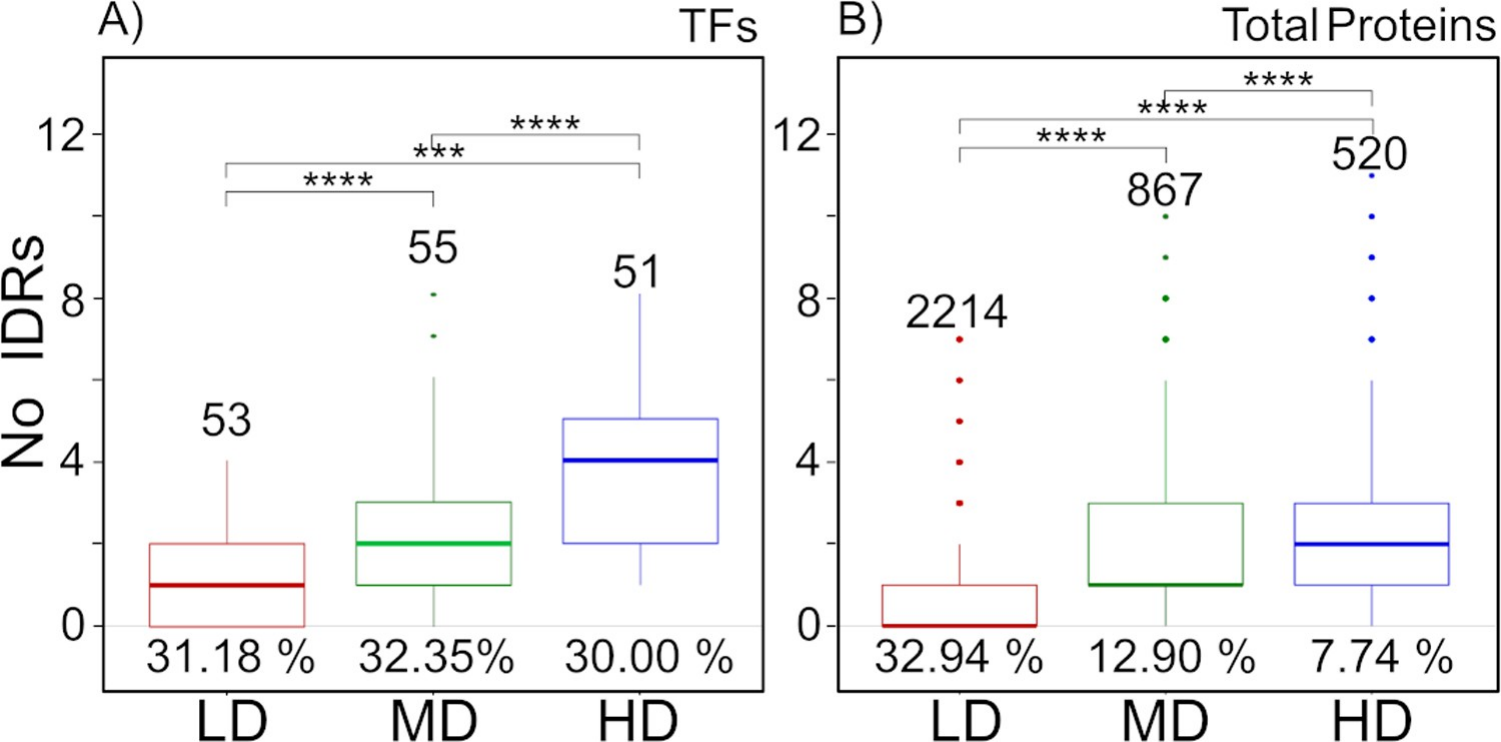
Intrinsically disordered regions (IDRs) *in S*. *cerevisiae* proteome. Number of IDRs identified in TFs (A) and total proteins (B) according to MobiDB disorder ratio. LD, MD and HD correspond to low D_ratio (0.1–0.29), medium D_ratio (0.3–0.49), and higher disorder (D_ratio ≥ 0.5), respectively. Ordered TFs or proteins were not included. Asterisks denote differences evaluated with the Wilcoxon test *** *p*-value ≤ 0.001 and **** *p*-value ≤ 0.0001).

To have a general idea of the IDR distribution in all proteins classified as TFs (170), they were grouped into three sets depending on their level of disorder (D_ratio). A group showing a low D_ratio (LD) (> 0.1–0.3), other with medium D_ratio (MD) (0.3–0.49), and a third one grouping those proteins with the higher level of disorder (HD) (D_ratio ≥ 0.5) (S3 Table). From the 170 TFs, 31% were classified into the LD group, 32% into the MD set, and 30% corresponded to the HD group. Although, IDRs in a protein could have different lengths, we focused on the long-disordered regions of at least 30 residues for this particular analysis, to avoid those IDRs related to flexible linkers or loops in globular proteins [44]. Proteins in the LD group have one IDR on average, whereas those in the MD and HD groups show a mean of three IDRs (Fig 2A). We found 29 TFs showing no disorder. We followed the same analysis using the complete set of yeast proteins, finding that IDRs are more frequent (85.23%) in the TF collection than in the entire group of yeast proteins (35.19%) (Fig 2B and S4 Table).

### Intrinsically disordered transcription factors (IDTFs) enriched in yeast stress response pathways

The intrinsic flexibility of structural disorder in proteins, resulting from the physicochemical properties of their amino acid residues and distribution along their primary sequence, has been associated to a particular sensitivity to the physicochemical characteristics of their surroundings [45–47]. This distinctive characteristic prompted us to look at the distribution of IDTFs in different signaling pathways; specifically, those participating in yeast stress responses.

GO analysis showed, as expected, that IDTFs are enriched in functions such as DNA binding and transcription from RNA polymerase II promoters. In addition to these molecular functions, IDTFs are also highly represented in the response to extracellular stimuli (Tables 1 and S5). The most abundant IDTFs are those regulated by MAPKs (S6 Fig). To dissect the participation of IDTFs in different stress response pathways, we used the information published in Kawakami et al. (2016) [28], where signaling proteins are shown with their corresponding distribution in each transduction path preceding the respective adjustment response(s). From this analysis, we found that pathways signaling heat, nutrient, osmotic and oxidative stress conditions were significantly enriched in IDPs (S7 Fig and S5 Table). This was not the case for other routes, such as those involved in the control of glycerolipid metabolism, biosynthesis of secondary metabolites or of ribosomes, and autophagy (S7 Fig and S5 Table). Considering this information, 62 (11.9%) of the proteins known to be involved in stress signaling and response can be classified as IDPs following the MobiDB stringent criteria. Whereas from the 51 identified as IDTFs, 27 (52.9%) participate in different stress response pathways (Table 2). However, VSL2 offers a considerably higher representation of IDTFs involved in stress responses, suggesting a selective advantage for structural flexibility in stress signaling proteins (S6 Table). It is worth mentioning that among the proteins (non-signaling proteins) showing high levels of disorder (D_ratio 0.8–1.0), those involved in stress defense response are highly represented (Gre1, Sip18 and Yjo4 hydrophilins and Ddr48, Edc1, Zeo1 and Hsp12 proteins) (S1 Table).

**Table 1:** GO clustering of IDTFs^a^^,^^b^.

Representative	Term_ID	Description	log10 *p*-value	uniqueness
Transcriptional regulation	GO:0045944	positive regulation of transcription from RNA polymerase II promoter	-47.506	0.349
	GO:0043620	regulation of DNA-templated transcription in response to stress	-7.0247	0.378
	GO:0043618	regulation of transcription from RNA polymerase II promoter in response to stress	-7.0247	0.342
	GO:0060962	regulation of ribosomal protein gene transcription from RNA polymerase II promoter	-6.2662	0.547
	GO:0043457	regulation of cellular respiration	-3.1083	0.66
	GO:0046015	regulation of transcription by glucose	-2.9257	0.57
Response to nutrient levels	GO:0031667	response to nutrient levels	-11.4795	0.479
	GO:0071310	cellular response to organic substance	-5.3192	0.507
	GO:0010033	response to organic substance	-4.766	0.544
	GO:0009605	response to external stimulus	-10.6486	0.668
	GO:0009628	response to abiotic stimulus	-3.5748	0.659
	GO:0071482	cellular response to light stimulus	-3.2034	0.673
	GO:0046685	response to arsenic-containing substance	-3.0086	0.646
	GO:1901700	response to oxygen-containing compound	-2.9925	0.585
	GO:0080134	regulation of response to stress	-2.5252	0.509
	GO:0010035	response to inorganic substance	-2.8247	0.589
	GO:0071214	cellular response to abiotic stimulus	-2.4344	0.628
Chromatin remodeling	GO:0006338	chromatin remodeling	-4.6724	0.926
	GO:0006325	chromatin organization	-2.3988	0.921
Cell communication	GO:0007154	cell communication	-6.6367	0.932

^a^ IDTFs according to MOBIDB ≥ 0.5 D_ratio

^b^ GO classification according to REVIGO.

**Table 2:** Distribution of yeast ordered proteins and IDPs involved in stress responses.

Stress pathway	^a^Total proteins/genes	Non-IDPs, Non-TFs	Non-IDTFs	^b^IDPs Non-TFs	^b^^,^^c^IDTFs
Heat shock	101	82	5	9	5
Nutrient adaptation	182	136	16	13	17
Osmotic stress	150	113	10	15	12
Oxidative stress	138	113	7	10	8

^a^ Data of total proteins (TPs) was taken from Kawakami et al. (2016) and from this work.

^b^ IDPs Non-TFs and IDTFs were identified with MOBIDB D_ratio ≥ 0.5.

^c^ Notice that, although the total of stress related IDTFs is 27, this table considers the number of IDTFs per stress pathway, which includes those IDTFs participating in more than one pathway.

**Fig 3:**
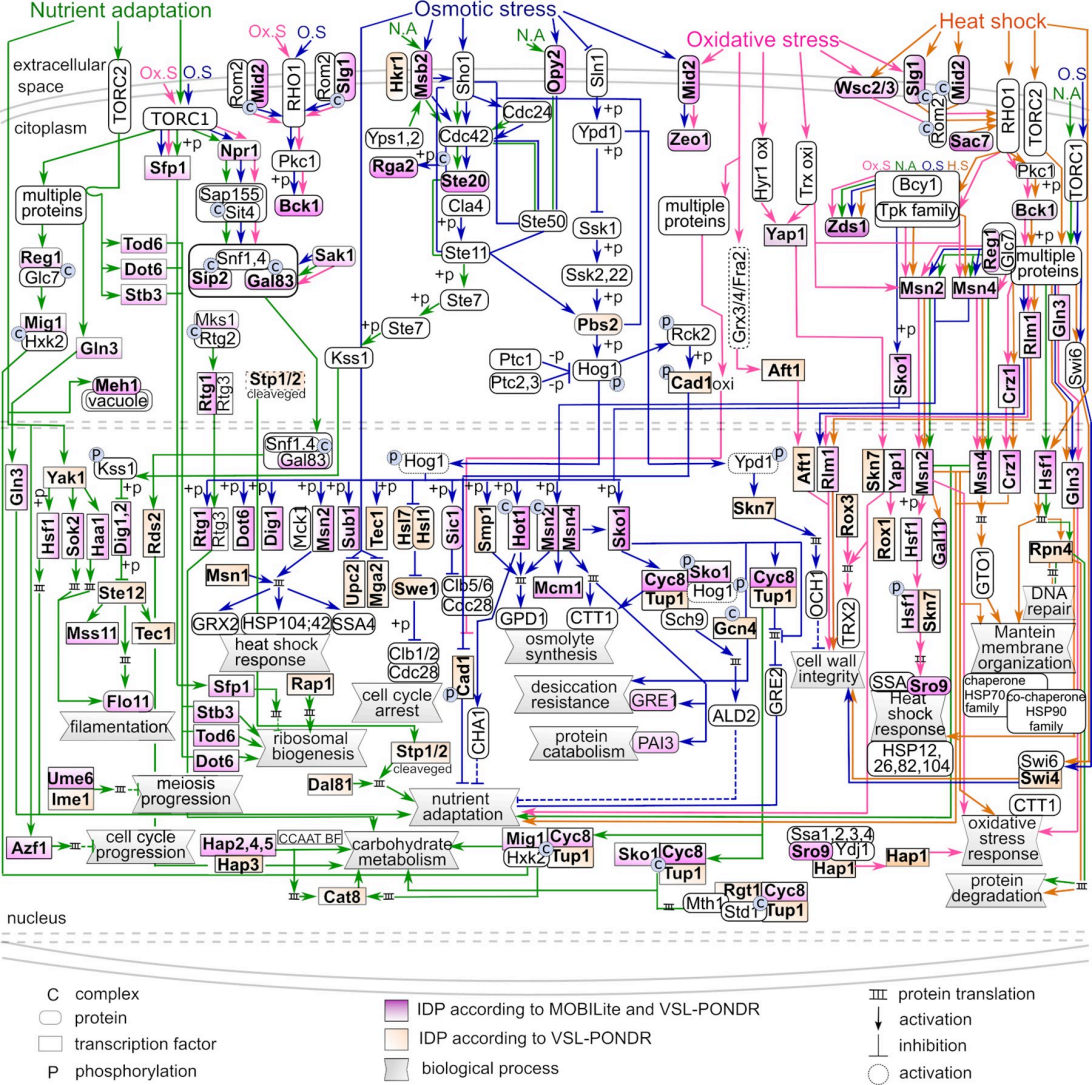
Stress signaling pathways in *S*. *cerevisiae*. Intrinsically disordered transcription factors (IDTFs) (boxes) and other signaling IDPs (ovals) identified with MobiDB are highlighted in violet. The nutrient adaptation, osmotic stress, oxidative stress and heat shock signaling pathways are marked with lines and arrows in green, blue, pink and orange, respectively. Signaling proteins showing lower structural disorder but filtered through VSL2 were also included in this figure (orange boxes and ovals). Stress response signaling pathways were modified from Kawakami et al. (2016).

**Fig 4:**
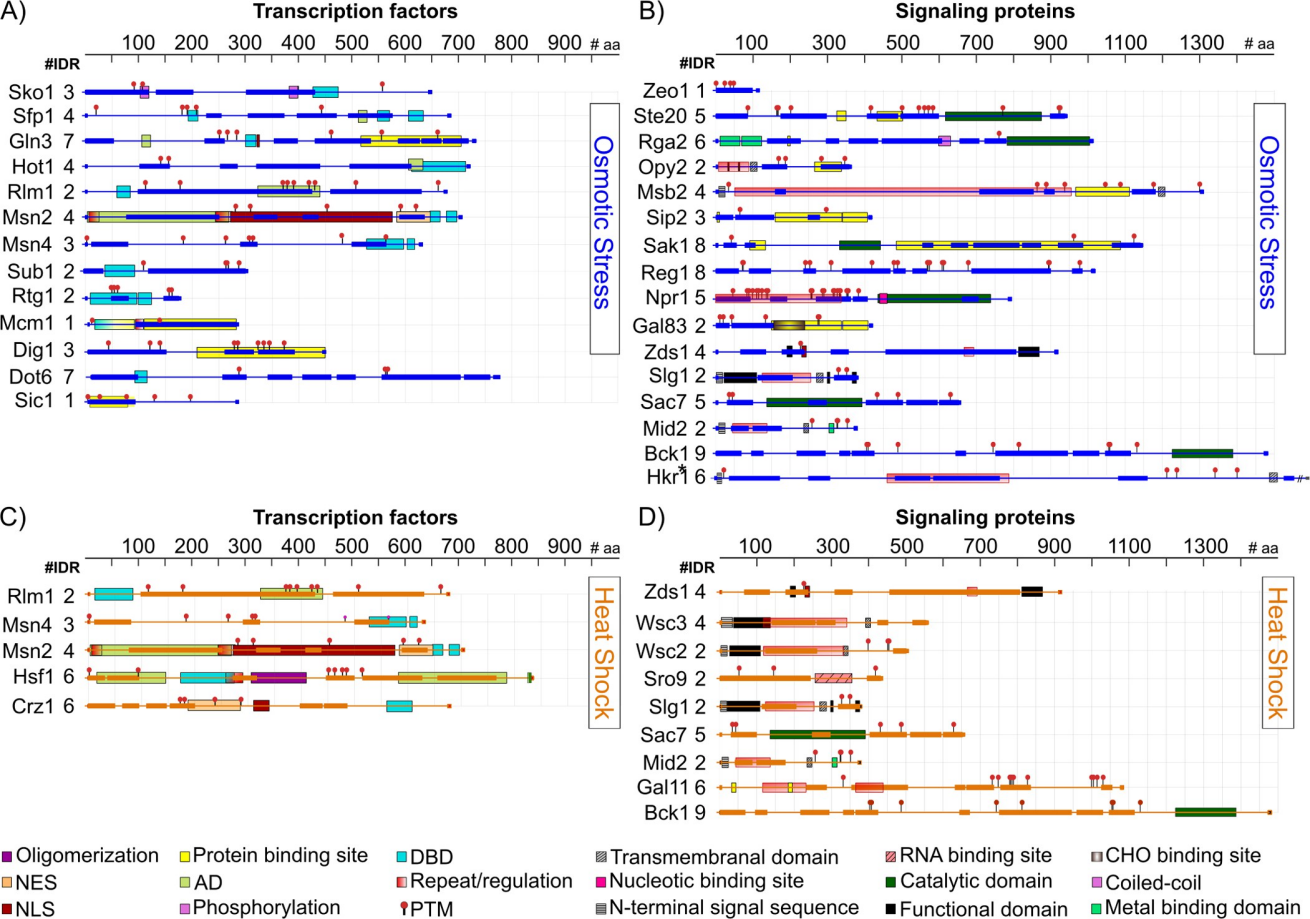
IDRs and functional domains in IDTFs and in signaling proteins involved in osmotic and heat shock response signaling pathways. (A and B) osmotic stress and (C and D) heat shock. The functional domains shown in this figure are those supported by experimental evidence. NES: Nuclear Export Sequence; NLS: Nuclear Localization Sequence; TAD: Transcription Activation Domain; DBD: DNA Binding Domain; PTM: Post-Translational Modification; CHO: Carbohydrate Binding Site. IDRs are showed as bars in osmotic stress (blue) and heat shock (orange). The rulers above each protein representation schemes indicate the number of amino acid residues.

**Fig 5:**
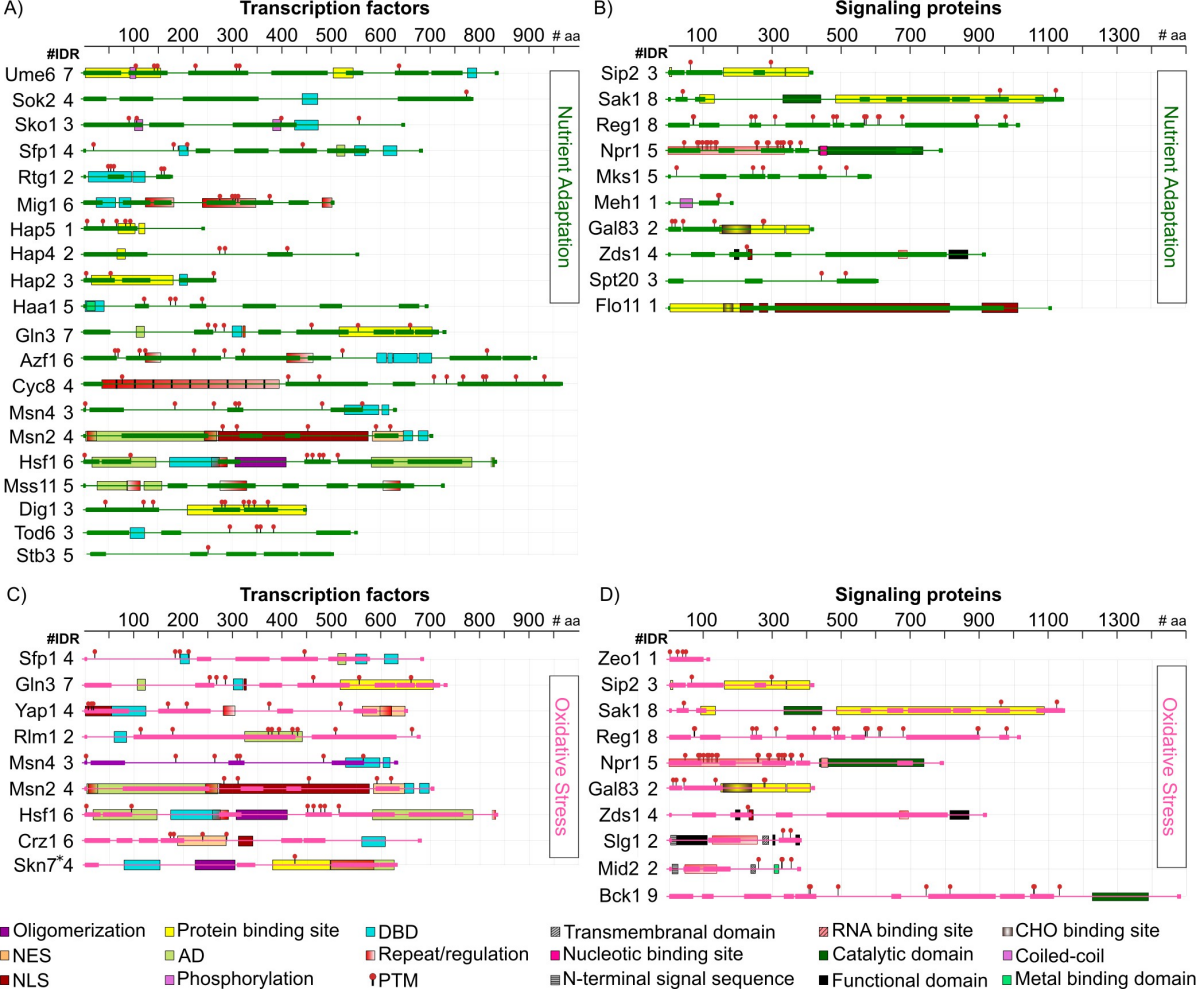
IDRs and functional domains in IDTFs and in signaling proteins involved in nutrient adaptation and oxidative stress response signaling pathways. (A and B) nutrient adaptation and (C and D) oxidative stress. The functional domains shown in this figure are those supported by experimental evidence. NES: Nuclear Export Sequence; NLS: Nuclear Localization Sequence; TAD: Transcription Activation Domain; DBD: DNA Binding Domain; PTM: Post-Translational Modification; CHO: Carbohydrate Binding Site. IDRs are showed as bars in nutrient adaptation (green) and oxidative stress (pink). The rulers above each protein representation schemes indicate the number of amino acid residues.

Structural disorder is considerably present in four of the best characterized stress response pathways, as shown in Figs 3–5. Keeping in mind that the number of signaling elements included for each pathway is limited by the available knowledge, we identified 27 signaling IDPs in the osmotic stress pathway, 30 in the signaling cascade leading to the nutrient limitation response, and 18 and 14 in the oxidative stress and heat stress signaling pathways, respectively. As expected, these numbers increase if we use VSL2-PONDR for this analysis (Fig 3).

### Intrinsically disordered signaling proteins in the osmotic stress response: The HOG pathway

The osmotic stress response signaling pathway is one of the best characterized in *S*. *cerevisiae*, due by the fact that this yeast thrives in a natural habitat where sugars could reach high concentrations [48, 49]. To cope with such osmolarity increase, yeast induce a diversity of adaptive strategies including the adjustment of transcription, translation and post-translational mechanisms, among others, leading to a transient growth arrest and rise in glycerol intracellular levels to balance cell water status [49]. Most of these adjustment responses are controlled by the high osmolarity glycerol (HOG) signaling pathway, a highly conserved pathway crosswise fungal species [50–52]. The core of this pathway is a mitogen activated protein kinase (MAPK) cascade, where the Hog1 MAPK plays a central role as responsible of the transcriptional response. The Hog pathway, in turn, crosstalk through some of its effectors with other signaling cascades, such as those controlling hypo-osmolarity response, cell wall biogenesis, and filamentous and invasive growth (S6 Fig) [53].

By tracking structural disorder throughout the known proteins participating in the osmotic stress signaling pathway, we found three osmostress sensors, Hkr1, Msb2, and Opy2 classified as IDPs according to the criteria described here (Figs 3 and 4B and S7 Table). Msb2 and Hkr1 are transmembrane proteins. These proteins are considered osmosensors of the Sho branch of this pathway, where Sho functions as a co-osmosensor, involved in the activation of the Hog1 MAPK. Msb2 contains four IDRs, three located at its extracellular domain, one at the amino-end, and the other two are nearby the region interacting with Opy2 that is necessary for the osmostress signal transduction. The remaining one is located at the cytoplasmic region, which associates with the scaffold protein Bem1 to recruit Ste20 to the plasma membrane [54]. Opy2 shows two IDRs, both localized to the cytoplasmic domain, which allow the interaction between the adaptor protein Ste50 and Ste11, targeting Ste11 to the plasma membrane, and leading to the activation of the Hog1 osmostress pathway. It is worth mentioning that Hkr1, structurally similar to Msb2 and also considered as an osmosensor, contains six IDRs, five in the extracellular region and one in the cytosolic segment. This last region also participates in the activation of Ste11 by Ste20 for their recruitment to the membrane through the Ste50 adaptor protein to stimulate the Hog1 cascade [53]. Among the Ste MAPKs, Ste20 qualifies as an IDP with five IDRs distributed throughout the protein. One of these IDRs overlap with the PXXP motifs, one binding to Bem1 and the other to the Sho1 osmosensor [55]. The next IDR towards the amino terminus includes the Cdc42 binding site located in Ste20 CRIB (Cdc42- and Rac-Interactive Binding) domain [55]. The protein-protein interactions occurring in these sites are essential for Ste20 function and, consequently, for its osmotransducing activity. A common target for the Sho1 and Sln1 branches in the Hog pathway is Pbs2, a MAPKK that specifically activates the Hog1 MAPK (Fig 3). Once Hog1 is turned on, it is translocated into the nucleus where controls the expression of multiple osmotic stress response genes through the activation of different TFs. Seventeen TFs seem to be activated by phosphorylation by the Hog1 kinase. Eleven exhibit a high score for structural disorder: Rtg1, Dot6, Dig1, Sub1, Sic1, Hot1, Msn2, Msn4, Sko1, Mcm1 and Cyc8, whereas Rtg3, Tec1, Hsl7, Hsl1, Tup1 and Smp1 present lower levels of disorder [53, 56] (Figs 3, 4A and 4B). Through the different TFs, Hog1 integrates the control of yeast stress responses in a promoter specific context.

The remarkable distribution of IDRs throughout the primary structure of Hog1 pathway TFs (Fig 4A and 4B) shows their potential involvement in transcriptional activation, protein-protein interactions, post-translational modifications and, in some cases, even in DNA binding (S7 Table). The relevance of these IDRs has been well documented for Msn2/Msn4, central players controlling general cellular stress responses [14, 24, 57] (Figs 4A and S8); whereas genetic and molecular analyses has demonstrated the participation of the IDRs in the function of other signaling proteins in this pathway (Pbs2, Hot1, Opy2, Sch9, and Sln3) [58].

### Intrinsically disordered signaling proteins participating in the heat shock response

When the temperature of the *S*. *cerevisiae* habitat increases to more than 35°C, yeast cells induce a transcriptional program that allows them to have the necessary protection, and the structural and metabolic adjustments to cope with this environmental strain [59]. This response to heat shock (HS) is primary modulated by two subsets of TFs, the heat shock transcription factor (Hsf) protein family, and by Msn2 and Msn4. While at present we still do not know the specific cues prompting the HSR signaling pathways, it has been proposed that, upon heat shock, cells activate the appropriate transcriptional response through the detection of misfolded protein accumulation that in turn triggers Hsf1 activation [60]. Even though, the activation of Msn2/4 by heat shock is not yet clear, Hsf1 and Msn2/4 are hyperphosphorylated in response to heat shock [61, 62]. As in the case of Msn2/4, Hsf1 presents IDRs throughout its sequence [63, 64] (Fig 4C and S8 Table). Interestingly, some IDRs overlap with the two AD domains that show a different response to heat shock located at its N- and C-terminal regions, while others involve phosphorylation sites, its DNA binding domain (DBD) and an oligomerization region [63] (Fig 4C). The IDR localized at Hsf1 C-terminus self-assembles into highly ordered structures showing amylogenic properties [64]. Additional TFs involved in yeast heat shock signaling pathways containing high structural disorder were identified, such as Rlm1 and Crz1 (Fig 3), where various IDRs were localized throughout their sequences (Fig 4C). Rlm1 shows two large IDRs, one which completely overlaps with its AD and contains hot spots for phosphorylation, whereas Crz1 contains six IDRs, one extending over its AD, and another partially overlapping a nuclear export sequence and containing three phosphorylation sites [65, 66] (Fig 4C). Rlm1 and Crz1 TFs play an essential role in the cell wall integrity pathway (CWI), which is activated by high temperature. The activation of Rlm1 requires the signaling proteins Wsc1, 2, 3, and 4, classified as IDPs [66, 67]. IDRs were also found in other signaling proteins participating in the heat shock signaling pathway, as Zds1, Sro9, Slg1, Sac7, Mid 2, Gal1, and Bck1 (Fig 4D).

### Intrinsically disordered signaling proteins involved in nutrient adaptation

Nutrients have acquired throughout evolution key regulatory roles in growth and developmental programs in all organisms due to their relevance as providers of essential elements to keep energy levels in cells. The study of different nutrient regulation pathways in *S*. *cerevisiae* has paved the way towards a better understanding of the control factors and their interactions involved in the more efficient use of the nutrients available in specific environments. Among the central transducers of yeast nutrient signaling and growth control are AMP-activated kinase (AMPK)/sucrose nonfermenting 1 protein (Snf1), involved in sensing depleted energy conditions; the GAL regulon, which is tightly regulated by the nature of the carbon source; the cAMP/PKA pathway, implicated in the perception and signaling of extracellular glucose and of other nutrients, through the G-Protein-Coupled Receptor (GPCR) and hexokinase systems; and the Target of Rapamycin Complexes (TORC1 and TORC2) pathways, whose core is TOR, a highly conserved protein kinase, whose activity is regulated by nutrient availability (amino acids and nitrogen sources) and cellular energy [68, 69].

The analysis of the signaling elements involved in different nutrient adaptation control pathways showed a remarkable high proportion of IDPs, including TFs (Figs 3, 5A and 5B, S9 Table). This is the case of Sfp1, a TF controlling expression, processing, and localization of ribosomal proteins, through its phosphorylation and interaction with TORC1, which promotes Sfp1 translocation into the nucleus. Sfp1 contains four IDRs, most of them in the C-terminal region, where one IDR overlaps with its DBD and with a recognized AD (Fig 5A). Npr1, a protein kinase phosphorylated by TORC1, is involved in the control of amino acid transport; this protein kinase presents five IDRs, overlapping with phosphorylation sites and with its catalytic domain (Fig 5B).

Sip2 and Gal83 are part of one of the Snf1 complex subunits; while Sip2 leads Snf1 localization into the cytosol, Gal83 directs this kinase to the nucleus, during glucose depletion [70–72]. Sip2 contains three IDRs, the longest one located at its N-terminal region (Fig 5B); whereas, Gal83 shows two IDRs, extending over its N-terminal domain (Fig 5B). Downstream of TOR, additional disordered signaling proteins and IDTFs were identified, such as Mks1 (Fig 3 and Fig 5B), Reg1, Mig1, Cyc8 (Fig 5A), Sko1 (Figs 4A and 5A), and Azf1 (Fig 5A). Gln3, a TF with seven IDRs, is essential in the activation of genes involved in the Nitrogen Catabolite Repression (NCR) system and responds to TORC1 and nitrogen limitation (Fig 5). Hsf1, a central TF in the heat shock response pathway, also responds to nutrient limitation through its phosphorylation by Snf1 [83]; in this way, a subset of Hsf1 gene targets is controlled by glucose starvation depending on Snf1 and the Hsf1 carboxyl-terminal activation domain, which overlaps with two of its six IDRs. Moreover, Hsf1 is also activated by phosphorylation through Yak1 kinase [73] (Fig 3), which shows structural disorder albeit in lower levels (Fig 3). Structural disorder was also predicted in Flo11, a GPI-anchored cell wall-associated glycoprotein (Figs 3, 5A and 5B) required for pseudohyphal and invasive growth, flocculation, and biofilm formation [74]. Other IDTFs controlling directly or indirectly Flo11 levels include Sok1, Haa1, Dig1 and Mss11 (Figs 3, 5A and 5B). Moreover, we identified IDTFs (Sfp1, Dot6, Stb3, and Tod6) involved in the control of yeast growth through the regulation of ribosome biogenesis [69] (Figs 3 and 5A). When yeast cells are exposed to a nitrogen limiting environment, their division is arrested in the G1 phase of the cell cycle, biosynthetic pathways are repressed, and catabolism is induced. Ume6 is an IDTF implied in coupling the control of meiosis with metabolic regulation triggered by nitrogen deprivation. Ume6 contains seven IDRs, three of them extending over protein binding regions and phosphorylation sites (Fig 5A). In yeast, the Hap complex functions as a positive and negative regulator and has been involved in the perception of oxidative stress, in the control of iron homeostasis, of nitrogen metabolism and of the balance between respiration and fermentation [75, 76]. Three subunits of this complex have a high number of disordered residues, some of them overlapping with protein binding or phosphorylation sites (Fig 5A) [93].

### Intrinsically disordered signaling proteins in the oxidative stress response

All organisms are exposed to ROS during the course of normal aerobic metabolism or following exposure to radical generating compounds; hence, diverse antioxidant mechanisms have been selected throughout evolution [77, 78]. The response to oxidative stress involves a global inhibition of protein synthesis and differential translational control of specific mRNAs, reprogramming of transcriptional gene expression, and of post-translational modifications of antioxidants proteins and metabolic enzymes, among other processes. All these to guarantee the reducing chemical potential necessary to maintain a redox balance and the restorative activities to overcome the stress situation [1, 79–82]. A major signaling module in this response is that controlled by the AP-1 like TF Yap1, a positive regulator of the yeast tolerance to H_2_O_2_, diamide and heavy metals [60, 83, 84] (Fig 3). Different regions of Yap1 are necessary for its functions, some of which contain structural disorder. This is the case of the cysteine rich domains (CRD), IDRs located at the N- and C-termini (n-CDR and c-CRD) and involved in the regulation of Yap1 nuclear localization and activity [85] (Fig 5C and S10 Table). This process requires that both CRDs undergo a conformational change from a disorder to an order structure, a rearrangement induced by the oxidation of the disulphide bonds located in the n-CRD and c-CRD segments [85, 86]. Because the association of Yap1 with the export receptor Crm1/Xpo1 requires the reduced cysteines in the CRDs, this conformational modification prevents this interaction, avoiding Yap1 exportation from the nucleus [87]. CRD disordered regions are also required for Yap1 transcriptional activity in response to a hyperoxidant environment produced by diamide or H_2_O_2_ [88, 89]. Under these stress conditions, the *in vivo* folding of Yap1 also requires its binding to Ybp1 (Yap-binding protein), involving both CRDs. Yap1 and Skn7 TFs participate in the same signaling pathway in response to oxidant stress (Fig 3). Even though Skn7 did not qualify as an IDP, it shows structural disorder in the phosphorylation receiver and the glutamine-rich C-terminal domains [90, 91].

Among the most interconnected signaling cascades are those participating in the response to heat and oxidative stress conditions. TFs containing substantial structural disorder, where these two responses converge are Hsf1, Msn2, Msn4, Crz1, Rlm1 and Gln3, all of them playing fundamental roles for an effective adjustment of yeast cells to these stressful environments (see Heat shock response, Figs 3, 4C and 5C and S10 Table). Hsf1 is phosphorylated in response to oxidative stress, most probably in one of its IDRs, because all phosphorylation sites are located in these regions (Figs 4C and 5C), and also interacts with Skn7, *in vivo* and *in vitro* [91]. Signaling proteins in the oxidative stress response pathway showing structural disorder, such as Mid2, Zeo1, Zds1, and Bck1, are also shared with other stress signaling routes (Figs 3 and 5D).

### Intrinsically disordered TFs as protein-protein interaction hubs in yeast stress responses

**Fig 6:**
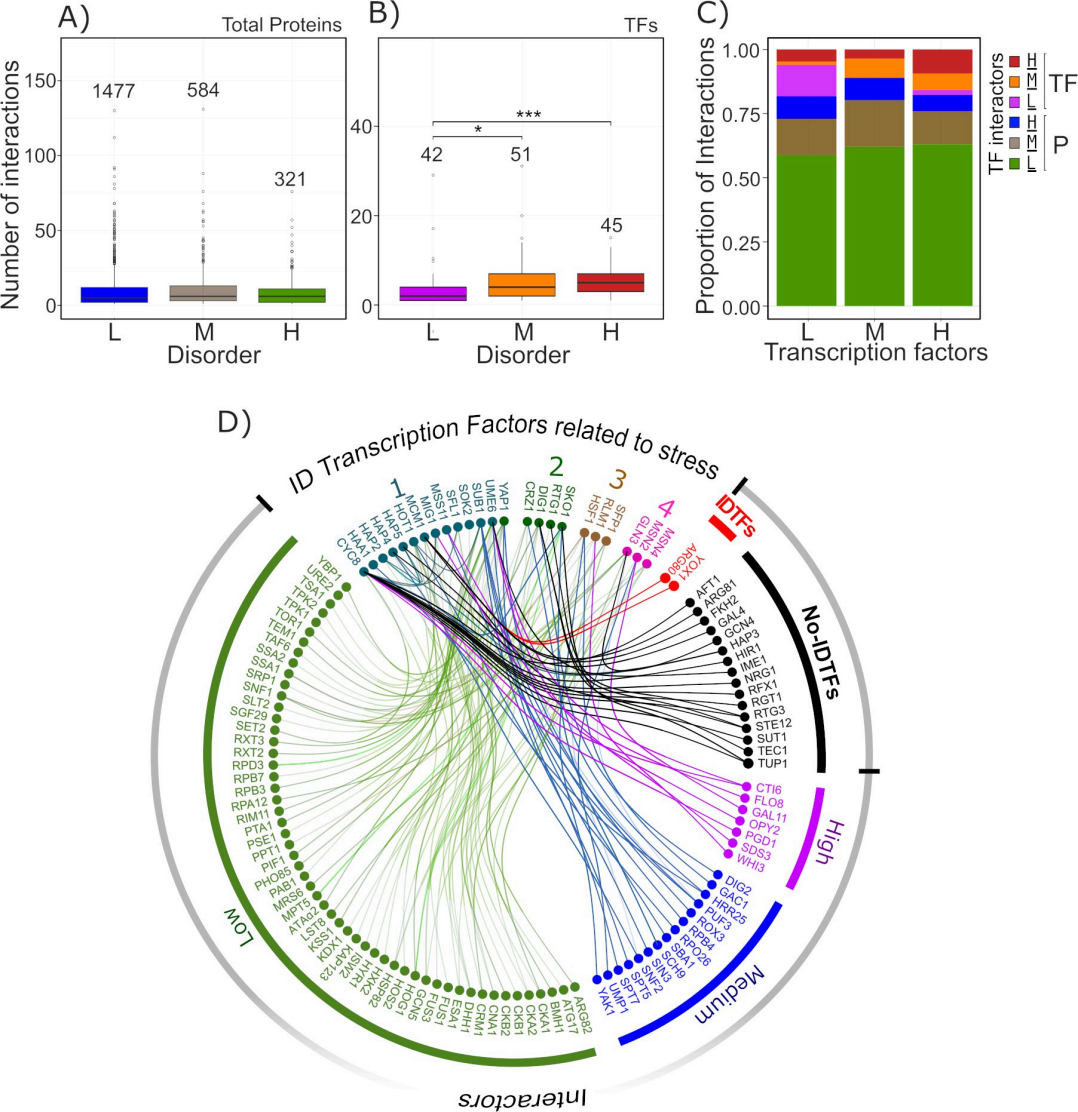
Protein-protein interaction network of *S*. *cerevisiae* IDTFs related to stress responses. (A) Number of interactions for total yeast proteins grouped in three disorder ratio ranges according to MobiDB: low (L) (0.1–0.29, blue box), medium (M) (0.3–0.49, brown box) and high (H) (D_ratio ≥ 0.5, green box). (B) Number of interactions for TFs grouped as described for (A): L (violet box), M (orange box) and H (red box). Asterisks denote differences evaluated with the Wilcoxon test * *p-*value ≤ 0.05 and *** *p-*value ≤ 0.001). The number of interactions was obtained from the node degree analysis. The outliers above 150 for (A) and 50 for (B) were not included to improve graph resolution; however, all data was used for the statistical analyses. (C) Proportion of interactions for TFs grouped according to their D_ratio range as described above. Each colour identifies the type of TF interaction: interactions between TFs (in violet, orange and red blocks) or between TFs and non-TFs proteins (in green, brown and blue blocks) classified by their disorder (Low, Medium and High, respectively). (D) Interaction network for IDTFs involved in yeast stress responses based on the protein-protein interaction analysis. The numbers above IDTF names represent the number of pathways where they participate (from 1 to 4). The connector line colours refer to the disorder level of the corresponding interactor. The external circle demarcates the different interactors, TFs were classified in IDTF (red lines) and non-IDTF (black lines), while proteins were categorized by their D_ratio (H, violet lines; M, blue lines; L, green lines).

The high assortment of IDPs interactions is a recognized characteristic for this set of proteins. For IDTFs, this property has been predicted in reported global analyses focused on IDPs [43, 92]. We performed here a similar analysis to get a closer view of the interaction potential of those yeast IDTFs involved in stress responses. The result of the evaluation of all yeast proteins showed no difference in the number of interactions among the proteins with different level of disorder (Fig 6A). However, in the sub-set of TFs, those with the higher level of disorder (D_ratio ≥ 0.5) show a larger number of interactions as compared to those with a lower D_ratio (Medium, D_ratio > 0.3–0.49; Low, > 0–0.3) (Fig 6B).

The identification of the TF interactors revealed that the highest proportion of the interaction partners are non-TF proteins with low disorder level, whereas the lowest proportion corresponds to those with high structural disorder. In contrast, for those partners identified as TFs, the proportion of interaction varies between 20% and 10%, considering TFs with low (L), medium (M), and high (H) structural disorder (Fig 6C). It is noteworthy that TFs with high level of disorder (H) interact with the utmost proportion of TFs also showing the highest D_ratio (Fig 6C). The known IDTFs participating in stress response signaling pathways exhibit higher number of interactions not only compared to the non-disordered TF involved in these responses, but also against those IDTFs not related to stress (S9 Fig). A closer assessment of the structure and function of the stress IDTFs interactors showed their interplay with either other IDTFs but also with non-disordered TFs, and with non-TF proteins, although most of them are components of stress signaling pathways (Fig 6D). The high connectivity of stress IDTFs exhibits their role as hub proteins in interaction networks, and the identity of their interactors show that stress response processes are considerably interconnected, highlighting the biological significance of this intercommunication. The outcome of this particular analysis strengthens the relevance of IDR participation in protein-protein interactions.

Many of different stress intrinsically disordered signaling proteins described in the previous sections are network hubs (S8 Fig). Among them, Cyc8, Dig1, Hot1, Sub1, Ume6, and Yap1 stand out because of the number of identified interactors (S8 Fig). Cyc8 is a hub participating in nutrient adaptation and osmotic stress response pathways; Dig1, Sub1, and Hot1 are components of the osmotic response signaling pathway; Ume6 is a bifunctional transcriptional regulator involved in the control of the nutrient deprivation response; and Yap1 is a key IDTF in the signaling pathway controlling the oxidative stress response. Other IDTFs for which various interactors have been identified are Hsf1, Gln3, Hap2, 4, and 5, Mig1, Crz1, Sko1, and Mcm1 (S8 Fig).

### Yeast IDP-TFs have a high probability to undergo a liquid-liquid transition phase

The interaction networks of different macromolecules in cells lead to the formation of condensates, which need a high number of connections to be stable. This property is conferred by the multivalence and the flexibility of the macromolecules involved [93, 94]. IDPs are macromolecules that possess these characteristics, similar to polymer-like molecules, with the potential to assemble adaptable and dynamic networks. A wealth of information supports an important role for IDPs and IDRs in the formation of intracellular condensates, allowing the coacervation of adaptable macromolecule assemblies with diverse morphologies and dynamics. Some of these intra cellular condensates correspond to proteinaceous membrane-less organelles (PMLO) [95–97]. Recently, liquid-liquid transition phase separation (LLPS), mediated by IDPs, has been described as crucial for the organization of different cellular activities [95]. In cells, the relevance of LLPS is represented by PMLOs, which allow the separation in time and space of some cellular functions [19]. PMLOs are the product of protein-protein and/or protein-nucleic acid (DNA and/or RNA) interactions; their generation is an extensively regulated and reversible process, highly influenced by the cellular environmental condition and by the cellular components present [97, 98]. LLPS also leads to the formation of granules or bodies, whose appearance, in some cases is triggered by stressful conditions [19, 97]. LLPS is favored by changes in the cell environment such as fluctuations in osmolarity, temperature, pH, and also by changes in the macromolecules involved, such as post-translational modifications which alter their charge distribution thus promoting electrostatic interactions. IDPs and IDRs are enriched in positively and/or negatively residues, and they may include repetitive sequences, properties offering flexibility and multivalency, that in turn allow weak multivalent interactions, responsible for the dynamics of this phenomenon.

**Fig 7:**
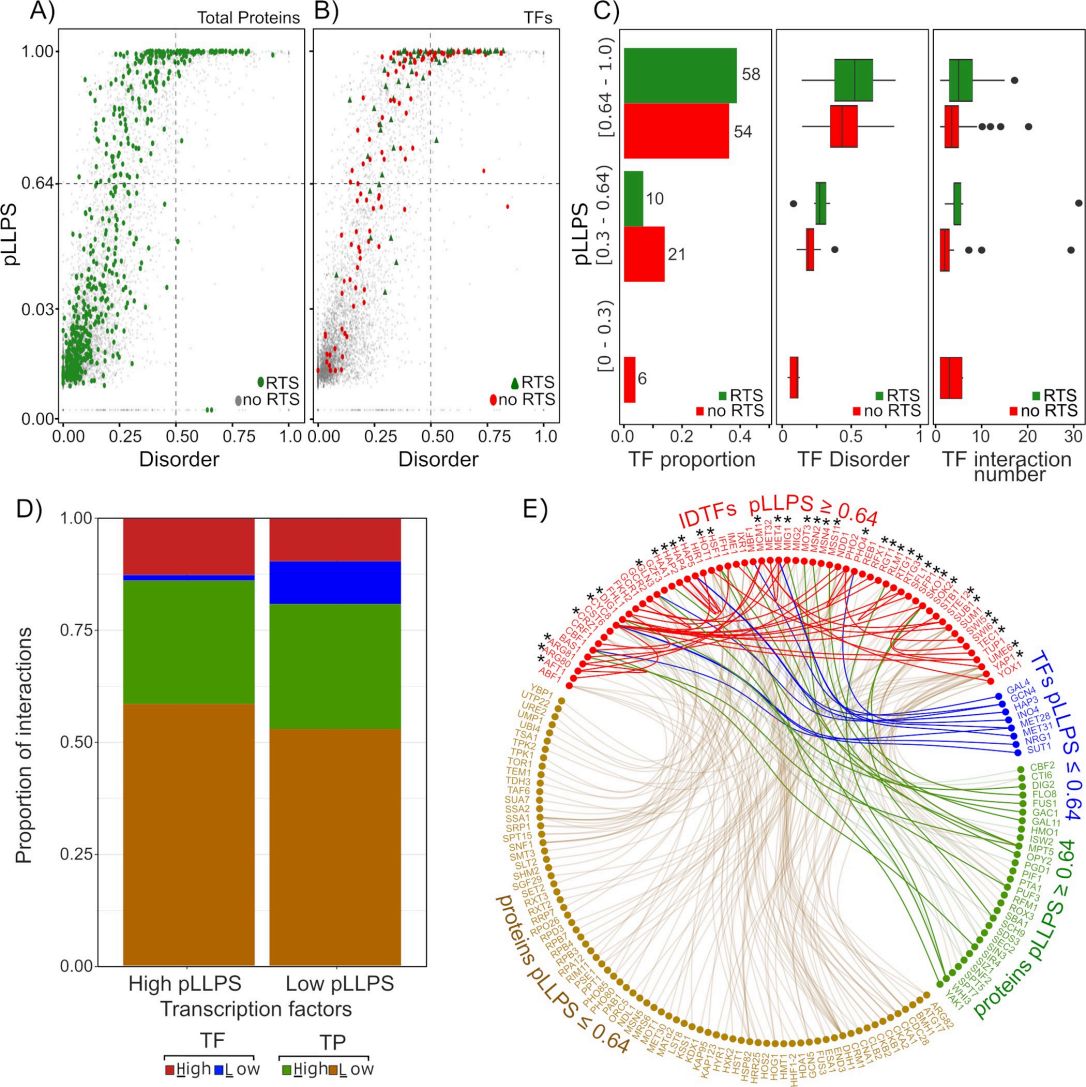
Liquid-Liquid Phase Separation (LLPS) propensity of yeast TFs. LLPS probability index (pLLPS) was determined using FuzPred, and disorder probability index was obtained using MobiDB. (A) Relation between pLLPS index and disorder ratio of the complete yeast proteome (light grey circles). Green circles represent proteins related to stress responses (RTS). Vertical and horizontal discontinue gray lines represent the cutoff values for disorder ratio (≥ 0.5) and for pLLPs (≥ 0.64), respectively. (B) Relation between pLLPS index and disorder ratio for TFs. Red circles and green triangles represent TFs no related to stress (no RTS) and RTS, respectively. (C) Distribution of TFs considering their pLLPS index, and its relation to their disorder ratio, number of interactions and participation in stress responses. (D) Interaction proportion for TFs according to their pLLPS index. Interaction proportions between TFs with high (≥ 0.64) and with low pLLPS (≤ 0.64) are represented in red and blue blocks, respectively, whereas proportions between TFs and proteins (TP) with high pLLPS (≥ 0.64) and low pLLPS (≤ 0.64) are represented with green and brown blocks, respectively. (E) Interaction network for IDTFs with pLLPS ≥ 0.64 (in red) with: TFs showing pLLPS ≤ 0.64 (in blue), proteins with pLLPS ≤ 0.64 (in brown) and proteins with pLLPS ≥ 0.64 (in green). IDTFs related to stress are indicated with an asterisk. The connector lines colour corresponds to the pLLPS index of the corresponding interactor.

These observations prompted us to examine the potential of yeast TFs for LLPS. We found, using FuzPred and MobiDB, that 21.3% of total yeast proteins showed high probability of undergoing LLPS (≥ 0.64) (Fig 7A). Interestingly, when TFs were sorted out, the analysis revealed that more than 65% have high LLPS propensity levels (≥ 0.64) (Fig 7B, and S3 Table).

A broader assessment, including the disorder ratio and interactions of TFs, showed that TFs with the highest pLLPs index grouped with the ones with high disorder ratio (Fig 7B and 7C). This analysis showed that TFs with the highest pLLPS tend to present higher number of interactions than those with lower pLLPS (Fig 7C). When we considered the total set of proteins related to stress (RTS), 30% showed high pLLPS, and from them 23% contain a disorder ratio < 0.5, whereas 7% are highly disordered (Fig 7C and S11 Table). We found that 85% of stress related TFs exhibit high pLLPS, with a disorder ratio ≥ 0.5 and higher number of interactions when compared to non-related to stress TFs, while the rest (pLLPS ≤ 0.64) show a disorder ratio between 0.25 and 0.5 (Fig 7B and 7C). The fact that highly disordered proteins, including TFs, show high propensity to undergo LLPS may not be surprising; however, the high proportion of yeast TFs with these characteristics involved in stress responses is noteworthy.

A common observation in diverse organisms has been the formation of stress granules triggered by adverse environmental conditions, whose composition is variable depending on the organism and on the stress condition [9]. The participation of stress TFs in the formation of some of these stress bodies has been reported, as is the case of human Hsf1 TF, which initiates the generation of nuclear stress bodies by interaction with pericentric tandem repeats leading to an increased transcription of large heterochromatic regions in response to stress [99, 100]. The potential of stress IDTFs and other signaling IDPs to undergo LLPS, and their physicochemical characteristics allowing them to establish diverse protein and nucleic acid interactions, point them as components of stress bodies or condensates, even though its role in the control of transcription is still an open question. Recently, it was demonstrated that human YAP (Yes-associated-protein) TF is part of liquid-like condensates formed in the nucleus after a few seconds of imposing hyperosmotic stress. YAP condensates include other TFs and co-activators, and its formation precedes transcriptional up-regulation of YAP-specific proliferation genes [23]. YAP intrinsically disordered AD was shown to be necessary for YAP condensates formation, and for downstream signaling in response to hyperosmotic stress [23]. These observations support the idea that mechanisms implicating TF condensates, compartmentalizing additional co-TFs and multiprotein complex as Mediator, enable the fine control of the expression of RNA PolII stress transcripts.

Our analysis indicates that transcription factors with high probability for LLPS interact with a high proportion of proteins with no recognized TF properties, from which a higher proportion corresponds to those with low probability for undergoing phase separation (Figs 7D and S10). As expected, TFs also associate to other TFs; in this case, TFs with high probability for LLPS have an evident higher proportion of interactions with those having high propensity to experience LLPS (Fig 7D and 7E). Regarding TFs with low probability for LLPS, they display similar proportion of interactions with non-TF proteins as that shown by TFs with high pLLPS. Also notice that TFs showing low pLLPS may interact in similar proportions to TFs with low or high pLLPS (Figs 7D, S10 and S11). These results suggest that the formation of TF ensembles driven by intracellular liquid-liquid phase separation is carefully balanced and selective to avoid non-desired interactions or networking. This information also alludes to the relevance of the type of protein-protein and/or protein nucleic acid interactions for the formation of this kind of protein condensates. Even though, the interactions in these complex ensembles may not be specific, the distribution of charge and aromatic/hydrophobic residues throughout the involved macromolecules seems to play an important role, properties that have been conserved along evolution in most if not all participants of these macromolecular communities.

## Concluding remarks

Throughout evolution diverse strategies of adaptation have been selected in the different domains of life, by adjusting their metabolism, physiology, and developmental programs, allowing the existence of a great diversity of lifestyles in so different environments. Hence, the intricacy of these processes and of the mechanisms involved in the responses to cope against environmental fluctuations, require plentiful versatility, leading to the selection of proteins with high structural flexibility in organisms of all taxa.

In this work, we present a comprehensive *in silico* analysis of the structural disorder in the budding yeast proteome where, by using data bases that include experimental information, we showed that this property is enriched in essential processes such as transcriptional regulation by RNA polymerase II, chromatin remodeling, RNA-mediated transposition, and in the responses to abiotic stimuli. This report highlights the fact that IDRs are more frequent in TFs than in all yeast proteins, among which stand out those involved in the stress response signaling pathways over TFs participating in other routes. IDTFs are highly represented in the osmotic and nutrient stress response pathways, and in lower level in the oxidative and heat stress signaling cascades. It is noteworthy the presence of structural disorder in many of the signaling proteins involved in this communication networks, where various IDRs are in protein regions critical for their perception function. The involvement of structural disorder in transcriptional activation, protein-protein interactions, post-translational modifications, and in DNA binding of TFs in the different stress pathways is evident given the remarkable distribution of IDRs throughout their primary structure, particularly, in those regions related with such functions. Also, from the analysis in this work, it is evident the larger number of protein-protein interactions among stress related TFs showing the higher level of disorder, when compared to those with lower disorder or not linked to stress, underpinning their function as hubs in the stress response signaling pathways. The interaction competence of IDTFs is in consonance with the predicted ability of IDPs to undergo LLPS, that in many cases has been experimentally demonstrated. This phenomenon is a dynamic process influenced by the cellular milieu, as it might be the structure of IDPs, in some instances triggered when cells are exposed to adverse environments. We found that TFs with high pLLPs index correspond to those with high disorder ratio, and that high percentage of stress IDTFs present high propensity to undergo phase separation compared to non-stress TFs.

## Supporting information

S1 Fig Methodology scheme followed for sequence selection and disorder structural analyses of the yeast proteome. (TIF)

S2 Fig Methodology for GO enrichment and network analysis. Methodology scheme followed for functional classification of yeast IDPs (GO and pathway enrichment).
(TIF)

S3 Fig Relation of disordered ratio determined by MobiDB, MobiDB-Lite, IUPred2 and VSL2. (A) Correlation of disorder ratio between the predictor programs used. (B) Venn diagram showing the distribution of IDP amount according to the D_ratio obtained with the different disorder prediction programs.
(TIF)

S4 Fig GO enrichment analysis in biological processes for *S*. *cerevisiae* IDPs identified according to MobiDB, IUPred2 and VSL2. (TIF)

S5 Fig Number of IDPs associated to different cellular components. The number of IDPs was obtained according to MobiDB, IUPred2 or VSL2. Proteins with only one reported cellular localization are represented with red bars (unique), while proteins assigned to more than one cellular compartment are represented with gray bars (diverse).
(TIF)

S6 Fig Enrichment of metabolic pathways. KEGG graph of MAPK signaling pathways rendered by Pathview. Orange boxes indicate the proteins considered as IDPs according to MobiDB ≥ 0.5.
(TIF)

S7 Fig GO enrichment analysis of IDPs involved in metabolic and stress pathways using three disorder predictor programs. (TIF)

S8 Fig Networks for *S*. *cerevisiae* IDTFs involved stress response pathways. Each TF is shown at the center of each node, whereas intermolecular interactions between nodes are represented with lines. Different attributes were defined to integrate all data in the graph model: no-TF proteins are indicated with squares and TFs with diamonds. The disorder ratio is shown in a color gradient from 0.5 (yellow) to 1.0 (violet), as indicated at the right bottom. Line colors indicate the stress pathway involved. The analysis and graph were done using Cytoscape 3.8.0.
(TIF)

S9 Fig Interaction number for yeast proteins and TFs involved in stress response pathways. (A) Number of interactions of yeast proteins related to stress (RTS) or not (no RTS). (B) Number of interactions of yeast TFs RTS or no RTS. Blue and green boxes correspond to ordered proteins and IDPs, respectively.
(TIF)

S10 Fig Interaction network of TFs according to their pLLPS index. TFs and their interactors were classified according to their propensity to undergo liquid-liquid phase separation: High pLLPS ≥ 0.64 and Low pLLPS ≤ 0.64. The connector lines colour is based on the pLLPS index of the corresponding interactor as indicated.
(TIF)

S11 Fig Propensity of yeast IDTFs related to stress response to undergo liquid-liquid phase separation. The pLLPS and disorder probability indexes were determined using FuzPred and MobiDB programs, respectively. Each graph represents the relation of pLLPS index and D_ratio of all yeast proteome (grey circle) and of stress related TFs (red circles) for the different stress pathways: (A) heat shock, (B) osmotic, (C) oxidative, (D) nutrient adaptation and (E) ion homeostasis. Vertical and horizontal discontinue lines represent the cutoff values of pLLPs (≥ 0.64) and D_ratio (≥ 0.5), respectively.
(TIF)

S1 Table Raw data from structural disorder analyses of *S*. *cerevisiae* proteome. (PDF)

S2 Table Clustering of GO categories from REVIGO, used for construct TreeMap of Fig 1. (PDF)

S3 Table Number of IDPs identified by each predictor and pLLPs index. (PDF)

S4 Table Number of IDRs of *S*. *cerevisiae* proteome according to MobiDB. (PDF)

S5 Table IDP KEGG enrichment using clusterProfiler including the stress pathways. (PDF)

S6 Table Distribution of IDTFs in stress response pathways. (PDF)

S7 Table Yeast IDPs involved in osmotic stress response. (PDF)

S8 Table Yeast IDPs involved in heat shock stress response. (PDF)

S9 Table Yeast IDPs involved in nutrient adaptation. (PDF)

S10 Table Yeast IDPs involved in oxidative stress response. (PDF)

S11 Table Yeast proteins organized by stress pathways, according to their range of disorder ratio, determined with four disorder predictor programs and FuzPred. (PDF)
