# Supplementary figures and images for: Intrinsically disordered signaling proteins: Essential hub players in the control of stress responses in Saccharomyces cerevisiae
Source: PLoS One. 2022 Mar 15;17(3):e0265422. doi: 10.1371/journal.pone.0265422 (PMC8923507; doi:10.1371/journal.pone.0265422)

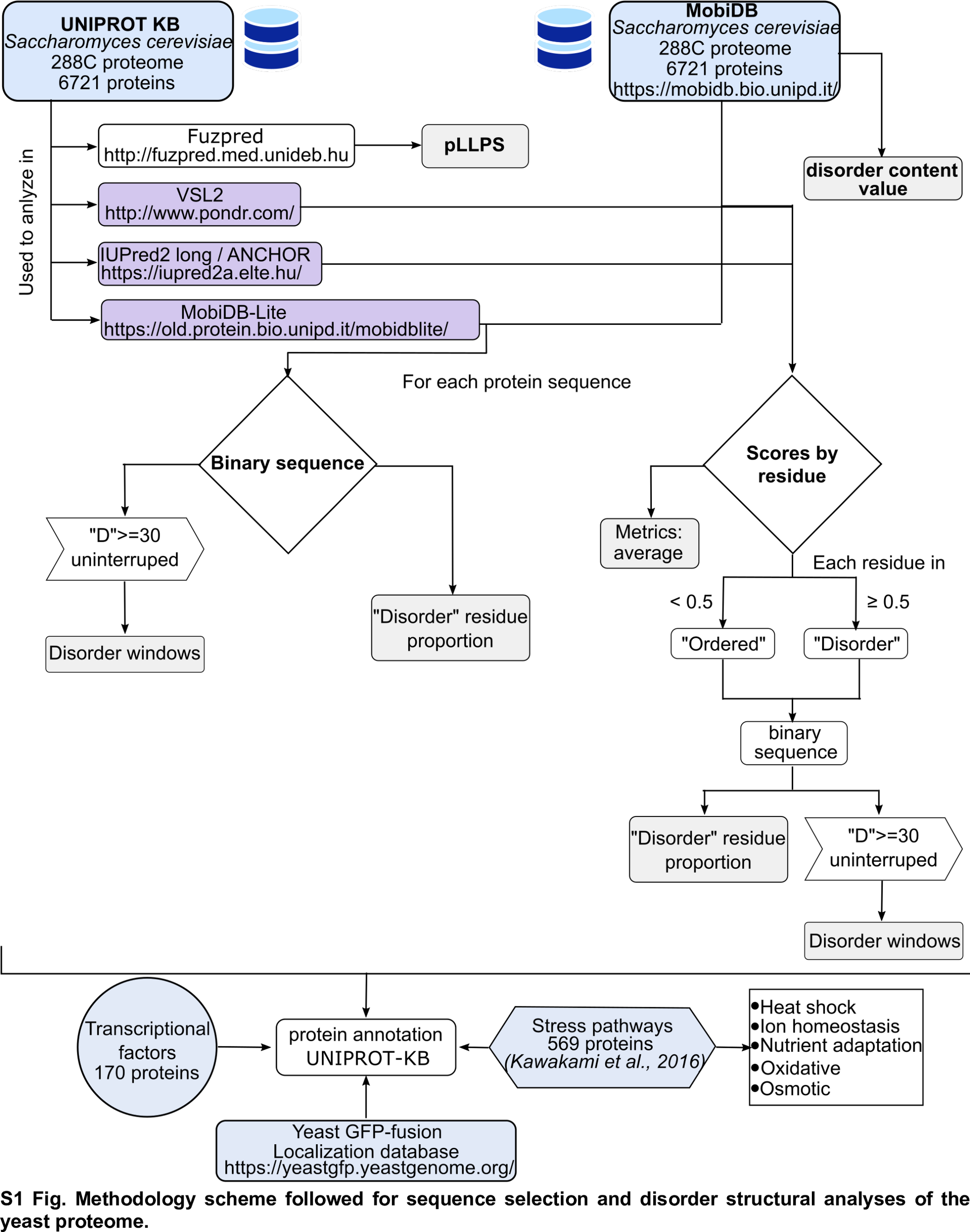

Supplement: S1 Fig — (TIF) [file pone.0265422.s001.tif]

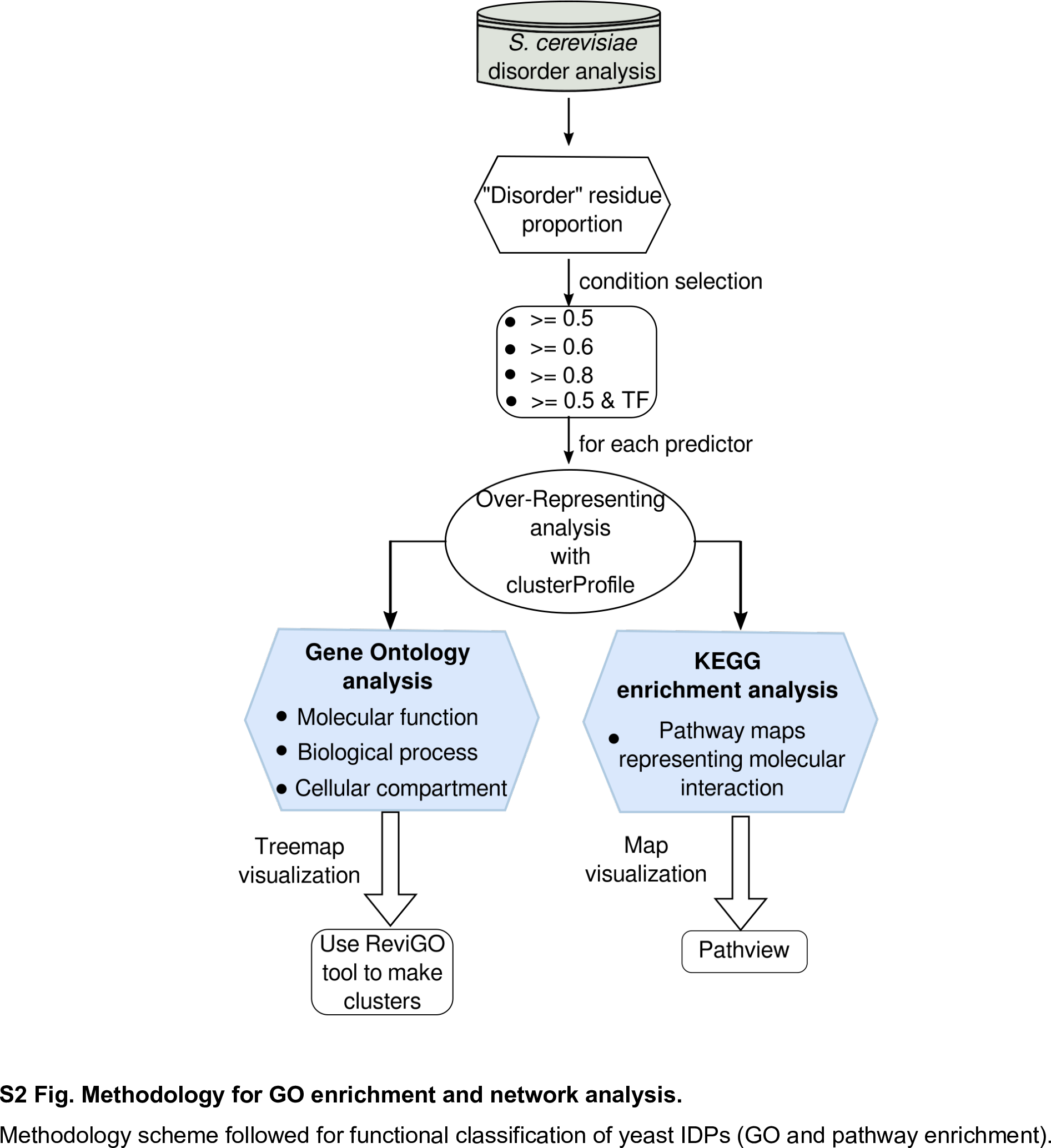

Supplement: S2 Fig — Methodology scheme followed for functional classification of yeast IDPs (GO and pathway enrichment). (TIF) [file pone.0265422.s002.tif]

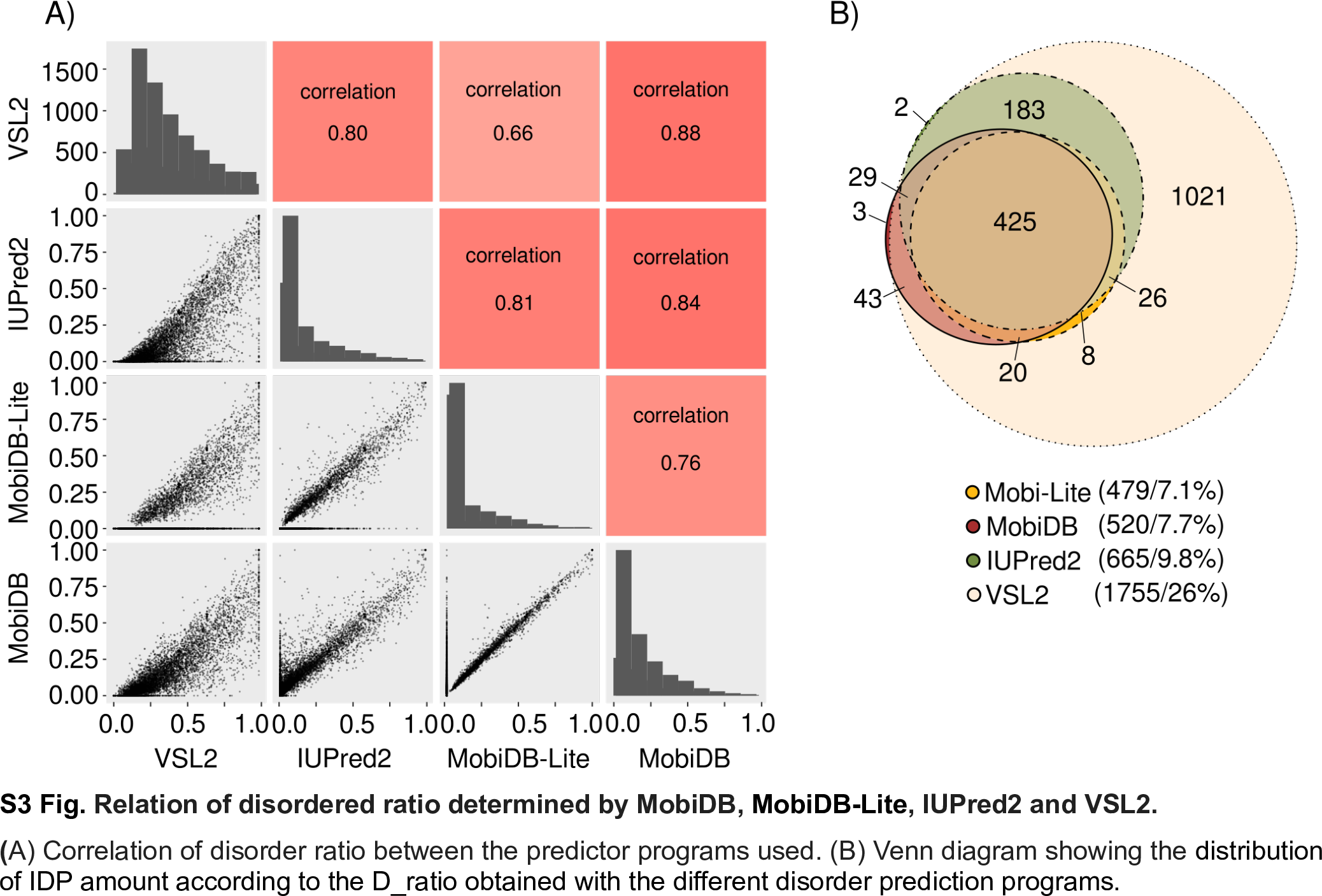

Supplement: S3 Fig — (A) Correlation of disorder ratio between the predictor programs used. (B) Venn diagram showing the distribution of IDP amount according to the D_ratio obtained with the different disorder prediction programs. (TIF) [file pone.0265422.s003.tif]

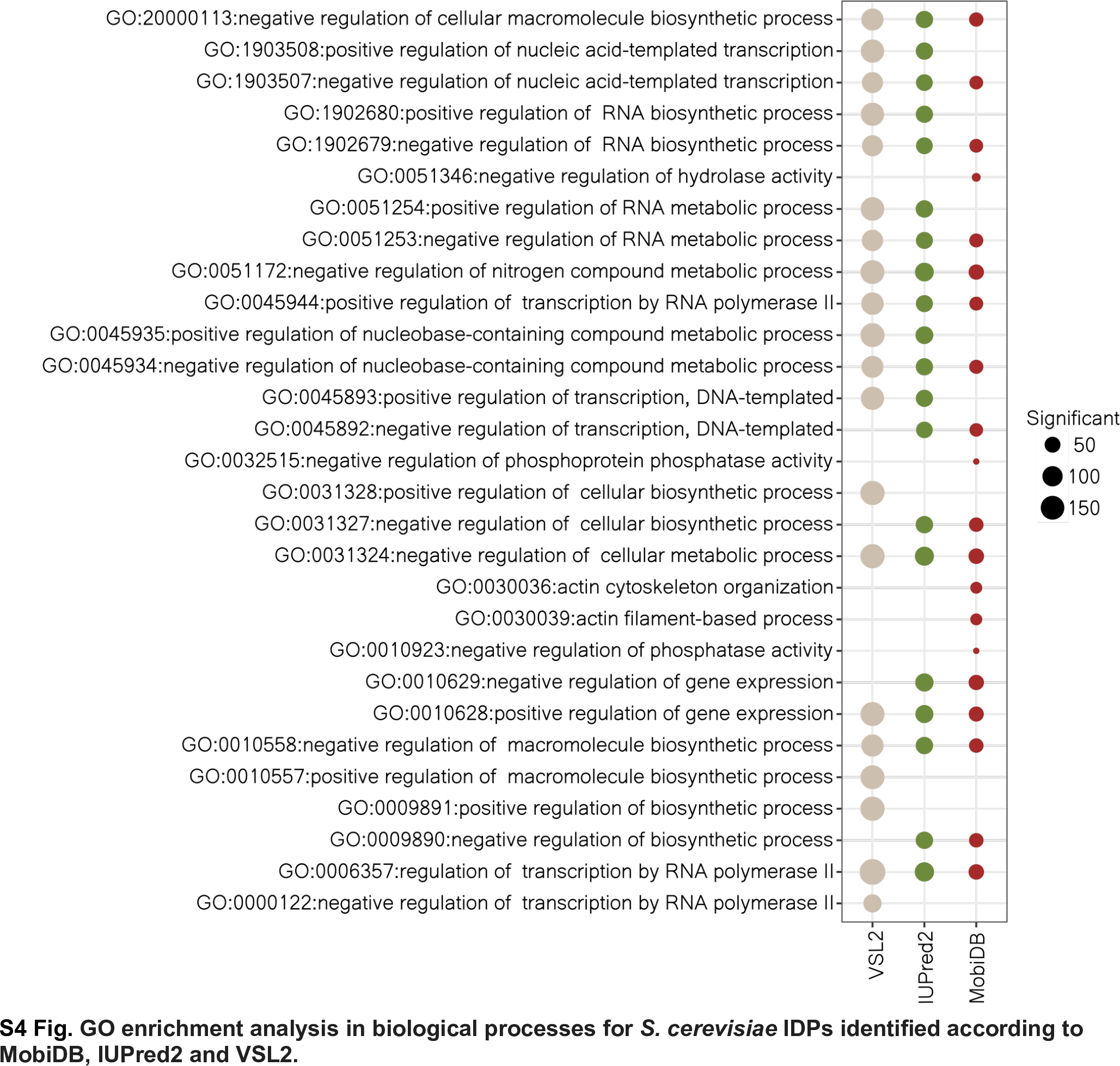

Supplement: S4 Fig — (TIF) [file pone.0265422.s004.tif]

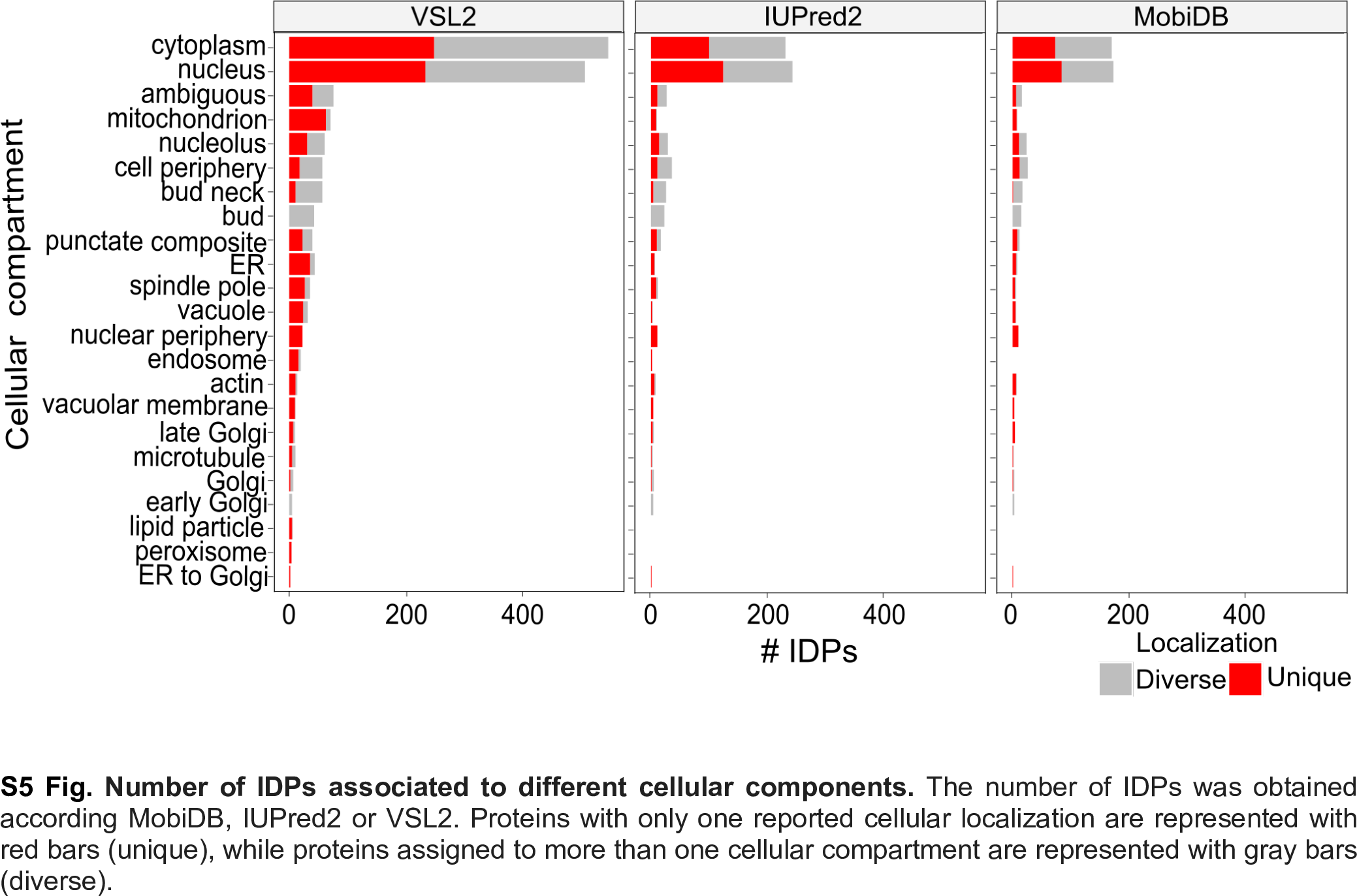

Supplement: S5 Fig — The number of IDPs was obtained according to MobiDB, IUPred2 or VSL2. Proteins with only one reported cellular localization are represented with red bars (unique), while proteins assigned to more than one cellular compartment are represented with gray bars (diverse). (TIF) [file pone.0265422.s005.tif]

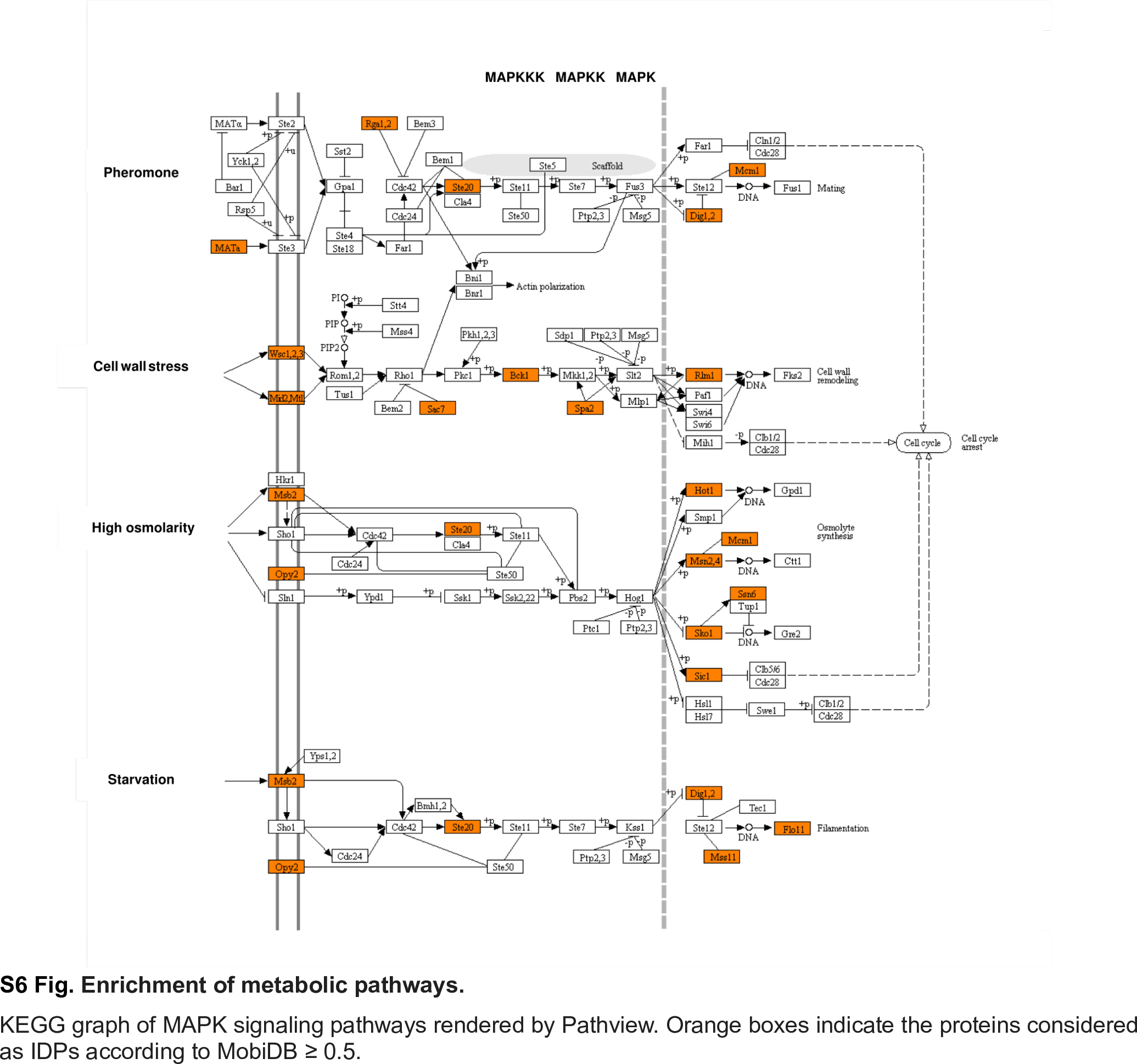

Supplement: S6 Fig — KEGG graph of MAPK signaling pathways rendered by Pathview. Orange boxes indicate the proteins considered as IDPs according to MobiDB ≥ 0.5. (TIF) [file pone.0265422.s006.tif]

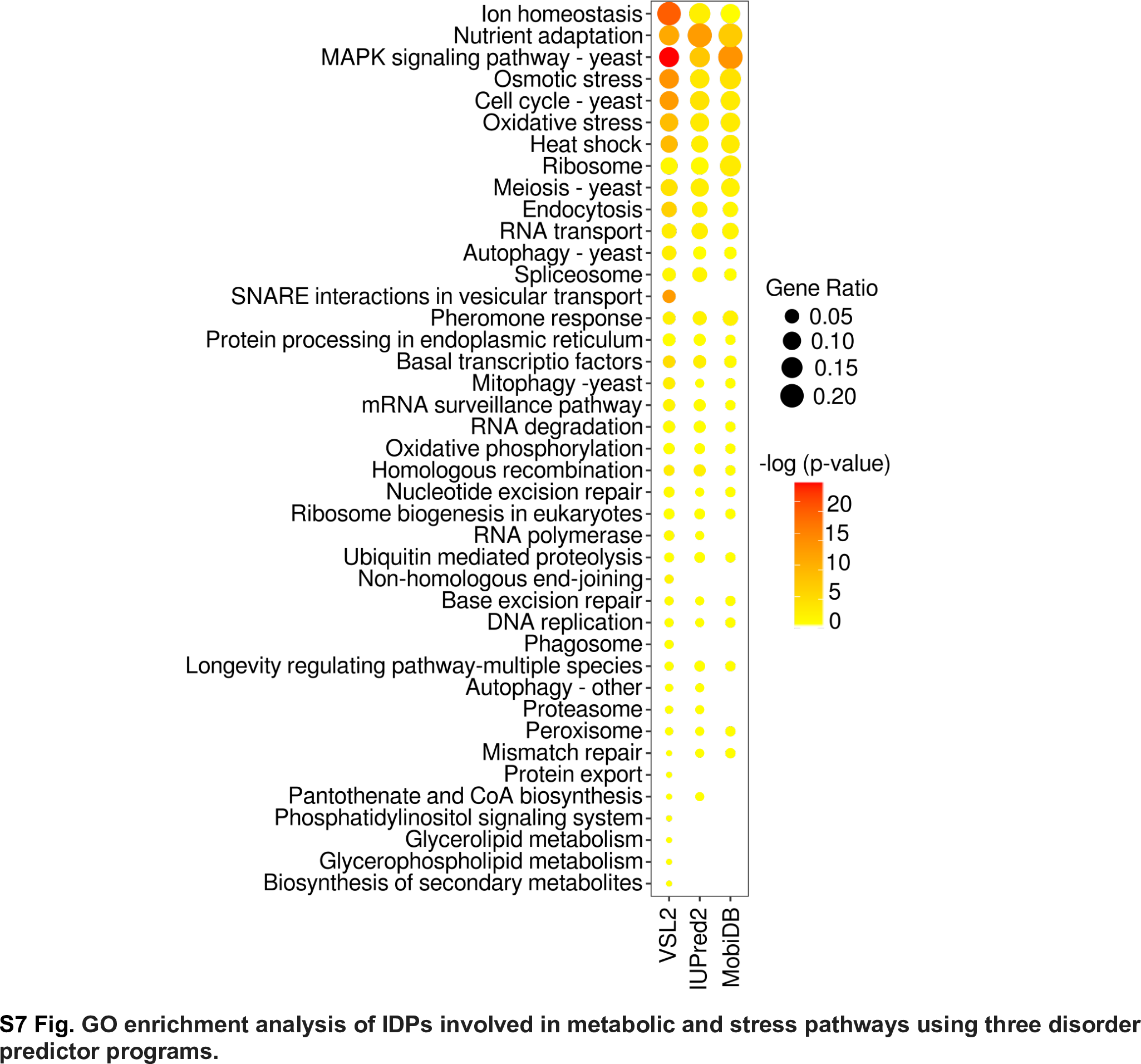

Supplement: S7 Fig — (TIF) [file pone.0265422.s007.tif]

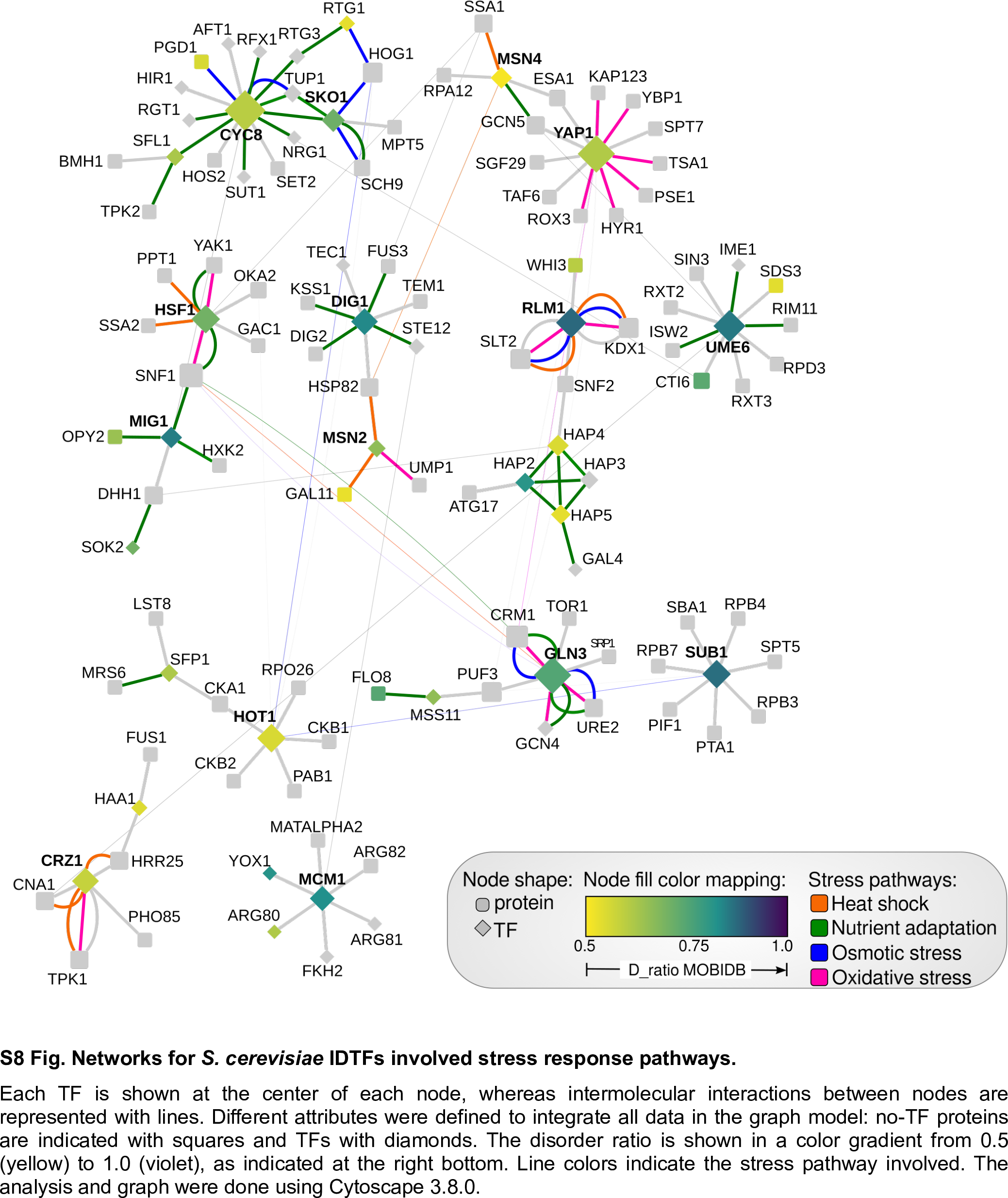

Supplement: S8 Fig — Each TF is shown at the center of each node, whereas intermolecular interactions between nodes are represented with lines. Different attributes were defined to integrate all data in the graph model: no-TF proteins are indicated with squares and TFs with diamonds. The disorder ratio is shown in a color gradient from 0.5 (yellow) to 1.0 (violet), as indicated at the right bottom. Line colors indicate the stress pathway involved. The analysis and graph were done using Cytoscape 3.8.0. (TIF) [file pone.0265422.s008.tif]

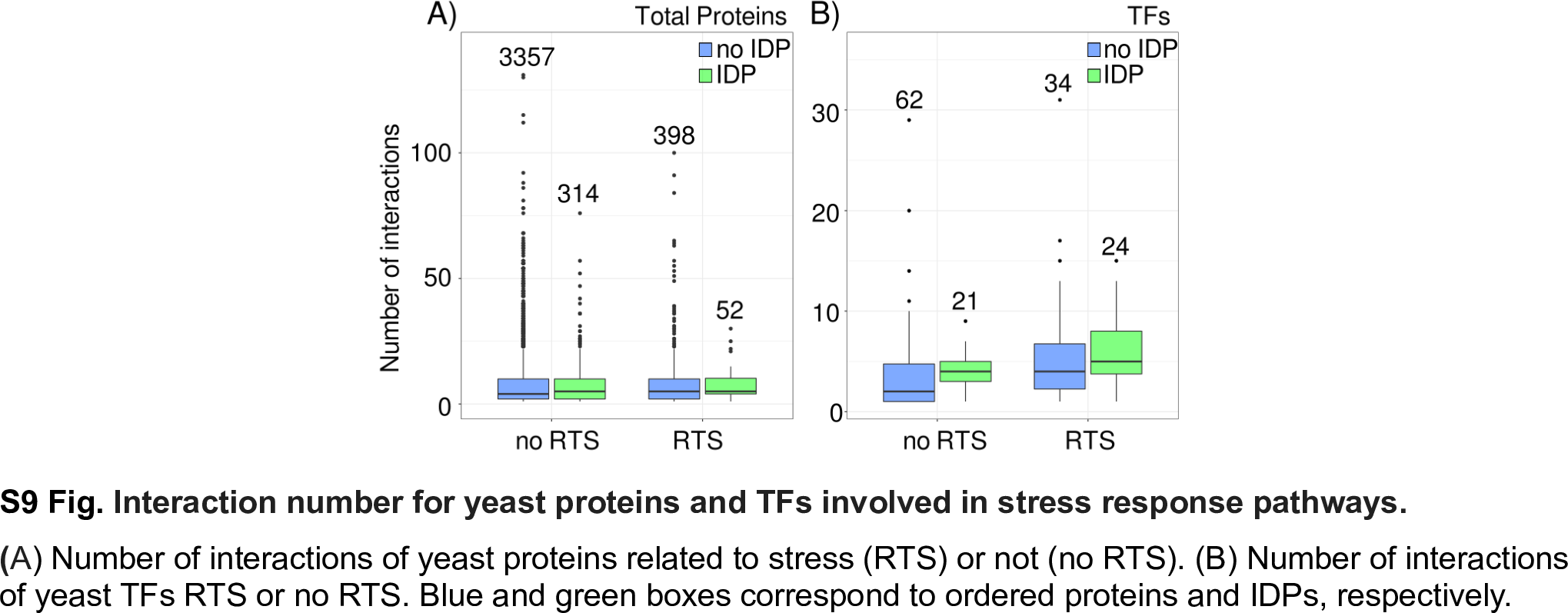

Supplement: S9 Fig — (A) Number of interactions of yeast proteins related to stress (RTS) or not (no RTS). (B) Number of interactions of yeast TFs RTS or no RTS. Blue and green boxes correspond to ordered proteins and IDPs, respectively. (TIF) [file pone.0265422.s009.tif]

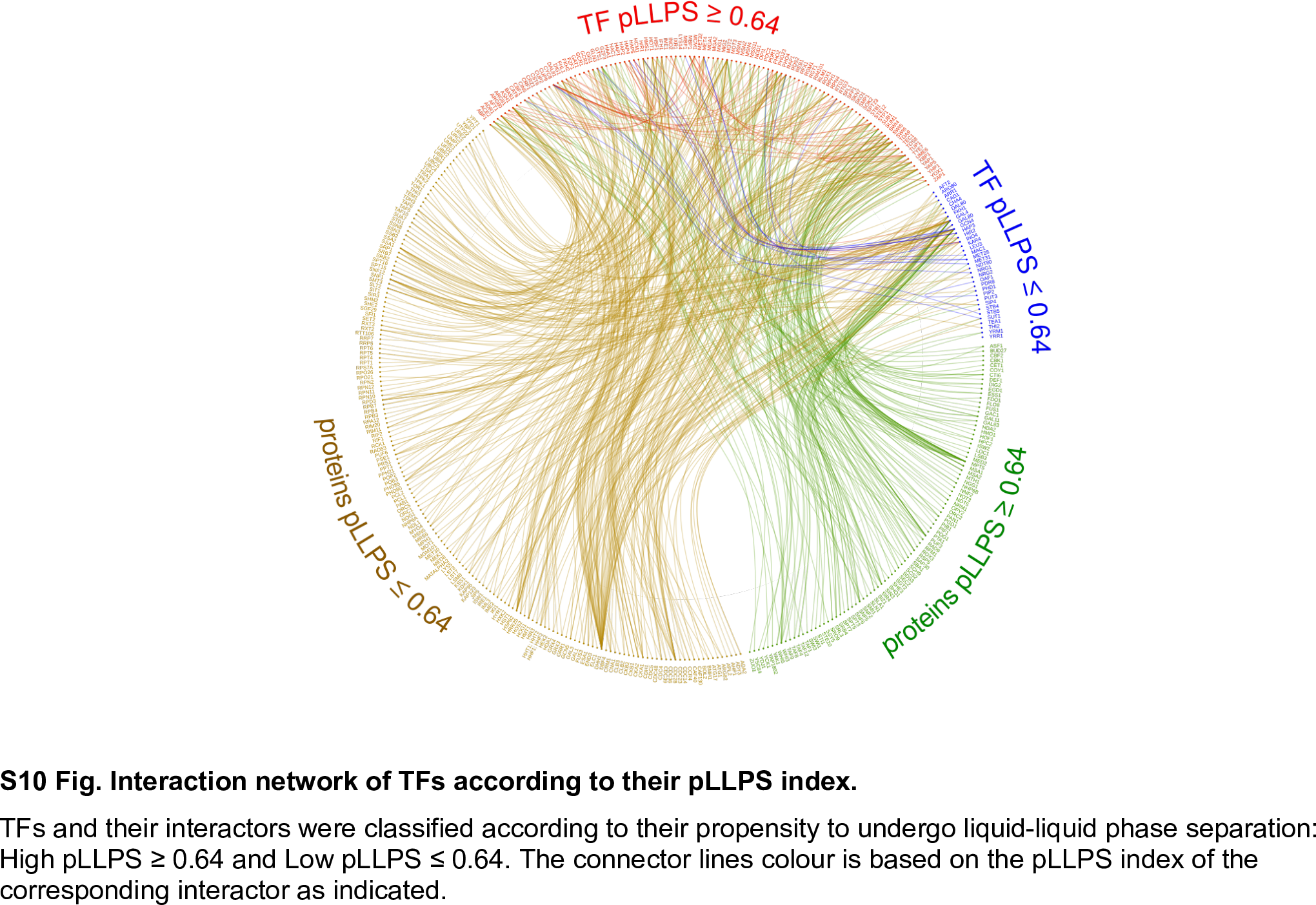

Supplement: S10 Fig — TFs and their interactors were classified according to their propensity to undergo liquid-liquid phase separation: High pLLPS ≥ 0.64 and Low pLLPS ≤ 0.64. The connector lines colour is based on the pLLPS index of the corresponding interactor as indicated. (TIF) [file pone.0265422.s010.tif]

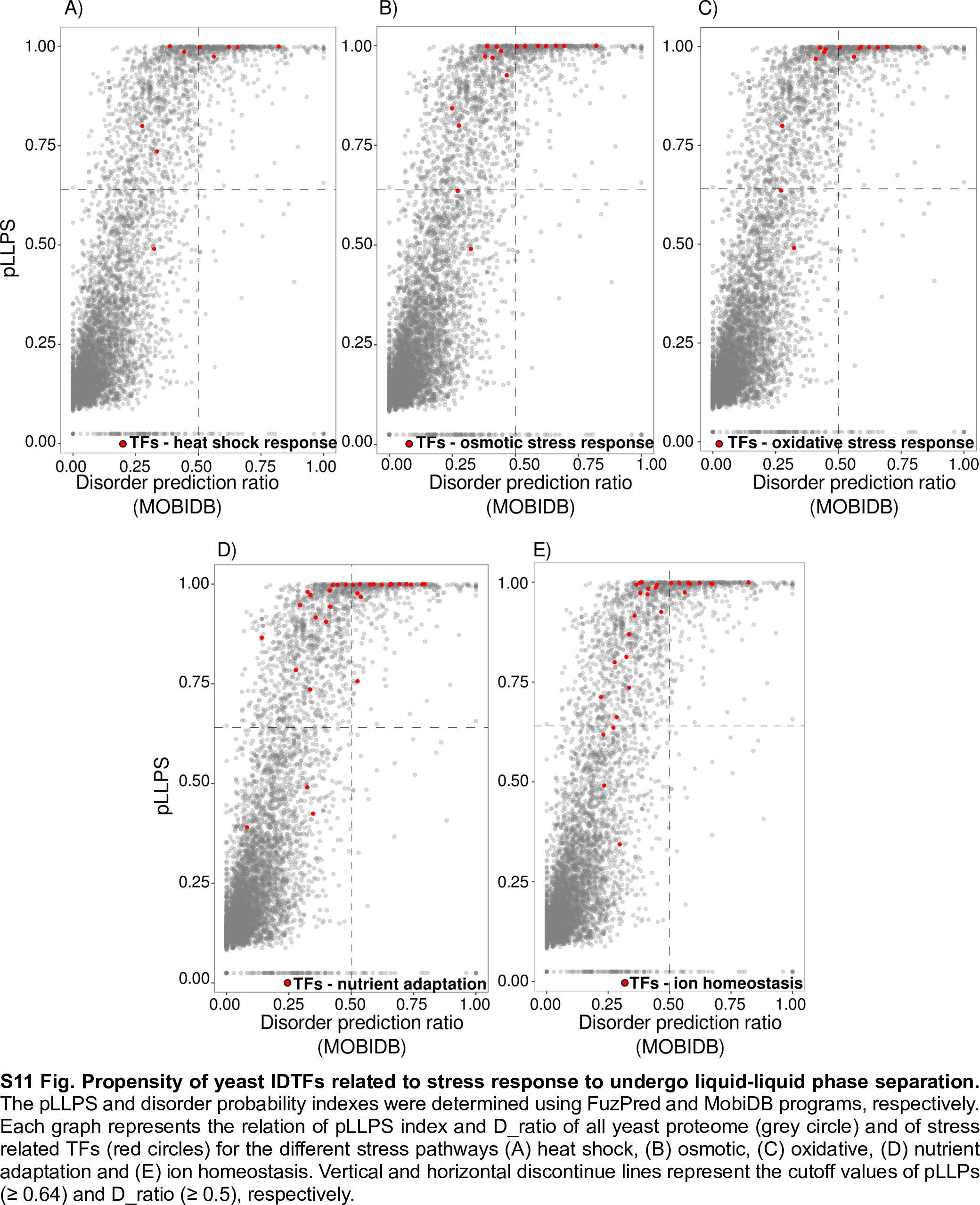

Supplement: S11 Fig — The pLLPS and disorder probability indexes were determined using FuzPred and MobiDB programs, respectively. Each graph represents the relation of pLLPS index and D_ratio of all yeast proteome (grey circle) and of stress related TFs (red circles) for the different stress pathways: (A) heat shock, (B) osmotic, (C) oxidative, (D) nutrient adaptation and (E) ion homeostasis. Vertical and horizontal discontinue lines represent the cutoff values of pLLPs (≥ 0.64) and D_ratio (≥ 0.5), respectively. (TIF) [file pone.0265422.s011.tif]
