# Supplementary material for: Intrinsically disordered signaling proteins: Essential hub players in the control of stress responses in Saccharomyces cerevisiae
Source: PLoS One. 2022 Mar 15;17(3):e0265422. doi: 10.1371/journal.pone.0265422 (PMC8923507; doi:10.1371/journal.pone.0265422)
Supplement: S1 Table — (PDF) [file pone.0265422.s012.pdf]

S1 Table: Raw data from structural disorder anayles of *S. cerevisiae* proteome.

UNIPROT ID	Disorder Ratio VSL2	#IDRs VSL2	Disorder Ratio IUPred2	#IDR IUPred	Disorder Ratio MOBI-Lite	#IDR MOBI-Lite	Disorder Ratio MobiDB	#IDR MobiDB	TF	pLLPS FuzPred
P25371	0.2374	3	0.0467	0	0	0	0.0648	0	NA	0.345
P38242	0.0802	0	0	0	0	0	0	0	NA	0.0949
P53909	0.1297	0	0	0	0	0	0.0173	0	NA	0.1373
P47143	0.1706	0	0	0	0	0	0.0441	0	NA	0.134
P43548	0.1577	1	0.0018	0	0	0	0.0323	0	NA	0.1225
Q03233	0.5505	1	0.1515	0	0	0	0.2273	0	NA	0.23
Q12157	0.3273	0	0.1152	0	0	0	0.1576	0	NA	0.3705
Q12028	0.274	1	0.0288	0	0	0	0.0397	0	NA	0.2296
P26449	0.0704	0	0	0	0	0	0.0147	0	NA	0.1175
P00854	0.0309	0	0	0	0	0	0	0	NA	0.1348
P07252	0.1177	0	0.0031	0	0	0	0.0092	0	NA	0.1602
P06101	0.7115	4	0.2055	1	0.1542	1	0.1779	1	NA	0.39
P06704	0.323	0	0.0683	0	0	0	0.118	0	NA	0.1789
P27636	0.2546	3	0.04	0	0.0821	2	0.1099	2	NA	0.3836
P43618	0.7175	3	0.6787	2	0.4737	2	0.4737	2	NA	0.983
P22137	0.0901	1	0.0139	0	0	0	0.0224	0	NA	0.1544
P47001	0.5198	1	0.2247	1	0.2247	1	0.326	1	NA	0.7667
Q2V2P9	0.1667	0	0	0	0	0	0.1515	0	NA	0.1374
P32323	0.8786	1	0.3131	1	0	0	0.4317	2	NA	0.9999
P40024	0.3492	2	0.0787	0	0.0705	1	0.0803	1	NA	0.1707
Q04413	0.8257	2	0.2661	0	0.4862	1	0.5505	1	NA	0.8125
P53983	0.2589	1	0.0148	0	0	0	0.0414	0	NA	0.1878
Q12675	0.3586	4	0.1998	2	0.1706	3	0.183	3	NA	0.6019
Q12406	0.3166	2	0.1279	1	0	0	0.109	1	NA	0.3901
P31386	0.2012	0	0.0631	0	0	0	0.0961	0	NA	0.2606
P03965	0.1252	0	0.0134	0	0	0	0.017	0	NA	0.1634
Q06702	0.0399	0	0	0	0	0	0.0166	0	NA	0.1247
P25651	0.5053	2	0.0526	0	0	0	0.1105	0	NA	0.33
P38930	0.2713	1	0.0775	0	0.1357	0	0.1473	0	NA	0.2533
Q12348	0.2868	2	0.1054	1	0.1054	1	0.1116	1	NA	0.1638
P53822	0.126	0	0	0	0	0	0.0604	0	NA	0.1209
P53943	0.1962	1	0.1263	0	0.1621	2	0.1621	2	NA	0.1788
Q99325	0.2891	1	0.1094	0	0.2344	1	0.2266	1	NA	0.2417
P38840	0.1209	0	0.0136	0	0	0	0.0429	0	NA	0.2605
P07834	0.3504	2	0.2195	2	0	0	0.163	2	NA	0.3668
P19073	0.1361	0	0	0	0	0	0	0	NA	0.1312
P38147	0.1423	1	0.0076	0	0	0	0.0304	0	NA	0.2104
P22516	0.2985	1	0.0906	0	0.0987	1	0.1161	1	NA	0.3151
Q00684	0.3938	1	0.3303	1	0.3249	2	0.3285	2	NA	0.4786
Q06156	0.3104	4	0.0468	0	0	0	0.0595	0	NA	0.2733
Q04368	0.1153	0	0.0068	0	0	0	0	0	NA	0.1568
P33307	0.1104	0	0.001	0	0	0	0.0073	0	NA	0.1188
P32623	0.3983	1	0.2934	1	0.2741	1	0.2677	1	NA	0.7377
P43497	0.7935	1	0.413	1	0	0	0.4022	1	NA	0.1818
P40351	0.1005	1	0.0121	0	0.0555	1	0.0624	1	NA	0.1144
Q02336	0.3894	2	0.1406	0	0	0	0.1406	1	NA	0.336
P38116	0.2896	0	0	0	0	0	0.0656	0	NA	0.1715
P0CZ17	0.1713	0	0.0193	0	0	0	0.0387	0	NA	0.3916

Q06596	0.3776	1	0.119	1	0.1224	1	0.1088	1	TRU	0.1694
P40344	0.3613	1	0.1968	0	0	0	0.1419	0	NA	0.2578
P14180	0.3873	2	0.2399	3	0.217	2	0.2222	2	NA	0.4598
P40452	0.3046	1	0.0355	0	0	0	0.0812	0	NA	0.1384
P19516	0.21	1	0.05	0	0	0	0.07	0	NA	0.1368
P49573	0.633	2	0.2906	2	0.3941	2	0.4113	3	NA	0.9991
Q06159	0.5226	1	0.1583	0	0.1633	1	0.2211	1	NA	0.3553
P0CX77	0.1713	0	0.0193	0	0	0	0.0387	0	NA	0.3916
P80428	0.2515	1	0.1513	1	0.1534	1	0.1779	1	NA	0.3076
P40518	0.3636	1	0.0519	0	0	0	0.0909	0	NA	0.189
P40580	0.1673	0	0.038	0	0	0	0.0494	0	NA	0.1872
P47113	0.9024	3	0.5941	1	0	0	0.3972	1	NA	0.9203
P53829	0.2922	1	0.2761	1	0.2708	1	0.2708	1	NA	0.2324
P53894	0.5622	4	0.4431	1	0.414	1	0.4286	2	NA	0.9423
P09119	0.2865	1	0.1248	1	0	0	0.1345	0	NA	0.2376
P32797	0.3442	3	0.1061	0	0.1353	1	0.1742	1	NA	0.5016
Q03705	0.1381	0	0	0	0	0	0	0	NA	0.1139
Q06675	0.4103	1	0.1875	1	0	0	0.1223	0	NA	0.2974
P32657	0.5184	4	0.3495	3	0.3045	4	0.3086	4	NA	0.8981
P11433	0.5082	5	0.2108	1	0.2237	2	0.2541	2	NA	0.9909
Q07897	0.512	1	0.2474	1	0.2749	1	0.3162	1	NA	0.4071
Q08955	0.0769	0	0	0	0	0	0	0	NA	0.1279
P53301	0.6765	2	0.2485	1	0.2623	1	0.3964	1	NA	0.988
P13711	0.1858	2	0.0334	0	0	0	0.0254	0	NA	0.1778
Q02895	0.231	1	0.0205	0	0	0	0.0322	0	NA	0.1073
P39010	0.2094	2	0.1113	2	0.1178	2	0.1257	2	NA	0.3531
P36117	0.6273	4	0.3257	0	0.2437	1	0.2612	1	NA	0.9124
Q12449	0.4086	1	0.1657	1	0.18	1	0.1914	1	NA	0.6113
P40851	0.2268	1	0.0132	0	0	0	0.0373	0	NA	0.1759
P47176	0.1489	0	0.0266	0	0	0	0.0745	0	NA	0.1587
Q12140	0.6067	1	0.1037	0	0	0	0.5366	2	NA	0.9944
P14905	0.1748	0	0.0206	0	0	0	0.0488	0	NA	0.2723
Q12018	0.2405	2	0.0344	0	0	0	0.0577	0	NA	0.3476
P38221	0.1641	1	0.046	0	0	0	0.0985	0	NA	0.1202
P10614	0.1509	0	0.0113	0	0	0	0.0283	0	NA	0.1299
P36090	0.1977	1	0.0446	0	0	0	0.0252	0	NA	0.1566
P38207	0.2981	1	0.0808	0	0	0	0.1135	0	NA	0.2013
P38700	0.3884	3	0.2066	1	0.205	1	0.2066	1	NA	0.4583
Q12142	0.4654	4	0.338	3	0.2778	4	0.2598	4	NA	0.5342
P47117	0.2695	1	0.0379	0	0	0	0.0535	0	NA	0.1969
P32504	0.4027	4	0.1715	2	0.1653	2	0.1715	2	NA	0.8388
P33322	0.383	1	0.2464	1	0.2774	1	0.3147	1	NA	0.5481
P53836	0.6962	2	0.5387	3	0.5245	3	0.5123	5	NA	0.9883
P32582	0.2426	2	0.0592	0	0	0	0.0513	0	NA	0.1462
P32562	0.3518	2	0.0993	1	0.0908	1	0.0894	0	NA	0.1999
Q08032	0.26	2	0.14	1	0.1492	1	0.1738	1	NA	0.2958
Q12262	0.5028	1	0.116	0	0	0	0.1713	0	NA	0.3712
P40202	0.1647	0	0.004	0	0	0	0.0763	0	NA	0.189
Q12734	0.6173	7	0.4291	7	0.3452	4	0.3586	3	NA	0.9984
P38287	0.2287	1	0.0452	0	0	0	0.0984	0	NA	0.2793
P19454	0.1062	0	0.0118	0	0	0	0.0383	0	NA	0.1055
P08679	0.2804	0	0.0239	0	0	0	0.0457	0	NA	0.3948

P04039	0.2564	0	0	0	0	0	0.0897	0	NA	0.1057
P47081	0.3559	0	0	0	0	0	0.1525	0	NA	0.1291
P00401	0.0581	0	0	0	0	0	0	0	NA	0.1226
P37898	0.1016	0	0.0058	0	0	0	0.0152	0	NA	0.1078
P10127	0.0838	0	0	0	0	0	0	0	NA	0.1781
P43633	0.6171	1	0.3342	3	0.3184	3	0.3974	5	NA	0.9843
P38929	0.3666	4	0.0665	0	0.0529	0	0.0691	0	NA	0.2754
Q12386	0.4381	5	0.2452	1	0.1952	1	0.2043	1	NA	0.3612
P06182	0.5948	2	0.4238	1	0.3978	1	0.4052	1	NA	0.8988
P00546	0.0772	0	0	0	0	0	0.0336	0	NA	0.1424
P43621	0.5201	2	0.2711	2	0.1868	1	0.207	1	NA	0.4741
P40512	0.1824	1	0.0428	0	0	0	0.0833	1	NA	0.1089
P53195	0.6129	2	0.1254	0	0	0	0.1613	0	NA	0.535
P00330	0.1121	0	0	0	0	0	0.0316	0	NA	0.1379
Q08548	0.2924	1	0.0549	0	0	0	0.0856	0	NA	0.4096
P39721	0.1341	0	0	0	0	0	0.0325	0	NA	0.1362
P25649	0.0938	0	0	0	0	0	0.0703	0	NA	0.1013
P34233	0.8656	2	0.5119	4	0.4575	2	0.4796	1	TRU	0.9972
P40467	0.5363	3	0.3558	1	0.2822	3	0.2666	3	NA	0.9035
P32447	0.5591	1	0.5305	1	0.4839	1	0.4946	1	NA	0.8414
P38772	0.5246	3	0.3169	1	0.2568	1	0.3142	1	NA	0.7143
P43573	0.8304	5	0.6394	5	0.5465	5	0.495	5	NA	0.9763
P14772	0.2245	2	0.0481	0	0.043	0	0.0513	0	NA	0.2361
Q12128	0.5134	2	0.3692	1	0.3105	2	0.2641	2	NA	0.8442
P50077	0.2408	2	0.1408	3	0.1442	2	0.1462	2	NA	0.8032
Q12510	0.3831	1	0.1992	1	0.1762	1	0.1686	1	NA	0.2404
P08019	0.0984	0	0.0091	0	0	0	0.0146	0	NA	0.1455
Q06598	0.0866	0	0.0223	0	0	0	0.0396	0	NA	0.1251
Q12349	0.6532	1	0.6129	1	0.5968	1	0.5161	1	NA	0.7955
P41697	0.7043	6	0.3858	1	0.3084	2	0.3135	2	NA	0.8998
Q08492	1	1	0.8972	1	0.8692	1	0.8458	2	NA	0.9567
P53630	0.2068	0	0.0802	0	0	0	0.1139	0	NA	0.23
P35203	0.2155	1	0.0858	1	0.1088	1	0.1151	1	NA	0.1885
Q06350	0.1937	0	0.0333	0	0	0	0.0548	0	NA	0.2585
P47167	0.4184	1	0.0418	0	0	0	0.0502	0	NA	0.1466
P29029	0.4555	1	0.1352	0	0.1352	0	0.2046	1	NA	0.9669
P08004	0.3643	3	0.2927	2	0.2237	3	0.2175	2	NA	0.5771
P20437	0.4597	3	0.2619	1	0.2308	1	0.2289	1	NA	0.8414
P17898	0.1069	0	0	0	0	0	0	0	NA	0.117
P32799	0.5349	1	0.0543	0	0	0	0.1628	0	NA	0.3015
P32074	0.2332	1	0.046	0	0	0	0.0727	1	NA	0.1827
P40094	0.2971	2	0.0587	0	0	0	0.0961	0	NA	0.2131
Q12218	0.7762	1	0.2115	0	0	0	0.4641	1	NA	1
P53220	0.364	1	0.1046	0	0	0	0.1506	0	NA	0.3372
P53299	0.4857	1	0.1429	0	0.3429	0	0.3429	0	NA	0.3724
Q12513	0.7667	2	0.3867	1	0.2867	1	0.32	1	NA	0.5094
P38238	0.3581	2	0.0806	0	0.1548	1	0.1806	1	NA	0.2372
P48561	0.4782	3	0.3364	3	0.3178	3	0.3209	3	NA	0.3739
Q08282	0.141	1	0.0173	0	0	0	0.0201	0	NA	0.133
P38334	0.2457	0	0.1029	0	0	0	0.1371	0	NA	0.4365
P26370	0.2973	1	0.0265	0	0	0	0.0549	0	TRU	0.1323
P38627	0.1401	0	0.0277	0	0	0	0.0173	0	NA	0.2036

Q03913	0.1579	0	0	0	0	0	0.0105	0	NA	0.1015
Q06685	0.4293	6	0.2531	3	0.212	4	0.2216	3	NA	0.8557
P38173	0.3091	0	0	0	0	0	0.2455	0	NA	0.1636
P25384	0.4475	4	0.3475	6	0.2944	5	0.2859	6	NA	0.9035
Q8TGQ2	0.5517	1	0	0	0	0	0.0603	0	NA	0.2681
P38233	0.3534	0	0.0086	0	0	0	0.1724	0	NA	0.5363
A0A023PXB	0.3554	0	0	0	0	0	0	0	NA	0.11
Q8TGK6	0.04	0	0	0	0	0	0.04	0	NA	0.0951
P25575	0.6916	1	0.028	0	0	0	0.2056	0	NA	0.2601
A0A023PXH	0.1931	0	0.0069	0	0	0	0.0138	0	NA	0.125
Q12448	0.114	0	0	0	0	0	0	0	NA	0.1041
P25606	0.1772	0	0	0	0	0	0.019	0	NA	0.1689
Q12115	0.69	1	0	0	0	0	0.11	0	NA	0.1719
P37265	0.0273	0	0	0	0	0	0	0	NA	0.1081
Q12210	0.0654	0	0	0	0	0	0	0	NA	0.0893
Q12023	0.1215	0	0	0	0	0	0	0	NA	0.1331
Q12301	0.3601	1	0.0548	0	0.0585	0	0.0695	1	NA	0.4374
P39566	0.0404	0	0	0	0	0	0	0	NA	0.0847
P89495	0.7297	1	0	0	0	0	0.0811	0	NA	0.2879
A0A023PYD	0.2616	0	0	0	0	0	0.1105	0	NA	0.129
A0A023PYD	0.096	0	0	0	0	0	0	0	NA	0.126
A0A023PZC	0.0426	0	0	0	0	0	0	0	NA	0.1095
A0A023PZA	0.2435	0	0.0522	0	0	0	0.1391	0	NA	0.4376
P87288	0.287	0	0	0	0	0	0	0	NA	0.1428
A0A023PZH	0.1102	0	0	0	0	0	0	0	NA	0.1281
Q8TGU7	0.5294	0	0.3382	0	0	0	0.4706	0	NA	0.7018
P87271	0.6275	1	0.2157	0	0.451	1	0.451	1	NA	0.9358
Q8TGK4	0.5068	0	0	0	0	0	0.1096	0	NA	0.1513
A0A023PXC	0.2087	0	0	0	0	0	0.087	0	NA	0.1949
P32616	0.2979	1	0	0	0	0	0	0	NA	0.1278
P40004	0.0556	0	0.0058	0	0	0	0	0	NA	0.1125
P87273	0.0901	0	0	0	0	0	0	0	NA	0.0998
Q3E834	0.2	0	0.0118	0	0	0	0.0471	0	NA	0.4066
P34251	0.2524	1	0.0356	0	0	0	0.0744	0	NA	0.4673
P33324	0.1516	0	0.0323	0	0	0	0.0613	0	NA	0.1948
P46994	0.1639	0	0	0	0	0	0	0	NA	0.1222
Q06695	0.4545	1	0.1288	0	0	0	0.2955	0	NA	0.654
Q02659	0.427	1	0.1189	0	0.1676	0	0.2108	0	NA	0.3172
P36065	0.7273	1	0	0	0	0	0.1061	0	NA	0.6076
P53132	0.1172	0	0	0	0	0	0	0	NA	0.1006
P53144	0.1349	0	0.0372	0	0	0	0.0791	0	NA	0.1822
P0CL38	0.2125	0	0	0	0	0	0	0	NA	0.1411
A0A023PZK	0.2698	0	0	0	0	0	0.0159	0	NA	0.2322
P0CX60	0.6432	2	0.5841	3	0.5386	3	0.5432	3	NA	0.9949
Q12273	0.4519	4	0.335	4	0.2997	4	0.3105	4	NA	0.8953
Q8TGJ2	0.4333	0	0	0	0	0	0.2667	0	NA	0.0248
P0C5R8	0.1311	0	0	0	0	0	0	0	NA	0.1607
P53105	0.3478	1	0.1217	0	0	0	0.2522	0	NA	0.1356
Q02831	0.6833	2	0.6083	2	0.3208	1	0.3542	2	NA	0.6384
P40326	0.2593	0	0	0	0	0	0.0463	0	NA	0.1269
Q8TGN0	0.3421	0	0	0	0	0	0.5	0	NA	0.0248
Q12322	0.4802	1	0.0891	0	0	0	0.1881	0	NA	0.491

Q3E7A5	0.05	0	0	0	0	0	0	0	NA	0.0997
Q3E809	0.1346	0	0	0	0	0	0	0	NA	0.1032
Q08993	0.2143	0	0	0	0	0	0.021	0	NA	0.1257
P35725	0.8144	1	0.6826	1	0.7665	1	0.7784	1	NA	0.997
Q3E7A0	0.359	0	0	0	0	0	0.1538	0	NA	0.0248
P0C2J0	0.4618	4	0.3371	4	0.3035	4	0.3041	4	NA	0.888
Q6B108	0.19	0	0.03	0	0	0	0.09	0	NA	0.1208
Q08954	0.5542	2	0.4333	1	0.4458	2	0.4083	1	NA	0.9572
Q07989	0.4245	1	0.0943	0	0	0	0	0	NA	0.1472
P36127	0.0288	0	0	0	0	0	0	0	NA	0.0863
Q08995	0.3905	1	0.2132	1	0.2219	1	0.2568	1	NA	0.9201
Q3E805	0.2157	0	0	0	0	0	0.1569	0	NA	0.1313
P53837	0.5115	0	0	0	0	0	0.0305	0	NA	0.1746
Q08756	0.2353	0	0.0294	0	0	0	0.1765	0	NA	0.1137
P40218	0.6413	1	0.3408	1	0	0	0.278	0	NA	0.8679
Q04336	0.2895	2	0.1461	2	0.1333	2	0.1305	2	NA	0.7995
Q08736	0.3529	0	0	0	0	0	0.1765	0	NA	0.287
Q08909	0.2966	0	0.0828	0	0	0	0.1483	0	NA	0.2542
Q06252	0.3184	0	0.0249	0	0	0	0.0597	0	NA	0.3317
P0CX72	0.6432	2	0.5773	3	0.5523	3	0.5591	3	NA	0.9958
P32795	0.3307	3	0.1486	2	0.1486	2	0.1526	2	NA	0.5796
O13561	0.2051	0	0	0	0	0	0.0342	0	NA	0.157
P0CF20	0.0947	0	0	0	0	0	0.0118	0	NA	0.1169
Q12225	0.3028	0	0	0	0	0	0	0	NA	0.2205
P32939	0.2596	0	0.0144	0	0	0	0.0577	0	NA	0.1262
A0A023PYG	0.213	0	0	0	0	0	0	0	NA	0.1273
P40538	0.0704	0	0	0	0	0	0.0141	0	NA	0.0966
P38815	0.3551	1	0.2617	1	0.2991	1	0.3271	1	NA	0.296
P38729	0.1333	0	0	0	0	0	0	0	NA	0.1203
P53906	0.28	0	0.03	0	0	0	0.16	0	NA	0.5788
Q2V2P5	0.0933	0	0	0	0	0	0	0	NA	0.1497
P40083	0.6149	1	0.1014	0	0	0	0.25	0	NA	0.3263
A0A023PZK	0.0709	0	0	0	0	0	0.0315	0	NA	0.1159
P47106	0.3583	0	0.0917	0	0	0	0.2833	0	NA	0.1891
P36025	0.3731	1	0.1157	0	0	0	0.1269	0	NA	0.2374
P40152	0.138	0	0.0092	0	0	0	0.0307	0	NA	0.1179
P47151	0.3769	1	0	0	0	0	0.1308	0	NA	0.2971
P47159	0.1496	1	0.0379	0	0	0	0.0558	0	NA	0.1495
Q08439	0.0903	0	0	0	0	0	0	0	NA	0.1349
Q8TGT3	0.125	0	0	0	0	0	0.1875	0	NA	0.0248
P53240	0.0984	0	0	0	0	0	0	0	NA	0.0994
P53245	0.0541	0	0	0	0	0	0	0	NA	0.0926
P47105	0.3937	1	0.1732	0	0.2283	0	0.2205	0	NA	0.2382
P36160	0.5378	2	0.2703	1	0.2616	1	0.2587	1	NA	0.2964
Q03465	0.6742	2	0.5311	4	0.3427	1	0.3352	2	TRU	0.7356
Q08723	0.2544	1	0.142	1	0.1331	1	0.1361	1	NA	0.1775
P53256	0.13	0	0	0	0	0	0.0269	0	NA	0.1113
P38792	0.39	2	0.1755	0	0	0	0.2117	0	NA	0.4056
P0CX34	1	1	0.873	1	0.7937	1	0.7937	1	NA	0.9937
Q12754	0.2761	2	0.1458	2	0.1213	1	0.1173	1	NA	0.3062
P32573	0.1473	0	0.0034	0	0	0	0.0445	0	NA	0.1214
P36048	0.2569	3	0.1081	1	0.1052	1	0.0992	1	NA	0.226

P39928	0.4492	3	0.2844	3	0.223	3	0.2336	3	NA	0.9223
P39543	0.1197	0	0.0128	0	0	0	0.0684	0	NA	0.1243
P06783	0.234	2	0.066	0	0.0979	1	0.1213	1	NA	0.1712
P38992	0.212	1	0.0115	0	0	0	0.0544	0	NA	0.1612
P06784	0.4447	3	0.1786	1	0	0	0.1398	0	NA	0.3801
P39073	0.364	3	0.2559	1	0.209	1	0.1964	1	NA	0.2393
P29055	0.3101	1	0.1536	0	0	0	0.142	1	NA	0.1947
P09734	0.173	0	0.0202	0	0	0	0.0382	0	NA	0.2347
P32802	0.1424	0	0.048	0	0	0	0.0435	0	NA	0.1269
P46959	0.3368	1	0.0862	0	0	0	0.0992	0	NA	0.134
Q12256	0.3551	2	0.2701	2	0.2656	2	0.2686	2	NA	0.6233
Q02159	0.2545	0	0.0485	0	0	0	0.0667	0	NA	0.2113
P20795	0.1722	0	0.0043	0	0	0	0.0145	0	NA	0.1358
P39904	0.2964	1	0.0671	0	0	0	0.0983	0	NA	0.1657
Q12045	0.6445	2	0.1592	1	0	0	0.1097	1	NA	0.2786
Q04301	0.0943	0	0.0285	0	0	0	0.0445	0	NA	0.1091
P53241	0.2007	1	0.0287	0	0	0	0.0523	0	NA	0.1173
P40463	0.9083	2	0.8968	5	0.7615	3	0.7133	5	NA	0.9979
Q12241	0.7314	3	0.3922	1	0.3216	2	0.3286	1	NA	0.3914
P40498	0.4244	2	0.2469	2	0.2427	1	0.2663	1	NA	0.4054
P53962	0.2185	1	0.0617	0	0.1054	1	0.1157	1	NA	0.2563
P47188	0.2328	0	0	0	0	0	0.0603	0	NA	0.1807
P0C5N6	0.322	0	0	0	0	0	0.3559	0	NA	0.5594
P40028	0.5007	2	0.2503	1	0.2108	0	0.2227	0	NA	0.5679
P47157	0.3534	1	0.1466	0	0	0	0.3276	1	NA	0.2327
P53213	0.3853	1	0.1743	0	0	0	0.1743	0	NA	0.1473
P53960	0.3158	1	0.0329	0	0	0	0.0329	0	NA	0.1445
P47174	0.3932	0	0.0085	0	0	0	0	0	NA	0.151
Q12085	0.6432	2	0.5795	3	0.5727	3	0.5795	3	NA	0.996
P53272	0.3184	2	0	0	0	0	0.0423	0	NA	0.1451
P32475	0.4261	1	0.0087	0	0	0	0.0174	0	NA	0.1384
P53302	0.3471	0	0	0	0	0	0.1488	0	NA	0.1327
P32804	0.1516	1	0.0505	0	0	0	0.0824	0	NA	0.1862
P0CX20	0.2906	3	0.1153	1	0.1281	1	0.147	1	NA	0.8955
P54786	0.8461	3	0.6635	8	0.5212	6	0.5021	7	NA	0.9842
P50875	0.7003	5	0.6523	5	0.5596	5	0.5033	3	NA	0.9944
Q03770	0.2547	3	0.0951	0	0.0692	0	0.0786	0	NA	0.4576
P16965	1	1	1	1	1	1	1	1	NA	0.971
P32917	0.5158	3	0.2846	4	0.2399	4	0.2323	2	NA	0.6633
P15625	0.1928	1	0.002	0	0	0	0.0159	0	NA	0.1338
P12612	0.195	0	0.0215	0	0	0	0.0429	0	NA	0.1479
Q12328	0.3043	0	0.1546	0	0	0	0.1594	0	NA	0.2216
P34111	0.3026	4	0.0966	1	0.0655	1	0.0966	1	NA	0.3209
Q06177	1	1	0.9767	2	0.907	1	0.8605	0	NA	0.6032
P53283	0.3274	1	0.2313	2	0.2052	2	0.1824	2	NA	0.3383
P23644	0.2041	1	0.0155	0	0	0	0.0517	0	NA	0.1675
P38426	0.3482	2	0.2201	3	0.2087	4	0.1907	3	NA	0.7215
Q08960	0.3642	3	0.0938	0	0.121	2	0.2333	3	NA	0.8155
P38254	0.1873	2	0.0292	0	0	0	0.0544	0	NA	0.159
P38067	0.1751	1	0	0	0	0	0.0342	0	NA	0.1271
Q12220	0.3086	3	0.0509	0	0	0	0.0604	0	NA	0.2674
Q92331	0.6978	5	0.3807	3	0.3037	1	0.3081	2	NA	0.6273

P22203	0.5622	1	0.0644	0	0	0	0.073	0	NA	0.2471
Q04602	0.3516	1	0.2591	1	0.2604	1	0.2734	2	NA	0.2896
P23642	0.2579	1	0.0654	0	0.0935	0	0.1178	1	NA	0.3651
P38239	0.5833	1	0.2278	1	0.3	1	0.2889	1	NA	0.2873
P38081	0.2495	1	0.0299	0	0	0	0.0619	0	NA	0.1483
P32788	0.6143	2	0.1893	0	0.1929	0	0.1964	0	NA	0.3688
P38178	0.305	0	0	0	0	0	0.0567	0	NA	0.111
A0A023PXA	0.0899	0	0	0	0	0	0.0159	0	NA	0.1505
Q8TGR8	0.2963	1	0.0185	0	0	0	0.0864	0	NA	0.7324
O13511	0.75	1	0.3828	0	0.5938	1	0.5547	0	NA	0.9886
P25608	0.1114	0	0.0163	0	0	0	0.0054	0	NA	0.1357
P25349	0.4332	1	0.1943	1	0.2267	1	0.247	1	NA	0.8673
Q07438	0.1935	0	0	0	0	0	0	0	NA	0.1226
P39989	0.1699	0	0.0131	0	0	0	0	0	NA	0.1154
P25629	0.5541	1	0.0878	0	0	0	0.3446	0	NA	0.8302
Q12257	0.4471	1	0.2059	0	0.2412	0	0.2176	0	NA	0.2208
Q03494	0.4458	4	0.3452	6	0.2859	6	0.2802	7	NA	0.8935
P40102	0.3617	0	0.2199	0	0	0	0.1986	0	NA	0.3936
P0CX16	0.3472	1	0.125	0	0	0	0.1667	0	NA	0.2764
A0A023PYD	0.0811	0	0	0	0	0	0.0811	0	NA	0.2325
Q8TGQ5	0.2759	0	0	0	0	0	0.3103	0	NA	0.0248
Q8TGP2	0.6	0	0.0714	0	0	0	0.4	0	NA	0.4058
Q8TGR3	0.0986	0	0	0	0	0	0.0423	0	NA	0.1699
P0C0V2	0.2525	0	0	0	0	0	0.2323	0	NA	0.1185
A0A023PYE	0.1639	0	0	0	0	0	0.0656	0	NA	0.1677
Q8TGK5	0.2062	0	0	0	0	0	0	0	NA	0.1445
P0C5M4	0.1467	0	0	0	0	0	0.04	0	NA	0.117
A0A023PZB	0.0804	0	0	0	0	0	0	0	NA	0.1212
P38354	0.6667	1	0.2361	0	0	0	0.25	0	NA	0.2391
A0A023PZE	0.1353	0	0	0	0	0	0	0	NA	0.12
Q02590	0.8082	1	0.0274	0	0	0	0.2877	0	NA	0.6597
P40097	0.0561	0	0	0	0	0	0	0	NA	0.1174
P39999	0.099	0	0	0	0	0	0	0	NA	0.1104
P34249	0.2574	0	0	0	0	0	0.0693	0	NA	0.1468
O13556	0.2079	0	0	0	0	0	0.0446	0	NA	0.132
P0CY00	0.2125	0	0	0	0	0	0	0	NA	0.1441
Q02754	0.3788	1	0.101	0	0	0	0.1111	0	NA	0.2956
P39532	0.2435	0	0	0	0	0	0.2	0	NA	0.2208
P53828	0.4808	0	0	0	0	0	0.2019	0	NA	0.382
Q8TGK8	0.1915	0	0	0	0	0	0.0319	0	NA	0.1574
Q12139	0.5252	4	0.3972	4	0.3786	4	0.3654	6	NA	0.8681
Q03083	0.4848	1	0.0485	0	0	0	0.1758	0	NA	0.455
Q08270	0.5833	1	0.3981	0	0.3981	1	0.3704	0	NA	0.3134
Q04223	0.1325	0	0	0	0	0	0.0199	0	NA	0.1215
A0A023PYJ	0.1261	0	0	0	0	0	0	0	NA	0.2149
P0CX73	0.6432	2	0.5773	3	0.5523	3	0.5591	3	NA	0.9958
Q3E760	0.0104	0	0	0	0	0	0	0	NA	0.093
A0A023PZL	0.0323	0	0	0	0	0	0	0	NA	0.0855
P36158	0.4154	2	0.1162	0	0	0	0.1641	1	NA	0.3178
Q6B0X2	0.1038	0	0	0	0	0	0	0	NA	0.1646
P47038	0.4275	1	0	0	0	0	0	0	NA	0.225
Q12177	0.1409	0	0.0268	0	0	0	0.0302	0	NA	0.1894

Q04897	0.0495	0	0	0	0	0	0.0495	0	NA	0.0979
Q12523	0.4187	3	0.2314	2	0.218	2	0.2352	2	NA	0.8066
Q8TGL4	0.2558	0	0.1047	0	0	0	0	0	NA	0.099
O13569	0.7353	2	0	0	0	0	0.1706	0	NA	0.7965
A0A023PZH	0.5425	1	0	0	0	0	0.0654	0	NA	0.463
P36019	0.3136	1	0.2045	1	0	0	0.2091	1	NA	0.1772
P38740	0.2032	1	0.0952	0	0.1143	0	0.1556	0	NA	0.177
P38763	0.1562	0	0	0	0	0	0	0	NA	0.2159
A0A023PXM	0.1964	0	0	0	0	0	0	0	NA	0.1303
P40483	0.2083	1	0.056	0	0	0	0.0647	0	NA	0.4683
A0A023PXM	0.1728	0	0	0	0	0	0.037	0	NA	0.1025
P40572	0.8075	1	0.5094	2	0.5358	2	0.5358	2	NA	0.9721
Q3E7Y9	0.2459	0	0	0	0	0	0.2295	0	NA	0.1819
O13537	0.3136	0	0	0	0	0	0	0	NA	0.2166
P39971	0.3194	1	0.1204	0	0	0	0.1435	0	NA	0.2284
Q12496	0.188	2	0.026	0	0	0	0.026	0	NA	0.1344
Q8TGN3	0.2857	0	0	0	0	0	0.2857	0	NA	0.0248
P53265	0.245	0	0.0075	0	0	0	0.0275	0	NA	0.1934
P53293	0.0691	0	0.0133	0	0	0	0	0	NA	0.1243
Q99176	0.5399	2	0.1033	0	0	0	0.169	0	NA	0.2184
P38858	0.2169	0	0.0321	0	0	0	0.0562	0	NA	0.1442
P38324	0.1447	0	0.0132	0	0	0	0.0395	0	NA	0.1203
P46950	0.309	2	0.128	0	0.2011	1	0.1865	1	NA	0.3416
Q06707	0.1894	0	0.0076	0	0	0	0.0909	0	NA	0.1482
P38960	0.2085	1	0.0324	0	0	0	0.0304	0	NA	0.1196
P54000	0.8836	2	0.839	2	0.8116	2	0.8116	2	TRU	0.9931
P41544	0.2512	1	0.0049	0	0	0	0.1773	0	NA	0.151
P47988	0.419	2	0.1739	1	0.1357	1	0.1673	1	TRU	0.3568
P53904	0.3465	0	0.1654	0	0.0866	0	0.1063	0	NA	0.1771
Q06510	0.3675	2	0.0709	0	0	0	0.0656	0	NA	0.1887
P39515	0.2278	0	0.0759	0	0	0	0.1456	0	NA	0.2275
P49957	0.3763	1	0.2401	0	0.319	1	0.3047	2	NA	0.6372
P20049	0.1394	0	0.0111	0	0	0	0.042	0	NA	0.1159
P29340	0.1093	0	0	0	0	0	0.0328	0	NA	0.1454
P40554	0.1717	0	0	0	0	0	0.0606	0	NA	0.1415
P39538	0.3453	3	0.2193	3	0.1874	3	0.1906	2	NA	0.5158
Q12132	0.9795	2	0.7826	4	0.6394	3	0.6547	3	TRU	0.9967
Q12208	0.2103	1	0.0586	0	0.0897	0	0.1034	0	NA	0.2332
Q06078	0.3152	2	0.0863	1	0	0	0.1076	0	NA	0.3227
Q05946	0.1322	0	0.0122	0	0	0	0.0073	0	NA	0.1286
P39967	0.5822	3	0.4005	2	0.366	3	0.3859	3	NA	0.8374
P48412	0.7364	2	0.5039	3	0.491	3	0.4806	3	NA	0.9436
Q03308	0.104	0	0.0013	0	0	0	0.0201	0	NA	0.1112
Q12206	0.394	2	0.212	1	0.1649	1	0.1777	1	NA	0.414
Q12416	0.9254	2	0.9729	2	0.9017	2	0.8983	2	NA	0.9979
P25361	0.3386	0	0	0	0	0	0.0787	0	NA	0.1769
Q07793	0.4732	4	0.366	4	0.3392	4	0.3392	4	NA	0.944
Q12394	0.4628	1	0.0083	0	0	0	0.2479	0	NA	0.5256
P0C5E0	0.0909	0	0	0	0	0	0	0	NA	0.3048
Q12472	0.4452	4	0.3429	6	0.2876	6	0.2757	6	NA	0.8962
Q3E789	1	1	0.5714	0	0	0	0.7143	0	NA	0.9515
Q05510	0.2245	0	0.0748	0	0	0	0.1701	0	NA	0.1325

Q8TGR5	0.2308	0	0.0192	0	0	0	0.1923	0	NA	0.1818
Q04033	0.2487	1	0.0107	0	0	0	0.0214	0	NA	0.2492
A0A023PXJ7	0.1887	0	0	0	0	0	0.0566	0	NA	0.1087
P38299	0.413	2	0.1128	1	0	0	0.109	1	NA	0.237
Q8TGU6	0.25	0	0.0469	0	0	0	0.1719	0	NA	0.1182
Q8TGR4	1	1	0.3958	0	0	0	0.8125	0	NA	0.9348
P87261	0.1443	0	0	0	0	0	0.0846	0	NA	0.1452
Q12266	0.6386	2	0.5909	3	0.5432	3	0.5273	3	NA	0.9935
P0C5L3	0.5957	0	0	0	0	0	0.2766	0	NA	0.0248
P39548	0.2778	1	0.1154	0	0	0	0.2521	1	NA	0.3284
O13519	0.4344	0	0	0	0	0	0	0	NA	0.2602
P36138	1	1	0.8962	1	0.765	1	0.7104	1	NA	0.9897
Q99395	0.7816	2	0.6117	2	0.6456	2	0.6214	2	NA	0.996
P35733	0.0726	0	0	0	0	0	0	0	NA	0.1198
Q3E7A6	0.0556	0	0	0	0	0	0	0	NA	0.0248
O13518	0.6691	1	0.0072	0	0	0	0.2302	0	NA	0.6774
P32329	0.3866	2	0.0703	0	0	0	0.1476	0	NA	0.8742
O13567	0.0588	0	0	0	0	0	0	0	NA	0.1188
Q08259	0.1863	0	0	0	0	0	0.1373	0	NA	0.1186
P0C2I8	0.6432	2	0.5773	3	0.5455	3	0.5545	3	NA	0.9962
A0A023PXG	0.6436	1	0	0	0	0	0	0	NA	0.1456
Q08486	0.1346	0	0	0	0	0	0	0	NA	0.0943
P0CL40	0.2125	0	0	0	0	0	0	0	NA	0.1411
Q04116	0.9603	2	0.6147	1	0	0	0.4363	1	TRU	0.9377
Q07738	0.2764	0	0.0244	0	0	0	0.0407	0	NA	0.2101
P0C5B8	0.2125	0	0	0	0	0	0	0	NA	0.1363
P53736	0.2031	0	0.0039	0	0	0	0.0703	0	NA	0.1649
P53967	0.1143	0	0.0095	0	0	0	0	0	NA	0.1596
A0A023PZE9	0.4878	1	0	0	0	0	0	0	NA	0.1633
P47098	0.4519	4	0.335	4	0.3043	4	0.3094	4	NA	0.885
P0C5N5	0.5588	0	0	0	0	0	0.2059	0	NA	0.0248
Q99345	0.5487	1	0.2035	0	0	0	0.3186	0	NA	0.635
P0CL25	0.04	0	0	0	0	0	0.0533	0	NA	0.106
Q3E7B9	0.1486	0	0	0	0	0	0	0	NA	0.098
Q08222	0.1415	0	0.0094	0	0	0	0	0	NA	0.1707
Q3E817	0.2222	0	0	0	0	0	0.2063	0	NA	0.1125
Q8TGT9	0.0566	0	0	0	0	0	0	0	NA	0.1445
P53273	0.2529	1	0.1207	0	0	0	0.0772	0	NA	0.4178
P47066	0.2255	0	0	0	0	0	0	0	NA	0.132
Q12149	0.4598	2	0.1883	1	0.1896	1	0.1855	1	NA	0.3404
P38781	0.3488	3	0.1416	2	0.1257	2	0.1438	2	NA	0.8636
Q02206	0.3632	3	0.184	1	0.168	2	0.1744	2	NA	0.4165
P40063	0.9471	1	0.8269	1	0.5385	1	0.5673	1	NA	0.9148
P17121	0.6774	4	0.6254	4	0.6223	4	0.6116	5	NA	0.9953
Q03940	0.1814	0	0.0562	0	0	0	0.0756	0	NA	0.2401
P03878	0.1439	1	0.0072	0	0	0	0.0108	0	NA	0.1273
P36024	0.7011	3	0.6441	2	0.5907	3	0.5783	4	NA	0.9915
P36068	0.122	0	0	0	0	0	0	0	NA	0.1048
P39707	0.32	1	0.0145	0	0	0	0.0145	0	NA	0.1148
Q12507	0.7803	4	0.1098	0	0.1705	0	0.1792	0	NA	0.6604
P35735	0.1926	0	0	0	0	0	0.0793	0	NA	0.1535
P29478	0.5055	2	0.3516	1	0.304	1	0.3004	1	NA	0.4563

P40482	0.324	1	0.1976	2	0.1501	1	0.1145	1	NA	0.4204
Q03029	0.3901	0	0.0673	0	0	0	0.0448	0	NA	0.2325
Q04438	0.9043	1	0.7739	1	0.9391	1	0.8696	1	NA	0.9766
P23624	0.7457	3	0.6048	1	0.4261	1	0.4502	1	NA	0.6925
Q12267	0.6685	3	0.2652	4	0.1453	2	0.1721	2	NA	0.726
P38989	0.5658	4	0.1145	0	0	0	0.0385	0	NA	0.2213
P22082	0.5385	5	0.4228	3	0.3605	3	0.3629	3	NA	0.8769
P18480	0.6519	4	0.5028	5	0.3978	4	0.3845	4	NA	0.9963
P32364	0.6387	2	0.2439	1	0	0	0.2073	1	NA	0.8542
Q04629	0.0863	0	0	0	0	0	0	0	NA	0.1384
P38123	0.0667	0	0	0	0	0	0.0095	0	NA	0.1112
P36085	0.4648	4	0.0339	0	0	0	0.0653	0	NA	0.2615
Q00947	0.7168	3	0.3815	1	0.2871	1	0.2948	1	TRU	0.9469
P53598	0.152	0	0.0365	0	0	0	0.0638	0	NA	0.274
Q06525	0.5032	3	0.2559	1	0.2925	1	0.3097	1	NA	0.7107
P32861	0.1944	1	0.0481	0	0	0	0.0341	0	NA	0.1496
Q12151	0.6473	3	0.4622	5	0.4304	6	0.3888	5	TRU	0.9982
P53146	0.7592	2	0.2	0	0	0	0.1061	0	NA	0.2321
P40343	0.701	3	0.4968	2	0.4952	3	0.4759	3	NA	0.9736
P23968	0.1221	0	0.0047	0	0	0	0	0	NA	0.1466
P40055	0.509	2	0.3267	2	0.1282	1	0.1408	1	NA	0.4314
P87012	0.3856	1	0	0	0	0	0.0261	0	NA	0.1411
P34224	0.285	0	0	0	0	0	0.057	0	NA	0.1206
P38190	0.7097	1	0	0	0	0	0.5	1	NA	0.9925
P38184	0.1068	0	0	0	0	0	0	0	NA	0.1665
Q8J0M4	0.4701	1	0.0672	0	0	0	0.1119	0	NA	0.2023
P25562	0.152	0	0	0	0	0	0	0	NA	0.1592
Q12491	0.4435	5	0.3418	6	0.2887	6	0.278	7	NA	0.8857
Q08976	0.1667	0	0	0	0	0	0	0	NA	0.1055
Q2V2P2	0.6849	1	0	0	0	0	0.1644	0	NA	0.1167
P35736	0.7495	6	0.5282	6	0.4295	4	0.4425	5	NA	0.9821
Q06089	0.1553	0	0	0	0	0	0	0	NA	0.1042
Q12010	0.2305	1	0.0714	0	0.1136	1	0.1039	0	NA	0.3162
Q12249	0.1295	0	0	0	0	0	0.0647	0	NA	0.1709
Q03263	0.1556	1	0.013	0	0	0	0.0463	0	NA	0.12
Q8TGS4	0.6	0	0	0	0	0	0.2571	0	NA	0.0248
P0C5P6	0.3243	0	0	0	0	0	0.2703	0	NA	0.0248
P47053	0.1323	0	0.0233	0	0	0	0.0428	0	NA	0.1396
Q99401	0.814	1	0	0	0	0	0	0	NA	0.5562
P0CX63	0.4492	4	0.3469	6	0.2825	5	0.2757	7	NA	0.8815
Q08504	0.1296	0	0	0	0	0	0	0	NA	0.1264
Q45U48	0.0959	0	0	0	0	0	0.0685	0	NA	0.0931
P47115	0.7924	2	0.589	2	0.5424	2	0.5381	1	NA	0.9854
P0CX96	0.2125	0	0	0	0	0	0	0	NA	0.1412
P53229	0.2	0	0	0	0	0	0.0833	0	NA	0.1055
P53278	0.9657	2	0.8284	5	0.587	5	0.5564	4	NA	0.9959
P53282	0.2984	0	0	0	0	0	0.0242	0	NA	0.1834
P53287	0.3874	0	0.045	0	0	0	0	0	NA	0.1296
Q3E786	0.2727	0	0	0	0	0	0	0	NA	0.1201
P42900	0.4199	2	0.1042	0	0.098	1	0.1244	1	NA	0.8141
P50278	0.3084	1	0.162	1	0.1807	1	0.2025	1	NA	0.3726
Q00772	0.3368	1	0.2211	1	0.2273	1	0.2211	1	NA	0.1719

P53852	0.2686	2	0.0847	0	0.0926	1	0.1004	1	NA	0.3076
P36104	0.1763	1	0	0	0	0	0.0182	0	NA	0.1137
P25345	0.2256	0	0.0081	0	0	0	0.0305	0	NA	0.2139
P50078	0.7862	2	0.4534	3	0.3006	2	0.3457	1	NA	0.9388
P17536	1	1	0.9045	1	0.9095	1	0.8141	2	NA	0.9586
P22217	0.1456	0	0	0	0	0	0.1262	0	NA	0.1039
P52492	0.1346	0	0	0	0	0	0.0641	0	NA	0.136
P50623	0.4904	1	0.1401	0	0	0	0.2166	0	NA	0.2857
P28274	0.1399	0	0.0121	0	0	0	0.0121	0	NA	0.2267
Q04179	0.0941	0	0	0	0	0	0.0265	0	NA	0.1995
Q02931	0.2366	2	0.0413	0	0	0	0.0413	0	NA	0.1702
P22219	0.1781	2	0.0392	0	0	0	0.0309	0	NA	0.1704
P53950	0.8283	3	0.782	6	0.6326	6	0.5828	6	NA	0.9999
Q07468	0.205	1	0.0124	0	0	0	0.0276	0	NA	0.317
P40478	0.1246	0	0.0036	0	0	0	0.0214	0	NA	0.1272
Q08831	0.8241	2	0.7017	3	0.6577	3	0.6157	3	NA	0.9985
P34215	0.0673	0	0	0	0	0	0.0481	0	NA	0.0992
P38269	0.2521	0	0	0	0	0	0	0	NA	0.2867
A0A023PYD	0.1643	0	0	0	0	0	0.0357	0	NA	0.133
A0A023PZ9	0.5948	1	0.1293	0	0.2155	0	0.1983	0	NA	0.8366
P25630	0.1471	0	0.0098	0	0	0	0.0588	0	NA	0.0943
Q03864	0.1091	0	0	0	0	0	0	0	NA	0.1069
O74302	0.6432	2	0.5841	3	0.5545	3	0.5591	3	NA	0.9961
P39992	0.3534	4	0.0865	0	0	0	0.1364	0	NA	0.6264
Q12240	0.5693	1	0.3431	0	0.2847	0	0.292	0	NA	0.6232
O43137	0.5647	0	0.0118	0	0	0	0.2824	0	NA	0.3546
P87281	0.486	1	0	0	0	0	0	0	NA	0.1438
P87268	0	0	0	0	0	0	0	0	NA	0.1073
Q3E794	0.7547	1	0.3585	0	0.6226	0	0.6226	0	NA	0.9192
P0CX71	0.6432	2	0.5773	3	0.5523	3	0.5591	3	NA	0.9958
P38320	0.2047	0	0	0	0	0	0	0	NA	0.145
Q04069	0.1359	0	0	0	0	0	0	0	NA	0.0948
P0CX94	0.2125	0	0	0	0	0	0	0	NA	0.1412
P47003	0.4874	1	0.0924	0	0	0	0.2773	0	NA	0.2042
Q3E795	1	1	1	1	0.8469	2	0.7959	1	NA	0.9899
P0CL35	0.2125	0	0	0	0	0	0	0	NA	0.1461
P0CE99	0.3571	0	0	0	0	0	0.1143	0	NA	0.0918
P53120	0.3158	6	0.155	3	0.155	4	0.1632	3	NA	0.6096
O13520	0.2198	0	0	0	0	0	0	0	NA	0.4454
Q03675	0.381	0	0.0159	0	0	0	0.0873	0	NA	0.157
Q04773	0.6138	2	0.4759	1	0.5586	1	0.4414	1	NA	0.9367
Q08540	0.0519	0	0	0	0	0	0	0	NA	0.1016
P0C5P2	0.4828	0	0.4483	0	0	0	0.5862	0	NA	0.0248
Q02864	0.5128	1	0.2051	0	0.2308	0	0.2564	0	NA	0.7234
Q12293	0.6347	2	0.5731	3	0.4703	3	0.468	3	NA	0.8537
Q08741	0.2736	0	0.0094	0	0	0	0.0755	0	NA	0.172
O13533	0.2245	0	0	0	0	0	0.0918	0	NA	0.1259
Q06813	0.7285	4	0.3253	0	0.2446	1	0.293	1	NA	0.5858
P53329	0.6552	3	0.431	0	0	0	0.3218	0	NA	0.3705
O13568	0.3037	1	0	0	0	0	0.037	0	NA	0.5086
Q08844	0.1124	1	0	0	0	0	0.0014	0	NA	0.1089
P53336	0.2727	0	0.0411	0	0	0	0.132	0	NA	0.3515

P53098	0.2982	0	0	0	0	0	0.2807	0	NA	0.2473
Q12470	0.6341	2	0.5795	3	0.5568	3	0.5614	3	NA	0.9941
Q07990	0.6211	1	0.1553	0	0.1615	0	0.2981	1	NA	0.8967
P53321	0.3356	1	0	0	0	0	0.0616	0	NA	0.2414
Q04814	0.4104	0	0.0522	0	0	0	0.1418	0	NA	0.2879
Q3E7B5	0.0484	0	0	0	0	0	0	0	NA	0.1123
Q6Q5H1	0.6432	2	0.5773	3	0.5341	3	0.5432	3	NA	0.9956
Q06251	0.4586	2	0.2755	1	0.215	1	0.2022	1	NA	0.5173
P38870	0.4038	3	0.121	1	0.1389	1	0.1503	2	NA	0.4393
P53870	0.2401	2	0.1559	0	0.172	0	0.1774	1	NA	0.2468
P53929	0.4333	1	0.1037	0	0	0	0.0926	0	NA	0.3204
P53750	0.0379	0	0	0	0	0	0	0	NA	0.1055
P38893	0.1613	0	0.0176	0	0	0	0.0205	0	NA	0.1391
A0A023PXL	0.2292	0	0	0	0	0	0	0	NA	0.4273
P32792	0.1688	1	0.026	0	0	0	0.0649	0	NA	0.1488
P53740	0.1578	1	0.0382	0	0	0	0.0662	0	NA	0.1819
P40587	0.1304	0	0	0	0	0	0.0348	0	NA	0.1235
P47152	0.5444	1	0.1065	0	0	0	0.213	0	NA	0.6846
P53227	0.6162	1	0.4686	1	0	0	0.2251	0	NA	0.2468
P40062	0.1927	0	0	0	0	0	0	0	NA	0.1257
P43576	0.1538	0	0	0	0	0	0	0	NA	0.1168
A0A023PYI2	0.1455	0	0	0	0	0	0	0	NA	0.1497
P53268	0.0465	0	0	0	0	0	0	0	NA	0.1497
Q04210	0.1562	0	0	0	0	0	0	0	NA	0.1003
P53107	0.5023	2	0.2795	1	0.2591	1	0.2705	1	NA	0.6084
Q06102	0.4231	2	0.0913	0	0	0	0.0865	0	NA	0.2747
Q01080	0.5904	3	0.1205	0	0	0	0.1253	0	NA	0.3674
Q12464	0.3142	0	0.1168	0	0	0	0.0955	0	NA	0.1748
P32368	0.1348	0	0.0064	0	0	0	0.0321	0	NA	0.1372
P40541	0.3304	3	0.2052	2	0.1983	2	0.2017	2	NA	0.3505
P17558	0.6792	3	0.2327	0	0	0	0.1792	0	NA	0.6359
P48415	0.8251	7	0.7376	12	0.6169	8	0.5759	9	NA	1
P36094	0.9752	2	0.6446	3	0.4298	2	0.4353	2	NA	0.932
P41808	0.1134	1	0	0	0	0	0.0387	0	NA	0.1479
P38915	0.4867	2	0.3505	2	0.3372	2	0.3389	2	NA	0.8472
P38904	0.9268	5	0.7463	5	0.6955	4	0.6857	8	NA	0.9995
Q08817	0.4968	3	0.292	2	0.2807	3	0.2895	3	NA	0.9054
P38869	0.1579	0	0.0132	0	0	0	0.0482	0	NA	0.1302
Q02521	0.8486	1	0.3514	0	0.4541	1	0.5027	1	NA	0.8546
Q01476	0.3042	3	0.103	2	0.0818	1	0.0747	1	NA	0.4327
P21576	0.3736	2	0.1477	1	0.1165	1	0.1307	1	NA	0.5441
O13584	0.0566	0	0	0	0	0	0	0	NA	0.125
P53285	0.167	1	0.0428	0	0	0	0.0685	0	NA	0.2511
P38329	0.3036	3	0.1018	1	0	0	0.1	1	NA	0.5584
P17255	0.1942	2	0.0243	0	0	0	0.0327	0	NA	0.146
P41806	0.2208	0	0.1169	0	0	0	0.2338	0	NA	0.1573
Q04338	0.6774	2	0.1705	0	0	0	0.0737	0	NA	0.2742
P47165	0.1579	0	0.067	0	0	0	0.0766	0	NA	0.1179
Q04585	0.2853	2	0.1273	0	0.1259	0	0.1371	0	NA	0.3036
P25571	0.0976	0	0	0	0	0	0	0	NA	0.1605
Q3E741	0.2	0	0	0	0	0	0.1667	0	NA	0.0248
O13514	0.736	2	0	0	0	0	0.112	0	NA	0.1679

Q3E791	0.1042	0	0	0	0	0	0.0521	0	NA	0.1058
Q07435	0.1284	0	0	0	0	0	0	0	NA	0.202
P25351	0.2308	2	0.0949	1	0.1473	2	0.1457	2	NA	0.2942
Q8TGP7	0	0	0	0	0	0	0	0	NA	0.0248
P25657	0.4	1	0	0	0	0	0.0129	0	NA	0.1042
Q12352	0.3419	1	0	0	0	0	0	0	NA	0.1961
Q12298	0.1503	1	0.026	0	0	0	0.0315	0	NA	0.1386
Q12185	0.1187	0	0	0	0	0	0.0404	0	NA	0.1415
Q8TGP8	0.1806	0	0	0	0	0	0.0417	0	NA	0.2338
A0A023PXH	0.3021	0	0	0	0	0	0	0	NA	0.1427
P0C2I2	0.4615	4	0.3373	4	0.3026	4	0.3031	4	NA	0.9126
P40017	0.4529	3	0.2178	2	0.1885	1	0.1831	1	NA	0.8139
Q8TGR6	0	0	0	0	0	0	0	0	NA	0.0248
Q05530	0.1491	0	0	0	0	0	0.0702	0	NA	0.0996
P0C289	0.1897	0	0	0	0	0	0	0	NA	0.133
Q8TGP6	0.8298	1	0.0638	0	0	0	0.4043	0	NA	0.0248
P87266	0.4555	1	0	0	0	0	0.0733	0	NA	0.1948
P40101	0.1569	0	0	0	0	0	0.0359	0	NA	0.1752
P38338	0.1628	1	0.016	0	0	0	0.0407	0	NA	0.155
P34248	0.247	3	0.0528	0	0.0818	1	0.0784	0	NA	0.1385
O94086	0.1548	0	0	0	0	0	0	0	NA	0.159
P47022	0.5388	2	0.0822	0	0	0	0.0868	0	NA	0.2823
Q05898	0.3796	0	0	0	0	0	0.1019	0	NA	0.2444
P47021	0.2897	0	0.0841	0	0	0	0.0841	0	NA	0.1695
P46991	0.3118	1	0	0	0	0	0.0294	0	NA	0.1481
Q03099	0.3421	2	0.1572	1	0.1572	1	0.1885	1	NA	0.902
Q12531	0.7085	2	0.2591	0	0	0	0.2105	0	NA	0.6345
P36058	0.2189	0	0	0	0	0	0	0	NA	0.2704
P0CY01	0.2125	0	0	0	0	0	0	0	NA	0.1441
P0C5Q7	0.5833	0	0.0833	0	0	0	0.3889	0	NA	0.1561
P47012	0.4	0	0	0	0	0	0.2952	0	NA	0.2192
O13585	0.3998	4	0.1791	2	0.1914	3	0.1723	2	NA	0.5332
P0CL39	0.2125	0	0	0	0	0	0	0	NA	0.1411
P0C272	0.4603	0	0	0	0	0	0.4127	0	NA	0.4279
P36128	0.1844	0	0	0	0	0	0.1489	0	NA	0.2237
Q870I1	0.0417	0	0	0	0	0	0	0	NA	0.1114
P53326	0.1441	0	0.02	0	0	0	0.03	0	NA	0.1386
Q02873	0.75	2	0.4113	0	0	0	0.3629	0	NA	0.7831
Q8TGS2	0.4	0	0	0	0	0	0.38	0	NA	0.3087
P53100	0.1873	0	0.0053	0	0	0	0.0501	0	NA	0.1214
P39523	0.9873	2	0.8378	6	0.6257	4	0.6182	5	NA	0.9989
Q12282	0.4322	1	0.1017	0	0	0	0.0932	0	NA	0.2138
Q12179	0.2225	1	0.0683	0	0.0683	0	0.0683	0	NA	0.141
Q6B0Y7	0.2397	0	0	0	0	0	0	0	NA	0.1282
A2P2R3	0.2252	0	0.0229	0	0	0	0.0305	0	NA	0.1299
O13532	0.1589	0	0	0	0	0	0.0561	0	NA	0.1023
Q03697	0.1719	0	0	0	0	0	0.0181	0	NA	0.147
Q12259	0.2186	2	0.0412	0	0	0	0.0515	0	NA	0.1922
Q99296	0.4699	4	0.2521	2	0.211	2	0.2425	2	NA	0.9618
P54072	0.3385	2	0.0851	0	0	0	0.0885	0	NA	0.2024
P38430	0.115	0	0.0032	0	0	0	0.0128	0	NA	0.1324
Q08503	0.3448	0	0	0	0	0	0.0603	0	NA	0.132

P47126	0.156	0	0	0	0	0	0	0	NA	0.0986
P38752	0.0923	0	0	0	0	0	0	0	NA	0.1056
P43625	0.3841	1	0	0	0	0	0.1391	0	NA	0.172
Q03186	0	0	0	0	0	0	0	0	NA	0.1056
P42936	0.12	0	0	0	0	0	0.0489	0	NA	0.1079
Q3E807	0.1912	0	0	0	0	0	0	0	NA	0.117
P47173	0.1579	0	0	0	0	0	0.038	0	NA	0.1312
Q12269	0.4462	4	0.3368	4	0.3043	4	0.3083	4	NA	0.9099
P53327	0.1586	2	0.0102	0	0	0	0.0097	0	NA	0.1641
Q04921	0.4184	2	0.1868	0	0.1348	0	0.1773	0	NA	0.3899
Q05471	0.469	3	0.3012	5	0.2642	3	0.2523	4	NA	0.7838
P08459	0.3167	1	0.0677	1	0.0697	1	0.0876	1	NA	0.1699
P37297	0.1774	5	0.0068	0	0	0	0.0184	0	NA	0.1574
Q06677	0.6796	5	0.4341	3	0.3368	2	0.3578	2	NA	0.8384
P25379	0.1389	0	0.0028	0	0	0	0.025	0	NA	0.1528
P00958	0.2477	0	0.0093	0	0	0	0.0226	0	NA	0.2857
P26637	0.245	3	0.0431	0	0	0	0.0486	0	NA	0.2343
Q6B2U8	0.0952	0	0	0	0	0	0.0476	0	NA	0.0989
P39077	0.1461	0	0.0225	0	0	0	0.0243	0	NA	0.1313
P46677	0.5938	6	0.303	4	0.1829	2	0.1848	2	NA	0.8532
Q04562	0.1235	0	0.0134	0	0	0	0.0208	0	NA	0.126
Q02776	0.5168	3	0.1723	1	0.1534	1	0.1597	1	NA	0.4824
Q07748	0.1382	0	0	0	0	0	0	0	NA	0.1166
Q03774	0.25	1	0.0901	1	0.1126	1	0.1239	1	NA	0.2179
P80967	0.58	0	0.2	0	0.48	0	0.5	0	NA	0.6252
P40061	0.3056	2	0.1923	2	0.1545	2	0.1545	2	NA	0.3722
P52490	0.366	0	0.085	0	0	0	0.1176	0	NA	0.2291
P00912	0.192	0	0	0	0	0	0.058	0	NA	0.1671
Q12063	0.288	1	0.1947	1	0.1787	1	0.192	1	NA	0.31
Q12059	0.1537	0	0	0	0	0	0.0216	0	NA	0.1743
P53044	0.4931	1	0.2493	2	0.2715	1	0.2909	1	NA	0.7728
P33202	0.2832	2	0.1416	1	0.1227	1	0.1362	1	NA	0.3964
P35194	0.1681	2	0.0193	1	0.0265	1	0.0305	1	NA	0.1504
P32563	0.1595	1	0.019	0	0	0	0.0571	0	NA	0.2
P36095	0.8348	2	0.3571	1	0	0	0.125	0	NA	0.3843
P47061	0.2798	3	0.0353	0	0	0	0.0706	0	NA	0.1646
P16140	0.1644	1	0.0735	1	0.0754	1	0.0716	1	NA	0.3159
P40080	0.6305	1	0.3153	2	0.3498	1	0.3596	1	NA	0.6094
P36172	0.1168	1	0.0155	0	0	0	0.055	0	NA	0.1456
P32366	0.1768	0	0	0	0	0	0	0	NA	0.1109
P38188	0.2368	0	0.0175	0	0	0	0.1579	0	NA	0.1094
P37264	0.0656	0	0	0	0	0	0	0	NA	0.1008
Q8TGU4	0.1667	0	0	0	0	0	0.1296	0	NA	0.1633
P38355	0.178	1	0.0703	0	0	0	0.0867	0	NA	0.1661
P39953	0.203	1	0.0388	0	0	0	0.0537	0	NA	0.3922
P39564	0.8012	2	0.0062	0	0	0	0	0	NA	0.3529
Q04978	0.8255	1	0.5142	1	0.1604	0	0.2406	0	NA	0.5038
Q05863	1	1	0.6774	1	0.6065	1	0.5484	1	NA	0.7899
Q12222	0.1867	1	0	0	0	0	0.0267	0	NA	0.1396
P40898	0.1497	0	0	0	0	0	0	0	NA	0.1386
P53143	0.2887	1	0	0	0	0	0.1197	0	NA	0.1807
P53085	0.5044	1	0	0	0	0	0.1239	0	NA	0.5333

P36050	0.0079	0	0	0	0	0	0	0	NA	0.1084
O13586	0.4455	0	0	0	0	0	0.1683	0	NA	0.0955
Q3E767	0.2826	0	0	0	0	0	0.2826	0	NA	0.0248
P36031	0.0364	0	0	0	0	0	0.0091	0	NA	0.0982
P38616	0.2881	1	0.0395	0	0	0	0.1045	0	NA	0.4326
P53056	0.1053	0	0	0	0	0	0.0658	0	NA	0.0956
P36111	0.4665	3	0.3292	3	0.2306	2	0.2711	2	NA	0.5993
P36119	0.7981	3	0.5642	4	0.3717	2	0.4075	2	NA	0.8957
Q3E7Y8	0.3864	0	0	0	0	0	0.1818	0	NA	0.0248
Q3E814	0.2414	0	0	0	0	0	0.2241	0	NA	0.1682
P36098	0.5766	1	0.1168	0	0	0	0.2044	0	NA	0.1809
Q12152	0.6981	3	0.4384	4	0.3097	3	0.3518	3	NA	0.904
Q02826	0.213	0	0	0	0	0	0.0648	0	NA	0.1204
Q12444	0.754	1	0.5238	1	0.5635	1	0.5794	1	NA	0.9017
A0A023PYK	0.3243	0	0.0541	0	0	0	0.0811	0	NA	0.1269
Q08272	0.155	0	0	0	0	0	0	0	NA	0.1164
Q08728	0.4296	1	0	0	0	0	0.0667	0	NA	0.2041
Q04215	0.6432	2	0.5886	3	0.5909	3	0.575	3	NA	0.9947
Q8TGT6	0.2414	0	0	0	0	0	0.2414	0	NA	0.0248
Q8TGS5	0.1143	0	0	0	0	0	0.0571	0	NA	0.0248
Q6B0W2	0.1927	0	0	0	0	0	0	0	NA	0.1126
Q12113	0.4452	4	0.3452	6	0.287	7	0.2814	6	NA	0.9169
Q12077	0.6481	1	0.1111	0	0	0	0.2963	0	NA	0.2345
P0CF19	0.1907	1	0	0	0	0	0.0837	0	NA	0.1353
Q05854	0.6122	5	0.4952	8	0.3497	6	0.3661	6	NA	0.9881
Q12274	0.5429	1	0.2229	0	0	0	0.2686	0	NA	0.4178
P36109	0.152	0	0	0	0	0	0.024	0	NA	0.1337
A0A0B7P3V	0.4601	4	0.2038	1	0	0	0.1141	0	NA	0.2797
P40536	0.5339	1	0.3644	0	0.4831	1	0.5	1	NA	0.9553
P40500	0.2683	0	0	0	0	0	0.1024	0	NA	0.1256
P43562	0.1685	1	0.0444	0	0.0907	1	0.0944	1	NA	0.1665
P38833	0.535	2	0.4609	1	0.3169	1	0.3086	1	NA	0.3743
Q02598	0.1731	0	0	0	0	0	0	0	NA	0.1515
P0C271	0.475	0	0.375	0	0	0	0.675	0	NA	0.0248
P0C5N7	0.3103	0	0	0	0	0	0.1034	0	NA	0.1238
P38867	0.6755	2	0.5077	4	0.3642	2	0.3113	1	NA	0.9601
Q3E7Z3	0.4222	0	0	0	0	0	0.2444	0	NA	0.0248
P40588	0.3607	0	0.0164	0	0	0	0.1639	0	NA	0.2125
P53910	0.3228	1	0.0688	0	0	0	0.1058	0	NA	0.1945
P40076	0.693	1	0.4474	0	0.386	0	0.2982	0	NA	0.4588
P47132	0.2414	0	0	0	0	0	0	0	NA	0.1251
P53758	0.3929	0	0	0	0	0	0.3452	0	NA	0.5507
P53975	0.1761	0	0.0035	0	0	0	0.0423	0	NA	0.2144
P53231	0.2966	0	0.1441	0	0.2119	0	0.2288	0	NA	0.1275
P38230	0.1108	0	0	0	0	0	0.024	0	NA	0.1305
P39729	0.271	1	0.0027	0	0	0	0.0379	0	NA	0.2693
Q12188	0.5338	3	0.3015	1	0.2824	3	0.2838	2	NA	0.845
P36054	0.4976	1	0.3981	2	0	0	0.3649	0	NA	0.304
P38234	0.1905	0	0	0	0	0	0.0238	0	NA	0.1157
Q12044	0.7434	1	0.5623	2	0.5736	2	0.5509	3	NA	0.9891
P40395	0.1761	1	0.0303	0	0	0	0.0436	0	NA	0.2141
P0CX23	0.2209	0	0	0	0	0	0.064	0	NA	0.186

O14455	0.78	2	0.25	0	0	0	0.23	0	NA	0.1806
P51402	1	1	0.75	1	0	0	0.7955	1	NA	0.8558
P32904	0.3037	0	0.0561	0	0	0	0.0374	0	NA	0.1619
P40693	0.5062	1	0.2236	0	0.2236	1	0.236	1	NA	0.2257
Q12338	0.5818	1	0.2273	0	0.3	0	0.3364	0	NA	0.5484
P34087	0.117	0	0.0058	0	0	0	0.0468	0	NA	0.1281
P22139	0.2	0	0	0	0	0	0.1286	0	NA	0.1109
Q06506	0.3717	3	0.1972	1	0.1431	1	0.1553	1	NA	0.2884
P40470	1	1	0.6117	0	0.3835	1	0.466	1	NA	0.6559
P28778	0.3664	0	0	0	0	0	0.0305	0	NA	0.1109
P38200	1	1	0.8905	5	0.7012	4	0.6864	5	NA	0.9984
P53011	0.2464	1	0.0544	0	0.1032	0	0.1261	0	NA	0.247
Q03529	0.2031	0	0.0104	0	0	0	0.0391	0	NA	0.1308
P38985	0.6233	2	0.2877	0	0.3562	0	0.4452	0	NA	0.4133
P20424	0.5712	1	0.2699	1	0.2181	1	0.1959	1	NA	0.3674
P23179	0.1809	1	0.0829	0	0	0	0.093	0	NA	0.1782
Q12749	0.5539	5	0.1912	2	0.1032	1	0.1014	1	NA	0.3303
P47037	0.6146	6	0.0878	0	0	0	0.0382	0	NA	0.263
Q06160	0.5053	2	0.233	1	0.1982	1	0.2073	1	NA	0.6595
P53823	0.0586	0	0	0	0	0	0.0135	0	NA	0.104
P32558	0.4039	4	0.229	3	0.1836	3	0.1874	3	NA	0.4763
P12685	0.5725	4	0.5053	4	0.4583	5	0.4478	5	NA	0.9878
P38129	0.4536	2	0.3296	3	0.3095	2	0.3045	3	NA	0.5137
P38793	0.1864	2	0.0782	0	0.0661	0	0.0762	0	NA	0.1957
P35169	0.1619	2	0.0215	0	0	0	0.0206	0	NA	0.2873
Q12094	0.5399	2	0.4026	3	0.4505	2	0.4345	3	NA	0.9385
P29509	0.232	1	0.0282	0	0	0	0.0251	0	NA	0.1639
P25372	0.0787	0	0	0	0	0	0.0315	0	NA	0.0902
Q05776	0.4057	1	0.0171	0	0	0	0.0514	0	NA	0.1189
P25386	0.6676	2	0.405	7	0.2654	5	0.252	5	NA	0.8279
P38932	0.2097	1	0.0208	0	0	0	0.0225	0	NA	0.123
P41807	0.1946	0	0	0	0	0	0.0167	0	NA	0.1251
O13549	0.287	0	0.0093	0	0	0	0.213	0	NA	0.355
P54787	0.4612	2	0.1973	0	0.2306	1	0.2217	1	NA	0.2528
P38180	0.9728	1	0.7663	3	0.7527	3	0.6576	3	NA	0.9992
Q03899	0.1313	1	0	0	0	0	0.0108	0	NA	0.1311
Q12235	0.1507	0	0.0056	0	0	0	0.0188	0	NA	0.1642
Q04608	0.4537	1	0.0772	0	0	0	0.1605	1	NA	0.2653
P25620	0.7105	1	0	0	0	0	0.0614	0	NA	0.1996
P0C5L2	0.125	0	0	0	0	0	0	0	NA	0.0889
P0C1Z1	0.2069	0	0	0	0	0	0.2069	0	NA	0.0248
Q12135	0.1698	0	0	0	0	0	0	0	NA	0.1924
P87270	0.2538	0	0	0	0	0	0.0769	0	NA	0.1525
P42937	0.1284	0	0	0	0	0	0.0541	0	NA	0.179
Q8TGQ6	0.5714	0	0	0	0	0	0.3265	0	NA	0.5459
P0C5M7	0.0952	0	0	0	0	0	0	0	NA	0.0905
O13522	0.4458	1	0	0	0	0	0	0	NA	0.1846
Q3E7X8	0.3892	3	0.2028	1	0.202	1	0.2216	1	NA	0.9131
P0C5K9	0.1111	0	0	0	0	0	0.2222	0	NA	0.0248
P0C5M8	0.5172	0	0.1724	0	0	0	0.4483	0	NA	0.0248
A0A023PZG	0.2871	0	0	0	0	0	0.0396	0	NA	0.133
O13544	0.2	0	0	0	0	0	0	0	NA	0.118

P39559	0.0714	0	0	0	0	0	0	0	NA	0.0919
Q8TGT1	0.1837	0	0	0	0	0	0.102	0	NA	0.1708
Q06479	0.2677	2	0.1252	1	0.1363	2	0.1301	1	NA	0.4591
O13579	0.371	0	0.0645	0	0	0	0.25	0	NA	0.4172
Q04521	0.0098	0	0	0	0	0	0	0	NA	0.0989
P46987	0.311	2	0.0458	0	0	0	0.0818	0	NA	0.254
Q03208	0.8543	3	0.5126	2	0.4146	2	0.423	3	NA	0.9778
P36042	0.8912	1	0	0	0	0	0.1451	0	NA	0.6714
O13564	0.1491	0	0.0088	0	0	0	0.1228	0	NA	0.1247
P34238	0.2857	0	0	0	0	0	0	0	NA	0.1552
P53831	0.0894	0	0.0081	0	0	0	0.0325	0	NA	0.1243
Q08293	0.1456	0	0	0	0	0	0.068	0	NA	0.125
Q02606	0.7927	2	0.4908	1	0.4147	1	0.4173	1	NA	0.9744
P0C5E1	0.2031	0	0	0	0	0	0	0	NA	0.2035
Q08748	0.2467	4	0.0504	0	0	0	0.0729	1	NA	0.3128
Q99326	0.5675	3	0.2727	0	0.1791	1	0.2066	1	NA	0.5325
Q08994	0.1961	0	0	0	0	0	0.0686	0	NA	0.1014
P53092	0.8269	2	0.0833	0	0	0	0.141	0	NA	0.6262
P36134	0.7	1	0.472	1	0.42	1	0.468	1	NA	0.9385
Q3E766	0.125	0	0	0	0	0	0	0	NA	0.1103
Q8TGR9	0.375	0	0	0	0	0	0.1875	0	NA	0.0248
Q08912	0.2724	1	0.1122	1	0.0994	1	0.1106	1	NA	0.4833
Q6Q572	0.3426	0	0	0	0	0	0.0648	0	NA	0.1593
P43587	1	1	0.9742	2	0.9806	2	0.9677	2	NA	0.9955
Q6B0W0	0.2342	0	0.0901	0	0	0	0.1982	0	NA	0.2749
Q08373	0.1339	0	0	0	0	0	0	0	NA	0.1216
Q08984	0.2805	2	0.0213	0	0	0	0.0696	0	NA	0.1592
P53183	0.1868	0	0.0057	0	0	0	0.0374	0	NA	0.1369
Q3E765	0.2429	0	0	0	0	0	0	0	NA	0.1018
O13571	0.1743	0	0	0	0	0	0	0	NA	0.1122
Q05016	0.1386	0	0.0112	0	0	0	0.0524	0	NA	0.1346
Q8TGR1	0.6061	0	0	0	0	0	0.3333	0	NA	0.0248
Q6B0R7	0.098	0	0	0	0	0	0	0	NA	0.1314
P0CX99	0.2125	0	0	0	0	0	0	0	NA	0.1441
P40434	0.3652	4	0.1758	1	0.1854	1	0.1985	1	NA	0.8908
Q8TGK0	0.0404	0	0	0	0	0	0	0	NA	0.0875
P40575	0.13	0	0	0	0	0	0.01	0	NA	0.0968
P53749	0.3163	2	0.0362	0	0	0	0.0659	0	NA	0.2131
O13535	0.4523	4	0.3246	4	0.2962	4	0.2967	4	NA	0.9107
P53928	0.1602	0	0	0	0	0	0	0	NA	0.1202
P53932	0.433	2	0.2508	1	0.2056	2	0.2025	2	NA	0.4004
P0CD97	0.2222	0	0	0	0	0	0.2222	0	NA	0.2011
P0C074	0.6941	1	0.1412	0	0	0	0.1647	0	NA	0.1408
P33338	0.5517	4	0.2624	2	0	0	0.1178	1	NA	0.5695
P18888	0.6265	2	0.3976	1	0.2349	1	0.238	1	NA	0.6657
P47154	0.117	0	0	0	0	0	0.0199	0	NA	0.1609
P23561	0.4059	5	0.1785	1	0.1283	1	0.1255	1	NA	0.5472
P39991	0.1019	1	0.0017	0	0	0	0.0152	0	NA	0.1494
P87282	0.1789	0	0	0	0	0	0.0081	0	NA	0.1107
P0CL37	0.2075	0	0	0	0	0	0	0	NA	0.1245
P38311	0.4	0	0	0	0	0	0.1048	0	NA	0.1827
A0A023PXI8 [illegible]	0.3238	1	0.0286	0	0	0	0.1714	0	NA	0.2112

A0A023PYE	0.028	0	0	0	0	0	0	0	NA	0.0966
Q2V2P7	1	1	1	1	1	1	1	1	NA	0.995
Q04408	0.3313	2	0.0807	0	0	0	0.089	0	NA	0.1414
P40000	0.1121	0	0	0	0	0	0	0	NA	0.1222
O13540	0.4419	1	0	0	0	0	0.1008	0	NA	0.251
P38357	0.2114	0	0.0081	0	0	0	0.1138	0	NA	0.1549
Q7LIF2	0.4545	1	0.0364	0	0	0	0.1545	0	NA	0.4199
Q86ZR7	0.1568	0	0	0	0	0	0.0424	0	NA	0.1377
Q3E7B4	0.381	0	0.0317	0	0	0	0.0635	0	NA	0.0979
P36099	0.4876	1	0.0697	0	0	0	0.1592	0	NA	0.3268
Q03795	0.2742	1	0.0494	0	0	0	0.1042	1	NA	0.258
P47059	0.1346	0	0	0	0	0	0	0	NA	0.1185
P0C2I6	0.4541	4	0.3368	4	0.306	4	0.31	4	NA	0.8776
Q07967	0.7681	1	0.5437	2	0.4829	2	0.4791	2	NA	0.7368
Q04898	0.1333	0	0	0	0	0	0.1048	0	NA	0.1338
Q6B0Y8	0.848	1	0.04	0	0	0	0.416	0	NA	0.9247
Q03434	0.4507	4	0.335	4	0.3009	4	0.3094	4	NA	0.8943
Q03730	0.2239	0	0	0	0	0	0.0687	0	NA	0.2203
Q07865	0.0794	0	0	0	0	0	0	0	NA	0.1115
P47028	0.1066	0	0	0	0	0	0	0	NA	0.1475
A0A023PXI5	0.2781	0	0.0428	0	0	0	0.0749	0	NA	0.1786
P38765	0.1088	0	0	0	0	0	0.0102	0	NA	0.1171
P40503	0.2451	0	0.0098	0	0	0	0.2157	0	NA	0.1669
A0A023PZJ9	0.5935	1	0	0	0	0	0.0569	0	NA	0.1183
P40449	0.5447	1	0.4766	1	0.4213	1	0.4255	1	NA	0.4438
Q8TGN2	1	1	0.0353	0	0	0	0.4235	0	NA	0.9069
Q03787	0.2842	2	0.0027	0	0	0	0.0188	0	NA	0.1888
Q03888	0.3115	1	0.0123	0	0	0	0.0369	0	NA	0.2244
P53908	0.5538	1	0.1769	0	0	0	0.3615	0	NA	0.5966
Q08187	0.9552	2	0.1741	0	0	0	0.2189	0	NA	0.4614
P53981	0.1286	0	0	0	0	0	0.0166	0	NA	0.1211
P42942	0.18	0	0.0292	0	0	0	0.0535	0	NA	0.138
P53274	0.9826	2	0.887	2	0.7522	2	0.7391	2	NA	0.9925
P40058	0.2139	0	0.0116	0	0	0	0.0578	0	NA	0.1513
Q07451	0.3793	1	0.0542	0	0	0	0.2167	1	NA	0.3418
Q12172	0.3444	3	0.1481	1	0.1506	2	0.1741	2	TRU	0.5931
O13559	0.3111	2	0.1397	1	0.1476	1	0.1773	1	NA	0.899
P25604	0.2935	1	0.2234	1	0.2052	1	0.213	1	NA	0.3356
Q05937	0.7026	2	0.5452	2	0.484	2	0.4665	2	NA	0.9881
P36086	0.0938	0	0	0	0	0	0	0	NA	0.1264
Q06235	0.8136	2	0.3475	0	0.5169	1	0.5085	1	NA	0.949
I2HB70	0.068	0	0	0	0	0	0	0	NA	0.0815
Q3E843	0.4444	0	0	0	0	0	0.3556	0	NA	0.0248
P40365	0.2328	0	0	0	0	0	0	0	NA	0.1505
P40544	0.2225	0	0.0462	0	0	0	0.0896	0	NA	0.6332
A0A023PZL7	0.6891	1	0.4958	1	0.4286	1	0.4706	1	NA	0.4574
Q8TGS6	1	0	0.6207	0	1	0	1	0	NA	0.0248
A0A023PXQ	0.3039	1	0.0294	0	0	0	0.1961	0	NA	0.1849
P53057	0.2364	0	0.0182	0	0	0	0.0545	0	NA	0.1249
P36018	0.453	2	0.2521	0	0.2564	1	0.2564	1	NA	0.4584
Q6B0V7	0.1846	0	0	0	0	0	0	0	NA	0.1537
P0C2J3	0.4446	4	0.3435	5	0.2915	6	0.2802	6	NA	0.9129

O13573	0.0891	0	0	0	0	0	0	0	NA	0.1078
Q99260	0.4326	1	0.1581	0	0.2186	1	0.2419	1	NA	0.3577
P40551	0.1327	0	0	0	0	0	0	0	NA	0.129
P43590	0.1439	0	0.0019	0	0	0	0.0037	0	NA	0.1269
P38834	0.1712	0	0	0	0	0	0	0	NA	0.0984
P40520	0.2727	1	0.157	0	0	0	0.1074	0	NA	0.1761
P38808	0.1646	0	0	0	0	0	0.0305	0	NA	0.1082
A0A023PXG	0.2707	0	0.0677	0	0	0	0.1729	0	NA	0.2104
P47107	0.0758	0	0	0	0	0	0.0054	0	NA	0.11
P53880	0.1655	0	0	0	0	0	0.0345	0	NA	0.1655
P53737	0.4155	1	0.1408	0	0	0	0.2042	0	NA	0.6572
P40440	0.5229	1	0.3486	0	0.4679	1	0.4862	1	NA	0.898
Q8TGJ8	0.8493	1	0.3699	0	0.4658	0	0.4384	0	NA	0.8744
P40159	0.9146	2	0.9447	2	0.9899	2	0.9246	2	NA	0.9972
P53747	0.3927	1	0.1598	1	0.2283	1	0.242	1	NA	0.2087
P47099	0.6432	2	0.5864	3	0.575	3	0.5795	3	NA	0.9962
P40022	0.827	2	0.9027	3	0.6054	2	0.5784	2	NA	0.8575
P0C5N9	0.5068	1	0.0274	0	0	0	0.2192	0	NA	0.146
P53217	0.1835	0	0	0	0	0	0.0755	0	NA	0.1168
Q8TGT8	0.3214	0	0	0	0	0	0.2143	0	NA	0.0248
P38280	0.7176	4	0.4286	2	0.2939	1	0.3021	2	NA	0.7911
P41920	0.6318	1	0.3284	1	0.3731	1	0.3731	1	NA	0.716
Q12312	0.128	0	0	0	0	0	0.008	0	NA	0.1142
P31111	0.9726	2	0.5794	2	0.3291	3	0.3566	3	NA	0.8116
Q12104	0.3624	2	0.2162	1	0	0	0.1528	1	NA	0.4023
Q12286	0.4291	1	0.0187	0	0	0	0.0746	0	NA	0.2008
Q06682	0.544	3	0.454	3	0.37	3	0.344	2	NA	0.8691
P38293	0.6959	1	0.5743	1	0.4392	1	0.4595	1	NA	0.3578
P32571	0.4806	3	0.1728	2	0.162	1	0.1436	0	NA	0.7863
Q07655	0.8259	4	0.5562	4	0.4746	3	0.4268	2	NA	0.9981
P37370	0.9633	2	0.9841	2	0.9498	1	0.9645	1	NA	0.9999
P40890	0.2298	3	0.0658	1	0.0613	1	0.0671	1	NA	0.3962
P38177	0.6009	4	0.5451	3	0.4442	2	0.4742	2	NA	0.9811
Q96VH1	0.1786	0	0	0	0	0	0	0	NA	0.1795
Q96VG5	0.1538	0	0	0	0	0	0.1692	0	NA	0.1084
P53169	0.209	0	0.0421	0	0	0	0.0702	0	NA	0.2232
P38315	0.2611	3	0.0134	0	0	0	0.0549	0	NA	0.1573
P39725	0.049	0	0	0	0	0	0	0	NA	0.0954
P25617	0.7552	2	0.4345	3	0.469	2	0.469	2	NA	0.8128
Q3E774	0.1395	0	0	0	0	0	0.093	0	NA	0.0248
P0C5L8	0.1333	0	0	0	0	0	0.1111	0	NA	0.0248
Q05612	0.3048	0	0	0	0	0	0.1714	0	NA	0.2471
Q12047	0.0392	0	0	0	0	0	0	0	NA	0.0935
P38327	0.3782	1	0	0	0	0	0	0	NA	0.1233
P38322	0.0882	0	0	0	0	0	0	0	NA	0.0983
Q04018	0.4789	2	0.1127	0	0	0	0.1408	0	NA	0.8294
Q08734	0.1742	0	0	0	0	0	0.0758	0	NA	0.1406
P47048	0.3711	2	0.2378	1	0.2422	1	0.2067	1	NA	0.1989
Q99208	0.3384	2	0.1572	1	0.1594	1	0.1863	1	NA	0.9054
Q04471	0.462	2	0.3641	2	0.3696	1	0.356	1	NA	0.6443
P47044	0.3102	1	0.1633	0	0	0	0.151	0	NA	0.1896
A0A023PZH	0.4609	0	0	0	0	0	0.1826	0	NA	0.1632

Q07895	0.1334	1	0.0371	0	0	0	0.051	0	NA	0.161
Q6B0Y6	0.0661	0	0	0	0	0	0	0	NA	0.1196
Q99318	0.4257	0	0.0594	0	0	0	0.1485	0	NA	0.1243
Q08816	0.4111	2	0.277	1	0	0	0.1924	0	NA	0.238
P53069	0.1827	0	0	0	0	0	0	0	NA	0.202
P32843	0.2706	2	0.0918	0	0.0753	1	0.0765	0	NA	0.2385
P0C5P7	0.1184	0	0	0	0	0	0	0	NA	0.1047
O13546	0.6087	1	0	0	0	0	0.2348	0	NA	0.5661
P38298	0.057	0	0	0	0	0	0.0285	0	NA	0.1014
A0A023PXP	0.1364	0	0	0	0	0	0	0	NA	0.1102
A0A023PZE	0.3514	1	0	0	0	0	0.2432	0	NA	0.3986
A0A023PZH	0.099	0	0	0	0	0	0	0	NA	0.1035
P38716	0.1402	0	0.0026	0	0	0	0.0397	0	NA	0.1652
P53864	0.1912	0	0	0	0	0	0	0	NA	0.1173
A0A023PZF	0.4937	2	0.0253	0	0	0	0.1899	0	NA	0.3777
Q08241	0.0342	0	0	0	0	0	0.0342	0	NA	0.1036
Q3E802	0.4167	0	0.0278	0	0	0	0.4167	0	NA	0.0248
P40355	0.2364	2	0.0075	0	0	0	0.031	0	NA	0.145
P39978	0.2	0	0.0154	0	0	0	0.0769	0	NA	0.129
P38750	0.5327	2	0.4338	3	0.2456	3	0.2424	1	NA	0.3657
P53957	0.717	1	0.1415	0	0	0	0.3019	0	NA	0.2939
P53234	0.2898	1	0.1272	1	0.1378	1	0.1696	1	NA	0.3442
Q8TGN8	1	1	0	0	0	0	0.8065	0	NA	0.0248
P40049	0.1325	0	0.0199	0	0	0	0.0298	0	NA	0.1345
P40057	0.6797	1	0.2266	0	0.5312	1	0.4844	0	NA	0.9689
P40021	0.9693	5	0.8745	13	0.7639	11	0.7035	10	NA	0.9994
P17123	1	1	0.5723	1	0.4798	1	0.4682	1	NA	0.7447
Q12074	0.1911	0	0	0	0	0	0.0375	0	NA	0.1653
P33328	0.5652	1	0.2261	0	0.2783	1	0.2696	0	NA	0.22
Q12483	0.1073	0	0	0	0	0	0.0215	0	NA	0.1193
P00445	0.5779	1	0.5779	1	0	0	0.4481	0	NA	0.7876
P38283	0.861	1	0.8052	3	0.5831	4	0.5759	3	NA	0.9813
P31377	0.5961	1	0.2902	1	0.2275	1	0.2471	1	NA	0.4073
P32911	0.213	0	0.0463	0	0	0	0.1944	0	NA	0.1373
P25623	0.5609	2	0.4184	1	0.4333	1	0.4345	1	NA	0.9799
P30822	0.1098	1	0.0018	0	0	0	0.0138	0	NA	0.208
P39102	0.4553	2	0.0871	0	0	0	0.1329	0	NA	0.581
Q06263	0.5879	1	0.3273	1	0.3485	1	0.3455	2	NA	0.6567
P38277	0.8187	3	0.395	2	0.2328	1	0.2557	2	NA	0.9466
P38214	0.1151	0	0	0	0	0	0.0935	0	NA	0.1087
P38186	0.0283	0	0	0	0	0	0	0	NA	0.1323
Q03904	0.4414	1	0	0	0	0	0	0	NA	0.215
P39728	0.2285	1	0	0	0	0	0.0712	0	NA	0.1313
P0CX18	0.7192	2	0	0	0	0	0.1823	0	NA	0.5279
A0A023PZE	0.3654	1	0	0	0	0	0	0	NA	0.3255
Q3E796	0.1642	0	0	0	0	0	0.0448	0	NA	0.1041
Q99303	0.6142	2	0.5616	3	0.4795	3	0.4795	3	NA	0.9777
Q07613	0.211	0	0	0	0	0	0.0826	0	NA	0.124
Q07649	0.2022	0	0	0	0	0	0.071	0	NA	0.2742
Q8TGP5	0.2903	0	0	0	0	0	0.1774	0	NA	0.1151
P0C5M3	0.1765	0	0	0	0	0	0	0	NA	0.1328
Q3E755	0.0926	0	0	0	0	0	0.0926	0	NA	0.0967

P38296	0.1694	0	0	0	0	0	0.0484	0	NA	0.1723
Q8TGU8	0.2812	0	0	0	0	0	0.125	0	NA	0.0248
A0A023PZG	0.0118	0	0	0	0	0	0	0	NA	0.1491
O13523	0.2299	0	0	0	0	0	0.0374	0	NA	0.2085
P0C5L5	0.0833	0	0	0	0	0	0	0	NA	0.0248
Q12260	0.6119	2	0.5457	3	0.4566	3	0.4498	3	NA	0.9025
Q8TGP3	0.3333	0	0	0	0	0	0.3667	0	NA	0.0248
P38150	0.2095	2	0.0514	0	0	0	0.0527	0	NA	0.1819
Q3E7B7	1	1	1	1	1	1	1	1	NA	0.9915
A0A023PYF	0.1349	0	0	0	0	0	0	0	NA	0.1301
P38318	0.1196	0	0.0036	0	0	0	0.0232	0	NA	0.1194
A0A023PXH	0.7872	1	0.5319	0	0.5957	0	0.617	0	NA	0.0248
P0CX90	0.1194	0	0	0	0	0	0	0	NA	0.1814
D6VPM8	0.0782	0	0.0056	0	0	0	0	0	NA	0.1266
P0CI66	0.4444	1	0.2424	0	0	0	0.2222	0	NA	0.2817
O13553	0.3264	0	0.0139	0	0	0	0.0764	0	NA	0.3198
P0CX92	0.0825	0	0	0	0	0	0	0	NA	0.0985
Q06070	0.7149	1	0.4518	1	0.4298	1	0.4298	1	NA	0.9516
P38362	0.1394	0	0	0	0	0	0.0242	0	NA	0.1297
P36071	0.2619	0	0.0238	0	0	0	0.0238	0	NA	0.1326
Q7LIF1	0.2376	0	0	0	0	0	0.198	0	NA	0.235
Q06188	0.6118	1	0.2796	1	0.25	1	0.2763	1	NA	0.6362
Q12251	0.1472	0	0	0	0	0	0.0399	0	NA	0.1196
P53139	1	1	1	1	1	1	1	1	NA	0.9851
P53097	0.0874	0	0	0	0	0	0.068	0	NA	0.1218
Q92393	0.445	4	0.3345	4	0.3026	4	0.3009	4	NA	0.9032
Q08604	0.3211	1	0.1468	0	0	0	0.1651	0	NA	0.1257
O13582	0.3226	0	0.1371	0	0	0	0.1935	0	NA	0.1891
P36153	0.1132	0	0	0	0	0	0	0	NA	0.1137
P47147	0.3096	3	0.0581	0	0	0	0.0814	1	NA	0.3003
Q08681	0.8	1	0.14	0	0	0	0.19	0	NA	0.8367
P53113	0.1156	0	0	0	0	0	0	0	NA	0.1487
P0C5R9	0.2588	0	0	0	0	0	0	0	NA	0.1665
P53821	0.2976	0	0	0	0	0	0.131	0	NA	0.1895
Q08990	0.39	0	0.3	0	0	0	0.31	0	NA	0.6643
A0A023PXI	0.3101	1	0	0	0	0	0.1318	0	NA	0.2135
A0A023PXE	0.4151	0	0	0	0	0	0	0	NA	0.6446
Q12346	0.2464	0	0.1232	0	0	0	0.1991	0	NA	0.2778
Q2V2P1	1	1	0.871	0	0	0	0.5323	0	NA	0.7946
Q12130	0.0719	0	0	0	0	0	0.0576	0	NA	0.0982
Q6B0Z2	0.4	0	0.1048	0	0	0	0.2	0	NA	0.2104
P0CX19	0.7192	2	0	0	0	0	0.1823	0	NA	0.5279
P40162	0.15	0	0	0	0	0	0.0571	0	NA	0.1007
P53862	0.562	2	0	0	0	0	0.1085	0	NA	0.7317
P47184	0.1083	0	0	0	0	0	0	0	NA	0.1599
P53212	0.4448	3	0.169	0	0	0	0.1172	0	NA	0.2335
P47139	0.4436	3	0.1463	1	0.1631	2	0.1677	2	NA	0.3556
Q3E744	0.5761	0	0.1957	0	0	0	0.4565	0	NA	0.8256
P53947	0.8038	3	0.3354	2	0.3987	1	0.5095	2	NA	0.9963
P53210	0.596	2	0.3603	1	0.3603	2	0.3872	1	NA	0.9009
Q04174	0.0446	0	0	0	0	0	0.0039	0	NA	0.1016
P10080	0.619	3	0.4864	0	0.3537	1	0.4082	3	NA	0.7028

P36150	0.2091	1	0.0809	1	0	0	0.059	0	NA	0.2351
P25344	0.5318	1	0.4364	2	0.3873	1	0.396	2	NA	0.9144
P38223	0.04	0	0	0	0	0	0	0	NA	0.1095
P38185	0.3529	0	0.0392	0	0	0	0.1667	0	NA	0.338
P38183	0.1958	0	0	0	0	0	0	0	NA	0.1548
P38240	0.1549	0	0	0	0	0	0.0563	0	NA	0.126
P38209	0.203	0	0	0	0	0	0.1053	0	NA	0.1309
Q07355	0.4146	1	0	0	0	0	0.1382	0	NA	0.2386
Q3E818	0.2	0	0	0	0	0	0.14	0	NA	0.1453
Q99231	0.449	4	0.335	4	0.306	4	0.3123	4	NA	0.8305
Q6B113	0.1471	0	0	0	0	0	0.0294	0	NA	0.0248
A0A023PZF5	0.3578	0	0	0	0	0	0.1743	0	NA	0.254
Q04093	0.2518	2	0.2198	3	0.1878	3	0.1951	3	NA	0.3847
Q07658	0.3427	0	0.0094	0	0	0	0.0704	0	NA	0.2878
P87267	0.252	0	0	0	0	0	0.0813	0	NA	0.2361
P53175	0.3465	0	0.1287	0	0	0	0.2079	0	NA	0.2079
Q04835	0.2609	1	0.1135	0	0.1401	1	0.1425	1	NA	0.2613
Q12303	0.2441	1	0.0768	0	0.0748	0	0.1063	0	NA	0.2965
P36103	0.9422	1	0.8845	4	0.8989	2	0.8556	2	NA	0.991
Q3E832	0.1887	0	0	0	0	0	0.2075	0	NA	0.3522
Q12219	0.3673	1	0.085	0	0	0	0.1259	0	NA	0.1807
P43537	0.7714	1	0.4686	1	0.4914	1	0.6286	1	NA	0.9995
Q3E815	0.1837	0	0	0	0	0	0	0	NA	0.2552
P47023	0.3527	2	0.1473	0	0.1232	1	0.1522	1	NA	0.1608
P0C5P0	0.3548	0	0	0	0	0	0.0645	0	NA	0.3936
P53878	0.2752	1	0.086	0	0.0958	1	0.0934	0	NA	0.2374
P53887	0.0492	0	0	0	0	0	0	0	NA	0.11
P40524	0.1333	0	0	0	0	0	0	0	NA	0.1012
P0CL41	0.2062	0	0	0	0	0	0	0	NA	0.1374
A0A023PZI9	0.3312	0	0	0	0	0	0.0255	0	NA	0.1554
P53956	0.4942	1	0.0698	0	0	0	0.1919	0	NA	0.1415
Q8TGN7	0.1111	0	0	0	0	0	0	0	NA	0.1353
P47162	0.3277	0	0.1765	0	0.1849	0	0.1933	0	NA	0.2855
P0CX62	0.621	2	0.5594	3	0.4658	3	0.4635	3	NA	0.8379
Q06681	0.6398	6	0.4826	6	0.4207	8	0.42	8	NA	0.9931
P39015	1	1	0.9304	2	0.8791	2	0.8205	2	NA	0.9918
P37296	0.2112	3	0.0461	0	0	0	0.0416	0	NA	0.1623
P53599	0.3477	5	0.2084	2	0.1678	3	0.1792	3	NA	0.8132
P07284	0.3247	1	0.0693	0	0	0	0.0974	0	NA	0.2137
P49334	0.4342	1	0.1842	0	0.3289	1	0.3224	0	NA	0.3467
P36029	0.1958	1	0.0793	1	0.1019	1	0.1003	0	NA	0.1525
P52488	0.3506	3	0.1808	1	0.184	1	0.1934	1	NA	0.4644
P00931	0.1231	0	0.0113	0	0	0	0.0184	0	NA	0.2141
P35200	0.1304	0	0.0087	0	0	0	0	0	NA	0.1207
P27515	0.1896	1	0.012	0	0	0	0.0279	0	NA	0.1672
P54730	0.5817	2	0.2788	2	0.1779	1	0.1803	1	NA	0.5795
P50102	0.1953	0	0.0021	0	0	0	0.0382	0	NA	0.2618
Q12254	0.7615	1	0.1769	0	0	0	0.1077	0	NA	0.456
P12866	0.1225	0	0.0085	0	0	0	0.014	0	NA	0.1181
P40489	0.6043	4	0.3261	3	0.3447	4	0.3539	5	TRU	0.9946
P40046	0.2171	0	0	0	0	0	0.124	0	NA	0.2274
Q8TGP9	0.1207	0	0	0	0	0	0.0862	0	NA	0.16

Q3E747	1	1	0.9882	1	0.8824	1	0.8824	1	NA	0.4072
O13578	0.2857	0	0	0	0	0	0.0536	0	NA	0.1904
Q03102	0.0822	0	0	0	0	0	0.0219	0	NA	0.1419
P47020	0.243	0	0	0	0	0	0.0841	0	NA	0.1138
P47014	0.172	2	0.0013	0	0	0	0.004	0	NA	0.1795
P36061	0.3415	1	0.0341	0	0	0	0.0634	0	NA	0.2187
P0CX17	0.3472	1	0.125	0	0	0	0.1667	0	NA	0.2764
P53151	0.1322	0	0	0	0	0	0	0	NA	0.1011
Q04276	0.2119	0	0.0424	0	0	0	0.0678	0	NA	0.2239
P53155	0.4199	1	0.3281	1	0.2992	1	0.2835	2	NA	0.3721
Q04203	0.2143	0	0	0	0	0	0	0	NA	0.1394
P53138	0.514	0	0	0	0	0	0.1963	0	NA	0.1736
Q02749	0.6587	3	0.4164	1	0.3276	2	0.3413	1	NA	0.7992
P53122	0.3913	2	0.0812	0	0	0	0.0696	0	NA	0.2058
Q3E7A7	0.3333	1	0.0808	0	0.3333	1	0.3535	1	NA	0.2599
P40895	0.3277	0	0.0168	0	0	0	0.1681	0	NA	0.2286
P53106	0.3021	1	0.0052	0	0	0	0.0729	0	NA	0.1291
Q08974	0.2332	0	0.0104	0	0	0	0.1503	0	NA	0.197
Q08620	0.3333	0	0	0	0	0	0.0909	0	NA	0.6944
P53186	0.5537	1	0.1818	0	0	0	0.3636	0	NA	0.4023
P0C5R1	0.1159	0	0	0	0	0	0	0	NA	0.1182
Q08872	0.1538	0	0	0	0	0	0.0342	0	NA	0.1339
Q08980	0.1275	0	0.0113	0	0	0	0	0	NA	0.1561
P0CX68	0.6432	2	0.5841	3	0.5386	3	0.5432	3	NA	0.9949
P0C5Q8	0.1458	0	0	0	0	0	0.125	0	NA	0.1724
A0A023PXP	0.2879	0	0	0	0	0	0.0682	0	NA	0.1741
Q04706	0.6432	2	0.5773	3	0.5364	3	0.5432	3	NA	0.9953
Q12110	0.3692	1	0.1542	0	0	0	0.1402	1	NA	0.3412
Q12506	0.1743	0	0	0	0	0	0	0	NA	0.1212
Q04214	0.4467	4	0.3333	4	0.3157	4	0.3105	4	NA	0.8596
Q03695	0.9073	2	0.6741	3	0.5783	3	0.5911	3	NA	0.9463
O13530	0.3025	1	0	0	0	0	0.0252	0	NA	0.1503
P0C2J4	0.6096	2	0.5434	3	0.4475	3	0.4384	2	NA	0.8758
Q04838	0.0098	0	0	0	0	0	0	0	NA	0.0952
P53071	0.2247	0	0	0	0	0	0.0393	0	NA	0.1919
Q06267	0.313	0	0.2	0	0.2783	0	0.2783	0	NA	0.4054
P0CX75	0.6432	2	0.5773	3	0.55	3	0.5591	3	NA	0.9956
P35995	0.3007	2	0.095	0	0	0	0.1064	0	NA	0.3416
Q03722	0.3946	2	0.2922	3	0.2605	2	0.2741	2	NA	0.3801
Q08419	0.3304	0	0.0609	0	0	0	0.1652	0	NA	0.2297
P43539	0.2647	0	0	0	0	0	0.0686	0	NA	0.1054
P43591	0.1728	0	0.0142	0	0	0	0.051	0	NA	0.1286
Q03884	0.0536	0	0	0	0	0	0	0	NA	0.0995
P53921	0.6174	1	0.3478	0	0	0	0.2261	0	NA	0.3116
Q03885	0.206	0	0	0	0	0	0.0704	0	NA	0.1632
P10356	0.228	1	0.1061	0	0	0	0.1061	0	NA	0.2358
P53964	0.1761	0	0.0035	0	0	0	0.0423	0	NA	0.2213
Q3E838	1	0	0.9286	0	1	0	1	0	NA	0.0248
P34225	0.4032	3	0.1339	1	0.1368	2	0.1441	2	NA	0.2985
P53310	0.0588	0	0	0	0	0	0.0588	0	NA	0.0939
P39977	0.2091	0	0	0	0	0	0.0818	0	NA	0.1036
P53232	0.6262	1	0.1028	0	0.1963	0	0.2991	0	NA	0.3723

Q3E824	0.7	1	0.4667	0	0.7111	1	0.6778	1	NA	0.9629
P47181	0.1676	0	0.0173	0	0	0	0	0	NA	0.1341
P53819	0.2873	3	0.1157	1	0.1205	1	0.142	1	NA	0.9123
Q03219	0.2299	0	0.0255	0	0	0	0	0	NA	0.1147
Q06328	0.3754	2	0.0568	0	0	0	0.142	0	NA	0.3595
P0CX21	0.2906	3	0.1153	1	0.1281	1	0.147	1	NA	0.8955
P28584	0.4432	3	0.261	1	0.2418	2	0.2396	2	NA	0.5422
P0CF17	0.194	1	0.0217	0	0	0	0.0516	0	NA	0.2012
P36096	0.1332	0	0.0079	0	0	0	0.0277	0	NA	0.2423
Q06696	0.3463	2	0.0636	0	0	0	0.0495	0	NA	0.3537
Q04272	0.6742	2	0.362	1	0.3258	1	0.2851	1	NA	0.3154
Q03433	0.6393	1	0.2464	0	0.2143	1	0.2643	1	NA	0.7806
P32842	0.0671	0	0	0	0	0	0	0	NA	0.1354
P53262	0.3509	1	0.0604	0	0	0	0.1132	0	NA	0.2309
P40157	0.3542	3	0.133	1	0.1381	2	0.156	2	NA	0.4838
Q03785	0.4902	2	0.0824	1	0.1106	1	0.1605	1	NA	0.8295
Q03631	0.5138	2	0.2754	4	0.2193	3	0.2214	2	TRU	0.7126
P47075	0.2067	1	0.0929	1	0	0	0.0804	0	NA	0.3775
P43585	0.4227	4	0.1087	1	0.1147	1	0.128	1	NA	0.6622
P38258	0.1024	0	0	0	0	0	0	0	NA	0.12
P32386	0.1969	4	0.0331	0	0	0	0.0572	0	NA	0.362
O13588	0.25	0	0	0	0	0	0	0	NA	0.185
Q12264	0.1321	0	0	0	0	0	0	0	NA	0.1115
Q03193	0.2161	1	0.0581	0	0	0	0.0935	0	NA	0.1276
Q12103	0.2272	2	0.0866	0	0	0	0.0835	0	NA	0.1985
Q03856	0.6432	2	0.5841	3	0.5386	3	0.5432	3	NA	0.9948
A0A023PZA	0.3276	1	0.0259	0	0	0	0.1121	0	NA	0.2847
P87287	0.0455	0	0	0	0	0	0	0	NA	0.138
P0C5L0	1	1	0.5769	0	0	0	0.5	0	NA	0.4622
P0C5M0	0.2889	0	0.0222	0	0	0	0.1778	0	NA	0.0248
P40103	0.1381	0	0.0042	0	0	0	0	0	NA	0.1371
Q3E7B0	0.4054	0	0	0	0	0	0.3243	0	NA	0.0248
Q8TGQ4	0.2581	0	0	0	0	0	0.2903	0	NA	0.0248
P38321	0.5433	4	0.3733	4	0.3578	5	0.3589	5	NA	0.9746
Q05497	0.3439	1	0.2014	1	0.164	0	0.1813	0	NA	0.3277
Q12217	0.6432	2	0.5841	3	0.5386	3	0.5432	3	NA	0.9947
P39557	0.0943	0	0	0	0	0	0	0	NA	0.0963
O94085	0.2787	0	0.0055	0	0	0	0.0874	0	NA	0.2042
P36067	0.0636	0	0	0	0	0	0	0	NA	0.1052
P34245	0.0662	0	0	0	0	0	0	0	NA	0.1672
Q3E810	0.0526	0	0	0	0	0	0	0	NA	0.0976
Q8TGR0	0.0667	0	0	0	0	0	0	0	NA	0.1033
Q03759	0.4286	0	0	0	0	0	0.1143	0	NA	0.1344
O13560	0.25	0	0	0	0	0	0	0	NA	0.1916
P39529	0.4024	2	0.1266	0	0	0	0.128	0	NA	0.4101
P47094	0.2707	1	0	0	0	0	0	0	NA	0.1685
Q04304	0.1322	0	0.0044	0	0	0	0.0308	0	NA	0.1126
Q3E841	0.1837	0	0.0408	0	0	0	0.1837	0	NA	0.1105
A0A023PZM	0.2263	0	0	0	0	0	0	0	NA	0.1458
Q06204	0.0831	0	0	0	0	0	0.0254	0	NA	0.1134
O13517	0.12	0	0	0	0	0	0	0	NA	0.0959
P0CE97	0.4561	0	0	0	0	0	0.0439	0	NA	0.1137

Q06107	0.2032	0	0.019	0	0	0	0.0762	0	NA	0.1248
P53842	0.4317	1	0	0	0	0	0.0935	0	NA	0.3706
P47090	0.1843	1	0.0725	0	0.1549	1	0.1275	1	NA	0.2386
Q06522	0.1184	0	0	0	0	0	0.0263	0	NA	0.1086
Q06116	0.2162	4	0.0414	0	0.0458	1	0.0627	1	NA	0.6824
Q04436	0.625	1	0	0	0	0	0.175	0	NA	0.5594
Q02872	0.5298	1	0.2976	1	0.2679	1	0.2976	1	NA	0.1437
P0C5R7	0.2361	0	0	0	0	0	0	0	NA	0.1371
O14468	1	1	0.8158	1	0.7368	1	0.6974	1	NA	0.9059
Q08953	0.0511	0	0	0	0	0	0.0292	0	NA	0.098
A0A023PYG	0.3457	1	0	0	0	0	0.0864	0	NA	0.4029
P42840	0.1408	0	0	0	0	0	0	0	NA	0.1641
Q06821	0.4693	3	0.1118	0	0	0	0.1776	1	NA	0.6931
Q08916	0.4286	1	0.1714	0	0	0	0.1333	0	NA	0.2974
Q08018	0.3571	1	0.1	0	0	0	0.15	0	NA	0.1759
P53339	0.3333	1	0	0	0	0	0	0	NA	0.1596
P53340	0.4658	0	0	0	0	0	0.0822	0	NA	0.1499
P0CY04	0.3256	0	0	0	0	0	0.3953	0	NA	0.0248
O13570	0.4652	1	0	0	0	0	0	0	NA	0.2591
P0CY03	0.3256	0	0	0	0	0	0.3953	0	NA	0.0248
P0C5R2	0.8659	1	0.4146	1	0.4146	0	0.561	0	NA	0.4841
Q07888	0.3588	2	0.1841	1	0.182	1	0.2082	1	NA	0.9025
P38887	0.2027	0	0.0415	0	0	0	0.0449	0	NA	0.2919
Q07688	0.2455	0	0	0	0	0	0.0479	0	NA	0.1081
P38726	0.0348	0	0	0	0	0	0	0	NA	0.1224
P40523	0.63	3	0.429	3	0.3732	3	0.3748	3	NA	0.9954
P38722	0.2177	0	0.0074	0	0	0	0.0443	0	NA	0.1227
A0A023PZF9	0.1402	0	0	0	0	0	0	0	NA	0.1877
O13534	0.8012	2	0.0062	0	0	0	0	0	NA	0.3498
P38864	0.3214	0	0.1339	0	0	0	0.1875	0	NA	0.2279
A0A023PXK	0.3732	1	0.0035	0	0	0	0.0986	0	NA	0.3832
P53980	0.2162	0	0.0248	0	0	0	0.0383	0	NA	0.1753
Q8TGS1	0.1061	0	0	0	0	0	0	0	NA	0.123
A0A023PZJ3	0.1875	0	0	0	0	0	0	0	NA	0.179
P53751	0.2778	3	0.0762	1	0.0833	1	0.1075	1	NA	0.5567
Q08956	0.1866	1	0.0586	0	0	0	0.0911	0	NA	0.2816
P38829	0.6364	2	0.2857	0	0.2597	1	0.2597	0	NA	0.7044
P09547	0.6005	5	0.4094	4	0.3729	4	0.3303	5	NA	0.9752
Q12026	0.7778	1	0.3333	0	0.5	0	0.463	0	NA	0.8514
Q12058	0.15	0	0	0	0	0	0	0	NA	0.1242
Q06146	0.9408	1	0.8879	2	0.8474	2	0.8349	1	NA	0.9969
Q03649	0.1804	0	0.0535	0	0	0	0.0579	0	NA	0.1529
P0CX91	0.1194	0	0	0	0	0	0	0	NA	0.1814
P40095	0.9075	4	0.726	5	0.6702	6	0.6771	6	NA	0.9989
Q7LHG5	0.2744	2	0.1455	1	0.1068	1	0.1188	1	NA	0.3711
P40455	0.6298	1	0.5447	1	0.5489	1	0.5447	1	NA	0.9859
P38841	0.1842	0	0	0	0	0	0.0614	0	NA	0.1192
Q3E763	0.4697	0	0.0909	0	0	0	0.2879	0	NA	0.1177
Q12351	0.3504	0	0	0	0	0	0.1095	0	NA	0.1614
P38898	0.8562	1	0.4967	1	0.5033	1	0.5098	1	NA	0.9633
P53719	1	1	0.5142	1	0.6651	2	0.7123	2	NA	0.9956
Q8TGL6	0.1594	0	0	0	0	0	0	0	NA	0.0897

P40052	0.9	2	0.819	3	0.7714	2	0.7238	3	NA	0.9847
Q3E740	0.1299	0	0	0	0	0	0.1169	0	NA	0.0999
Q12512	0.1245	0	0.004	0	0	0	0	0	NA	0.1128
P40517	0.7309	1	0.5596	2	0.6086	2	0.63	2	NA	0.9955
Q12138	0.7868	1	0.625	2	0.6912	1	0.6765	1	NA	0.916
P34163	0.2774	1	0.1551	1	0.1551	1	0.1624	2	NA	0.2177
P53840	0.3788	5	0.1212	2	0.13	2	0.1535	2	NA	0.3468
P40310	0.2258	2	0.0767	1	0.1129	1	0.1216	1	NA	0.2795
Q12463	0.1963	0	0.0046	0	0	0	0.0323	0	NA	0.1343
P38128	0.7412	3	0.5929	2	0.4447	1	0.3805	1	TRU	0.9727
P32572	0.4033	1	0.0733	0	0	0	0.05	0	NA	0.2565
Q12379	0.4647	1	0.4647	1	0.4824	1	0.4824	1	NA	0.9465
P32944	0.6545	2	0.4579	5	0.3834	4	0.3858	3	NA	0.9909
P39706	0.216	1	0.054	0	0	0	0.0469	0	NA	0.1337
P32584	0.1423	0	0.0042	0	0	0	0.0544	0	NA	0.1427
P13188	0.3486	4	0.0902	1	0.0766	1	0.0989	1	NA	0.2377
P54003	0.3444	1	0.1457	0	0	0	0.1391	0	NA	0.3107
Q07921	0.125	0	0	0	0	0	0.0375	0	NA	0.0969
P53882	0.8726	2	0.3365	1	0.2814	2	0.5346	5	NA	1
P41835	0.1407	0	0.0167	0	0	0	0.0574	0	NA	0.1577
P33891	0.2254	2	0.02	0	0	0	0.0328	0	NA	0.2091
P00927	0.1545	1	0.0069	0	0	0	0.0069	0	NA	0.1722
Q03290	0.4393	1	0.0312	0	0	0	0.028	0	NA	0.1724
Q08975	0.0708	0	0.0018	0	0	0	0.0272	0	NA	0.1993
Q12004	0.3107	0	0.0207	0	0	0	0.0947	0	NA	0.2538
P53099	0.1623	1	0.0363	0	0	0	0.0397	0	NA	0.1141
P42943	0.1582	0	0.0436	0	0.0545	0	0.0564	0	NA	0.1651
P02557	0.2123	1	0.105	1	0.0941	1	0.0963	1	NA	0.2412
P07236	0.2381	1	0.0195	0	0	0	0.0281	0	NA	0.1765
P23255	0.3362	5	0.108	1	0.086	1	0.1038	1	NA	0.3234
P40040	0.9266	1	0.9495	2	0.9817	2	0.8349	2	NA	0.9974
P41814	0.3787	1	0.1151	0	0.1297	0	0.1255	0	NA	0.2153
P48232	0.3235	0	0.0588	0	0	0	0.2451	0	NA	0.1902
P43554	0.4318	3	0.2087	0	0	0	0.1156	0	NA	0.3821
P38967	0.1672	1	0.0405	0	0.0811	0	0.0828	0	NA	0.2227
Q06836	0.6142	4	0.3728	4	0.3483	4	0.3564	4	NA	0.9986
P40340	0.546	7	0.4307	5	0.3358	5	0.3452	6	NA	0.8354
P53378	0.3975	3	0.0951	0	0	0	0.0973	0	NA	0.3069
Q03750	0.8	2	0.5235	2	0.3941	1	0.3294	1	NA	0.8491
P32830	0.2569	0	0	0	0	0	0.1468	0	NA	0.1784
P54837	0.3507	1	0.0142	0	0	0	0.0379	0	NA	0.1563
Q12000	0.7652	4	0.4377	1	0.3942	2	0.3913	2	NA	0.9238
P25576	0.1395	0	0	0	0	0	0.0271	0	NA	0.128
P35842	0.1135	0	0.0043	0	0	0	0.0107	0	NA	0.1242
P40352	0.5051	4	0.27	1	0.2046	2	0.2194	1	NA	0.8157
P21651	0.9809	2	0.5541	1	0.3981	1	0.4045	1	NA	0.6702
Q12192	0.771	1	0.5935	1	0.5806	1	0.5548	2	NA	0.9951
P06780	0.3301	1	0.1388	0	0	0	0.2344	0	NA	0.2498
P21524	0.2072	1	0.0743	1	0.0743	1	0.08	1	NA	0.3371
P16664	0.3997	3	0.0784	0	0.0709	1	0.1026	1	NA	0.3499
P40188	0.622	2	0.1951	0	0	0	0.1524	0	NA	0.297
P38339	0.503	2	0.2838	1	0.2583	1	0.2643	1	NA	0.7666

P20084	0.3488	1	0.0698	0	0.2326	0	0.2907	0	NA	0.2389
P31334	0.3197	1	0.1375	1	0	0	0.145	0	NA	0.3077
P36159	0.185	1	0.0215	0	0	0	0.0513	0	NA	0.1168
P32561	0.1663	1	0.0808	1	0.1178	1	0.1201	1	NA	0.1991
P38711	0.4024	0	0.0488	0	0	0	0.2317	0	NA	0.1198
P39938	0.605	2	0.4538	0	0.479	1	0.479	1	NA	0.7486
O13516	0.4822	2	0.2538	0	0.2741	1	0.2893	1	NA	0.1793
Q03124	0.2358	2	0.0482	0	0.0551	0	0.0568	0	NA	0.2322
Q07794	0.2638	1	0.0459	0	0	0	0.0803	0	NA	0.1693
P53036	0.3191	3	0.132	0	0.1259	1	0.1259	1	NA	0.5602
P38990	0.7531	3	0.5867	5	0.5298	6	0.521	8	NA	0.9992
Q04002	0.1755	2	0.0335	0	0	0	0.0315	0	NA	0.2571
P15179	0.2508	1	0.0137	0	0	0	0.0334	0	NA	0.1524
Q08553	0.3511	0	0.1011	0	0	0	0.1436	0	NA	0.2009
P38198	0.4627	3	0.2062	3	0.1712	3	0.189	3	NA	0.9113
Q12383	0.437	2	0.105	0	0	0	0.1345	0	NA	0.3736
P31688	0.2411	3	0.0781	0	0	0	0.0848	0	NA	0.2292
P08525	0.2979	0	0	0	0	0	0.1489	0	NA	0.1991
Q12090	0.198	1	0.0099	0	0	0	0.0421	0	NA	0.1956
Q12367	0.2561	1	0.0381	0	0	0	0.0899	0	NA	0.1885
P36144	0.1971	0	0.0109	0	0	0	0.0657	0	NA	0.1332
P05744	0.2804	0	0.0187	0	0	0	0.0561	0	NA	0.1404
P53875	0.3924	0	0.0823	0	0	0	0.1582	0	NA	0.5348
P08518	0.2279	3	0.0923	1	0	0	0.0711	0	NA	0.4044
Q07829	0.1062	0	0	0	0	0	0	0	NA	0.1105
P41057	0.3393	0	0	0	0	0	0.4107	0	NA	0.5768
P32902	0.5482	2	0.2589	0	0	0	0.1904	0	NA	0.4591
P53324	0.1344	0	0	0	0	0	0	0	NA	0.1331
Q00916	0.53	2	0.35	2	0.39	2	0.4833	2	NA	0.9676
Q08199	0.3017	1	0.0618	0	0	0	0.0926	0	NA	0.2454
Q06132	0.5573	3	0.3126	3	0.3181	2	0.3259	2	NA	0.6138
P15303	0.2057	1	0.0443	0	0	0	0.0404	0	NA	0.2602
P25567	0.7235	2	0.7097	2	0.6889	2	0.6774	2	NA	0.9975
Q04951	0.4422	1	0.2519	1	0.1671	1	0.2237	1	NA	0.4539
P32916	0.351	1	0.1916	1	0.1498	1	0.1723	1	NA	0.3454
Q07549	0.4214	1	0.3214	0	0.4571	1	0.4143	1	NA	0.6088
P25357	0.8271	5	0.7007	6	0.5742	4	0.5677	5	NA	0.999
P32568	0.2019	4	0.056	1	0.0626	1	0.072	1	NA	0.459
P48526	0.1507	0	0.003	0	0	0	0.008	0	NA	0.1282
Q07395	0.2145	1	0.0613	1	0.0694	1	0.0839	1	NA	0.163
P08425	0.2026	1	0.0235	0	0	0	0.0277	0	NA	0.167
P53738	0.1852	0	0.0222	0	0	0	0	0	NA	0.1179
P32600	0.1742	3	0.0453	1	0.0376	1	0.0428	1	NA	0.3221
P38427	0.378	2	0.275	4	0.2413	5	0.2341	5	NA	0.5262
P36166	0.7861	3	0.6388	3	0.5779	2	0.5694	2	NA	0.9921
P40048	0.4795	5	0.195	2	0.1778	2	0.2241	2	NA	0.8285
Q06991	0.1255	0	0	0	0	0	0	0	NA	0.1124
P12753	0.6593	5	0.0907	0	0	0	0.0252	0	NA	0.2149
P14737	0.6478	3	0.5355	7	0.3568	4	0.3468	4	NA	0.9788
P12689	0.3391	3	0.0904	0	0	0	0.0538	0	NA	0.247
P25378	0.2392	0	0	0	0	0	0.0957	0	NA	0.1877
Q12362	0.2166	1	0.0981	0	0	0	0.0846	0	NA	0.302

P38629	0.1471	0	0.0294	0	0	0	0.0529	0	NA	0.1186
Q06208	0.3494	2	0	0	0	0	0.0734	0	NA	0.1923
P26785	0.2929	1	0	0	0	0	0.1212	0	NA	0.2734
P23369	0.7516	2	0.0892	0	0	0	0.1019	0	NA	0.3126
P36531	0.7458	2	0.4294	0	0.3503	1	0.4294	1	NA	0.3333
P10622	0.4623	1	0.2264	0	0.4245	1	0.434	1	NA	0.5615
Q12158	0.659	2	0.5018	2	0.3269	2	0.318	2	NA	0.7622
P36124	0.6112	3	0.3848	3	0.4261	4	0.4048	4	NA	0.9802
P34164	0.612	2	0.5783	2	0.5277	2	0.5373	3	NA	0.9707
Q04401	0.4662	1	0.1805	0	0	0	0.203	0	NA	0.2712
P40548	0.6541	2	0.1824	0	0.2327	0	0.2893	0	NA	0.6177
P15705	0.5297	4	0.3786	1	0.2733	1	0.2513	1	NA	0.7192
Q04748	0.2494	2	0.02	0	0	0	0.0267	0	NA	0.2357
P40413	0.2811	2	0.0605	0	0	0	0.0391	0	NA	0.1598
Q04226	0.4942	2	0.1936	0	0	0	0.1994	0	NA	0.4384
Q12297	0.5807	2	0.4023	2	0.3201	1	0.2975	1	NA	0.6844
P47045	0.477	3	0.2238	0	0	0	0.2134	0	NA	0.4953
P32776	0.5483	3	0.2508	2	0.1931	1	0.2103	1	NA	0.8761
P15565	0.2947	2	0.1509	1	0.1158	1	0.1298	1	NA	0.248
Q08760	0.269	0	0.0782	0	0	0	0.1517	0	NA	0.5707
Q05131	0.1475	0	0.0207	0	0	0	0.0737	0	NA	0.11
P05748	0.6176	2	0.3088	0	0	0	0.2794	0	NA	0.3933
P43602	0.5866	4	0.3233	2	0.2524	2	0.3479	4	NA	0.9881
P25443	0.2835	1	0.1772	1	0.1654	1	0.1732	1	NA	0.3059
P0C0X0	0.4627	0	0.0299	0	0	0	0.194	0	NA	0.2498
P0CX31	0.4	1	0.1481	0	0	0	0.3111	1	NA	0.1708
P43609	0.5619	2	0.1921	1	0.2011	2	0.2154	2	NA	0.4822
P38850	0.4131	1	0.2776	3	0.2093	3	0.2439	2	NA	0.3901
P53064	0.7867	2	0.5896	2	0.543	1	0.5412	2	NA	0.9671
P46954	0.4234	2	0.0591	0	0.0579	0	0.082	0	TRU	0.39
Q12216	0.5372	5	0.4463	4	0.3581	2	0.3375	3	NA	0.847
P11075	0.3738	6	0.2245	2	0.212	3	0.2145	3	NA	0.6266
Q6Q595	0.2286	0	0.0743	0	0	0	0.1543	0	NA	0.4221
P32828	0.7205	4	0.462	4	0.2569	3	0.2859	2	NA	0.7083
P38956	0.6686	1	0.4024	1	0.1834	1	0.1716	0	NA	0.4443
P38343	0.5333	1	0	0	0	0	0.0533	0	NA	0.1554
P38925	0.2348	2	0.0487	0	0	0	0.0904	0	NA	0.278
Q06466	0.2163	0	0.0278	0	0	0	0.0685	0	NA	0.3439
P53916	0.1244	0	0.0023	0	0	0	0.0207	0	NA	0.1142
P18412	0.6296	4	0.321	1	0.2634	1	0.3004	1	TRU	0.903
P46670	0.4515	1	0.0112	0	0	0	0.0224	0	NA	0.2089
P33315	0.2673	2	0.0558	0	0	0	0.0558	0	NA	0.1818
Q08693	0.3078	4	0.1372	1	0.1261	1	0.1384	1	NA	0.4696
Q06266	0.6176	2	0.3701	2	0.3436	3	0.3947	2	NA	0.6386
P46944	0.2679	3	0.0831	0	0.1046	1	0.1461	1	NA	0.3598
P36082	0.1575	0	0	0	0	0	0	0	NA	0.1176
P07256	0.3085	1	0.0284	0	0	0	0.0306	0	NA	0.2045
Q12189	0.1705	0	0.0194	0	0	0	0.0969	0	NA	0.1604
Q08774	0.1901	0	0	0	0	0	0.0455	0	NA	0.119
P53733	0.1758	0	0.1978	0	0	0	0.2088	0	NA	0.1469
O14464	0.3226	0	0.0323	0	0	0	0.1398	0	NA	0.1141
P40357	0.9969	2	0.914	3	0.7327	2	0.6805	2	NA	0.9821

Q07657	0.5681	1	0.3648	2	0.3376	2	0.3085	2	NA	0.8559
P38804	0.5946	1	0.2162	0	0	0	0.3514	0	NA	0.2873
P40167	0.594	1	0.4094	1	0.4295	2	0.4463	2	NA	0.6376
P30619	0.2624	2	0.1533	1	0.1354	1	0.134	1	NA	0.2187
P42843	0.3211	3	0.0537	0	0	0	0.0708	0	NA	0.246
P13130	0.4387	1	0.0184	0	0	0	0.0736	0	NA	0.787
P53873	0.1737	0	0.0224	0	0	0	0.0336	0	NA	0.1281
P39933	0.5221	3	0.2145	0	0.1981	1	0.2075	1	NA	0.557
P40071	0.1232	0	0.0085	0	0	0	0.0255	0	NA	0.1707
P35202	0.1818	1	0.0031	0	0	0	0.0251	0	NA	0.1106
Q08224	0.0944	0	0	0	0	0	0.02	0	NA	0.196
P33753	0.3615	2	0.1111	1	0.1002	1	0.1189	1	NA	0.4715
P32473	0.1776	1	0	0	0	0	0.0191	0	NA	0.1148
P53397	0.2048	0	0	0	0	0	0.0399	0	NA	0.1417
Q05518	0.9198	3	0.9519	4	0.7776	2	0.7856	2	NA	0.9991
P53844	0.26	0	0.0743	0	0	0	0.0971	0	NA	0.3351
P47180	0.2742	1	0.0111	0	0	0	0.0609	0	NA	0.5861
P42836	0.1726	1	0	0	0	0	0.0327	0	NA	0.1216
P32383	0.2716	3	0.0633	0	0.0794	1	0.1139	1	NA	0.361
Q02771	0.7477	1	0.6729	1	0	0	0.6729	0	NA	0.3669
P34230	0.3072	2	0.1477	1	0.1618	2	0.1852	2	NA	0.4878
P32522	0.1275	1	0.0041	0	0	0	0.0249	0	NA	0.1489
P14736	0.3966	2	0.2361	2	0.2281	1	0.244	2	NA	0.4475
P0C0W9	0.4138	1	0.1149	0	0	0	0.1494	0	NA	0.1526
Q06090	0.35	1	0	0	0	0	0.05	0	NA	0.1407
P02381	0.7638	4	0.0879	0	0	0	0.0452	0	NA	0.4121
Q03441	0.3628	1	0.2814	1	0.2581	1	0.2628	1	NA	0.2686
P53942	0.2997	1	0.0326	0	0	0	0.0814	0	NA	0.3599
P39677	0.2613	2	0.072	0	0	0	0.0537	0	NA	0.1966
P25359	0.1497	0	0.0254	0	0	0	0.0482	0	NA	0.1312
P38429	0.6667	1	0.6418	1	0.4776	1	0.5075	1	NA	0.7574
P33759	0.228	1	0.101	0	0	0	0.1173	0	NA	0.253
P17883	0.169	1	0.044	1	0.0426	1	0.0461	1	NA	0.167
P40317	0.4939	2	0.3896	3	0.3196	2	0.3219	3	NA	0.8651
P0C0W1	0.1692	0	0	0	0	0	0.0385	0	NA	0.106
P32608	0.2347	1	0.0119	0	0	0	0.034	0	NA	0.1464
P42948	0.6107	3	0.3268	3	0.2625	2	0.3	2	NA	0.8353
Q12118	0.685	2	0.4913	2	0.4335	2	0.3353	1	NA	0.9262
P40210	0.6605	4	0.499	2	0.411	2	0.4438	2	NA	0.9573
Q06168	0.3638	2	0.2465	1	0	0	0.0915	0	NA	0.3048
P47008	0.1361	0	0.0034	0	0	0	0.0408	0	NA	0.1183
P24280	0.3026	1	0.0757	0	0	0	0.1579	0	NA	0.1808
Q04969	0.3146	1	0.0169	0	0	0	0.073	0	NA	0.1383
P08458	0.3673	1	0.1143	0	0	0	0.1184	0	NA	0.2599
Q12306	0.6832	1	0.505	0	0	0	0.396	0	NA	0.4435
Q6WNK7	0.4375	0	0.0208	0	0	0	0.1354	0	NA	0.1074
P46676	0.7919	4	0.6817	6	0.5424	6	0.5085	6	TRU	0.998
P47030	0.755	3	0.7053	4	0.6424	6	0.6093	5	NA	0.997
Q03860	0.2098	0	0	0	0	0	0.1818	0	NA	0.1761
P36026	0.3752	2	0.1199	0	0.159	2	0.1967	2	NA	0.627
P39944	0.328	2	0.1304	1	0	0	0.113	1	NA	0.3246
Q12071	0.3667	3	0.1485	1	0.1271	1	0.1564	1	NA	0.54

P40041	0.6871	3	0.3426	2	0.3366	2	0.3426	2	NA	0.763
Q12498	0.5246	1	0.1056	0	0	0	0.1549	0	NA	0.3027
P40476	0.8239	3	0.3836	1	0.3742	1	0.4748	1	NA	0.9898
P41903	0.255	1	0.0774	0	0	0	0.1003	0	NA	0.2182
P08456	0.3768	1	0.1304	0	0.1558	1	0.1703	0	NA	0.3204
P07259	0.1454	2	0.0158	0	0	0	0.0117	0	NA	0.1439
P32387	0.4335	2	0.1065	0	0	0	0.1179	0	NA	0.352
P20435	0.5742	1	0.5355	1	0.5355	1	0.4903	1	NA	0.487
Q3E7Y3	0.1923	0	0	0	0	0	0.0308	0	NA	0.1053
P05750	0.2333	1	0.1333	0	0.1667	0	0.1875	0	NA	0.1587
P06105	0.4812	5	0.2062	1	0.1203	2	0.1391	1	NA	0.6517
P53732	0.6536	1	0.3987	0	0	0	0.3007	0	NA	0.2756
P20606	0.0789	0	0	0	0	0	0	0	NA	0.1205
Q01163	0.2667	1	0.0333	0	0	0	0.0956	1	NA	0.2315
P38165	0.8704	2	0.7181	4	0.5206	2	0.4444	2	TRU	0.9982
P40962	0.6379	1	0.431	0	0.3448	1	0.3707	0	NA	0.5192
P32578	0.7215	3	0.5865	8	0.4847	5	0.4908	5	NA	0.9969
Q04675	0.3438	1	0.0781	0	0	0	0.2344	0	NA	0.1519
Q06058	0.1263	0	0.0175	0	0	0	0.0702	0	NA	0.1428
P21825	0.3467	1	0.0839	0	0	0	0.1496	0	NA	0.2556
Q04658	0.1924	0	0.0542	0	0.0596	0	0.0678	0	NA	0.2075
Q3E752	1	1	0.1493	0	0	0	0.6866	0	NA	0.922
P33336	0.4565	1	0.3346	2	0.3256	3	0.3256	3	NA	0.9551
P40150	0.3768	2	0.0212	0	0	0	0.0163	0	NA	0.1621
P53394	0.2759	2	0.13	1	0.1472	2	0.1737	1	NA	0.3968
P15180	0.335	1	0.2064	1	0.1844	1	0.181	1	NA	0.2603
Q05506	0.145	0	0.0198	0	0	0	0.028	0	NA	0.1354
P41895	0.8	4	0.7483	5	0.6435	5	0.6204	5	NA	0.9988
Q07381	0.3921	3	0.231	1	0.1827	2	0.1954	1	NA	0.2451
Q04120	0.1837	0	0.0051	0	0	0	0.0765	0	NA	0.1368
P00937	0.1426	0	0.0103	0	0	0	0.0351	0	NA	0.1411
P28263	0.5367	1	0.3761	1	0.3853	1	0.3853	1	NA	0.8141
P32837	0.1208	0	0.007	0	0	0	0.0263	0	NA	0.2144
P38759	0.4007	1	0.344	1	0.3404	1	0.3546	1	NA	0.8647
P53142	0.0864	0	0	0	0	0	0.0247	0	NA	0.1217
P53853	0.5	2	0.2235	1	0.2917	1	0.3106	1	NA	0.8314
P54780	0.6176	2	0.3137	0	0	0	0.2892	0	NA	0.4897
P36105	0.1739	0	0	0	0	0	0.0725	0	NA	0.1333
P0CX82	0.5661	2	0.3757	1	0.3915	1	0.381	1	NA	0.7658
P04456	0.3662	1	0.0141	0	0	0	0.0775	0	NA	0.1296
P04650	1	1	0.9412	1	0	0	0.8039	0	NA	0.7067
P36527	0.8367	2	0.0816	0	0	0	0.1088	0	NA	0.206
P19956	0.449	1	0.0306	0	0	0	0.0612	0	NA	0.1537
P32910	0.2808	1	0.0315	0	0	0	0.0662	0	NA	0.1912
P38255	0.5465	3	0.3372	2	0.2349	1	0.2395	1	NA	0.6361
P39735	0.1264	0	0.0536	0	0	0	0.0728	0	NA	0.1639
P14693	0.2006	0	0.0061	0	0	0	0	0	NA	0.1815
P32607	0.6215	2	0.5932	1	0.5367	2	0.5254	2	TRU	0.7564
P38166	0.3023	1	0.0744	0	0.1488	1	0.1488	1	NA	0.2223
P40363	0.1672	0	0.0234	0	0	0	0.0334	0	NA	0.1837
P38968	0.5679	3	0.4242	2	0.348	1	0.3543	3	NA	0.9739
Q02651	0.5429	2	0.1061	0	0	0	0.1102	0	NA	0.2182

P53207	0.6903	2	0.4177	2	0.3242	1	0.3548	2	NA	0.8156
P32380	0.8919	1	0.4555	6	0.214	2	0.2267	1	NA	0.5654
P53824	0.2315	0	0.0101	0	0	0	0	0	NA	0.1226
P12954	0.471	4	0.2768	2	0.2215	4	0.2325	3	NA	0.7236
Q12232	0.54	3	0.1993	2	0.1772	1	0.1823	1	NA	0.4988
P35207	0.2883	2	0.1958	4	0.1546	4	0.1515	4	NA	0.5937
P25302	0.7164	3	0.5682	6	0.473	6	0.4437	7	TRU	0.9862
P08153	0.8082	3	0.5994	5	0.3413	3	0.3794	3	TRU	0.9566
P40468	0.2795	4	0.1044	1	0.0825	2	0.0905	1	NA	0.6989
Q99190	0.229	1	0	0	0	0	0.029	0	NA	0.1107
P36101	0.2953	1	0.0582	0	0	0	0.0828	0	NA	0.3456
Q04083	0.2108	1	0.0027	0	0	0	0.0568	0	NA	0.1443
P50105	0.8454	3	0.6082	2	0.4253	1	0.451	1	NA	0.9133
P42883	0.1382	0	0	0	0	0	0	0	NA	0.1192
P42949	0.5705	1	0.3557	1	0.3557	1	0.4094	1	NA	0.9371
P33890	0.6494	1	0.2191	0	0	0	0.3745	1	NA	0.9891
P32773	0.6189	1	0.6608	1	0.4091	2	0.458	3	NA	0.8719
P23254	0.1971	0	0.0382	0	0	0	0.0515	0	NA	0.3429
Q12239	0.0936	0	0	0	0	0	0.0234	0	NA	0.1146
Q07824	0.227	2	0.1553	1	0.1143	1	0.1331	1	NA	0.1895
P07273	0.4531	1	0.3236	1	0.2362	1	0.2524	1	NA	0.5903
Q07878	0.292	11	0.0792	1	0.0674	1	0.0709	1	NA	0.8816
P41834	0.6156	2	0.078	0	0	0	0.0231	0	NA	0.2215
Q99222	0.2396	2	0.0829	0	0.0806	1	0.0963	1	NA	0.3487
Q07732	0.7139	3	0.3316	1	0.2076	1	0.2203	1	NA	0.5477
P38628	0.0934	0	0.0018	0	0	0	0.0126	0	NA	0.1301
Q99186	0.2118	1	0.0326	0	0	0	0.0998	0	NA	0.3155
Q06497	0.1707	0	0.0244	0	0	0	0.0732	0	NA	0.1715
P38182	0.3761	1	0	0	0	0	0.1624	0	NA	0.1248
Q12165	0.4437	1	0	0	0	0	0.05	0	NA	0.1262
Q08754	0.2091	1	0.0389	0	0	0	0.0349	0	NA	0.1913
Q05949	0.3013	2	0.0658	0	0.1443	1	0.1418	1	NA	0.2634
P38308	0.5931	4	0.3149	2	0.3106	4	0.3528	3	NA	0.9961
P04037	0.3871	1	0.1806	0	0	0	0.2258	0	NA	0.4697
P53314	0.1799	0	0.0418	0	0	0	0.0837	0	NA	0.1756
P29056	0.5352	4	0.3238	2	0.2081	1	0.2198	1	NA	0.3147
P38756	0.1212	0	0	0	0	0	0.0186	0	NA	0.1515
Q02457	0.4021	2	0.3327	2	0.2776	2	0.2687	2	NA	0.8108
Q12116	0.1734	0	0.0233	0	0	0	0.0338	0	NA	0.3848
P53322	0.2191	1	0.1292	1	0.1292	1	0.1255	1	NA	0.166
Q02721	0.0909	0	0	0	0	0	0	0	NA	0.1158
P53147	0.6522	2	0.471	2	0	0	0.2935	1	TRU	0.7059
P35127	0.3347	1	0.0551	0	0	0	0.1314	0	NA	0.2816
P34241	0.1553	2	0.0187	0	0.0204	0	0.0306	0	NA	0.1689
Q99344	0.0435	0	0	0	0	0	0	0	NA	0.0964
P38959	0.248	2	0.1391	1	0.1472	1	0.1603	1	NA	0.5433
P53076	0.4697	4	0.309	3	0.2871	3	0.3017	4	NA	0.8722
P47118	0.4326	1	0.0496	0	0	0	0.2199	0	NA	0.1968
Q12195	0.3038	0	0	0	0	0	0.2785	0	NA	0.2199
Q07509	0.1302	0	0	0	0	0	0.0592	0	NA	0.1778
Q12392	0.6096	2	0.5434	3	0.4635	3	0.4498	2	NA	0.9087
Q03935	0.9426	2	0.7755	2	0.6397	2	0.6005	2	TRU	0.9947

Q03483	0.6142	2	0.5502	3	0.4703	3	0.4726	3	NA	0.8905
P87274	0.2949	0	0	0	0	0	0.1218	0	NA	0.205
P25650	0.0171	0	0	0	0	0	0	0	NA	0.0987
Q3E778	0.1765	0	0	0	0	0	0.1471	0	NA	0.0248
A0A023PXD	0.1103	0	0	0	0	0	0.0294	0	NA	0.1041
O13542	0.2478	0	0	0	0	0	0.0442	0	NA	0.1228
Q8TGM4	0.25	0	0	0	0	0	0.2727	0	NA	0.0248
P36114	0.3214	3	0.0524	0	0	0	0.0579	0	NA	0.2865
P54007	0.1037	0	0	0	0	0	0.0133	0	NA	0.1303
Q12492	0.1079	0	0	0	0	0	0	0	NA	0.1167
P0CL28	0.7749	1	0.3403	1	0.3822	1	0.466	1	NA	0.9992
Q12079	0.1949	0	0.0614	0	0	0	0.1155	0	NA	0.2631
P53116	0.4059	0	0	0	0	0	0	0	NA	0.1362
P46992	0.2626	0	0	0	0	0	0.0328	0	NA	0.242
Q3E7Y7	0.1429	0	0	0	0	0	0.1429	0	NA	0.1027
Q06594	0.2569	0	0.1284	0	0	0	0.1835	0	NA	0.2066
P47091	0.125	0	0	0	0	0	0	0	NA	0.1813
P47092	0.2091	0	0	0	0	0	0.0182	0	NA	0.1397
Q12112	0.4484	4	0.3356	4	0.3066	4	0.3071	5	NA	0.8986
Q92392	0.6432	2	0.5773	3	0.5477	3	0.5568	3	NA	0.9947
P36089	0.068	0	0	0	0	0	0	0	NA	0.1013
Q08922	0.4488	1	0.2126	0	0.2835	1	0.2835	1	NA	0.4185
Q12012	0.2311	0	0	0	0	0	0.0398	0	NA	0.1431
O13566	0.1806	0	0	0	0	0	0.0417	0	NA	0.2151
Q8TGL0	0.0864	0	0	0	0	0	0.0617	0	NA	0.1294
Q8TGL2	0.0606	0	0	0	0	0	0	0	NA	0.0961
P33199	0.5769	1	0.0769	0	0	0	0.2231	0	NA	0.31
A0A023PXH	0.0787	0	0	0	0	0	0	0	NA	0.1052
Q03559	0.6091	2	0.5888	0	0.5178	2	0.4365	2	NA	0.8309
A0A023PXQ	0.868	1	0.0051	0	0	0	0.0279	0	NA	0.285
P38087	0.2158	1	0.1003	0	0.0942	0	0.1246	0	NA	0.1772
P0CE98	0.4561	0	0	0	0	0	0.0439	0	NA	0.1137
Q03703	0.7912	1	0.6206	1	0.6618	1	0.6471	1	NA	0.9722
Q3E813	0.2245	0	0	0	0	0	0.2245	0	NA	0.121
A0A023PYJ	0.1466	0	0	0	0	0	0	0	NA	0.1864
P38555	0.3767	1	0.2108	1	0.2063	1	0.2063	1	NA	0.1918
P38831	0.0594	0	0	0	0	0	0	0	NA	0.0987
P38842	0.1883	0	0	0	0	0	0	0	NA	0.152
P38690	0.1678	0	0.0591	0	0	0	0.0733	0	NA	0.2856
P39959	0.8465	2	0.3747	1	0.2641	1	0.2709	1	TRU	0.7997
P0C2I4	0.6046	2	0.523	3	0.4979	3	0.5	3	NA	0.9927
A0A023PZE	1	1	0.1942	0	0.7184	1	0.7087	1	NA	0.999
P53748	0.159	0	0.0214	0	0	0	0.0459	0	NA	0.1625
Q6Q5P6	0.3535	2	0.1404	0	0.109	0	0.1453	1	NA	0.1294
P53729	0.2051	0	0.0256	0	0	0	0.0466	0	NA	0.2032
P53723	0.2426	1	0.0916	1	0.1064	1	0.1163	1	NA	0.2362
P40445	0.2269	1	0	0	0	0	0.0387	0	NA	0.142
P0CF21	0.5276	1	0.0079	0	0	0	0.0787	0	NA	0.2041
P53717	0.306	0	0	0	0	0	0	0	NA	0.2128
Q99219	0.3586	1	0.169	1	0	0	0.1552	0	NA	0.2609
Q07804	0.2304	1	0.0419	0	0	0	0.0838	0	NA	0.3205
P0C5Q9	0.5455	0	0.1212	0	0	0	0.5152	0	NA	0.0248

P25637	0.3217	1	0.1395	0	0	0	0.1744	0	NA	0.2946
P43580	0.5811	1	0.2027	0	0	0	0.1824	0	NA	0.2787
Q12485	0.6432	2	0.5841	3	0.5477	3	0.5545	3	NA	0.9962
P40093	0.2396	0	0.003	0	0	0	0.0266	0	NA	0.1783
P53876	0.4167	1	0.1759	0	0.3241	1	0.3704	1	NA	0.2181
P0C5N1	0.2642	0	0	0	0	0	0.1509	0	NA	0.2172
P53243	0.2998	3	0.0336	0	0	0	0.0473	0	NA	0.3957
P0C5N2	0.2857	0	0	0	0	0	0.2619	0	NA	0.0248
P35723	0.4078	1	0.199	0	0.2379	0	0.2282	0	NA	0.4997
P47006	0.8283	3	0.3219	1	0.3391	1	0.3605	1	NA	0.6692
Q12481	1	1	0.67	2	0.7467	1	0.7433	1	NA	0.9398
P26786	0.4105	1	0.0737	0	0	0	0.1263	0	NA	0.1518
P39940	0.4116	1	0.3597	1	0.2608	2	0.2806	2	NA	0.788
Q03919	0.4156	0	0	0	0	0	0.1039	0	NA	0.1397
P28707	0.5417	1	0.4444	0	0.4444	1	0.4722	1	NA	0.8755
Q04053	0.5568	3	0.1888	2	0.2064	1	0.2128	1	NA	0.8375
Q06245	0.2227	2	0.0299	0	0	0	0.0666	0	NA	0.1865
P38789	0.6137	2	0.426	2	0.3753	2	0.3687	2	NA	0.8216
Q12427	0.8752	3	0.7329	4	0.729	4	0.7018	5	NA	0.9996
P40825	0.2869	4	0.0214	0	0	0	0.0356	0	NA	0.2711
Q12109	0.2894	1	0.0671	0	0	0	0.0833	0	NA	0.1978
Q03533	0.5	2	0.2747	2	0.1962	1	0.2406	1	NA	0.875
P36142	0.1268	0	0.0335	0	0	0	0.055	0	NA	0.1311
Q08919	0.2095	1	0.0664	0	0.0613	1	0.0485	0	NA	0.2338
P06786	0.3473	3	0.1982	3	0.2038	3	0.2024	3	NA	0.6786
P19812	0.2179	5	0.0872	2	0.0764	2	0.0785	2	NA	0.3777
P40362	0.4377	2	0.2609	1	0.2677	1	0.2424	1	NA	0.4371
P47111	0.2	0	0	0	0	0	0.0714	0	NA	0.2434
O13554	0	0	0	0	0	0	0	0	NA	0.0872
P40522	0.4766	3	0.2484	1	0.2656	1	0.2891	2	NA	0.8934
P21373	0.3943	2	0.2811	2	0.2925	2	0.2547	2	NA	0.5687
Q08474	0.5789	1	0	0	0	0	0	0	NA	0.2279
Q12215	0.7626	2	0.4946	5	0.5018	4	0.5054	4	NA	0.9997
P34761	0.8321	4	0.649	5	0.5688	5	0.5764	6	NA	0.9998
Q8TGQ1	0.5147	1	0	0	0	0	0.4412	0	NA	0.5008
P38243	0.8957	2	0.5735	2	0.5308	2	0.564	2	NA	0.9971
P25561	0.1197	0	0	0	0	0	0	0	NA	0.1101
P38235	0.2235	0	0.0279	0	0	0	0.0754	0	NA	0.2186
Q12426	0.1273	0	0	0	0	0	0.0727	0	NA	0.1132
P38267	0.275	0	0.0063	0	0	0	0.2062	0	NA	0.1472
P25572	0.7143	1	0.4958	0	0.6807	1	0.6891	1	NA	0.9524
P0CX57	0.6432	2	0.5864	3	0.5545	3	0.5614	3	NA	0.9955
Q12489	0.8224	1	0.6449	1	0.8411	1	0.8318	1	NA	0.9853
Q2V2Q0	0.4824	0	0.0118	0	0	0	0.0471	0	NA	0.1841
P25654	0.2143	0	0	0	0	0	0.0385	0	NA	0.1105
Q07379	0.1372	0	0.0152	0	0	0	0.0488	0	NA	0.225
Q07454	0.2683	1	0.0894	1	0.0783	1	0.0904	1	NA	0.4691
Q03205	0.285	0	0	0	0	0	0.03	0	NA	0.1481
P0CX65	0.6432	2	0.5841	3	0.5386	3	0.5432	3	NA	0.9949
P25631	0.4054	1	0.4054	1	0.2027	0	0.2297	0	NA	0.3249
Q12148	0.35	1	0.06	0	0.27	0	0.36	1	NA	0.3116
P40917	0.7763	2	0.6915	2	0.322	0	0.3661	1	TRU	0.7357

Q12407	0.3726	4	0.1994	2	0.1907	2	0.1878	1	NA	0.5619
P40574	0.8653	2	0.5551	2	0.3429	1	0.3347	1	TRU	0.8695
Q3E756	0.266	0	0.1064	0	0	0	0.1596	0	NA	0.1115
Q8TGQ3	0.1538	0	0	0	0	0	0.2564	0	NA	0.0248
Q12424	0.2585	3	0.0157	0	0	0	0.0052	0	NA	0.1339
Q12193	0.4499	4	0.3309	5	0.2899	5	0.2847	5	NA	0.8894
Q3E762	0.2273	0	0	0	0	0	0.1515	0	NA	0.1263
P32618	0.8002	4	0.4958	4	0.41	3	0.4153	2	NA	0.9879
P40001	0.0476	0	0	0	0	0	0	0	NA	0.1194
P38317	0.0787	0	0	0	0	0	0	0	NA	0.1014
P0C268	0.322	0	0.0339	0	0	0	0.339	0	NA	0.4621
Q05899	0.4729	0	0.1085	0	0	0	0.2326	0	NA	0.3293
P39563	0.5455	1	0	0	0	0	0.1313	0	NA	0.1215
Q08521	0.1584	0	0	0	0	0	0.0693	0	NA	0.1099
Q8TGS7	0.2857	0	0	0	0	0	0.2857	0	NA	0.0248
Q08971	0.2945	0	0	0	0	0	0.089	0	NA	0.1553
Q02961	0.1414	0	0.0051	0	0	0	0.0202	0	NA	0.1147
Q08915	0.1701	0	0	0	0	0	0	0	NA	0.1047
P36151	0.1193	0	0.0142	0	0	0	0.0227	0	NA	0.1533
P0CX76	0.6432	2	0.5773	3	0.55	3	0.5591	3	NA	0.9956
Q6B0Y1	0.1329	0	0.0759	0	0	0	0	0	NA	0.2849
P0C5S0	0.6842	1	0.0105	0	0	0	0.1368	0	NA	0.7666
Q6Q571	0.1071	0	0	0	0	0	0	0	NA	0.0999
Q07986	0.2167	0	0	0	0	0	0.1429	0	NA	0.1421
Q03648	0.1597	1	0.0022	0	0	0	0.0131	0	NA	0.1811
P48559	0.5276	3	0.3046	0	0.1966	1	0.2422	1	NA	0.7819
P0C5S1	0.2692	0	0	0	0	0	0.1538	0	NA	0.0248
Q12070	0.0841	0	0	0	0	0	0.028	0	NA	0.1087
P40542	0.2984	0	0	0	0	0	0.0726	0	NA	0.164
Q3E7Z4	0.0556	0	0	0	0	0	0	0	NA	0.1254
A0A023PYH	0.0492	0	0	0	0	0	0	0	NA	0.131
P0CL32	0.1562	0	0	0	0	0	0	0	NA	0.1202
P0C5P1	0.6053	0	0.1579	0	0	0	0.5526	0	NA	0.0248
P0C5L9	0.9583	0	0.5	0	0	0	0.625	0	NA	0.0248
Q08216	0.3705	1	0.0402	0	0	0	0.0982	0	NA	0.2583
P43622	0.651	2	0.0417	0	0	0	0.1406	0	NA	0.5117
P53922	0.4099	1	0	0	0	0	0.0559	0	NA	0.1661
P0C5N8	0.0667	0	0	0	0	0	0	0	NA	0.0897
P53211	0.6881	1	0.0092	0	0	0	0.4954	0	NA	0.8049
Q8TGU1	1	1	0.2609	0	0	0	0.5435	0	NA	0.0248
P53185	0.4224	2	0.2387	1	0.1837	1	0.2167	1	NA	0.663
P53976	0.5801	4	0.2892	1	0.2075	1	0.2386	1	NA	0.9511
P38745	0.2406	1	0.0357	0	0	0	0.0677	0	NA	0.148
Q12337	0.4458	4	0.3407	6	0.2718	5	0.2678	6	NA	0.8723
Q3E737	0.4545	0	0	0	0	0	0.1818	0	NA	0.0248
Q3E816	0.0563	0	0	0	0	0	0.0704	0	NA	0.0927
P0C5N3	0.6558	2	0.0519	0	0	0	0.0519	0	NA	0.1982
Q3E750	0.1833	0	0	0	0	0	0	0	NA	0.1553
Q08746	0.7291	2	0.4877	2	0.3695	1	0.3645	0	NA	0.7755
P0CX52	0.3846	0	0.042	0	0.2028	0	0.2238	0	NA	0.1649
P47141	0.0865	0	0.0075	0	0	0	0.0113	0	NA	0.1682
Q03067	0.3939	0	0	0	0	0	0.1313	0	NA	0.1058

P53266	0.3496	1	0.1362	0	0	0	0.1748	0	NA	0.5662
P38751	0.1933	0	0	0	0	0	0.0467	0	NA	0.1549
P40316	0.9732	1	0.9115	4	0.8097	4	0.7373	1	NA	0.9166
P42941	0.1165	0	0	0	0	0	0.0194	0	NA	0.1129
P45819	0.6489	4	0.4068	1	0.3196	1	0.3801	1	NA	0.7291
P35184	0.2204	1	0.1369	1	0	0	0.1021	1	NA	0.1341
P38889	0.5788	4	0.4582	4	0.4084	4	0.41	4	TRU	0.9694
P32048	0.2205	0	0.0035	0	0	0	0.0122	0	NA	0.2444
P04801	0.2248	2	0.0668	0	0.0695	1	0.0736	1	NA	0.1768
Q12052	0.2921	1	0.1206	1	0.1524	1	0.1619	1	NA	0.1511
Q12199	0.4494	1	0.236	1	0	0	0.177	0	NA	0.473
Q3E7C1	0.25	0	0.0972	0	0	0	0.25	0	NA	0.1901
P10863	0.6378	1	0.5157	1	0.5118	1	0.4921	1	NA	0.9794
P38288	0.4462	2	0.189	0	0	0	0.211	1	NA	0.8791
P43637	0.4607	2	0.0429	0	0	0	0.05	0	NA	0.2338
P53250	0.4337	2	0.1657	1	0.1506	1	0.1536	1	NA	0.2761
P36097	0.2139	2	0.0328	1	0.0462	1	0.0568	1	NA	0.1949
P34760	0.1633	0	0	0	0	0	0.0663	0	NA	0.1175
P25037	0.4462	3	0.3399	4	0.3115	4	0.3041	3	NA	0.5258
Q12229	0.4659	3	0.1956	0	0.2549	2	0.2725	1	NA	0.7255
P42945	0.156	3	0.009	0	0	0	0.0277	0	NA	0.1605
P53254	0.2247	1	0.0833	1	0.08	1	0.0849	1	NA	0.3618
P34223	0.6407	2	0.6832	2	0.5485	2	0.5579	2	NA	0.9587
P40570	0.1694	0	0.0083	0	0	0	0.1074	0	NA	0.1861
P39111	0.3305	0	0.1017	0	0	0	0.1271	0	NA	0.1336
P39702	0.175	1	0.0298	0	0	0	0.0408	0	NA	0.274
Q04177	0.3717	3	0.2131	1	0.1913	1	0.2006	1	NA	0.3525
P38882	0.4052	2	0.1322	1	0.1722	1	0.1704	1	NA	0.3899
P33418	0.0945	0	0.0045	0	0	0	0.0136	0	NA	0.1677
P53060	0.4606	2	0.2448	0	0.1618	0	0.1494	0	NA	0.1845
P12686	0.2684	2	0.0973	0	0	0	0.1209	0	NA	0.208
Q03799	0.2452	0	0.0129	0	0	0	0.0194	0	NA	0.1333
Q12100	0.6048	2	0.5339	2	0.4968	2	0.4935	4	NA	0.9794
Q04007	0.7822	3	0.5382	4	0.4424	3	0.4458	4	NA	0.9964
P43321	0.3267	0	0.1881	0	0	0	0.2376	0	NA	0.1601
P39932	0.2021	1	0.0756	1	0.0949	1	0.0949	1	NA	0.2378
Q03497	0.7157	3	0.6954	5	0.5879	5	0.5527	5	NA	0.999
Q12153	0.5916	2	0.4371	2	0.3974	2	0.4018	2	NA	0.8369
P04803	0.2718	1	0.0369	0	0	0	0.0501	0	NA	0.1313
Q04372	0.5738	3	0.2377	1	0.2158	1	0.2268	1	NA	0.6275
P57744	0.7816	1	0	0	0	0	0.2529	0	NA	0.3565
P36145	0.5427	2	0.3506	1	0.3079	2	0.3323	1	NA	0.2777
Q07471	0.1527	1	0.0328	0	0.0624	1	0.0722	1	NA	0.1604
Q02208	0.8288	3	0.6122	3	0.4812	2	0.5032	3	NA	0.9808
P39961	0.2531	2	0.0013	0	0	0	0.0327	0	NA	0.1647
P09880	0.1439	0	0.0387	0	0	0	0.0459	0	NA	0.149
P38825	0.3959	3	0.1299	1	0.1408	1	0.1487	1	NA	0.2885
Q03796	0.1639	0	0	0	0	0	0	0	NA	0.1301
Q06412	0.3703	3	0.2028	2	0.2112	2	0.2127	2	NA	0.6593
Q04235	0.1558	0	0.0152	0	0	0	0.0325	0	NA	0.1605
Q99394	0.3731	1	0.1343	0	0.1119	0	0.1343	0	NA	0.2262
Q04411	0.399	5	0.1645	1	0.1619	1	0.1826	1	NA	0.2226

P36135	0.4411	1	0.1096	0	0	0	0.1397	0	NA	0.8594
Q12339	0.4921	1	0.4213	1	0.4409	1	0.4567	1	NA	0.728
P34110	0.1917	1	0.0212	0	0	0	0.0403	0	NA	0.2451
P32388	0.3577	0	0.0073	0	0	0	0.0584	0	NA	0.1212
P38805	0.4169	2	0.2102	1	0.1424	0	0.1559	0	NA	0.1885
P36517	0.4263	1	0.1755	1	0.2006	1	0.2132	1	NA	0.2797
P32496	0.3139	1	0.0401	0	0	0	0.0292	0	NA	0.1427
Q02933	0.1429	1	0.0184	0	0	0	0.0276	0	NA	0.1276
P07280	0.3403	0	0.1944	0	0	0	0.25	0	NA	0.1843
P0CX39	0.545	2	0.355	1	0.2	1	0.24	1	NA	0.2544
Q04003	0.8358	4	0.4595	1	0	0	0.1767	0	NA	0.5841
P34758	0.8979	2	0.7741	4	0.7626	5	0.7076	9	NA	0.9993
P89114	0.7033	1	0.1429	0	0	0	0.2308	0	NA	0.1938
Q9ZZX1	0.1143	1	0.0048	0	0	0	0.0048	0	NA	0.121
P33757	0.2983	1	0.0153	0	0	0	0.0822	0	NA	0.2046
P38839	0.7568	1	0.4257	0	0	0	0.4797	0	NA	0.8427
Q12078	0.0782	0	0	0	0	0	0	0	NA	0.1153
Q02793	0.1763	0	0	0	0	0	0.0126	0	NA	0.2135
P46675	0.58	2	0.2511	1	0	0	0.1982	0	NA	0.9199
P50104	0.4046	2	0.254	3	0.2171	2	0.2297	2	TRU	0.5108
P09436	0.1371	0	0.0075	0	0	0	0.0205	0	NA	0.1324
P11325	0.2092	1	0.0201	0	0	0	0.0291	0	NA	0.2813
P53038	0.2558	2	0.016	0	0	0	0.0422	0	NA	0.1821
Q06417	0.0123	0	0	0	0	0	0	0	NA	0.1082
P53040	0.2442	1	0.0795	0	0	0	0.0678	0	NA	0.347
Q12415	0.3862	3	0.2276	1	0.1977	1	0.2115	1	NA	0.4584
Q06451	0.3264	1	0.2315	2	0.2299	2	0.2186	2	NA	0.3752
P04786	0.5345	3	0.2289	1	0.2094	1	0.2302	1	NA	0.433
P06785	0.2862	1	0.0164	0	0	0	0.0559	0	NA	0.1916
P47168	0.1093	0	0	0	0	0	0.0166	0	NA	0.1203
P06104	0.5233	1	0.314	0	0.2733	0	0.2791	0	NA	0.3034
P18562	0.0648	0	0.0046	0	0	0	0.0324	0	NA	0.1163
Q08562	0.5374	6	0.2532	5	0.1878	5	0.1748	4	NA	0.6536
Q03010	0.2739	1	0.0217	0	0	0	0.0522	0	NA	0.1761
Q05919	0.262	1	0.0159	0	0	0	0.0182	0	NA	0.1805
P40453	0.423	3	0.2017	1	0.1811	2	0.197	2	NA	0.4891
P39968	0.2768	1	0.0692	1	0.0744	1	0.0934	1	NA	0.1639
P33301	0.6581	2	0.4063	3	0.2319	1	0.2482	2	NA	0.8837
P28000	0.4859	1	0.5141	1	0.4577	1	0.4507	1	NA	0.2439
P50263	1	1	0.9747	1	1	1	1	1	NA	0.9946
P15367	0.1138	0	0	0	0	0	0	0	NA	0.1114
Q08826	0.321	1	0.0494	0	0	0	0.1605	0	NA	0.1613
P38863	0.2199	2	0.034	0	0	0	0.0437	0	NA	0.2167
Q12455	0.1067	0	0.0067	0	0	0	0.0367	0	NA	0.1102
P11484	0.3719	2	0.0196	0	0	0	0.0163	0	NA	0.1598
Q12034	0.7405	2	0.5861	1	0.5324	2	0.4877	2	NA	0.9774
P53438	0.8548	3	0.7631	4	0.6904	4	0.6561	4	TRU	0.9998
P35180	0.541	1	0.2896	0	0	0	0.2295	0	NA	0.3215
Q05024	0.646	2	0.3584	1	0.3407	2	0.3274	1	NA	0.8173
Q03660	0.1016	0	0.0027	0	0	0	0.0181	0	NA	0.1724
P38962	0.1256	0	0	0	0	0	0.0704	0	NA	0.1227
P38187	0.5609	3	0.3039	1	0.3079	1	0.336	2	NA	0.7054

P43604	0.3491	1	0.142	0	0	0	0.2071	0	NA	0.5502
P47049	0.4596	2	0.197	1	0.1818	1	0.1818	1	NA	0.3029
P32610	0.5	2	0.1406	0	0.207	1	0.2188	1	NA	0.1727
P32319	0.2755	5	0.0627	1	0.0564	1	0.0817	1	NA	0.3662
P23643	0.3076	3	0.1246	1	0.1286	1	0.1325	1	NA	0.1621
P38735	0.1633	1	0.0113	0	0	0	0.0258	0	NA	0.1633
P40547	0.2161	1	0.0901	1	0.0912	1	0.1075	1	NA	0.3069
P32912	0.6835	2	0.3513	1	0	0	0.1835	0	NA	0.2916
O13513	0.25	0	0	0	0	0	0.0345	0	NA	0.1788
O13527	0.3829	2	0.2517	1	0.2341	1	0.2341	1	NA	0.5934
Q07530	0.1558	0	0.039	0	0	0	0.0844	0	NA	0.1207
Q03831	0.4632	1	0.2206	0	0	0	0.3235	0	NA	0.4978
Q12027	0.2175	1	0.0847	0	0	0	0.1017	0	NA	0.378
P87280	0.0642	0	0	0	0	0	0	0	NA	0.103
Q3E6R5	0.6053	1	0.1667	0	0	0	0.3596	0	NA	0.3127
Q03057	0.8803	1	0.6127	1	0.4718	1	0.4366	1	NA	0.8539
Q04162	0.1441	1	0.036	0	0	0	0.0865	0	NA	0.259
P39710	0.1863	0	0	0	0	0	0	0	NA	0.1036
P32622	0.2687	1	0.103	1	0.1111	1	0.1172	1	NA	0.1821
Q04434	0.1145	0	0	0	0	0	0	0	NA	0.1069
P87263	0	0	0	0	0	0	0	0	NA	0.1034
Q03619	0.4564	4	0.3322	4	0.3037	4	0.3009	4	NA	0.8861
Q03051	0.2626	0	0.0404	0	0	0	0.2424	0	NA	0.1335
Q05867	0.6051	3	0.1561	0	0.1943	1	0.2102	1	NA	0.4871
P36079	0.0294	0	0	0	0	0	0	0	NA	0.1815
O13541	0.8276	1	0.7155	1	0.8017	1	0.8017	1	NA	0.978
P0C5P8	0.2143	0	0	0	0	0	0.3214	0	NA	0.0248
Q04526	0.3804	1	0.11	0	0	0	0.0933	0	NA	0.4078
P46986	0.381	1	0	0	0	0	0.0762	0	NA	0.1011
Q04674	0.4553	1	0	0	0	0	0.0732	0	NA	0.1613
Q06595	0.183	1	0	0	0	0	0.0447	0	NA	0.1111
P53054	0.2629	0	0.0171	0	0	0	0.0229	0	NA	0.11
Q08989	0.2752	1	0.1109	1	0.0945	1	0.1047	1	NA	0.289
Q12261	0.121	0	0	0	0	0	0.0637	0	NA	0.1263
P53342	0.2157	0	0	0	0	0	0.0588	0	NA	0.1374
P0CL31	0.7749	1	0.3403	1	0.3822	1	0.466	1	NA	0.9992
P53316	0.5583	6	0.2714	1	0.2074	2	0.21	3	NA	0.9256
P53825	0.3817	1	0	0	0	0	0.0382	0	NA	0.1581
Q12244	0.2491	1	0.0237	0	0	0	0.0237	0	NA	0.2199
P0CX69	0.6432	2	0.5841	3	0.5386	3	0.5432	3	NA	0.9949
Q12528	0.3091	0	0.0182	0	0	0	0.2273	0	NA	0.3478
O13562	0.1628	0	0	0	0	0	0	0	NA	0.0928
P40583	0.1993	1	0.0149	0	0	0	0.0391	0	NA	0.257
P40211	0.3627	0	0.0392	0	0	0	0.1373	0	NA	0.2268
Q8TGQ7	0.2727	0	0	0	0	0	0.303	0	NA	0.0248
Q3E806	0.7971	1	0.1014	0	0	0	0.3333	0	NA	0.4246
Q8TGT0	0.5263	0	0.1228	0	0.4211	0	0.4737	0	NA	0.4878
P0C5R4	0.1957	0	0	0	0	0	0.1522	0	NA	0.0248
Q8TGL1	0.3462	0	0	0	0	0	0.4231	0	NA	0.0248
O13531	0.0556	0	0	0	0	0	0	0	NA	0.0934
Q8TGS0	0.3542	0	0	0	0	0	0.375	0	NA	0.5581
Q05948	0.2506	1	0.0025	0	0	0	0.0393	0	NA	0.178

P36083	0.4978	4	0.1889	0	0.2756	1	0.2778	1	NA	0.8567
O13550	0.1121	0	0	0	0	0	0.0776	0	NA	0.1269
Q04867	0.1893	0	0.0126	0	0	0	0.0631	0	NA	0.1536
Q8TGT5	0.3077	0	0	0	0	0	0.2821	0	NA	0.0248
P0C2I5	0.4507	4	0.3345	4	0.3043	4	0.3077	4	NA	0.8965
Q06152	0.7263	2	0.5292	1	0.5036	2	0.4124	1	NA	0.9673
P43566	0.3774	0	0.0755	0	0	0	0.3585	0	NA	0.3382
P38806	0.6348	2	0.4184	1	0.4433	1	0.4326	1	NA	0.9646
P40514	0.1917	1	0.0649	0	0.0723	0	0.0885	0	NA	0.1952
P28626	0.0345	0	0	0	0	0	0	0	NA	0.0973
P53752	0.211	1	0.0069	0	0	0	0.0161	0	NA	0.1546
P43546	0.3947	1	0.0855	0	0	0	0.1447	0	NA	0.2444
P38721	0.3242	1	0.1621	1	0.1894	1	0.2009	1	NA	0.5286
P0CF22	0.1333	0	0.0095	0	0	0	0.081	0	NA	0.1048
Q08465	0.5297	1	0.1416	0	0	0	0.1826	0	NA	0.609
Q08172	0.3721	0	0	0	0	0	0.0581	0	NA	0.2164
P53757	0.2105	0	0.0643	0	0	0	0.0439	0	NA	0.1543
Q8TGN1	0.1837	0	0	0	0	0	0.2041	0	NA	0.1975
P0CL23	0.2222	0	0	0	0	0	0	0	NA	0.0248
P48525	0.2257	1	0	0	0	0	0.0149	0	NA	0.1207
P36100	0.7075	2	0.5996	1	0.5705	2	0.5622	2	NA	0.9961
P36421	0.3198	2	0.1345	1	0.1675	1	0.1802	1	NA	0.3257
P13393	0.3417	1	0.1958	1	0.2042	1	0.2042	1	NA	0.2879
P38114	0.4022	4	0.245	2	0.213	2	0.2102	2	NA	0.5665
P25638	0.1892	0	0.045	0	0	0	0.1802	0	NA	0.167
P35189	0.3361	1	0.2787	1	0.1885	1	0.2049	1	NA	0.4401
P53215	0.1477	0	0	0	0	0	0.0253	0	NA	0.1017
Q06490	0.1031	0	0	0	0	0	0.0227	0	NA	0.1825
P38141	0.3133	3	0.1089	0	0.1	0	0.1578	0	TRU	0.3067
P53177	0.359	1	0.1282	1	0.1538	1	0.1538	1	NA	0.2467
P21734	0.2558	0	0.1349	0	0	0	0.1488	0	NA	0.6409
P15731	0.2703	0	0.0405	0	0	0	0.1892	0	NA	0.2826
Q03337	0.4488	2	0.0954	0	0.1943	0	0.2085	1	NA	0.5259
Q08747	0.5921	2	0.2851	1	0.2675	0	0.2807	1	NA	0.8386
P32893	0.3714	2	0.1018	0	0.0911	0	0.1054	1	NA	0.6489
P36017	0.4476	1	0.1667	0	0.1619	0	0.2	1	NA	0.1809
Q06679	0.2668	1	0.0696	0	0	0	0.0399	0	NA	0.2077
P42826	0.2117	0	0.0217	0	0	0	0.0183	0	NA	0.1288
P40438	0.2298	3	0.0639	1	0.0613	1	0.0671	1	NA	0.396
Q12147	0.1359	0	0	0	0	0	0	0	NA	0.1228
P38268	0.2286	0	0	0	0	0	0.04	0	NA	0.1357
O13515	0.2906	0	0	0	0	0	0	0	NA	0.1227
Q02896	0.0726	0	0.0095	0	0	0	0.041	0	NA	0.105
O13512	0.2778	0	0.0397	0	0	0	0	0	NA	0.1482
P25607	0.033	0	0	0	0	0	0.0275	0	NA	0.1205
Q07535	0.127	0	0	0	0	0	0.1032	0	NA	0.1547
P38966	0.4005	4	0.1151	1	0.1082	2	0.1358	2	NA	0.5883
P25601	0.3836	0	0.0342	0	0	0	0.0822	0	NA	0.1923
P0C5M1	0.1765	0	0	0	0	0	0.1765	0	NA	0.0248
P25640	0.0147	0	0	0	0	0	0	0	NA	0.0917
Q00590	0.45	1	0	0	0	0	0	0	NA	0.1866
A0A023PXC	0.1049	0	0	0	0	0	0	0	NA	0.1122

P0C269	0.2821	0	0	0	0	0	0.2821	0	NA	0.0248
Q02217	0.16	0	0	0	0	0	0.03	0	NA	0.1337
Q07791	0.4452	4	0.3446	6	0.2859	5	0.2819	7	NA	0.8815
Q7M4S9	0.6111	1	0.4811	1	0.4571	1	0.4874	1	NA	0.9991
P87269	0.6164	1	0.0274	0	0	0	0.1575	0	NA	0.4911
A0A023PZC	0.2124	0	0	0	0	0	0	0	NA	0.1598
A0A023PXJ	0.2174	0	0	0	0	0	0	0	NA	0.1056
Q3E820	0.0612	0	0	0	0	0	0	0	NA	0.1326
P0CL26	0.04	0	0	0	0	0	0.0533	0	NA	0.106
P47004	0.14	0	0	0	0	0	0	0	NA	0.1087
P03881	0.1398	0	0	0	0	0	0.0021	0	NA	0.1454
Q06158	0.1913	0	0	0	0	0	0	0	NA	0.1513
P0C5Q0	1	1	0.0652	0	0	0	0.3043	0	NA	0.0248
P0C5R5	0.0233	0	0	0	0	0	0.0698	0	NA	0.1276
Q03207	0.2698	0	0	0	0	0	0.0556	0	NA	0.1103
Q03161	0.1785	0	0.0572	0	0	0	0.0471	0	NA	0.136
Q12391	0.6455	2	0.5886	3	0.5659	3	0.5523	3	NA	0.9961
Q12042	0.1795	0	0	0	0	0	0.0696	0	NA	0.1696
Q12439	0.6119	2	0.5548	3	0.4498	3	0.4566	3	NA	0.797
P38070	0.4514	3	0.1429	0	0.1181	0	0.139	0	NA	0.4704
Q07927	0.303	0	0	0	0	0	0.0909	0	NA	0.1137
P53181	0.0495	0	0	0	0	0	0	0	NA	0.0899
A0A023PXE	0.5214	1	0	0	0	0	0.1026	0	NA	0.5184
Q3E754	0.2759	0	0.046	0	0	0	0.1264	0	NA	0.1229
P07281	0.3403	0	0.1944	0	0	0	0.2361	0	NA	0.2
Q02983	0.4959	2	0.1791	1	0.1928	1	0.2066	1	NA	0.8122
P14127	0.5515	1	0.0882	0	0	0	0.1838	0	NA	0.2465
P40033	0.5652	1	0.2899	0	0	0	0.1449	0	NA	0.2134
Q07534	0.1759	0	0.0228	0	0	0	0.0391	0	NA	0.104
Q03246	0.5907	2	0.0464	0	0	0	0.0506	0	NA	0.1793
P38120	0.3813	0	0.0827	0	0	0	0.0935	0	NA	0.3111
P46674	0.5327	4	0.3036	5	0.2314	4	0.236	3	NA	0.8955
P53850	0.5711	1	0.1546	1	0.1646	1	0.1895	0	NA	0.2759
P42223	0.6412	2	0.294	2	0.2072	1	0.2431	1	NA	0.9064
Q05900	0.684	3	0.5325	2	0.4199	1	0.4113	1	NA	0.9175
P39980	0.1863	2	0.0016	0	0	0	0.0366	0	NA	0.1553
P38345	0.8696	1	0.7826	2	0.7464	2	0.6739	1	NA	0.5504
P40075	0.4877	1	0.3607	1	0.377	1	0.4016	1	NA	0.8502
P32602	0.339	1	0.0205	0	0	0	0.0719	0	NA	0.1406
Q07953	0.256	1	0.016	0	0	0	0.096	0	NA	0.1842
P18410	0.251	1	0.1583	0	0.2008	1	0.2085	1	NA	0.2048
Q03707	0.6127	2	0.4796	1	0.4436	1	0.446	2	NA	0.9574
P38342	0.0813	0	0.0031	0	0	0	0.0344	0	NA	0.1069
P38820	0.175	1	0.0364	0	0	0	0.05	0	NA	0.1281
Q02724	0.6312	3	0.2367	1	0.1771	2	0.2174	2	NA	0.8056
Q04500	0.9255	3	0.8532	3	0.6552	4	0.6429	4	NA	0.9736
P39001	0.9713	2	0.9115	5	0.8182	7	0.7943	7	TRU	1
P32913	0.5408	3	0.3358	2	0.2958	2	0.3212	2	NA	0.6753
P54860	0.257	2	0.0552	0	0	0	0.0552	0	NA	0.1857
P22147	0.3645	5	0.1983	2	0.1492	2	0.1538	2	NA	0.4804
Q07993	0.0927	0	0.014	0	0	0	0.0365	0	NA	0.1336
P25383	0.6142	2	0.5616	3	0.4612	3	0.4566	3	NA	0.8872

P38275	0.2707	0	0.0376	0	0	0	0	0	NA	0.1711
Q3E7Z8	0.0114	0	0	0	0	0	0	0	NA	0.0881
P37263	0.732	1	0.2876	1	0.3464	1	0.3464	1	NA	0.513
P38161	0.0792	0	0	0	0	0	0.0297	0	NA	0.0914
P25565	0.0342	0	0	0	0	0	0	0	NA	0.1061
P0C5M5	0.1081	0	0	0	0	0	0.1081	0	NA	0.0248
Q04597	0.03	0	0	0	0	0	0	0	NA	0.0942
P38194	0.6148	0	0.1885	0	0	0	0.1721	0	NA	0.1837
P25614	0.814	2	0.093	0	0	0	0.1395	0	NA	0.6657
Q12441	0.6432	2	0.5773	3	0.5364	3	0.5432	3	NA	0.9948
P0C5L6	0.1463	0	0	0	0	0	0	0	NA	0.1232
Q12082	0.4407	0	0.0508	0	0	0	0.1695	0	NA	0.1699
Q3E6R4	0.5909	1	0.2273	0	0	0	0.2727	0	NA	0.5698
Q3E821	0.2025	0	0	0	0	0	0	0	NA	0.1366
P25652	0.0526	0	0	0	0	0	0	0	NA	0.1221
P89888	0.2857	1	0.0246	0	0	0	0.133	0	NA	0.2803
Q04170	0.194	0	0.0474	0	0	0	0.0776	0	NA	0.1348
P87285	0.2	0	0	0	0	0	0	0	NA	0.1298
P0C270	0.4412	0	0	0	0	0	0.1765	0	NA	0.0248
Q8TGK7	0.2976	0	0	0	0	0	0.2024	0	NA	0.2211
P38309	0.6449	1	0	0	0	0	0	0	NA	0.1402
Q3E7Y4	0.1048	0	0	0	0	0	0.0286	0	NA	0.1474
Q8TGR7	0.2105	0	0	0	0	0	0.1842	0	NA	0.0248
Q06127	0.0952	0	0	0	0	0	0	0	NA	0.1042
P42947	0.8527	1	0.739	2	0.5401	2	0.5969	2	NA	0.9907
P36072	0.0874	0	0	0	0	0	0	0	NA	0.0984
P53133	0.1887	0	0	0	0	0	0.0377	0	NA	0.134
Q03080	0.4367	1	0.1994	1	0.2184	1	0.231	1	NA	0.5947
Q06051	0.0764	0	0	0	0	0	0.0573	0	NA	0.106
P53134	0.1669	1	0.029	0	0	0	0.0331	0	NA	0.2455
P0C5P5	0.3247	0	0	0	0	0	0.0909	0	NA	0.097
P40896	0.1118	0	0	0	0	0	0.0151	0	NA	0.1811
P36140	0.4455	1	0.0297	0	0	0	0.2178	0	NA	0.159
Q06493	0.4207	3	0.1806	0	0.196	1	0.2159	2	NA	0.3084
P46945	0.2852	1	0.0596	0	0	0	0.0812	0	NA	0.3249
P53826	0.0822	0	0	0	0	0	0	0	NA	0.1704
Q3E7Z0	0.3065	0	0.0161	0	0	0	0.2581	0	NA	0.3472
Q12160	0.35	1	0.1857	0	0.2286	0	0.2571	0	NA	0.3331
P0C2I9	0.4479	4	0.3379	4	0.3077	4	0.3077	4	NA	0.9
Q07811	0.3069	0	0	0	0	0	0.0495	0	NA	0.1153
Q12744	0.1429	0	0	0	0	0	0.0301	0	NA	0.103
Q8TGS9	0.2368	0	0	0	0	0	0.0395	0	NA	0.118
Q08964	0.3176	4	0.0336	0	0	0	0.0417	1	NA	0.2674
Q02981	0.102	0	0	0	0	0	0.0076	0	NA	0.1195
A0A023PYJ(	0.1389	0	0	0	0	0	0	0	NA	0.1402
Q8TGK9	0.4386	0	0.0877	0	0	0	0.3684	0	NA	0.5479
P32859	0.4923	1	0.0692	0	0	0	0.3231	1	NA	0.7418
Q03508	0.1887	1	0.0043	0	0	0	0.0456	0	NA	0.1604
Q03879	0.1532	0	0	0	0	0	0.0484	0	NA	0.1054
Q07880	0.1757	0	0	0	0	0	0	0	NA	0.1687
P46951	0.1775	0	0.0233	0	0	0	0.0282	0	NA	0.1549
Q04711	0.4627	4	0.3362	4	0.2986	4	0.3043	4	NA	0.8965

Q08930	0.3472	1	0.0556	0	0.1417	1	0.1389	1	NA	0.1502
Q04909	0.1	0	0	0	0	0	0	0	NA	0.1058
P53182	0.1642	0	0	0	0	0	0	0	NA	0.2198
Q8TGM0	0.1622	0	0	0	0	0	0.1622	0	NA	0.0248
A0A023PZD	0.0915	0	0	0	0	0	0	0	NA	0.1414
Q3E732	0.2368	0	0	0	0	0	0.1316	0	NA	0.0248
P40539	0.0909	0	0	0	0	0	0	0	NA	0.0973
P43552	0.275	0	0.0125	0	0	0	0.05	0	NA	0.1651
P38727	0.166	0	0	0	0	0	0	0	NA	0.1251
P43608	0.3158	1	0	0	0	0	0.1228	0	NA	0.104
A0A023PXD	0.581	1	0.3143	1	0.4381	1	0.4762	1	NA	0.8549
P40446	0.1261	0	0.0084	0	0	0	0.0756	0	NA	0.1508
A0A023PXF	0.1226	0	0	0	0	0	0	0	NA	0.0988
P0CI67	0.4444	1	0.2424	0	0	0	0.2222	0	NA	0.2817
P0CX61	0.621	2	0.5594	3	0.4658	3	0.4635	3	NA	0.8379
P0CX89	0.6667	1	0.036	0	0	0	0.1622	0	NA	0.1358
P40584	0.0364	0	0	0	0	0	0.0273	0	NA	0.1014
P53925	0.2842	3	0.1211	0	0.1677	2	0.1708	2	NA	0.4732
P0CL33	0.1562	0	0	0	0	0	0	0	NA	0.1202
Q8TGN4	0.0674	0	0	0	0	0	0	0	NA	0.0894
P53907	0.5851	3	0.3257	4	0.3149	3	0.3378	2	NA	0.9677
P0CL36	0.2125	0	0	0	0	0	0	0	NA	0.1461
P53977	0.3125	1	0	0	0	0	0.125	0	NA	0.167
P47137	0.078	0	0.0071	0	0	0	0.0426	0	NA	0.1103
Q06676	0.1241	0	0	0	0	0	0	0	NA	0.1566
Q08240	0.0552	0	0	0	0	0	0	0	NA	0.1026
P47121	0.1311	0	0	0	0	0	0	0	NA	0.135
P53307	0.0796	0	0	0	0	0	0	0	NA	0.1643
P47080	0.4519	1	0.125	0	0	0	0.2596	0	NA	0.5141
P53952	0.8444	2	0.5741	1	0.7037	3	0.6778	4	NA	0.9261
P53247	0.0081	0	0	0	0	0	0	0	NA	0.1078
P0CL27	0.7749	1	0.3403	1	0.3822	1	0.466	1	NA	0.9992
Q12327	0.2719	0	0	0	0	0	0.0702	0	NA	0.1174
P53061	0.3082	2	0.0327	0	0	0	0.0384	0	NA	0.19
Q06511	0.952	2	0.556	1	0.552	1	0.54	1	NA	0.9045
P35179	0.35	0	0.1625	0	0	0	0.2375	0	NA	0.201
Q04279	1	1	0.9823	5	0.9646	3	0.9521	5	NA	0.9993
P36057	0.3648	1	0	0	0	0	0.0287	0	NA	0.15
P22215	0.2031	0	0.0132	0	0	0	0.128	0	NA	0.1851
P53955	0.6418	4	0.346	4	0.2912	2	0.2759	1	NA	0.6653
P25582	0.6076	5	0.4435	3	0.3793	2	0.3555	3	NA	0.6772
P38705	0.2309	1	0.009	0	0	0	0.0067	0	NA	0.156
P41896	0.6675	4	0.4825	2	0.3675	3	0.3825	3	NA	0.812
P48527	0.2846	1	0.0407	0	0	0	0.0447	0	NA	0.1469
P53072	0.4602	2	0.2007	0	0.3322	1	0.2941	1	NA	0.52
P40085	0.2608	1	0.0899	0	0	0	0.1187	0	NA	0.3458
P15019	0.3104	1	0.0746	0	0	0	0.0537	0	NA	0.2073
Q05998	0.1957	1	0.0619	0	0.0936	1	0.0936	1	NA	0.1957
P42938	0.231	0	0.0069	0	0	0	0.0414	0	NA	0.1511
P47183	0.1382	0	0	0	0	0	0	0	NA	0.1166
P53257	0.1146	0	0	0	0	0	0	0	NA	0.1134
P07213	0.5089	4	0.1345	1	0.1491	1	0.1491	1	NA	0.5667

Q12400	0.3823	1	0.2696	1	0.2048	1	0.215	1	NA	0.2341
Q03280	0.2742	5	0.1252	2	0.1007	3	0.1019	3	NA	0.4936
P07285	0.2737	0	0.0105	0	0	0	0.0342	0	NA	0.1892
P23202	0.322	1	0.2655	1	0.2768	1	0.2768	1	NA	0.3579
P08067	0.3581	1	0.093	0	0	0	0.0698	0	NA	0.2268
P36116	0.7866	1	0.5549	1	0.561	1	0.5488	1	NA	0.9597
P53058	0.0485	0	0	0	0	0	0	0	NA	0.1321
Q03085	0.1068	0	0	0	0	0	0.0249	0	NA	0.1074
P53315	0.1137	0	0	0	0	0	0	0	NA	0.1351
P36077	0.5433	1	0.0709	0	0	0	0.189	0	NA	0.3214
Q12469	0.3252	1	0.1679	1	0.1252	1	0.1573	2	NA	0.2271
Q07084	0.5351	5	0.4284	4	0.3933	4	0.3947	3	NA	0.9888
P07263	0.3168	2	0.0513	0	0	0	0.0861	0	NA	0.1944
Q04048	0.1746	0	0.021	0	0	0	0.0349	0	NA	0.1348
P38758	0.369	1	0.1721	2	0.1855	1	0.2103	1	NA	0.6639
Q06817	0.2249	1	0.0081	0	0	0	0.0307	0	NA	0.1808
P47153	0.1004	0	0	0	0	0	0	0	NA	0.1363
Q04545	0.454	6	0.2462	3	0.211	3	0.2006	3	TRU	0.8728
Q08921	0.8949	4	0.8048	6	0.7322	4	0.6783	5	NA	0.9988
P39076	0.2144	0	0.0114	0	0	0	0.0361	0	NA	0.1396
Q07914	0.7143	2	0.2024	0	0.2024	0	0.2143	0	NA	0.5301
Q03784	0.3836	1	0.1689	0	0	0	0.1963	0	NA	0.5783
P50101	0.1911	1	0.0488	0	0	0	0.0463	0	NA	0.2632
P25515	0.0875	0	0	0	0	0	0	0	NA	0.1527
Q03390	1	1	0.7162	0	0.4367	1	0.3755	1	NA	0.8076
P25594	0.0873	0	0.0197	0	0	0	0.0284	0	NA	0.1045
P42839	0.3161	3	0.2137	2	0.2181	2	0.2115	2	NA	0.6146
Q02792	0.3946	4	0.2674	4	0.1491	1	0.173	1	NA	0.3883
P53832	0.7435	3	0.5149	2	0.4573	2	0.5189	2	NA	0.9994
P38838	0.4349	2	0.1375	0	0.1004	0	0.1413	0	NA	0.2133
P38215	0.1628	0	0	0	0	0	0	0	NA	0.139
P38252	0.3618	0	0.005	0	0	0	0.0352	0	NA	0.3809
P25563	0.1304	0	0	0	0	0	0.0696	0	NA	0.0977
Q07790	0.0229	0	0	0	0	0	0	0	NA	0.0981
P25350	0.1083	0	0.0255	0	0	0	0.0191	0	NA	0.1024
Q07555	0.8351	2	0.6598	2	0.5533	2	0.6014	3	NA	0.9886
Q12281	0.0485	0	0	0	0	0	0	0	NA	0.1343
Q03900	0.2727	2	0.0364	0	0	0	0.0606	0	NA	0.2413
P25347	0.0865	0	0	0	0	0	0	0	NA	0.1072
P0C2I3	0.4496	4	0.3339	4	0.3031	4	0.3066	4	NA	0.8925
Q03780	0.9886	3	0.737	4	0.5794	3	0.5858	2	NA	0.9995
P38987	0.2286	1	0.151	1	0.1918	1	0.1959	1	NA	0.1943
Q03761	0.7922	4	0.7199	3	0.5028	3	0.475	3	NA	0.9378
P53552	0.3644	4	0.2029	2	0.186	2	0.1904	1	NA	0.6949
Q03525	0.6872	1	0.6351	1	0.5877	1	0.6209	1	NA	0.9897
Q08687	0.618	2	0.3989	0	0	0	0.309	0	NA	0.3026
Q04767	0.1078	0	0	0	0	0	0.018	0	NA	0.1048
P15732	0.3108	0	0.0135	0	0	0	0.1757	0	NA	0.266
Q08926	0.7697	1	0.7401	1	0.6908	1	0.6908	1	NA	0.9884
P40537	0.6809	3	0.4971	4	0.4845	6	0.471	5	NA	0.9877
Q03714	0.2554	3	0.1062	1	0.1122	1	0.1217	1	NA	0.3062
Q05947	0.1491	0	0.0298	0	0	0	0.0921	0	NA	0.2395

P31412	0.2143	0	0.0026	0	0	0	0.0204	0	NA	0.1205
P32341	0.4791	1	0	0	0	0	0.0419	0	NA	0.1335
Q03944	0.6457	4	0.4901	3	0.3742	1	0.3808	2	NA	0.6662
Q08438	0.727	3	0.6409	3	0.6202	5	0.5994	4	NA	0.9994
Q02354	0.2023	1	0.0659	0	0.0773	1	0.075	0	NA	0.1701
Q3E830	0.1733	0	0.0267	0	0	0	0.2133	0	NA	0.6423
P38253	0.3934	1	0.1148	0	0	0	0.2787	0	NA	0.6814
P38201	0.6516	2	0.3803	2	0.4229	3	0.4043	2	NA	0.991
A0A023PZE2	0.1927	0	0	0	0	0	0.0642	0	NA	0.1459
Q03855	0.4598	5	0.3385	4	0.3037	4	0.3066	4	NA	0.9001
P48569	0.4375	2	0.0469	0	0	0	0.0938	0	NA	0.1915
A0A023PXI4	0.2571	0	0	0	0	0	0	0	NA	0.1172
P48568	0.7545	3	0.4657	0	0	0	0.3394	0	NA	0.4517
Q12121	0.6909	3	0.1828	0	0	0	0.1505	0	NA	0.4353
P39983	0.2232	0	0.0987	0	0	0	0.1116	0	NA	0.1137
Q04100	0.2815	1	0	0	0	0	0.037	0	NA	0.1377
Q8TGU5	0.4615	0	0	0	0	0	0.1795	0	NA	0.0248
P0CX95	0.2125	0	0	0	0	0	0	0	NA	0.1412
P38142	0.1291	0	0.0574	0	0.082	0	0.0984	0	NA	0.1676
P39549	0.5811	1	0.2297	0	0	0	0.473	0	NA	0.154
Q04502	0.1393	0	0	0	0	0	0	0	NA	0.1619
O13557	0.3005	1	0.0273	0	0	0	0.1257	0	NA	0.293
A5Z2X5	0.4444	0	0	0	0	0	0.1944	0	NA	0.1203
P28320	0.8345	2	0.5791	1	0.4101	1	0.4065	1	NA	0.6314
Q06057	0.1746	0	0	0	0	0	0	0	NA	0.2847
Q8TGM9	0.236	0	0	0	0	0	0	0	NA	0.0962
P39541	0.133	0	0	0	0	0	0.03	0	NA	0.179
P40323	0.0435	0	0	0	0	0	0	0	NA	0.1064
Q8TGT2	0.5714	0	0	0	0	0	0.0612	0	NA	0.1016
Q04501	0.0156	0	0	0	0	0	0.0156	0	NA	0.0881
P34231	0.4933	3	0.096	1	0.0987	1	0.1387	1	NA	0.732
Q02203	0.3657	3	0.1429	0	0	0	0.1333	0	NA	0.3534
Q3E758	0.1591	0	0	0	0	0	0.1591	0	NA	0.0248
Q6B2I9	0.1009	0	0	0	0	0	0	0	NA	0.1959
Q3E7A8	0.2778	0	0.0139	0	0	0	0.2361	0	NA	0.1256
Q07881	0.0866	0	0	0	0	0	0	0	NA	0.1107
Q12501	0.4508	4	0.3503	6	0.2876	5	0.2814	7	NA	0.9066
Q3E826	0.7179	1	0.2564	0	0	0	0.5769	0	NA	0.9677
Q06537	0.0714	0	0	0	0	0	0.0714	0	NA	0.1227
O13583	0.065	0	0	0	0	0	0.0488	0	NA	0.1211
Q12697	0.2955	2	0.1501	3	0.1705	4	0.1556	4	NA	0.6841
Q3E804	0.6	0	0.0182	0	0	0	0.3455	0	NA	0.5835
Q08321	0.0177	0	0	0	0	0	0	0	NA	0.0987
Q06247	0.1266	1	0.0345	0	0.0444	0	0.0576	0	NA	0.1723
A0A023PZG	0.3832	0	0.028	0	0	0	0	0	NA	0.1912
P38146	0.2161	0	0.005	0	0	0	0.0553	0	NA	0.117
Q02209	0.3654	2	0.17	0	0.1841	0	0.1898	1	NA	0.2714
Q08900	0.1393	0	0	0	0	0	0	0	NA	0.1346
Q08428	0.8319	1	0.4867	1	0.4867	1	0.469	1	NA	0.9907
P38730	0.0738	0	0	0	0	0	0	0	NA	0.095
P40560	0.0526	0	0	0	0	0	0	0	NA	0.1108
P38780	0.2401	0	0.0169	0	0	0	0.0819	0	NA	0.6224

P53039	0.2782	1	0.0484	0	0	0	0.121	0	NA	0.2123
P43597	0.9278	1	0.8954	3	0.837	9	0.8078	8	NA	0.9991
P40488	0.1584	0	0.0891	0	0	0	0.1683	0	NA	0.1126
A0A023PZI4	0.0917	0	0	0	0	0	0	0	NA	0.1142
P53857	0.5211	2	0.3615	2	0.3638	2	0.3638	2	NA	0.9098
P0C5M9	0.2706	0	0	0	0	0	0.0353	0	NA	0.1135
P47100	0.4501	4	0.335	4	0.3117	4	0.3128	4	NA	0.8861
Q08411	0.6853	1	0	0	0	0	0.0909	0	NA	0.3277
P0C2J7	0.4262	5	0.2181	1	0	0	0.1199	1	NA	0.2932
P53093	0.2468	1	0.0553	0	0	0	0.1021	0	NA	0.1295
A0A023PXF	0.1048	0	0	0	0	0	0.0286	0	NA	0.1474
Q08207	0.06	0	0	0	0	0	0	0	NA	0.1032
P40009	0.2905	1	0.0429	0	0.0635	0	0.073	0	NA	0.2822
P53308	0.7018	1	0	0	0	0	0	0	NA	0.2388
P53216	0.38	0	0	0	0	0	0.12	0	NA	0.1152
Q8TGP0	0.6531	0	0	0	0	0	0.4286	0	NA	0.4604
P0CX67	0.6432	2	0.5841	3	0.5386	3	0.5432	3	NA	0.9949
Q12174	0.4495	1	0	0	0	0	0.0367	0	NA	0.1123
P0CX14	0.2873	3	0.1157	1	0.1205	1	0.142	1	NA	0.9126
P32338	0.3667	1	0.1533	0	0.1633	0	0.17	0	TRU	0.5094
Q12224	0.8861	1	0.8683	2	0.8447	2	0.821	2	TRU	0.9995
P36516	0.3846	1	0.0026	0	0	0	0.0205	0	NA	0.1423
Q04307	0.1182	0	0	0	0	0	0.0545	0	NA	0.1222
P38192	0.0097	0	0	0	0	0	0	0	NA	0.0992
P32832	0.4851	2	0.3678	1	0.2874	1	0.3149	1	NA	0.6301
P36123	0.546	4	0.4259	3	0.4085	3	0.4114	3	NA	0.8925
P52871	0.5568	1	0.2386	0	0.5114	1	0.5114	1	NA	0.7533
Q12368	0.5155	1	0.0052	0	0	0	0.1289	0	NA	0.1882
P22224	0.2275	2	0.0692	0	0	0	0.0495	0	NA	0.1682
P39990	0.1984	0	0	0	0	0	0.0635	0	NA	0.1153
Q06134	0.3753	3	0.1363	0	0.1698	0	0.1845	0	NA	0.4768
P53540	0.1998	2	0.0083	0	0	0	0.0236	0	NA	0.1405
P40031	0.1888	0	0	0	0	0	0.1119	0	NA	0.3694
P06843	1	1	0.8769	2	0.961	3	0.8829	2	NA	0.9975
Q3E784	0.8588	1	0.4941	1	0.5059	1	0.4824	1	NA	0.701
P32356	0.2503	1	0.1119	0	0.1305	1	0.1385	1	NA	0.2578
P0CG63	0.3438	0	0.1181	0	0	0	0.0236	0	NA	0.1617
P48836	0.8596	1	0.0702	0	0	0	0.1667	0	NA	0.3111
P38358	0.0654	0	0.0021	0	0	0	0	0	NA	0.1338
Q06385	0.2754	1	0.1159	0	0.1275	1	0.142	1	NA	0.2005
Q02725	0.3952	3	0.1485	1	0.1389	1	0.1617	1	NA	0.4094
Q96VG8	0.2927	0	0	0	0	0	0.3659	0	NA	0.0248
Q3E819	0.4921	0	0	0	0	0	0.1587	0	NA	0.2187
Q96VG6	0.0341	0	0	0	0	0	0	0	NA	0.0976
Q8TGM3	0.3667	0	0	0	0	0	0.2667	0	NA	0.0248
P36074	0.2072	0	0	0	0	0	0	0	NA	0.1604
Q3E771	0.1071	0	0	0	0	0	0	0	NA	0.1058
P0C261	0.1163	0	0	0	0	0	0	0	NA	0.131
P0CF34	0.2882	4	0.1126	1	0.1307	3	0.1469	2	NA	0.3669
Q07978	0.5538	1	0.0054	0	0	0	0	0	NA	0.194
P36087	0.4083	1	0.0828	0	0	0	0.142	0	NA	0.292
Q6B0R2	0.0859	0	0	0	0	0	0	0	NA	0.1171

Q3E842	0.7738	1	0.1548	0	0	0	0.6667	1	NA	0.9967
P53325	0.1103	0	0	0	0	0	0	0	NA	0.1185
Q8TGM8	1	1	0.1	0	0	0	1	1	NA	0.0248
Q12336	0.3512	0	0.0357	0	0	0	0.0833	0	NA	0.3172
Q06325	0.3624	2	0.0554	0	0	0	0.1074	0	NA	0.9029
P0CX58	0.6432	2	0.5864	3	0.5545	3	0.5614	3	NA	0.9955
P43599	0.146	0	0.0055	0	0	0	0	0	NA	0.1505
Q8TGN5	0.1522	0	0	0	0	0	0	0	NA	0.0248
Q3E7A3	1	1	0.027	0	0	0	0.4595	0	NA	0.8853
P53845	0.3885	1	0.172	1	0	0	0.1879	1	NA	0.4239
P40168	0.9272	3	0.908	2	0.8161	3	0.7969	3	NA	0.997
Q08234	0.1762	0	0.0371	0	0	0	0.0386	0	NA	0.1589
P26725	0.1589	1	0	0	0	0	0.0257	0	NA	0.1176
P0CX35	0.2337	0	0.0421	0	0	0	0.0881	0	NA	0.1483
P46654	0.2103	1	0.1548	1	0.1825	1	0.1825	1	NA	0.1793
P11655	0.1868	0	0.0255	0	0	0	0.0276	0	NA	0.179
P22214	0.4299	1	0	0	0	0	0.0234	0	NA	0.1446
P53313	0.5502	4	0.3768	3	0.3168	3	0.3181	3	NA	0.5923
P53189	0.5424	2	0.3911	1	0.3432	1	0.3856	1	NA	0.947
P53541	0.2631	1	0.0016	0	0	0	0	0	NA	0.1471
P17122	0.1162	0	0	0	0	0	0.0202	0	NA	0.1324
P14906	0.3047	3	0.1026	1	0.1026	1	0.1041	1	NA	0.4075
Q06411	0.274	2	0.1059	0	0	0	0.1017	0	NA	0.3385
P53538	0.4029	1	0.0049	0	0	0	0.0631	0	NA	0.1717
P11972	0.3238	1	0.1375	1	0.1261	1	0.1347	1	NA	0.359
Q07478	0.213	1	0.1345	1	0.1413	1	0.1457	1	NA	0.2467
Q04673	0.3232	1	0.1887	1	0.167	1	0.1844	1	NA	0.1889
Q07904	0.1989	1	0.0191	0	0	0	0.0727	0	NA	0.1943
P32613	0.1579	0	0	0	0	0	0	0	NA	0.1304
O13539	0.4751	2	0.069	0	0	0	0.092	0	NA	0.1341
P41338	0.1809	1	0.0678	0	0	0	0.0653	0	NA	0.1585
Q12308	0.1259	0	0	0	0	0	0.0136	0	NA	0.1597
P33296	0.42	1	0.288	0	0.208	0	0.256	0	NA	0.8474
P47108	0.1405	0	0.0017	0	0	0	0.0162	0	NA	0.1281
P36137	0.2415	0	0.0564	0	0	0	0.1038	0	NA	0.207
P22543	0.2411	2	0.064	0	0	0	0.0594	0	NA	0.2285
Q99385	0.1509	0	0.0097	0	0	0	0.0438	0	NA	0.1231
P40151	0.46	4	0.1193	0	0	0	0.1175	1	NA	0.3266
Q12024	0.2783	2	0.087	0	0	0	0.0826	0	NA	0.1824
P35181	0.1859	0	0	0	0	0	0.0577	0	NA	0.1014
Q07879	0.1018	0	0	0	0	0	0	0	NA	0.0995
P53731	0.2047	0	0.0468	0	0	0	0.0731	0	NA	0.169
Q12067	0.1374	0	0	0	0	0	0.0032	0	NA	0.1331
Q03153	0.3088	3	0.0735	0	0	0	0.0817	0	NA	0.2408
P41696	0.8446	2	0.8184	4	0.7396	4	0.721	6	TRU	0.9998
P15703	0.2204	0	0.0032	0	0	0	0.0735	0	NA	0.1315
P53934	0.105	0	0.0175	0	0	0	0.04	0	NA	0.1454
P09798	0.4381	3	0.2786	4	0.2702	3	0.2655	4	NA	0.6429
P32943	0.3132	1	0.1816	1	0.1605	1	0.15	1	NA	0.152
P38323	0.4308	3	0.0923	0	0	0	0.1038	0	NA	0.4266
Q03981	0.216	0	0	0	0	0	0	0	NA	0.1137
P32493	0.112	0	0	0	0	0	0.0174	0	NA	0.1573

P32454	0.1429	0	0.0084	0	0	0	0.0063	0	NA	0.1147
P38316	0.6667	1	0.414	2	0.4086	2	0.4247	2	NA	0.8704
P40458	0.5595	3	0.3006	1	0.2552	2	0.2741	2	NA	0.6682
P38862	0.1333	0	0.0111	0	0	0	0.0444	0	NA	0.1349
P36107	0.2145	1	0	0	0	0	0.1047	1	NA	0.2571
P53226	0.7112	2	0.6051	5	0.5393	3	0.521	4	NA	0.9874
P26448	0.1765	0	0.0065	0	0	0	0.0458	0	NA	0.1375
P38813	0.1642	1	0.0119	0	0	0	0.0537	0	NA	0.1184
Q06150	0.1035	0	0	0	0	0	0.0158	0	NA	0.1309
P80235	0.2969	3	0.0946	0	0	0	0.1223	0	NA	0.4498
P21657	0.5278	2	0.4165	3	0.4309	3	0.399	4	TRU	0.9053
P46681	0.1925	0	0.0113	0	0	0	0.0208	0	NA	0.1529
P32471	0.4951	1	0.199	0	0	0	0.199	0	NA	0.3866
P32528	0.1635	0	0.0218	0	0	0	0.0229	0	NA	0.2516
Q06673	0.3925	7	0.1695	2	0.19	3	0.2182	3	NA	0.9686
Q02202	0.1008	0	0.0027	0	0	0	0	0	NA	0.1294
Q02647	0.3043	0	0	0	0	0	0.1087	0	NA	0.1446
P38071	0.2526	0	0.0342	0	0	0	0.0237	0	NA	0.1529
P32178	0.1602	0	0.0117	0	0	0	0.0586	0	NA	0.2459
P53332	0.1934	1	0.023	0	0	0	0.0689	0	NA	0.146
P53865	0.5972	3	0.3098	2	0.222	1	0.2289	1	NA	0.6523
Q3E846	1	1	0.2981	0	0.3365	0	0.3462	0	NA	0.5158
Q06139	0.5149	1	0.198	0	0	0	0.3861	0	NA	0.2313
Q08058	0.0676	0	0.0097	0	0	0	0.0435	0	NA	0.1269
P53318	0.1232	0	0.0042	0	0	0	0.0251	0	NA	0.1537
Q06705	0.277	2	0.076	0	0	0	0.1348	0	NA	0.3434
Q07560	0.1449	0	0.0106	0	0	0	0.0141	0	NA	0.1124
Q08226	0.1923	2	0.0522	0	0.069	1	0.0815	1	NA	0.2169
P38877	0.5338	1	0.1429	0	0	0	0.2256	0	NA	0.205
P36162	0.7368	2	0.4662	1	0.5188	1	0.4361	1	NA	0.795
P25616	0.0915	0	0	0	0	0	0.0095	0	NA	0.1216
P25355	0.1758	1	0.0231	0	0.0426	0	0.0426	0	NA	0.1394
P40442	0.7678	3	0.2412	1	0.2141	1	0.3899	1	NA	1
P53267	0.7318	3	0.6356	3	0.5394	3	0.5306	3	NA	0.972
Q08232	0.295	1	0.1863	1	0.2267	1	0.2391	1	NA	0.4564
P53168	0.7287	2	0.5223	1	0.4696	1	0.4939	1	NA	0.9675
P25358	0.1268	0	0.0375	0	0	0	0.0663	0	NA	0.0962
P53861	0.7018	1	0.5515	1	0.5066	1	0.4565	2	NA	0.8673
P47163	0.1298	0	0	0	0	0	0.0177	0	NA	0.1155
Q12099	0.1855	1	0.0075	0	0	0	0.0326	0	NA	0.1151
Q12480	0.2791	1	0.0029	0	0	0	0.0233	0	NA	0.1674
P36001	0.086	0	0.0116	0	0	0	0.0209	0	NA	0.1186
P53954	0.1022	0	0.0036	0	0	0	0.031	0	NA	0.1149
Q04412	0.4066	2	0.139	1	0.1846	1	0.2116	1	NA	0.6619
P25612	0.2672	1	0.0165	0	0	0	0.0523	0	NA	0.1853
Q08223	0.3316	2	0.1468	0	0	0	0.162	0	NA	0.201
P04710	0.1165	0	0.0194	0	0	0	0	0	NA	0.1507
P25376	0.2512	1	0.1643	2	0.1801	2	0.1517	2	NA	0.2776
P32858	0.0593	0	0	0	0	0	0	0	NA	0.1395
Q06822	0.149	1	0.0293	0	0	0	0.0609	0	NA	0.1613
P25627	0.4145	1	0.1927	0	0.1564	0	0.1745	0	NA	0.157
Q08235	0.4536	2	0.1271	0	0	0	0.1512	0	NA	0.2095

P47041	0.5709	2	0.302	1	0.1676	1	0.1823	1	NA	0.5299
Q12291	0.1041	0	0.0055	0	0	0	0.0274	0	NA	0.1225
P35817	0.7391	4	0.5481	5	0.5379	5	0.5306	7	NA	0.9984
P25385	0.4672	1	0.2992	1	0	0	0.1885	0	NA	0.5015
Q07442	0.7194	5	0.3777	3	0.4169	4	0.4169	4	NA	0.988
P38041	0.7602	3	0.602	5	0.5694	6	0.5582	7	NA	0.9989
P47158	0.1871	1	0.0704	0	0	0	0.1066	0	NA	0.3052
P21269	0.1081	0	0.0238	0	0	0	0.0165	0	NA	0.1195
Q2V2P8	0.5506	1	0.1124	0	0	0	0.2472	0	NA	0.2724
P38307	0.0664	0	0	0	0	0	0	0	NA	0.1069
Q99288	0.2635	0	0.0719	0	0	0	0.0868	0	NA	0.1853
P13663	0.1342	0	0.0466	0	0	0	0.0795	0	NA	0.2988
Q04110	0.7583	1	0.5331	3	0.4868	2	0.4868	2	NA	0.8008
Q06337	0.6303	4	0.4511	3	0.4084	3	0.3737	7	NA	0.9108
P32502	0.273	1	0.0735	0	0.1076	0	0.1129	0	NA	0.4124
P02992	0.1991	0	0.0572	0	0	0	0.016	0	NA	0.1599
P38732	0.0889	0	0	0	0	0	0	0	NA	0.1319
P53235	0.3738	2	0.2741	1	0.2523	1	0.2586	1	NA	0.4188
P32604	0.2301	1	0.0265	0	0	0	0.0708	0	NA	0.2293
P38333	0.4803	1	0.3333	2	0.3354	3	0.3188	2	NA	0.7871
Q05359	0.2009	0	0	0	0	0	0	0	NA	0.1264
P32629	0.374	1	0.21	1	0.176	1	0.17	1	NA	0.2298
Q07528	0.6734	5	0.35	1	0.275	2	0.2609	2	NA	0.8296
Q05789	0.6504	2	0.1726	0	0	0	0.1106	0	NA	0.3203
Q12092	0.6197	1	0.3944	1	0.3239	1	0.3239	1	NA	0.7612
P14843	0.2459	1	0.0703	0	0	0	0.0757	0	NA	0.1559
P40527	0.2676	1	0.0947	2	0.073	1	0.0799	0	NA	0.2584
P53090	0.23	0	0.078	0	0	0	0.132	0	NA	0.4243
P38111	0.1318	0	0.003	0	0	0	0.0068	0	NA	0.175
Q06631	0.7341	5	0.4738	3	0.3521	1	0.3652	2	NA	0.6833
P39960	0.4121	6	0.2349	5	0.2247	5	0.2335	5	NA	0.9916
P33306	0.9671	2	0.8813	9	0.611	5	0.6381	4	NA	0.9999
P53615	0.2081	0	0.0136	0	0	0	0.0724	0	NA	0.1595
P32796	0.2015	0	0.0119	0	0	0	0.0224	0	NA	0.2241
P07245	0.1681	2	0.0814	0	0	0	0.0497	0	NA	0.2326
P47179	0.8786	2	0.4358	1	0.3945	2	0.4505	2	NA	1
P40077	0.2845	2	0.0262	0	0	0	0.0873	1	NA	0.2935
P38167	0.6697	6	0.4709	5	0.3966	4	0.3948	6	NA	0.9925
P32497	0.3805	3	0.1613	1	0.1761	1	0.1921	1	NA	0.5321
Q12050	0.4223	3	0.0986	0	0	0	0.0506	0	NA	0.1817
Q02884	0.4868	3	0.3706	1	0.3158	3	0.3333	3	NA	0.7814
P53971	0.6953	7	0.0653	0	0	0	0.0829	0	NA	0.3178
Q03769	0.6642	1	0.4793	1	0.5182	1	0.5036	1	NA	0.9681
P23542	0.0742	0	0	0	0	0	0	0	NA	0.1326
Q07988	0.4018	1	0.0804	0	0	0	0.1295	0	NA	0.4446
Q12089	0.3614	2	0.0429	0	0	0	0.0363	0	NA	0.1537
Q03862	0.2546	0	0.0472	0	0	0	0.0438	0	NA	0.3648
Q12233	0.1043	0	0	0	0	0	0	0	NA	0.1576
P13587	0.2301	2	0.0376	0	0.0596	1	0.0587	0	NA	0.3879
P05626	0.6107	2	0.0328	0	0	0	0.0861	0	NA	0.1884
P22035	0.7928	3	0.7078	5	0.6153	5	0.5931	8	TRU	0.9962
P41832	0.6539	5	0.49	8	0.4347	6	0.425	6	NA	0.9999

P48558	0.1919	0	0.0168	0	0	0	0.1582	0	NA	0.2923
P11709	0.8227	2	0.6273	1	0.3705	1	0.3795	1	NA	0.884
Q01532	0.1677	0	0	0	0	0	0.0373	0	NA	0.1639
P40450	0.5215	6	0.2378	2	0.2	3	0.2124	2	NA	0.9111
Q3E793	0.3091	0	0.1091	0	0	0	0.2182	0	NA	0.6285
P22804	0.662	1	0.1901	0	0.4648	1	0.4437	1	NA	0.8773
P53838	0.1615	1	0.0503	0	0.0903	1	0.0972	1	NA	0.155
P07258	0.1436	0	0.0024	0	0	0	0.0195	0	NA	0.1803
P39113	0.6148	6	0.395	6	0.3182	7	0.3252	6	TRU	0.9804
P34237	0.8704	2	0.4065	2	0	0	0.2518	0	NA	0.9808
P25342	0.1522	0	0.0186	0	0	0	0.0745	0	NA	0.1372
P39525	0.2014	1	0	0	0	0	0.0204	0	NA	0.1651
Q06218	0.3653	2	0.2054	2	0.1919	2	0.1869	2	NA	0.3131
P38773	0.1951	0	0.0203	0	0	0	0.0366	0	NA	0.1225
P38213	0.803	1	0.625	6	0.5707	4	0.5666	4	NA	0.9932
Q04868	0.1758	0	0.0073	0	0	0	0.0842	0	NA	0.2653
Q00362	0.2966	1	0.2186	1	0.2053	1	0.2053	1	NA	0.4021
P40563	1	1	0.9867	3	0.975	3	0.972	3	NA	0.9987
P53930	0.469	1	0.1416	0	0	0	0.1814	0	NA	0.4856
P38884	0.433	2	0.0374	0	0	0	0.053	0	NA	0.1488
P22136	0.1638	1	0.0052	0	0	0	0.0103	0	NA	0.2336
P07249	0.7288	1	0.7458	1	0.5932	1	0.5876	1	TRU	0.9897
Q12292	0.4175	3	0.0534	0	0	0	0.0437	0	NA	0.3167
P32660	0.2903	3	0.1961	2	0.1445	1	0.154	1	NA	0.3525
P32449	0.2216	0	0.0892	0	0	0	0.073	0	NA	0.2749
Q08004	0.6145	1	0.4096	0	0	0	0.1928	0	NA	0.2534
Q06604	0.9688	2	0.8507	4	0.6701	3	0.684	5	NA	0.9913
P35727	0.7168	1	0.0619	0	0	0	0.1327	0	NA	0.1243
P36038	0.2251	0	0.0185	0	0	0	0.0849	0	NA	0.38
P40558	0.2628	1	0.0922	0	0	0	0.1024	0	NA	0.1758
Q06819	0.0769	0	0	0	0	0	0.0559	0	NA	0.1103
P29547	0.2578	1	0.0916	1	0.1325	1	0.1422	1	NA	0.2632
P32324	0.1615	1	0.0321	0	0	0	0.0273	0	NA	0.16
P25039	0.2733	3	0.0355	0	0	0	0.0263	0	NA	0.2171
P40023	0.9586	1	0.9724	2	0.9379	2	0.8483	2	NA	0.9728
P42935	0.1269	1	0.0495	1	0.0533	1	0.0723	1	NA	0.2095
Q05874	0.2567	1	0.0651	0	0.1341	1	0.1341	1	NA	0.1485
P53974	0.5329	1	0.4389	3	0.4342	1	0.3762	2	NA	0.8562
P28777	0.4149	2	0.2952	1	0.2128	0	0.242	0	NA	0.6483
Q12380	0.1531	1	0	0	0	0	0.0442	0	NA	0.1085
P53895	0.4325	1	0.2872	1	0.2734	1	0.2664	0	NA	0.3517
Q06541	0.0374	0	0	0	0	0	0	0	NA	0.1057
Q12421	0.6224	2	0.3112	0	0.301	0	0.3367	0	NA	0.7175
Q03048	0.3217	0	0	0	0	0	0.0909	0	NA	0.1297
P08432	0.1502	1	0.0064	0	0	0	0.0386	0	NA	0.1371
O13577	1	1	0.8045	1	0.9248	2	0.8421	2	NA	0.8935
P47178	0.5638	1	0.2886	1	0.2416	1	0.2953	1	NA	0.8337
P33327	0.1786	1	0.0119	0	0	0	0.0229	0	NA	0.1816
P33413	0.1592	1	0.0231	0	0	0	0.0503	0	NA	0.1506
P40217	0.2133	0	0.0375	0	0	0	0.0461	0	NA	0.2533
Q05050	0.8648	5	0.605	5	0.3559	3	0.382	3	NA	0.9836
P39013	0.4441	1	0.2779	1	0.2092	1	0.2006	1	NA	0.3018

P38874	0.2718	1	0.1165	0	0	0	0.1392	0	NA	0.2168
P32771	0.0466	0	0.0078	0	0	0	0.0311	0	NA	0.1999
Q12048	0.8897	1	0.1324	0	0	0	0.1912	0	NA	0.4017
Q06408	0.1795	1	0.0157	0	0	0	0.0283	0	NA	0.125
P47082	0.3422	1	0.1096	0	0	0	0.0648	0	NA	0.1976
P31384	0.4421	2	0.3094	3	0.2413	3	0.233	3	NA	0.5523
Q05809	0.5312	0	0.3229	0	0	0	0.4896	0	NA	0.3303
P20486	0.4267	1	0.2667	1	0.3	1	0.3067	1	NA	0.2415
P36064	0.7027	2	0.1532	0	0	0	0.2613	0	NA	0.2125
Q12289	0.2294	1	0.0581	0	0.104	0	0.1284	1	NA	0.229
P41810	0.2487	3	0.038	0	0	0	0.0637	0	NA	0.2624
P0CX12	0.1372	0	0	0	0	0	0.0422	0	NA	0.1168
Q05779	0.2423	1	0	0	0	0	0	0	NA	0.2008
P32328	0.3014	1	0.1082	1	0.1436	1	0.1826	1	NA	0.2632
Q12517	0.29	1	0.1255	0	0.1818	1	0.2121	1	NA	0.298
Q06063	0.1771	0	0.0163	0	0	0	0.0436	0	NA	0.121
Q08729	0.7023	3	0.2279	0	0	0	0.1744	0	NA	0.3675
P39995	0.4516	1	0.1792	1	0.1649	1	0.1505	1	NA	0.4425
Q06011	0.5	1	0.4107	0	0.2946	1	0.4286	1	NA	0.2725
P32525	0.4307	1	0.2821	1	0.2938	1	0.2955	2	NA	0.5471
P22768	0.2405	0	0.0643	0	0	0	0.0643	0	NA	0.1426
P81451	0.5735	1	0.2059	0	0	0	0.2941	0	NA	0.1561
P18496	0.2939	1	0.0323	0	0	0	0.0824	0	NA	0.1636
P47068	0.7528	2	0.726	2	0.6975	3	0.6733	4	NA	0.997
P39713	0.1894	1	0.1103	1	0.0935	1	0.0959	1	NA	0.1251
Q3E835	0.1889	0	0.0778	0	0	0	0.1778	0	NA	0.1116
P48562	0.5368	3	0.4287	1	0.3967	2	0.4311	2	NA	0.9973
P32378	0.164	1	0.0269	0	0	0	0.0618	0	NA	0.2075
Q12309	0.2169	0	0.0015	0	0	0	0.0335	0	NA	0.1136
P00890	0.3027	0	0.0292	0	0	0	0.0626	0	NA	0.3608
P53137	0.4647	2	0.3093	2	0.2708	3	0.2933	2	NA	0.7743
P00175	0.2623	2	0.0914	0	0	0	0.0863	0	NA	0.2624
P53769	0.6139	1	0.4208	1	0.3861	1	0.3977	1	NA	0.7285
Q06469	0.377	2	0.0476	0	0	0	0.0635	0	NA	0.137
P04819	0.3179	1	0.1682	2	0.1788	2	0.1801	2	NA	0.4504
Q03798	0.3451	1	0.0353	0	0	0	0.0941	0	NA	0.1786
P38770	0.6348	2	0.4501	2	0.482	3	0.4395	3	NA	0.9747
Q06333	0.2377	0	0.123	0	0	0	0.2459	0	NA	0.1509
P38843	0.057	0	0	0	0	0	0	0	NA	0.1229
P35190	0.5442	3	0.1836	0	0	0	0.1991	0	NA	0.9419
P27895	0.648	6	0.252	3	0.166	2	0.166	3	NA	0.3578
Q04659	0.6688	2	0.2965	0	0	0	0.2555	0	NA	0.3632
P52924	0.139	0	0	0	0	0	0.0321	0	NA	0.123
Q06440	0.4777	1	0.3702	1	0.3579	1	0.3564	1	NA	0.6256
P23285	0.2195	0	0.0293	0	0	0	0.0683	0	NA	0.2083
P13483	0.9516	2	0.9194	3	0.7702	2	0.75	2	NA	0.998
P53063	0.2222	0	0.0026	0	0	0	0.0258	0	NA	0.1421
P38281	0.2722	1	0.0253	0	0	0	0.0665	0	NA	0.1554
P24813	0.6333	1	0.3692	2	0	0	0.2714	2	TRU	0.6358
P06787	0.5782	1	0.0544	0	0	0	0.068	0	NA	0.1392
P38910	0.1226	0	0	0	0	0	0.0849	0	NA	0.1556
Q12748	0.1214	0	0	0	0	0	0	0	NA	0.1198

P40986	0.2648	2	0.0631	0	0	0	0.1283	1	NA	0.2634
P39110	0.1099	0	0	0	0	0	0.0314	0	NA	0.1332
P47146	0.1626	0	0.0081	0	0	0	0.0569	0	NA	0.1543
P33304	0.7871	4	0.3597	1	0	0	0.279	1	NA	0.9485
P38080	0.713	1	0.6399	2	0.6011	3	0.5542	5	NA	0.9999
P36154	0.2903	0	0.0129	0	0	0	0.0968	0	NA	0.1325
P18239	0.1226	0	0.0409	0	0	0	0.0755	0	NA	0.138
P11076	0.1105	0	0	0	0	0	0	0	NA	0.1171
P40532	0.1014	0	0	0	0	0	0	0	NA	0.1114
P38153	0.2443	1	0.0248	0	0	0	0.0145	0	NA	0.1208
P35193	0.4964	3	0.2169	1	0.159	1	0.1928	1	NA	0.3006
Q06671	0.7373	3	0.245	1	0.1987	1	0.1965	1	NA	0.4843
P53867	0.1964	0	0.0243	0	0	0	0.0607	0	NA	0.2129
Q12186	0.6954	3	0.5084	2	0.4727	1	0.4874	1	NA	0.9341
P40501	0.1918	1	0.0571	0	0	0	0.1204	0	NA	0.2842
P39724	0.322	0	0.161	0	0	0	0.1525	0	NA	0.2017
P50080	0.137	1	0.0163	0	0.0669	1	0.0832	1	NA	0.1151
P12962	0.7391	1	0.6832	2	0.6335	1	0.6273	2	NA	0.9419
Q03941	0.2116	1	0.0581	0	0	0	0.1162	0	NA	0.1513
P05374	0.1634	2	0.0092	0	0	0	0.0437	0	NA	0.1309
P32463	0.336	0	0.016	0	0	0	0.088	0	NA	0.1231
Q02486	0.5956	2	0.0492	0	0	0	0.0765	0	NA	0.3517
Q04894	0.1917	1	0.0028	0	0	0	0.0444	0	NA	0.2602
P85052	0.1046	0	0	0	0	0	0.1373	0	NA	0.135
P39969	0.7731	4	0.6413	7	0.5567	6	0.5606	6	NA	0.9996
Q02948	0.5583	3	0.2298	1	0.1849	1	0.2047	1	NA	0.5683
P14066	0.2402	0	0.0087	0	0	0	0.0306	0	NA	0.1976
Q02732	0.3659	2	0.1572	1	0.1138	1	0.122	0	NA	0.1741
P20438	0.4587	2	0.2752	2	0.222	1	0.244	1	NA	0.7148
Q3E823	0.5735	1	0.0588	0	0	0	0.5294	0	NA	0.3264
Q07622	0.4639	1	0.3451	2	0.2713	2	0.297	1	NA	0.441
P03875	0.3165	2	0.0707	0	0.054	0	0.0755	1	NA	0.2731
Q04689	0.1704	0	0.0611	0	0	0	0.0514	0	NA	0.1359
Q04516	0.0865	0	0	0	0	0	0.0032	0	NA	0.1359
P18544	0.0922	0	0.0024	0	0	0	0.0236	0	NA	0.1656
P53244	0.4198	2	0.1689	0	0.1843	0	0.2014	1	NA	0.8393
P53296	0.1551	0	0	0	0	0	0.0131	0	NA	0.1372
Q06071	0.2049	0	0.041	0	0	0	0	0	NA	0.1311
P50084	0.8029	1	0.3942	0	0	0	0.3942	0	NA	0.4646
P00729	0.1071	0	0	0	0	0	0.0132	0	NA	0.1664
Q06703	0.2212	1	0	0	0	0	0.016	0	NA	0.1339
P24868	0.3928	2	0.1592	1	0	0	0.1423	0	NA	0.175
P38903	0.498	2	0.4201	1	0.4029	2	0.4095	2	NA	0.8963
Q07716	0.1205	0	0	0	0	0	0	0	NA	0.1295
P25377	0.1108	0	0	0	0	0	0.0222	0	NA	0.1497
P47015	0.5112	2	0.2135	1	0	0	0.1685	0	NA	0.1987
P53068	0.16	0	0.032	0	0	0	0.024	0	NA	0.1346
Q05933	0.1236	0	0	0	0	0	0.0674	0	NA	0.147
Q12500	0.5118	1	0.394	1	0.3914	1	0.394	2	NA	0.5787
P07251	0.2862	3	0.022	0	0	0	0.0294	0	NA	0.2846
P16474	0.3226	3	0.1232	0	0.0469	1	0.0616	1	NA	0.2536
Q03654	0.6831	3	0.461	3	0.2661	1	0.2678	1	NA	0.9104

Q12102	0.3434	4	0.1711	2	0.0943	1	0.1234	1	NA	0.4653
P32468	0.4275	3	0.2039	0	0	0	0.1646	0	NA	0.5221
P38305	0.6341	1	0.8618	1	0.6423	1	0.748	1	NA	0.6105
P07248	0.4868	6	0.2041	3	0	0	0.1413	1	TRU	0.8657
P38885	0.3774	1	0.0903	0	0	0	0.0903	0	NA	0.1921
P38266	0.9968	1	0.9778	1	0.9968	1	0.9652	2	NA	0.9994
P48360	0.2049	1	0.0142	0	0	0	0.0345	0	NA	0.1583
P38328	0.2188	1	0.0495	0	0	0	0.099	0	NA	0.2327
P53323	0.2912	1	0	0	0	0	0.0498	0	NA	0.138
Q05611	0.4681	1	0.0426	0	0	0	0.1064	0	NA	0.249
P53286	0.7024	2	0.4707	1	0.4732	1	0.4829	2	NA	0.8822
P37366	0.4478	2	0.2595	2	0.2774	2	0.2799	2	NA	0.4797
P07267	0.1383	0	0.0099	0	0	0	0.0148	0	NA	0.1543
P38042	0.3522	1	0.2427	1	0.2586	1	0.2704	2	NA	0.5723
P32375	0.1804	1	0.0565	0	0	0	0.0674	0	NA	0.3176
Q03673	0.4596	1	0.0354	0	0	0	0.0707	0	NA	0.128
P38856	0.5447	3	0.4568	2	0.3658	1	0.2873	1	NA	0.9712
Q08702	0.2535	0	0	0	0	0	0.0507	0	NA	0.1131
P49090	0.1311	0	0.035	0	0	0	0.0245	0	NA	0.2153
P48361	0.6161	5	0.4834	6	0.3866	7	0.3953	4	NA	0.9997
P32381	0.1586	0	0.0435	0	0	0	0.0639	0	NA	0.2983
P40416	0.1232	1	0.0029	0	0	0	0.0116	0	NA	0.1165
P41695	0.4564	5	0.3232	3	0.2821	4	0.284	3	NA	0.8534
P53841	0.2214	0	0.0153	0	0	0	0.0916	0	NA	0.1475
P36106	0.5337	3	0.298	3	0.2677	2	0.2744	2	NA	0.4311
P39714	0.1126	0	0.0131	0	0	0	0.0288	0	NA	0.1261
Q12365	0.8494	3	0.3714	2	0.2727	1	0.3221	1	NA	0.5015
Q03630	0.2767	0	0.0189	0	0	0	0.0503	0	NA	0.175
P50277	0.1083	0	0.0042	0	0	0	0.025	0	NA	0.1279
P17555	0.4563	2	0.289	3	0.3213	3	0.2871	3	NA	0.7628
Q03702	0.2946	1	0.0255	0	0	0	0.0623	0	NA	0.1494
P53868	0.0703	0	0	0	0	0	0.0126	0	NA	0.1084
P0CD91	0.2459	1	0.118	0	0.1869	1	0.1902	1	NA	0.4162
P18634	0.3417	2	0.0425	0	0	0	0.0753	0	NA	0.3116
P40013	0.4971	2	0.2936	2	0.3227	2	0.3169	2	NA	0.9088
P38346	0.5376	1	0.3982	2	0.3615	2	0.3468	2	NA	0.9485
P32839	0.1908	1	0.0702	0	0	0	0.0614	0	NA	0.1517
P25558	0.5984	3	0.423	5	0.3686	4	0.3674	6	NA	0.9843
Q01389	0.7253	5	0.5507	8	0.5257	8	0.5318	9	NA	0.9997
P25656	0.2583	1	0.0665	0	0	0	0.0793	0	NA	0.2432
Q12114	0.6483	1	0.5976	1	0.6006	1	0.6006	1	NA	0.9929
P13365	0.4672	2	0.1448	0	0.1414	1	0.1931	2	NA	0.5721
P16522	0.1885	1	0.0383	0	0	0	0.0623	0	NA	0.1355
P15274	0.3543	2	0.2469	1	0.2025	2	0.179	2	NA	0.4556
P46672	0.5027	2	0.2739	1	0.2527	1	0.2553	1	NA	0.3407
P32451	0.3947	2	0.0293	0	0	0	0.0267	0	NA	0.2036
P32639	0.2367	4	0.0804	2	0.0513	0	0.0601	0	NA	0.2803
P53741	0.668	2	0.5825	2	0.567	2	0.5864	3	NA	0.9896
P39011	0.2085	1	0.019	0	0	0	0.019	0	NA	0.1269
Q08347	0.1331	1	0.0046	0	0	0	0.0418	0	NA	0.1786
P27825	0.2948	2	0.1275	1	0	0	0.1036	0	NA	0.2108
P04817	0.1814	1	0.1186	1	0.1	1	0.1102	0	NA	0.1454

P0C155	0.2186	1	0.0413	0	0	0	0.0929	0	NA	0.3016
P40047	0.0923	0	0.0038	0	0	0	0.0269	0	NA	0.1307
P52892	0.1854	0	0.0611	0	0	0	0.0454	0	NA	0.1619
P25335	0.1545	0	0.0496	0	0	0	0.0671	0	NA	0.1583
P38278	0.3591	1	0.0772	0	0	0	0.089	0	NA	0.3422
P47134	0.7013	3	0.499	4	0.3553	4	0.3753	4	NA	0.9225
Q08965	0.5545	5	0.4311	4	0.3702	4	0.3626	5	NA	0.7713
Q07457	0.75	4	0.1771	1	0.1343	1	0.1443	1	NA	0.4414
P25356	0.1218	1	0.0083	0	0	0	0.0152	0	NA	0.2004
Q04199	0.2201	1	0.1197	0	0	0	0.1047	0	NA	0.1573
P43569	0.1384	0	0.0277	0	0	0	0.0554	0	NA	0.1317
P36130	0.3204	1	0.0902	0	0	0	0.0855	0	NA	0.3535
P03874	0.1603	0	0.0032	0	0	0	0.0206	0	NA	0.1281
Q3E829	0.5	0	0.125	0	0	0	0.1875	0	NA	0.6281
P47112	0.2566	1	0.0596	0	0	0	0.0712	0	NA	0.414
Q3E7B2	0.2941	0	0	0	0	0	0.2	0	NA	0.0999
P38794	0.4585	1	0.2314	1	0.2795	1	0.3188	1	NA	0.6305
Q03050	0.15	0	0	0	0	0	0.025	0	NA	0.0872
P0CE85	0.1613	0	0	0	0	0	0.0403	0	NA	0.0997
P0CE89	0.175	0	0	0	0	0	0.025	0	NA	0.0884
Q02969	0.236	1	0.1548	1	0.1853	1	0.198	1	NA	0.3096
P06197	0.1818	0	0.0364	0	0	0	0	0	NA	0.1182
Q03674	0.3484	2	0.068	0	0.0637	1	0.1048	1	NA	0.8899
P38218	0.2034	0	0	0	0	0	0.0508	0	NA	0.1339
P34222	0.3269	1	0.2115	1	0.2404	1	0.2981	1	NA	0.492
P06778	0.7261	2	0.6433	3	0.4862	3	0.4904	4	NA	0.896
Q06709	0.6134	2	0.3773	2	0.2477	1	0.2917	1	NA	0.325
P40016	0.2505	2	0.0669	1	0.1128	1	0.1205	1	NA	0.2841
P36121	0.4894	2	0.4078	1	0.3475	1	0.3617	1	NA	0.7265
Q02950	0.4186	2	0.0552	0	0	0	0.061	0	NA	0.2662
P10870	0.4853	2	0.3959	2	0.3575	4	0.3371	4	NA	0.86
P32597	0.5298	5	0.2465	2	0.1678	2	0.1803	2	NA	0.4735
P13099	0.2149	1	0.0808	0	0	0	0.096	0	NA	0.3843
P53118	0.2788	2	0.0774	0	0	0	0.0788	0	NA	0.1817
Q06488	0.5051	4	0.4139	6	0.3746	5	0.3678	3	NA	0.9361
P11792	0.4903	5	0.4223	6	0.3604	4	0.3459	4	NA	0.9884
P25554	0.4595	2	0.2355	0	0.139	0	0.1853	0	NA	0.3487
Q03323	0.7143	1	0.8171	1	0.48	1	0.4971	1	NA	0.737
Q03868	0.3494	3	0.1815	1	0.1582	3	0.1647	2	NA	0.3624
P39929	0.95	1	0.6417	1	0.4292	2	0.3792	1	NA	0.4648
P53135	0.6048	3	0.3593	1	0.2141	1	0.2665	1	NA	0.5263
P09959	0.6015	3	0.3923	3	0.2839	3	0.2765	3	TRU	0.7994
Q08646	0.3585	1	0.0755	0	0	0	0.097	0	NA	0.2192
Q02875	0.7609	2	0.5842	3	0.5147	3	0.4935	3	NA	0.9985
P53228	0.2793	1	0.0991	0	0	0	0.1201	0	NA	0.1811
Q12043	0.3351	1	0.1762	2	0.1682	2	0.1762	2	NA	0.5371
Q03496	0.1014	1	0	0	0	0	0.0063	0	NA	0.113
P35209	0.8021	4	0.6636	6	0.4116	3	0.4169	4	NA	0.7466
Q06010	0.1431	0	0.0282	0	0	0	0.0243	0	NA	0.1955
P22438	0.2191	1	0.0243	0	0	0	0.0678	1	NA	0.1407
P53065	0.235	0	0.005	0	0	0	0.0325	0	NA	0.1193
P32897	0.3514	1	0.1396	0	0	0	0.1396	0	NA	0.3249

Q9URQ3	0.1708	0	0.0093	0	0	0	0.0186	0	NA	0.1378
P34219	0.8362	4	0.779	5	0.539	4	0.5429	3	NA	0.9963
Q07527	0.1156	0	0.0056	0	0	0	0.0063	0	NA	0.1146
P38319	0.2353	1	0.0515	0	0	0	0.0699	0	NA	0.2586
P38816	0.1667	1	0.0029	0	0	0	0.0205	0	NA	0.1368
P43123	0.3291	1	0.0419	0	0	0	0.0629	0	NA	0.219
P38152	0.1973	0	0.0167	0	0	0	0.0435	0	NA	0.2155
P22515	0.2305	2	0.0703	0	0	0	0.084	0	NA	0.2505
P38709	0.0913	0	0	0	0	0	0.0203	0	NA	0.1563
P53726	0.5882	1	0	0	0	0	0.0924	0	NA	0.3076
Q08206	0.866	3	0.7411	5	0.5046	4	0.5085	3	NA	0.9958
P47172	0.2421	1	0	0	0	0	0	0	NA	0.1249
P47073	0.0957	0	0	0	0	0	0	0	NA	0.0881
P47078	0.0833	0	0	0	0	0	0	0	NA	0.1184
P40579	0.1339	0	0.0039	0	0	0	0.0276	0	NA	0.1054
P35691	0.2395	0	0.1198	0	0	0	0.0778	0	NA	0.2693
P53230	0.1766	0	0.013	0	0	0	0.039	0	NA	0.119
P47058	0.144	0	0.032	0	0	0	0.092	0	NA	0.1374
P32643	0.2776	0	0	0	0	0	0.1003	0	NA	0.168
Q12049	0.534	1	0.3085	3	0.2149	2	0.2255	2	NA	0.4784
P54857	0.1902	0	0.0031	0	0	0	0.0429	0	NA	0.1417
Q06339	0.3006	1	0.2247	3	0.2068	2	0.1935	2	NA	0.2641
P38811	0.1461	2	0.0355	1	0.0334	2	0.0385	1	NA	0.2483
P48560	0.7549	1	0.0686	0	0	0	0.0392	0	NA	0.1861
Q06672	0.4244	1	0.2634	1	0.322	1	0.3756	1	NA	0.53
P52491	0.4202	1	0.133	0	0.1862	0	0.2394	0	NA	0.2468
Q04006	0.1285	0	0	0	0	0	0.0503	0	NA	0.1269
P12887	0.4178	1	0.1476	0	0	0	0.1281	0	NA	0.3671
Q04228	0.3596	4	0.1233	0	0	0	0.125	0	NA	0.39
P34247	0.988	2	0.648	0	0.52	0	0.508	1	NA	0.698
P51401	0.1047	0	0	0	0	0	0.0314	0	NA	0.1242
P36521	0.2651	1	0.1285	0	0.1044	0	0.1124	0	NA	0.1632
P32565	0.2095	1	0.0878	1	0.0783	1	0.0825	1	NA	0.2856
P04050	0.3006	3	0.1991	1	0.1489	1	0.1708	1	NA	0.3517
Q12377	0.2097	1	0.0369	0	0	0	0.0783	0	NA	0.1987
P05756	0.2318	0	0.0066	0	0	0	0.1391	0	NA	0.361
P05759	0.5329	0	0.0987	0	0	0	0.0526	0	NA	0.1315
P05755	0.4769	2	0.2359	0	0.2615	1	0.2821	1	NA	0.2225
Q12751	0.2559	1	0.0479	0	0.0428	0	0.0571	0	NA	0.2883
P53289	0.9316	2	0.7452	2	0.6008	1	0.6768	3	NA	0.9923
P43682	0.7113	1	0.1134	0	0	0	0.1753	0	NA	0.1251
Q99287	0.2036	1	0.0155	0	0	0	0.0387	0	NA	0.1356
P20048	0.0482	0	0	0	0	0	0.0077	0	NA	0.118
P40479	0.311	1	0.2105	1	0.2297	1	0.2488	1	NA	0.1682
P39931	0.4444	1	0.2137	0	0	0	0.141	0	NA	0.4129
Q06688	0.219	0	0.0182	0	0	0	0.0766	0	NA	0.1258
Q07351	0.8061	2	0.5061	4	0.4551	3	0.5122	3	NA	0.9981
Q12009	0.4091	2	0.1189	0	0	0	0.1084	0	NA	0.1474
P38228	0.2028	0	0.035	0	0	0	0.021	0	NA	0.1475
P11747	0.6347	1	0.4311	1	0.491	1	0.491	1	NA	0.6548
P32318	0.1534	0	0.0153	0	0	0	0.046	0	NA	0.2217
Q03322	0.808	1	0.5	2	0	0	0.1875	0	NA	0.4685

P35172	0.2397	2	0.1192	1	0.0949	1	0.1115	1	NA	0.2544
Q3E790	0.3	0	0	0	0	0	0.15	0	NA	0.1518
Q03327	0.253	2	0.0956	0	0	0	0.0857	0	NA	0.2132
P38349	0.6239	3	0.4037	1	0.3303	1	0.344	4	NA	0.9108
Q04305	0.3138	1	0.0858	0	0.0994	0	0.0936	0	NA	0.1609
Q07950	0.29	2	0.1561	1	0.1636	1	0.1654	1	NA	0.2204
Q99248	0.6589	3	0.2836	0	0.289	1	0.3507	3	NA	0.9625
P50083	0.087	0	0	0	0	0	0.1014	0	NA	0.1195
P39974	0.215	0	0.028	0	0	0	0	0	NA	0.2155
Q8TGT7	0.1081	0	0	0	0	0	0.0811	0	NA	0.0248
P53288	0.8621	1	0.4384	1	0.6502	2	0.67	1	NA	0.9983
P25036	0.3703	1	0.0607	0	0	0	0.0397	0	NA	0.4391
Q03231	0.6883	2	0.1494	0	0	0	0.2792	0	NA	0.6199
Q03236	0.051	0	0	0	0	0	0	0	NA	0.1058
Q08285	0.1625	0	0	0	0	0	0.0542	0	NA	0.156
P50943	0.1268	0	0	0	0	0	0	0	NA	0.1527
Q12234	0.9215	2	0.5806	2	0.3533	2	0.3678	2	NA	0.8843
P06701	0.3937	2	0.2699	3	0.2495	3	0.2515	3	NA	0.3115
P32905	0.2183	1	0.1587	1	0.2024	1	0.2024	1	NA	0.2364
P33419	0.8261	2	0.6561	1	0.6047	1	0.6126	1	NA	0.9192
Q00416	0.3622	4	0.2237	3	0.2004	3	0.1999	3	NA	0.9191
P38687	0.3272	2	0.0568	0	0.0801	1	0.0785	1	NA	0.2439
P36131	0.4169	1	0.0712	0	0	0	0.1017	0	NA	0.2554
P00447	0.1974	0	0.0386	0	0	0	0.0901	0	NA	0.1916
P38241	0.3407	1	0.0934	1	0.1154	1	0.1264	1	NA	0.2023
Q06524	0.534	3	0.1845	0	0	0	0.068	0	NA	0.2174
P25390	0.1803	3	0.0323	0	0	0	0.0541	0	NA	0.1516
Q3E739	0.3571	0	0.0286	0	0	0	0.2571	0	NA	0.126
Q2V2P6	0.15	0	0	0	0	0	0	0	NA	0.1056
P53249	0.7351	2	0.3432	1	0.3378	1	0.3703	1	NA	0.9773
P0C2J2	0.6096	2	0.5411	4	0.4658	3	0.4498	2	NA	0.8903
P53291	0.2613	0	0.027	0	0	0	0	0	NA	0.1008
P38079	0.2587	1	0.1017	0	0.1831	1	0.189	1	NA	0.1764
P0CX15	0.2873	3	0.1157	1	0.1205	1	0.142	1	NA	0.9126
P0CX22	0.2906	3	0.1153	1	0.1281	1	0.147	1	NA	0.8955
P46974	0.5312	3	0.3138	5	0.2239	3	0.2174	3	NA	0.9864
Q05543	0.7482	1	0.4841	2	0.4279	1	0.4523	1	NA	0.7314
P53969	0.1798	0	0.0145	0	0	0	0.0289	0	NA	0.3941
Q02825	0.379	2	0.1884	1	0.212	2	0.2184	2	NA	0.4886
P53012	0.1211	0	0.0105	0	0	0	0	0	NA	0.1321
Q03954	0.8788	1	0.3576	1	0	0	0.2424	0	NA	0.1555
P43545	0.2315	0	0.0101	0	0	0	0	0	NA	0.123
P36167	0.9797	1	0.6707	1	0.4837	1	0.4878	1	NA	0.9037
P24276	0.5336	5	0.4064	3	0.3752	3	0.3672	5	NA	0.8616
P38871	0.6375	3	0.4203	3	0.3065	2	0.3152	2	NA	0.7014
P39926	0.7322	3	0.339	1	0.1525	1	0.1864	1	NA	0.4426
P38707	0.2581	1	0.0415	0	0	0	0.0469	0	NA	0.1404
P38276	0.2682	0	0	0	0	0	0.0391	0	NA	0.1129
Q3E787	0.25	0	0	0	0	0	0.0577	0	NA	0.1608
P39109	0.1736	1	0.0185	0	0	0	0.0297	0	NA	0.1622
Q12187	0.3578	0	0.0183	0	0	0	0.211	0	NA	0.1985
O42831	0.3822	1	0.0382	0	0	0	0.0955	0	NA	0.1759

A0A023PZ94	0.2941	0	0	0	0	0	0.1275	0	NA	0.1685
P38202	1	1	0.8302	1	0.8491	1	0.8491	1	NA	0.9933
A0A023PYC	0.2596	0	0	0	0	0	0.0288	0	NA	0.0921
Q3E7Z7	0.475	0	0	0	0	0	0.375	0	NA	0.0248
P39703	0.2698	0	0.0233	0	0	0	0.0465	0	NA	0.3004
P25354	0.2469	0	0.0293	0	0	0	0.1046	0	NA	0.1498
Q07410	0.1277	0	0	0	0	0	0	0	NA	0.1245
Q07589	0.1011	0	0	0	0	0	0.0197	0	NA	0.1107
P46683	0.53	1	0.34	1	0.31	1	0.3	1	NA	0.2089
Q03482	0.96	1	0.1867	0	0.6933	1	0.7333	1	NA	0.9518
Q03983	0.2419	1	0.0022	0	0	0	0.0626	0	NA	0.1945
P0CX70	0.6432	2	0.5773	3	0.5523	3	0.5591	3	NA	0.9958
Q05503	0.22	0	0	0	0	0	0	0	NA	0.1218
P87283	0.4553	1	0	0	0	0	0.065	0	NA	0.2051
P0C5L1	0.1667	0	0	0	0	0	0	0	NA	0.0918
Q03788	0.5604	1	0.033	0	0	0	0.2527	0	NA	0.393
P38303	0.6408	1	0.3495	0	0	0	0.4854	1	NA	0.8331
P38294	0.3173	0	0.0385	0	0	0	0.2692	0	NA	0.2127
P40104	0.3525	0	0.0574	0	0	0	0.1639	0	NA	0.1447
Q03049	0.1192	0	0.0291	0	0	0	0.061	0	NA	0.1253
P39550	0.0708	0	0	0	0	0	0.0354	0	NA	0.1063
Q3E825	0.8276	1	0.1724	0	0	0	0.3678	0	NA	0.26
Q8TGM1	0.1765	0	0	0	0	0	0.1471	0	NA	0.0248
Q05881	0.3859	2	0.0338	0	0	0	0.0817	0	NA	0.241
P0CL24	0.2222	0	0	0	0	0	0	0	NA	0.0248
P36073	0.1462	0	0	0	0	0	0	0	NA	0.1442
P0C5Q1	0.125	0	0	0	0	0	0.1	0	NA	0.0248
P0CX59	0.6432	2	0.5841	3	0.5386	3	0.5432	3	NA	0.9949
P53820	1	1	0.8462	1	1	1	1	1	NA	0.9939
P18961	0.2969	1	0.1846	1	0.192	1	0.1965	1	NA	0.3262
Q12155	0.0621	0	0	0	0	0	0	0	NA	0.0995
Q03823	0.3444	4	0.1532	1	0.1495	2	0.1532	1	NA	0.5268
P0C2J6	0.6142	2	0.5616	3	0.4658	3	0.4612	3	NA	0.8677
P40361	0.268	2	0.1014	1	0.1025	1	0.1115	1	NA	0.3297
P38733	0.1278	0	0	0	0	0	0	0	NA	0.1162
P40490	0.3675	0	0.0855	0	0	0	0.2051	0	NA	0.1723
A0A023PYI5	0.3083	0	0.1053	0	0	0	0.1579	0	NA	0.1492
Q07746	0.3077	0	0.0684	0	0	0	0.1368	0	NA	0.294
P0CX93	0.0825	0	0	0	0	0	0	0	NA	0.0985
A0A023PXF	0.4286	0	0	0	0	0	0.102	0	NA	0.2453
P53891	0.3621	1	0.1946	1	0.2118	1	0.1995	1	NA	0.3615
P53912	0.1569	0	0	0	0	0	0	0	NA	0.1429
Q8TGT4	0.1429	0	0	0	0	0	0	0	NA	0.0894
Q3E735	0.2125	0	0	0	0	0	0.1375	0	NA	0.1089
P40169	0.3588	1	0.1528	0	0.2326	1	0.2525	1	NA	0.3034
P40519	0.4375	1	0	0	0	0	0.0417	0	NA	0.1808
Q8TGU2	0.2414	0	0	0	0	0	0	0	NA	0.2392
Q08110	0.0968	0	0	0	0	0	0	0	NA	0.1142
P0CL42	0.2062	0	0	0	0	0	0	0	NA	0.1374
Q8TGN6	0.3657	1	0.0278	0	0	0	0.0602	0	NA	0.2882
O13536	0.3689	0	0	0	0	0	0.2039	0	NA	0.1283
P53888	0.4046	1	0.0229	0	0	0	0.0916	0	NA	0.3128

Q3E7Z5	1	1	0.5217	0	0	0	0.4058	0	NA	0.575
Q08238	0.0379	0	0	0	0	0	0	0	NA	0.1251
P47024	0.4359	5	0.2241	2	0.1187	2	0.1265	2	NA	0.3464
P43578	0.2195	0	0.0793	0	0	0	0.1463	0	NA	0.1973
Q3E837	0.4359	0	0	0	0	0	0.2308	0	NA	0.0248
P38374	0.5692	1	0.1538	0	0	0	0.4462	0	NA	0.3582
P40422	0.4286	0	0	0	0	0	0.2	0	NA	0.1376
Q3E7X9	0.4328	0	0.0597	0	0	0	0.194	0	NA	0.2307
P39955	0.7001	3	0.5318	4	0.4604	4	0.4593	3	NA	0.9969
P20840	0.4846	1	0.0554	0	0	0	0.0369	0	NA	0.5635
P38957	0.1525	0	0	0	0	0	0	0	NA	0.1101
P38890	0.3403	2	0.1711	1	0.2167	1	0.2224	1	NA	0.198
Q04359	0.6902	2	0.2771	0	0.0982	0	0.1285	0	NA	0.2465
P32342	0.8743	1	0.5569	1	0.4251	1	0.497	1	NA	0.3855
P47057	0.5816	3	0.0851	0	0.0993	0	0.1111	0	NA	0.2494
P31109	0.5812	1	0.2051	0	0.2564	1	0.265	1	NA	0.1371
Q03406	0.483	2	0.1429	0	0	0	0.0952	0	NA	0.1894
P33894	0.232	1	0.0849	1	0.0924	1	0.0956	1	NA	0.255
P47002	0.3705	1	0.2103	3	0.2203	2	0.226	2	NA	0.6608
Q04311	0.5823	5	0.2832	2	0.2136	2	0.2215	2	NA	0.6914
Q3E7B6	0.2055	0	0	0	0	0	0	0	NA	0.1512
Q06708	0.2943	3	0.1	1	0.1102	2	0.1193	2	NA	0.2785
P38784	0.547	1	0.232	1	0.2707	1	0.2818	1	NA	0.8262
P04840	0.2155	1	0.0318	0	0	0	0.0671	0	NA	0.142
P38739	0.762	3	0.5041	5	0.443	3	0.4793	2	NA	0.9995
P43582	0.763	2	0.8104	2	0.7488	1	0.7441	1	NA	0.9973
Q04925	0.2482	0	0.0145	0	0	0	0.0337	0	NA	0.1518
P25577	0.2372	1	0.0577	0	0	0	0.109	0	NA	0.1829
A0A023PXB	0.2455	0	0.2	0	0.2636	0	0.2727	0	NA	0.3116
P25639	0.2599	2	0.0713	1	0.1173	1	0.1141	1	NA	0.3339
P38749	0.7394	2	0.4576	1	0.2303	1	0.2455	1	TRU	0.5721
Q12307	0.2119	0	0	0	0	0	0	0	NA	0.1218
Q08182	0.8939	2	0.7796	1	0.6531	1	0.6694	1	TRU	0.9954
Q06640	0.2531	2	0.1862	1	0.1653	2	0.1653	2	NA	0.2148
Q04923	0.1649	0	0	0	0	0	0	0	NA	0.098
Q8TGP4	0.1321	0	0	0	0	0	0	0	NA	0.1263
P0CX66	0.6432	2	0.5841	3	0.5386	3	0.5432	3	NA	0.9949
P87191	0.5935	1	0.1545	0	0	0	0.2683	0	NA	0.2731
Q07629	0.4953	1	0.3817	1	0.3722	1	0.3754	1	NA	0.8133
P90471	0.0946	0	0	0	0	0	0	0	NA	0.1181
I2HB52	0.9848	1	0	0	0	0	0.4091	0	NA	0.7543
Q2V2Q3	0.4627	0	0	0	0	0	0.3582	0	NA	0.6844
P0C5L4	0.1923	0	0	0	0	0	0.2115	0	NA	0.1908
Q03362	0.0893	0	0	0	0	0	0.0536	0	NA	0.1065
Q03418	0.2025	0	0.0123	0	0	0	0.0184	0	NA	0.1619
A0A023PZF2	0.1481	0	0	0	0	0	0.0296	0	NA	0.1129
A0A023PXD	0.9252	1	0.7477	0	0.5701	0	0.5794	0	NA	0.686
Q03612	0.4519	4	0.335	4	0.3003	4	0.3083	4	NA	0.8926
P39711	0.1383	0	0	0	0	0	0.1489	0	NA	0.13
P38331	0.2311	1	0.0336	0	0	0	0.1008	0	NA	0.3591
Q8TGM2	0.3043	0	0	0	0	0	0.3261	0	NA	0.0248
O13576	0.2205	0	0	0	0	0	0.126	0	NA	0.1877

O13565	0.3797	1	0.016	0	0	0	0.1925	0	NA	0.5662
Q06689	0.4267	3	0.0133	0	0	0	0.0459	0	NA	0.2949
P34161	0.9974	2	0.813	2	0.7377	3	0.7429	3	TRU	0.9905
P0CF00	0.3889	0	0	0	0	0	0.1296	0	NA	0.1812
P47087	0.5749	1	0.4155	2	0.4155	2	0.372	2	NA	0.9329
Q8TGJ7	1	1	0.2679	0	0.6964	0	0.6786	0	NA	0.9722
P39542	0.2065	1	0.005	0	0	0	0.0572	0	NA	0.1563
Q06199	0.2941	0	0.049	0	0	0	0.0882	0	NA	0.1013
Q6Q5F3	0.298	0	0.0397	0	0	0	0.1921	0	NA	0.3473
P47009	0.8365	2	0.8365	2	0.875	2	0.8558	2	NA	0.9578
P38746	0.2025	1	0.0123	0	0	0	0.0444	0	NA	0.1445
P53084	0.1065	0	0	0	0	0	0.0046	0	NA	0.1192
P0C5P3	0.3	0	0	0	0	0	0.3333	0	NA	0.0248
P36133	0.2216	1	0.0778	0	0.1796	1	0.1916	1	NA	0.1567
Q08842	0.1475	0	0	0	0	0	0.0492	0	NA	0.0969
P0CD98	0.0987	0	0.0197	0	0	0	0	0	NA	0.1308
Q6B0X1	0.5085	1	0.3559	1	0.3616	1	0.3842	1	NA	0.5136
Q06839	0.4166	2	0.164	1	0.1398	1	0.1566	1	NA	0.7363
P0C5R3	0.6234	0	0	0	0	0	0.0779	0	NA	0.1592
P0C5P4	0.3	0	0	0	0	0	0.18	0	NA	0.1635
Q08621	0.3248	0	0	0	0	0	0.1111	0	NA	0.1712
A0A023PZG	0.1074	0	0	0	0	0	0	0	NA	0.1166
P0CX97	0.2125	0	0	0	0	0	0	0	NA	0.1412
Q08027	0.0687	0	0.0076	0	0	0	0	0	NA	0.1243
Q12182	0.2759	1	0.1348	1	0.1473	1	0.1599	1	NA	0.2223
O13548	0.1695	0	0	0	0	0	0	0	NA	0.1366
Q08786	0.2432	0	0	0	0	0	0	0	NA	0.105
P36092	0.1509	0	0	0	0	0	0	0	NA	0.0869
O13575	0.7236	1	0	0	0	0	0.1789	0	NA	0.4361
Q08789	0.1739	0	0	0	0	0	0	0	NA	0.1669
Q08927	0.145	0	0	0	0	0	0	0	NA	0.1242
Q12458	0.1346	0	0.0032	0	0	0	0.0321	0	NA	0.1427
Q08743	0.2104	1	0	0	0	0	0	0	NA	0.1226
P53162	0.3052	1	0	0	0	0	0.0065	0	NA	0.1736
Q12486	0.2339	0	0.0413	0	0	0	0.0505	0	NA	0.1404
P38708	0.3285	3	0.0974	0	0	0	0.0916	0	NA	0.2246
P43541	0.1589	0	0	0	0	0	0.1391	0	NA	0.1814
P38809	0.9454	2	0.9426	3	0.694	2	0.6967	2	NA	0.9662
P53926	0.065	0	0	0	0	0	0	0	NA	0.0996
P38868	0.0982	0	0	0	0	0	0	0	NA	0.1764
P40461	0.2558	0	0	0	0	0	0.0853	0	NA	0.102
Q8TGU3	1	1	0.0303	0	0	0	0.303	0	NA	0.0248
A0A023PXN	0.0533	0	0	0	0	0	0.04	0	NA	0.1127
Q8TGR2	0.6632	1	0.1579	0	0.2632	0	0.2947	0	NA	0.1718
P43624	0.0984	0	0	0	0	0	0.1148	0	NA	0.1197
P25380	0.1814	1	0.0216	0	0	0	0.0259	0	NA	0.1134
Q02100	0.9645	4	0.7898	4	0.6213	3	0.6615	3	TRU	0.9995
P13574	0.7631	3	0.5669	4	0.4811	4	0.4666	3	TRU	0.9858
P38237	0.3636	4	0.1421	0	0	0	0.0883	0	NA	0.3616
P47161	0.3255	2	0.1443	2	0.1492	2	0.1517	2	NA	0.6806
Q12016	0.1522	0	0	0	0	0	0.0435	0	NA	0.1923
P32630	0.247	0	0.0361	0	0	0	0.1205	0	NA	0.2201

Q03897	0.3423	4	0.0897	1	0.0732	1	0.1106	0	NA	0.507
Q12363	0.3043	2	0.1465	0	0.1327	1	0.1419	1	NA	0.2082
P12611	0.5556	4	0.3045	1	0.3107	3	0.2963	2	NA	0.9549
Q8TGQ0	0.0513	0	0	0	0	0	0	0	NA	0.1042
P38168	0.4231	1	0	0	0	0	0.1058	0	NA	0.1617
P38216	0.8438	1	0.8047	1	0.8281	1	0.8281	1	NA	0.9954
P38189	0.0476	0	0	0	0	0	0.0476	0	NA	0.1322
P38256	0.0696	0	0	0	0	0	0	0	NA	0.0992
Q12111	0.1346	0	0	0	0	0	0	0	NA	0.1691
P25352	0.3529	1	0.1691	0	0	0	0.2132	0	NA	0.1631
P0C5L7	0.7632	0	0.1316	0	0	0	0.5526	0	NA	0.0248
Q03964	0.6409	2	0.5841	3	0.5477	3	0.5318	3	NA	0.9953
P87264	0.0093	0	0	0	0	0	0	0	NA	0.0977
Q6Q5X2	0.5686	0	0	0	0.549	0	0.549	0	NA	0.7798
Q12490	0.4462	4	0.3345	4	0.3066	4	0.2997	4	NA	0.8016
Q03480	0.0584	0	0	0	0	0	0	0	NA	0.1168
P87272	0.1217	0	0	0	0	0	0.0261	0	NA	0.1079
P0CX88	0.6667	1	0.036	0	0	0	0.1622	0	NA	0.1358
P53160	0.1852	0	0	0	0	0	0	0	NA	0.1456
P14680	0.6047	3	0.4486	5	0.4188	4	0.3854	3	NA	0.9381
Q06170	0.4417	1	0.275	1	0.3042	1	0.3458	1	NA	0.599
Q3E742	1	1	0.7059	1	0.7353	1	0.75	1	NA	0.9888
P53110	0.1108	0	0.0054	0	0	0	0	0	NA	0.1212
P36015	0.32	1	0	0	0	0	0.015	0	NA	0.135
Q8TGJ4	0.7368	0	0	0	0	0	0.3421	0	NA	0.0248
P40891	0.0533	0	0	0	0	0	0.04	0	NA	0.1127
P46996	0.236	2	0.0613	0	0	0	0.1189	0	NA	0.236
Q3E7Z2	0.1294	0	0.0353	0	0	0	0.1765	0	NA	0.1179
Q03177	0.5576	5	0.2614	1	0.2278	3	0.2758	2	NA	0.8132
P53066	0.5193	1	0.5635	1	0.2541	1	0.2928	1	NA	0.3728
P0C5Q2	0.2308	0	0	0	0	0	0.2308	0	NA	0.0248
Q8TGJ1	0.098	0	0	0	0	0	0	0	NA	0.156
Q99337	0.446	4	0.3293	4	0.3019	4	0.3047	4	NA	0.8692
O13587	0.37	0	0.08	0	0	0	0.17	0	NA	0.1205
Q2V2P3	0.6933	1	0.28	0	0	0	0.4667	0	NA	0.7912
Q3E751	0.1724	0	0	0	0	0	0	0	NA	0.0891
Q8TGL9	0.3333	0	0	0	0	0	0.2963	0	NA	0.0248
Q12253	0.1296	0	0	0	0	0	0.0556	0	NA	0.1089
P12688	0.2956	1	0.1794	1	0.1882	1	0.1926	1	NA	0.7464
Q06523	0.7862	2	0.423	2	0.3563	2	0.3609	2	NA	0.8976
A0A023PXL	0.0964	0	0	0	0	0	0	0	NA	0.1106
Q06616	0.8054	2	0.5339	1	0.5113	1	0.5023	1	NA	0.8029
Q04019	0.2767	1	0.0728	0	0	0	0.1408	0	NA	0.1701
Q3E769	0.1444	0	0	0	0	0	0	0	NA	0.0925
Q3E736	0.4744	1	0.0256	0	0	0	0.3846	1	NA	0.7321
Q12402	0.2889	0	0	0	0	0	0.1333	0	NA	0.1982
Q07834	0.1927	1	0.0727	1	0.0764	1	0.0788	1	NA	0.3615
Q12088	0.4581	4	0.3345	4	0.3037	4	0.3026	4	NA	0.8811
P0CF18	0.0995	0	0.0023	0	0	0	0.0255	0	NA	0.1103
P0CY02	0.3256	0	0	0	0	0	0.3953	0	NA	0.0248
Q8TGQ8	1	1	0.1429	0	0	0	0.4	0	NA	0.4672
P0C2J5	0.4712	4	0.383	6	0.3148	6	0.3091	7	NA	0.9475

P43538	0.1811	0	0	0	0	0	0.0434	0	NA	0.1253
P40082	0.1077	0	0	0	0	0	0.0615	0	NA	0.1279
P40497	0.6066	5	0.3112	0	0.2006	0	0.2449	1	NA	0.6586
P43617	0.1133	0	0	0	0	0	0	0	NA	0.1158
Q3E801	0.3704	0	0	0	0	0	0.3333	0	NA	0.0248
P53948	0.2727	0	0.0545	0	0	0	0.1	0	NA	0.1703
P50089	0.6739	4	0.5146	4	0.4599	4	0.4828	4	NA	0.9969
P53242	0.1815	0	0	0	0	0	0.0308	0	NA	0.1284
A0A023PYH	0.0887	0	0	0	0	0	0	0	NA	0.1157
Q06224	0.2503	2	0.0719	0	0	0	0.0822	0	NA	0.3469
P53735	0.4975	2	0.2628	1	0.2364	2	0.2512	2	NA	0.8236
P32527	0.5935	2	0.2887	1	0.2263	1	0.2818	1	NA	0.8226
P34240	0.5567	1	0.2843	1	0.2942	2	0.3201	2	NA	0.9662
P38204	0.2207	0	0.0207	0	0	0	0.0552	0	NA	0.1248
P41058	0.3036	0	0	0	0	0	0.4107	0	NA	0.4704
Q06639	0.3864	3	0.1345	1	0	0	0.122	0	NA	0.5688
Q03201	0.3448	1	0.0099	0	0	0	0.0443	0	NA	0.179
P38337	0.838	2	0.3732	0	0	0	0.4155	0	NA	0.7415
Q04195	0.6593	4	0.6051	5	0.5144	5	0.5254	6	NA	0.9953
P21691	0.1911	1	0.0076	0	0	0	0.0107	0	NA	0.1293
P39742	0.2642	1	0.0207	0	0	0	0	0	NA	0.1353
Q12133	0.1359	0	0	0	0	0	0.0326	0	NA	0.114
Q03012	0.2776	1	0.0453	0	0	0	0.1076	0	NA	0.1716
P54867	0.8492	2	0.4788	1	0.4709	2	0.5185	2	NA	0.9996
P53866	0.7405	6	0.4928	4	0.3677	2	0.3781	3	NA	0.8908
P53032	0.5686	3	0.204	0	0	0	0.1773	0	TRU	0.6397
P38326	0.8053	3	0.5809	1	0.5479	1	0.5413	1	NA	0.8514
P33300	0.2539	1	0	0	0	0	0.0576	0	NA	0.1587
P38931	0.4324	3	0.3014	2	0.2387	3	0.2585	3	NA	0.8683
P53277	0.9953	2	0.7302	2	0.6512	2	0.6326	2	NA	0.9257
P39079	0.1593	0	0.0073	0	0	0	0.0311	0	NA	0.1716
P36165	0.4165	3	0.3154	4	0.2725	3	0.278	4	NA	0.7623
P46678	0.8013	3	0.6582	4	0.5909	5	0.5774	5	NA	0.9947
P40308	0.1464	2	0.0639	1	0.0685	1	0.0779	1	NA	0.1612
P25368	0.4747	1	0.0673	0	0	0	0.0909	0	NA	0.244
P0CX32	0.4	1	0.1481	0	0	0	0.3111	1	NA	0.1708
P48164	0.4211	1	0.0684	0	0	0	0.1579	0	NA	0.1609
P40472	0.5273	2	0.292	1	0	0	0.3088	1	NA	0.9594
P38827	0.5352	5	0.3833	3	0.3389	3	0.3463	3	NA	0.9261
P39000	0.5039	2	0.2227	0	0.2051	1	0.2305	0	NA	0.7813
P33332	0.4731	5	0.3481	5	0.2844	5	0.2545	3	NA	0.666
P32566	0.6158	3	0.5842	2	0.4871	2	0.4574	1	NA	0.8487
P38314	0.4231	2	0.3681	2	0.3624	2	0.3397	2	NA	0.8709
P32790	0.7283	5	0.6286	10	0.422	6	0.414	7	NA	0.9992
Q02260	0.3699	1	0.2055	1	0.2055	1	0.2055	1	NA	0.3196
P46655	0.2218	1	0.048	0	0	0	0.0551	0	NA	0.2301
P43534	0.1382	0	0	0	0	0	0	0	NA	0.1196
P40552	0.6171	1	0.3457	1	0.5279	1	0.4572	1	NA	0.9996
P38764	0.281	3	0.1813	2	0.1833	2	0.1853	2	NA	0.5716
P46948	0.4634	1	0.0813	0	0	0	0.122	0	NA	0.1739
P36070	0.3461	2	0.1643	1	0.1627	1	0.1818	1	NA	0.2311
P0CX33	1	1	0.873	1	0.7937	1	0.7937	1	NA	0.9937

P38786	0.3003	1	0.099	0	0	0	0.099	0	NA	0.309
P33442	0.3725	1	0.1059	0	0	0	0.1216	0	NA	0.1752
P38352	0.4144	1	0.2669	1	0.2182	1	0.2198	1	NA	0.5836
P13856	0.4154	1	0.364	1	0.3676	1	0.375	1	NA	0.3558
P35187	0.6275	6	0.4623	6	0.3621	8	0.3469	4	NA	0.9991
Q08446	0.6177	2	0.2785	1	0.3443	2	0.3418	1	NA	0.8438
P40561	0.62	2	0.592	1	0.52	2	0.468	2	NA	0.6961
P53953	0.25	3	0.0548	1	0.0548	1	0.0537	1	NA	0.2214
Q12369	0.2949	1	0.1723	1	0.1586	2	0.1723	2	NA	0.2629
P46965	0.1915	0	0	0	0	0	0.117	0	NA	0.1037
Q08457	0.3035	2	0	0	0	0	0.0506	0	NA	0.1218
P41901	0.4141	3	0.123	0	0.127	1	0.1523	1	NA	0.2392
P40533	0.167	0	0.0106	0	0	0	0.0106	0	NA	0.1582
Q01852	0.5476	1	0.2065	0	0	0	0.1253	0	NA	0.3158
P33339	0.2966	3	0.1229	1	0.1395	1	0.1385	1	NA	0.316
P40414	1	1	0.8571	1	0.7702	1	0.7267	1	NA	0.966
P47131	0.1143	0	0	0	0	0	0	0	NA	0.1097
P53963	0.6013	5	0.3137	1	0.2663	1	0.268	1	NA	0.9476
Q8TGP1	0.3947	0	0	0	0	0	0.1842	0	NA	0.4045
P53222	1	1	0.8534	2	0.8966	2	0.8448	2	NA	0.9718
P53225	0.0485	0	0	0	0	0	0	0	NA	0.1115
Q08205	0.3846	1	0	0	0	0	0.2137	0	NA	0.1557
Q99315	0.265	3	0.1351	0	0	0	0.1034	0	NA	0.38
P53270	0.1134	0	0	0	0	0	0.0336	0	NA	0.1685
P53263	0.1342	0	0.0201	0	0	0	0.0067	0	NA	0.1684
P53303	0.3745	2	0.1831	0	0.177	1	0.1728	1	NA	0.318
P20107	0.3801	2	0.1674	0	0.267	1	0.2557	2	NA	0.6644
P38279	0.1486	0	0	0	0	0	0	0	NA	0.1105
P43551	0.2602	1	0.0215	0	0	0	0.0538	0	TRU	0.1742
Q12340	0.3142	3	0.084	1	0.1005	1	0.1069	1	TRU	0.3418
P47043	0.7966	5	0.4102	3	0.3432	3	0.3648	3	TRU	0.9937
Q02326	0.4773	1	0.0511	0	0	0	0.108	0	NA	0.1821
Q08219	0.2193	1	0.0058	0	0	0	0.0906	0	NA	0.1387
Q02805	0.5759	3	0.399	4	0.2891	2	0.3023	3	NA	0.9078
P53437	0.4712	3	0.1534	0	0	0	0.0932	0	NA	0.2009
Q08417	0.2356	1	0	0	0	0	0.0393	0	NA	0.1599
P26783	0.3911	0	0.1111	0	0	0	0.1511	0	NA	0.2652
Q05043	0.5904	3	0.4787	2	0.3511	1	0.391	2	NA	0.8338
P25343	0.2906	1	0	0	0	0	0.0453	0	NA	0.1432
P40496	0.3485	1	0.2462	1	0.197	1	0.2386	1	NA	0.2623
Q06236	0.244	0	0.0417	0	0	0	0.1786	0	NA	0.5123
P25808	0.2558	1	0.0215	0	0	0	0.0561	0	NA	0.119
P22213	0.2943	1	0.0946	0	0	0	0.0961	0	NA	0.3131
Q04398	0.063	0	0	0	0	0	0.0394	0	NA	0.1332
P40072	0.7701	1	0.7007	1	0.646	1	0.6569	1	NA	0.9624
P38778	0.2386	2	0.1056	0	0.1585	2	0.173	2	NA	0.3617
P32914	0.1961	0	0.0294	0	0	0	0.1275	0	NA	0.107
Q12505	0.3267	1	0.2311	1	0.1912	1	0.1952	1	NA	0.2683
P40485	0.5948	3	0.395	2	0.3848	3	0.3732	2	NA	0.8273
P04802	0.3339	1	0.1741	1	0.1706	1	0.1759	2	NA	0.3079
Q08686	0.375	1	0.0987	0	0.1382	1	0.148	1	NA	0.2091
Q08144	0.5013	3	0.2065	1	0.2015	1	0.2166	1	NA	0.5557

Q00764	0.1313	0	0.0202	0	0	0	0.0505	0	NA	0.1865
Q12272	0.2261	0	0.0087	0	0	0	0.0522	0	NA	0.2449
P16370	0.2987	1	0.2327	1	0.217	1	0.2201	0	NA	0.548
P25441	0.8294	3	0.6943	5	0.6327	4	0.5853	4	NA	0.9876
P0CX38	0.5551	1	0.2669	0	0	0	0.1695	0	NA	0.2486
Q07458	0.3844	1	0.2313	1	0.2177	1	0.2041	0	NA	0.5455
Q08986	0.184	1	0.0511	0	0.0647	1	0.0733	1	NA	0.1328
P06782	0.4297	2	0.2512	3	0.2417	3	0.2338	3	NA	0.6301
Q2V2Q2	0.4684	1	0.3038	0	0	0	0.3165	0	NA	0.4876
P34220	0.1818	0	0.012	0	0	0	0.0311	0	NA	0.1829
A0A023PXK	0.5781	3	0.0156	0	0	0	0.1198	0	NA	0.5033
P0C5P9	0.2778	0	0	0	0	0	0.1481	0	NA	0.2178
P0CL29	0.7749	1	0.3403	1	0.3822	1	0.466	1	NA	0.9992
P53156	0.45	2	0.1688	0	0.0938	0	0.1625	0	NA	0.3764
P53161	0.0672	0	0	0	0	0	0	0	NA	0.089
Q06137	0.2475	0	0.0393	0	0	0	0.0452	0	NA	0.1871
P47086	0.1839	0	0.0345	0	0	0	0.0651	0	NA	0.2246
P40892	0.148	0	0.0306	0	0	0	0.0765	0	NA	0.1726
Q8TGL8	0.7647	1	0	0	0	0	0.3922	0	NA	0.7179
Q04299	0.0915	0	0	0	0	0	0.0282	0	NA	0.1225
Q8TGQ9	0.8621	0	0	0	0	0	0.4483	0	NA	0.0248
Q8TGL7	1	1	0.0312	0	0	0	0.7812	0	NA	0.0248
Q02781	0.3955	1	0.2761	1	0.3358	1	0.3582	1	NA	0.8663
Q08533	0.4158	1	0	0	0	0	0.1188	0	NA	0.4412
Q3E7Z6	0.3333	0	0	0	0	0	0.2593	0	NA	0.0248
Q3E7Z1	0.1094	0	0	0	0	0	0	0	NA	0.098
O13521	0.1304	0	0	0	0	0	0.0261	0	NA	0.128
P0C5R0	0.1667	0	0	0	0	0	0.1667	0	NA	0.0248
Q08157	0.7114	2	0.5572	1	0.4936	4	0.4809	2	NA	0.989
Q8TGL3	1	1	0.2899	0	0	0	0.5652	0	NA	0.1924
Q08910	0.0388	0	0	0	0	0	0	0	NA	0.129
Q3E782	0.3404	0	0	0	0	0	0.1702	0	NA	0.0248
Q12040	0.3174	0	0.1217	0	0	0	0.0826	0	NA	0.2425
P53089	0.1386	0	0	0	0	0	0	0	NA	0.1069
P0C5Q5	0.4219	0	0	0	0	0	0.1094	0	NA	0.2353
Q08560	0.3542	1	0.0694	0	0	0	0.2014	0	NA	0.2313
Q8TGJ0	0.2182	0	0	0	0	0	0	0	NA	0.1854
Q08634	0.2409	1	0	0	0	0	0.0693	0	NA	0.1613
P32331	0.2085	0	0.101	0	0	0	0.0717	0	NA	0.1934
Q08811	0.3793	0	0	0	0	0	0	0	NA	0.3016
Q8TGL5	1	0	0.0385	0	0	0	0.5	0	NA	0.0248
Q04670	0.4581	4	0.335	4	0.3048	4	0.3043	4	NA	0.8855
A0A023PXH	0.1892	0	0	0	0	0	0	0	NA	0.1129
P0C5Q3	0.0816	0	0	0	0	0	0	0	NA	0.1523
P53827	0.0783	0	0	0	0	0	0	0	NA	0.1055
P51996	0.2658	1	0.1667	1	0.1892	1	0.1982	1	NA	0.3188
P38775	0.1429	0	0.0268	0	0	0	0.0125	0	NA	0.1228
P38776	0.1654	1	0.037	0	0	0	0.0739	0	NA	0.1553
P43540	0.2241	0	0	0	0	0	0.046	0	NA	0.1361
P53108	0.4129	1	0.2871	1	0.271	1	0.3097	1	NA	0.6992
P40521	0.0957	0	0	0	0	0	0	0	NA	0.1126
Q03187	0.4494	1	0.0075	0	0	0	0.1086	0	NA	0.6435

Q2V2P4	0.2192	0	0	0	0	0	0.0822	0	NA	0.1006
Q3E746	0.3962	0	0	0	0	0	0.3208	0	NA	0.5782
P53902	0.3778	1	0.0074	0	0	0	0.2296	0	NA	0.2776
Q08498	0.1416	0	0	0	0	0	0.0177	0	NA	0.1171
Q12331	0.3235	0	0.0515	0	0	0	0.0588	0	NA	0.1907
Q45U18	0.1791	0	0	0	0	0	0	0	NA	0.1224
P40586	0.1441	0	0	0	0	0	0.0508	0	NA	0.1384
A0A023PZD	0.6759	2	0	0	0	0	0.1389	0	NA	0.2933
P40448	0.1795	0	0	0	0	0	0	0	NA	0.142
Q12169	0.1513	0	0	0	0	0	0.0504	0	NA	0.1174
P0CX74	0.6432	2	0.5773	3	0.55	3	0.5591	3	NA	0.9956
P47101	0.455	2	0.1087	0	0	0	0.1409	0	NA	0.2272
Q3E7Z9	1	1	0.2581	0	0	0	0.4839	0	NA	0.0248
Q12141	0.4479	4	0.3328	4	0.31	4	0.3145	4	NA	0.8927
P53205	0.1111	0	0	0	0	0	0	0	NA	0.0967
P42844	0.454	1	0.1379	0	0	0	0.1437	0	NA	0.1601
Q12098	0.7366	3	0.5294	6	0.3369	3	0.3396	3	NA	0.9058
Q08491	0.3842	4	0.1473	1	0.1419	1	0.1473	2	NA	0.4199
Q04175	0.1176	1	0.0064	0	0	0	0.0148	0	NA	0.1693
P38088	0.2725	1	0.0435	0	0	0	0.0377	0	NA	0.192
P38854	0.9623	2	0.8829	4	0.8393	4	0.7857	5	NA	0.9992
Q08231	0.0703	0	0.0044	0	0	0	0.0308	0	NA	0.124
P00942	0.1452	0	0	0	0	0	0	0	NA	0.1196
P53507	0.35	0	0	0	0	0	0.1833	0	NA	0.1202
P25040	0.5613	2	0.174	0	0.1495	0	0.1667	0	NA	0.6247
P47142	0.3416	1	0.0891	0	0	0	0.1535	0	NA	0.2676
Q01477	0.6195	5	0.4243	3	0.3882	3	0.4112	3	NA	0.9946
P52917	0.4462	2	0.238	1	0.2243	1	0.2357	1	NA	0.6922
Q03388	0.8214	2	0.7094	3	0.6289	4	0.6025	5	NA	0.9882
Q05934	0.2775	3	0.1632	2	0.162	2	0.1731	2	NA	0.5281
P25591	0.948	2	0.5603	3	0.3262	2	0.3688	1	NA	0.8495
Q08924	0.0938	0	0	0	0	0	0.0049	0	NA	0.1174
Q96VH2	0.4167	0	0	0	0	0	0.2024	0	NA	0.1958
Q96VH3	0.424	1	0	0	0	0	0	0	NA	0.1362
P38109	0.1496	0	0	0	0	0	0.0118	0	NA	0.1383
P38220	0.0636	0	0	0	0	0	0	0	NA	0.0928
P25593	0.1308	0	0	0	0	0	0.0385	0	NA	0.1114
P38083	0.2698	0	0.0322	0	0	0	0.0248	0	NA	0.1362
P0C1V3	0.2131	0	0	0	0	0	0.0492	0	NA	0.2501
Q8TGV0	1	0	0	0	0	0	0.9615	0	NA	0.0248
P0C5M6	0.1077	0	0	0	0	0	0	0	NA	0.1376
Q3E7Y5	0.2339	1	0.0525	0	0	0	0.069	0	NA	0.1374
P38306	0.871	2	0.8802	3	0.7005	2	0.6682	2	NA	0.9883
P25600	0.1104	0	0	0	0	0	0.026	0	NA	0.1565
Q3E7A2	0.0926	0	0	0	0	0	0	0	NA	0.0959
P0C5M2	0	0	0	0	0	0	0	0	NA	0.1078
Q3E776	0.3167	1	0.025	0	0	0	0.175	0	NA	0.096
A0A023PYF	0.3655	1	0.0621	0	0	0	0.2828	0	NA	0.5482
Q08532	0.5	1	0	0	0	0	0.0154	0	NA	0.4319
Q2V2P0	0.8846	1	0.6026	0	0.5256	1	0.5256	1	NA	0.6217
Q08903	0.2143	0	0.0536	0	0	0	0.1429	0	NA	0.146
Q07074	0.4225	0	0.2535	0	0	0	0.3662	0	NA	0.6978

Q3E7B3	0.3	0	0	0	0	0	0.1429	0	NA	0.1136
Q04869	0.1547	1	0.0057	0	0	0	0.0287	0	NA	0.1596
P0CX98	0.2125	0	0	0	0	0	0	0	NA	0.1412
Q12484	0.3981	0	0.0185	0	0	0	0.1296	0	NA	0.4511
P53190	0.045	0	0	0	0	0	0	0	NA	0.1264
Q04893	0.9763	2	0.5702	7	0.6386	3	0.7149	7	NA	1
P0CY05	0.3256	0	0	0	0	0	0.3953	0	NA	0.0248
P0CF36	0.2138	0	0.1034	0	0	0	0.1034	0	NA	0.1404
Q8TGS8	0.1562	0	0	0	0	0	0	0	NA	0.0972
O13574	0.5128	1	0.1453	0	0	0	0.3504	0	NA	0.7393
O13572	0.104	0	0	0	0	0	0.0578	0	NA	0.1372
P01123	0.301	1	0.1117	0	0.1553	0	0.1748	0	NA	0.18
P53049	0.2857	4	0.0792	1	0.088	2	0.1022	2	NA	0.4685
P0CE96	0.4561	0	0	0	0	0	0.0439	0	NA	0.1137
Q12204	0.4308	2	0.2224	2	0.2126	2	0.2392	2	NA	0.5561
Q12243	0.1081	0	0	0	0	0	0	0	NA	0.1244
P40543	0.3545	1	0.1323	0	0	0	0.164	0	NA	0.2186
P38900	0.5144	1	0.3413	1	0.3141	1	0.351	1	NA	0.8723
A0A023PYH	0.1176	0	0	0	0	0	0	0	NA	0.1261
P38835	0.7047	5	0.4494	4	0.4106	5	0.4082	5	NA	0.9968
P0C5N4	0.4231	0	0	0	0	0	0.2692	0	NA	0.0248
P53856	0.3497	0	0	0	0	0	0	0	NA	0.1426
P53754	0.4081	1	0.0882	0	0	0	0.1029	0	NA	0.1624
A0A023PXN	0.3386	0	0	0	0	0	0	0	NA	0.1652
Q12275	0.2385	3	0.0959	2	0.0801	1	0.0892	1	NA	0.3308
Q3E743	0.0826	0	0	0	0	0	0.055	0	NA	0.087
Q05451	0.1786	0	0	0	0	0	0.1429	0	NA	0.0876
Q03886	0.275	0	0.05	0	0	0	0.3	0	NA	0.3267
Q3E828	0.0962	0	0	0	0	0	0	0	NA	0.1301
P40166	0.35	1	0.05	0	0	0	0.33	0	NA	0.2215
P53269	0.9382	1	0.0386	0	0	0	0.4286	0	NA	0.9988
P53275	0.3333	1	0.0417	0	0	0	0.0737	0	NA	0.1862
P47063	0.2029	0	0.0435	0	0	0	0.1522	0	NA	0.1395
P53300	0.2137	0	0	0	0	0	0.1624	0	NA	0.1127
Q12436	0.3128	1	0.1445	0	0	0	0.0924	0	NA	0.248
P32793	0.5321	2	0.438	1	0.4209	1	0.4423	1	NA	0.88
P53584	0.1307	1	0	0	0	0	0.0545	0	NA	0.1455
P50111	0.9158	2	0.8721	4	0.7923	5	0.765	4	NA	0.9993
P10964	0.3576	6	0.1611	1	0.1118	1	0.1208	2	NA	0.4898
P38771	0.6739	2	0.0565	0	0	0	0.0652	0	NA	0.1949
P25381	0.1385	0	0.0265	0	0	0	0.0122	0	NA	0.1452
P0CX55	0.2671	0	0.0685	0	0	0	0.1027	0	NA	0.125
P0CX51	0.3846	0	0.042	0	0.2028	0	0.2238	0	NA	0.1649
P25632	0.53	4	0.4037	4	0.3375	2	0.3251	2	NA	0.9394
P40161	0.4659	2	0.3407	1	0.3626	1	0.3714	1	NA	0.455
P38814	0.642	3	0.419	1	0.3228	1	0.3415	2	NA	0.6896
Q03976	0.5172	2	0.0972	0	0	0	0.1003	0	NA	0.326
P50110	0.2752	2	0.0306	0	0	0	0.0367	0	NA	0.1263
Q08281	0.5899	6	0.3729	5	0.3311	6	0.3542	7	NA	0.9963
Q12378	0.0867	0	0	0	0	0	0.0408	0	NA	0.1375
Q07798	0.1382	1	0	0	0	0	0.0138	0	NA	0.114
P36126	0.4528	9	0.2169	5	0.1961	5	0.2097	5	NA	0.8946

P06844	0.5104	2	0.2938	1	0.273	1	0.2671	1	NA	0.9308
P25648	0.1836	1	0.0385	0	0.0484	0	0.0568	0	NA	0.1862
Q08673	0.9095	1	0.3333	0	0	0	0.281	0	NA	0.9071
P41930	0.155	1	0	0	0	0	0.0764	1	NA	0.152
Q02794	0.518	3	0.3266	2	0.2568	1	0.2703	1	NA	0.3875
P40045	0.4683	1	0.0794	0	0	0	0.1508	0	NA	0.1963
Q8TGM6	0.6371	1	0.5242	1	0.5403	1	0.5323	1	NA	0.9899
Q04600	0.354	3	0.0496	0	0	0	0.0673	0	NA	0.1804
P40462	0.1617	1	0.0053	0	0	0	0.0137	0	NA	0.1317
P33448	0.5246	0	0.0164	0	0.4426	0	0.4098	0	NA	0.9045
P0CF16	0.2207	0	0	0	0	0	0	0	NA	0.1133
P40032	0.3401	2	0.0776	0	0	0	0.0683	0	NA	0.163
P16649	0.5694	3	0.4362	2	0.3534	1	0.3576	2	TRU	0.9159
P33122	0.8041	2	0.4674	0	0	0	0.4948	0	TRU	0.9408
P14682	0.6475	1	0.539	1	0.5153	1	0.5288	1	NA	0.933
P39547	0.2298	1	0.0766	0	0	0	0.166	0	NA	0.2451
P53874	0.6578	3	0.4912	3	0.4823	3	0.4798	3	NA	0.9882
P38290	0.3935	2	0.2058	0	0.1986	0	0.2166	0	NA	0.3343
P53276	0.3043	2	0.0533	0	0	0	0.0631	0	NA	0.1954
P53979	0.12	0	0	0	0	0	0	0	NA	0.1001
Q3E775	0.1875	0	0	0	0	0	0	0	NA	0.0248
Q12173	0.3586	1	0.169	1	0	0	0.1793	0	NA	0.2467
Q8TGU0	0.3214	0	0	0	0	0	0.2857	0	NA	0.0248
Q12316	0.4444	4	0.3345	4	0.3037	4	0.3014	4	NA	0.8929
P0CX64	0.4492	4	0.3469	6	0.2825	5	0.2757	7	NA	0.8815
P48238	0.7926	1	0.1152	0	0	0	0.2396	0	NA	0.3195
P40341	0.4473	3	0.2012	2	0.1927	1	0.1964	1	NA	0.5677
P40105	0.2986	3	0.1279	1	0.1315	1	0.1576	1	NA	0.9178
P40856	0.5388	4	0.4074	6	0.3809	6	0.3629	5	NA	0.8415
P32605	0.6879	2	0.1611	0	0	0	0.104	0	NA	0.3869
Q04947	0.4508	1	0.2441	1	0.2949	1	0.3051	1	NA	0.3472
P10663	0.1043	0	0	0	0	0	0	0	NA	0.1262
P40084	0.3274	1	0.0664	0	0	0	0.1283	0	NA	0.1542
P36136	0.1771	0	0.0627	0	0	0	0.0775	0	NA	0.1772
P34228	0.5967	7	0.4591	4	0.3118	5	0.3284	5	NA	0.9931
P32564	0.3155	1	0.0856	0	0	0	0.1658	0	NA	0.2538
P14359	0.5789	1	0.3759	0	0.5188	1	0.5188	1	NA	0.9889
P31376	0.6752	3	0.5584	3	0.496	3	0.4944	4	NA	0.9696
P53312	0.2904	1	0.096	0	0	0	0.0328	0	NA	0.1763
P39965	0.2205	0	0.0156	0	0	0	0.0087	0	NA	0.1415
Q06163	0.1448	1	0.0011	0	0	0	0.0124	0	NA	0.1629
Q12466	0.2251	3	0.059	1	0.0556	1	0.0641	1	NA	0.2421
P87108	0.5161	0	0	0	0	0	0.2903	0	NA	0.2435
Q08749	0.2135	1	0	0	0	0	0.026	0	NA	0.1144
Q04183	0.2071	3	0.0147	0	0	0	0.0636	1	NA	0.7731
Q05942	0.7636	1	0.4864	1	0.4727	1	0.5	1	NA	0.9424
P21771	0.4825	2	0.0629	0	0	0	0.1259	0	NA	0.3334
P47166	0.9137	3	0.5219	2	0.2107	1	0.1924	1	NA	0.5103
P39709	0.172	1	0.0034	0	0	0	0.0354	0	NA	0.1239
P49955	0.2091	1	0.1298	1	0.1195	2	0.1071	1	NA	0.1215
P40054	0.209	1	0.0362	0	0	0	0.0235	0	NA	0.1706
P38634	1	1	0.8908	2	0.6549	1	0.6268	1	NA	0.8918

P07560	0.2419	1	0.0279	0	0	0	0.1767	0	NA	0.256
P53628	0.4912	4	0.318	2	0.2615	2	0.2827	2	NA	0.5031
Q00711	0.2625	2	0.1313	0	0	0	0.0875	0	NA	0.1883
P89102	0.3543	3	0.0669	0	0	0	0.0896	0	NA	0.3083
P38699	0.4805	2	0.3042	2	0.2719	2	0.2692	2	TRU	0.6004
P46679	0.3259	3	0.0518	0	0	0	0.1047	0	NA	0.4159
P38704	0.7671	3	0.4917	3	0.3789	2	0.4159	2	TRU	0.943
P32343	0.4318	4	0.3022	1	0.2383	1	0.2297	1	NA	0.4044
Q03656	0.5633	2	0.3908	4	0.3679	2	0.3895	3	NA	0.9756
O74700	0.7471	0	0.0115	0	0	0	0.2529	0	NA	0.2055
P16120	0.2665	2	0.035	0	0	0	0.0389	0	NA	0.1262
P32367	0.5578	4	0.2542	2	0.2157	1	0.2157	1	NA	0.5477
Q3E764	1	1	1	1	1	1	0.9375	1	NA	0.9827
Q02648	0.2646	1	0.0688	0	0	0	0.0705	0	NA	0.2485
P47072	0.4581	3	0.1693	0	0.1515	1	0.164	1	NA	0.6657
Q12159	0.7168	2	0.7301	2	0.5487	2	0.5398	2	NA	0.9593
P36036	0.7586	2	0.5567	1	0.6305	2	0.6256	2	NA	0.9448
P32603	0.0854	0	0.0022	0	0	0	0	0	NA	0.1101
P52286	0.6237	1	0.4278	1	0.4433	1	0.4175	1	NA	0.9206
P43544	0.0405	0	0	0	0	0	0.018	0	NA	0.1036
P37262	0.3079	1	0.1365	0	0.2032	1	0.1841	0	NA	0.5396
P32579	0.2746	1	0.061	0	0	0	0.0399	0	NA	0.2274
P36164	0.2404	1	0.0297	0	0	0	0.1128	0	NA	0.174
P22803	0.25	0	0	0	0	0	0.1827	0	NA	0.0913
P00899	0.3156	1	0.0966	0	0	0	0.0493	0	NA	0.2003
P32772	0.6188	2	0.5336	2	0.4529	1	0.4709	1	NA	0.7686
Q02863	0.3106	2	0.0842	0	0	0	0.0842	0	NA	0.226
Q04511	0.3862	1	0.2455	2	0.226	1	0.2275	2	NA	0.483
P43593	0.4228	2	0.1202	0	0	0	0.0902	0	NA	0.2247
Q07963	0.196	3	0.0475	0	0.0502	0	0.0689	0	NA	0.29
Q02767	0.2603	1	0.0702	0	0	0	0.1157	0	NA	0.2636
P40003	0.5532	2	0.0567	0	0	0	0.0851	0	NA	0.2159
P38171	0.2647	0	0.0196	0	0	0	0.0588	0	NA	0.1993
Q3E781	1	1	0.5882	0	0.7941	0	0.7941	0	NA	0.0248
P38350	0.3985	0	0	0	0	0	0	0	NA	0.1285
O13543	0.3945	0	0	0	0	0	0.1468	0	NA	0.1326
Q03629	0.3731	0	0.1294	0	0	0	0.1443	0	NA	0.2023
O13545	0.2171	0	0.0543	0	0	0	0	0	NA	0.2747
Q06698	0.324	6	0.1359	2	0.108	2	0.1213	2	NA	0.5839
P53126	0.2162	0	0.045	0	0	0	0	0	NA	0.1261
P0C5R6	0.5179	0	0.0714	0	0	0	0.3929	0	NA	0.262
P40889	0.3652	4	0.1758	1	0.1854	1	0.1985	1	NA	0.8845
P0C2J1	0.4627	4	0.3368	4	0.3026	4	0.3066	4	NA	0.88
P36030	0.2174	0	0	0	0	0	0.0435	0	NA	0.133
P0CE69	0.1895	0	0.0327	0	0	0	0.0948	0	NA	0.1756
P53103	0.3178	0	0	0	0	0	0	0	NA	0.1127
P36155	0.5961	1	0.2443	1	0.1238	0	0.1433	0	NA	0.3253
Q6B0Y5	0.3095	0	0	0	0	0	0.3333	0	NA	0.0248
P0C5Q4	0.0811	0	0	0	0	0	0	0	NA	0.1449
Q08543	0.297	0	0.0099	0	0	0	0.1683	0	NA	0.1147
Q12414	0.4507	4	0.3345	4	0.3043	4	0.3077	4	NA	0.8974
P53087	0.528	1	0.0497	0	0	0	0.1056	0	NA	0.2325

Q12288	0.0996	0	0	0	0	0	0	0	NA	0.1071
P0CF33	0.2621	0	0.0097	0	0	0	0.2039	0	NA	0.2483
Q08958	0.3246	1	0	0	0	0	0.2368	0	NA	0.1511
P0C2I7	0.4496	4	0.3339	4	0.3026	4	0.306	4	NA	0.8921
P53074	0.3061	1	0.102	0	0.2041	0	0.2313	0	NA	0.1792
P0C5Q6	0.1622	0	0	0	0	0	0	0	NA	0.1039
P40556	0.2198	1	0.0241	0	0	0	0.0349	0	NA	0.1331
P28625	0.0849	0	0.0027	0	0	0	0.0356	0	NA	0.1286
P0CL30	0.7749	1	0.3403	1	0.3822	1	0.466	1	NA	0.9992
P38799	0.1486	0	0.0054	0	0	0	0.0199	0	NA	0.1197
P53884	0.1579	0	0	0	0	0	0.0053	0	NA	0.1347
P53936	0.3418	1	0	0	0	0	0.0823	0	NA	0.2313
Q8TGK1	0.5152	1	0	0	0	0	0.0303	0	NA	0.1321
P25602	0.413	1	0.0324	0	0	0	0.2105	0	NA	0.4446
Q04013	0.1624	0	0.0541	0	0	0	0.1051	0	NA	0.4057
P40163	0.3498	1	0	0	0	0	0.0296	0	NA	0.2412
P0CT86	0.5385	1	0.3462	0	0.3974	0	0.3974	0	NA	0.2185
P0C5N0	0.1129	0	0	0	0	0	0	0	NA	0.0832
P39973	0.5893	1	0.0268	0	0	0	0.3482	0	NA	0.4949
P39972	0.3607	0	0.0574	0	0	0	0.1639	0	NA	0.1502
P0CL34	0.2125	0	0	0	0	0	0	0	NA	0.1461
P38899	0.2289	1	0.058	0	0	0	0.0697	0	NA	0.1392
P53209	0.2526	1	0	0	0	0	0.1105	0	NA	0.1023
Q12324	0.2711	1	0.0622	0	0	0	0.0859	0	NA	0.1882
P53284	0.1964	0	0	0	0	0	0	0	NA	0.1142
P47062	0.2072	0	0	0	0	0	0.1081	0	NA	0.1489
Q08245	1	1	0.885	1	0.9292	1	0.9292	1	NA	0.9893
P32459	0.2154	0	0.0462	0	0	0	0.0103	0	NA	0.1548
P38179	0.1004	0	0.024	0	0	0	0.0633	0	NA	0.1555
P53629	0.3146	2	0.1495	1	0	0	0.0903	0	NA	0.1576
P27351	0.1729	1	0.1129	1	0.1229	1	0.1357	1	NA	0.1469
P25568	0.1307	0	0	0	0	0	0.0455	0	NA	0.1574
P61829	0.0395	0	0	0	0	0	0	0	NA	0.1252
P41698	0.8027	2	0.7297	4	0.6949	3	0.6534	2	NA	0.9991
P04821	0.4984	8	0.2687	4	0.2568	4	0.2838	4	NA	0.9951
P39103	0.4714	0	0.3286	0	0.3857	0	0.3857	0	NA	0.151
P53951	0.4739	4	0.0025	0	0	0	0.0546	0	NA	0.1478
Q08412	0.9173	3	0.9538	2	0.9538	2	0.9221	2	NA	0.9963
Q06686	0.2158	0	0	0	0	0	0.1203	0	NA	0.1703
P38909	0.2213	0	0.0219	0	0	0	0.0273	0	NA	0.1644
P47005	0.2579	2	0.0664	0	0	0	0.0664	0	NA	0.1488
P32325	0.6449	3	0.5043	4	0.2315	2	0.2841	2	NA	0.4917
Q05924	0.1367	0	0.0104	0	0	0	0.019	0	NA	0.1807
Q06409	0.2396	4	0.043	0	0.0461	0	0.0735	0	NA	0.4815
P40553	0.5628	1	0.2186	1	0.2605	1	0.2698	1	NA	0.2395
P47120	0.3569	2	0.1415	0	0	0	0.0646	0	NA	0.2537
Q07786	0.1261	0	0.0084	0	0	0	0.0504	0	NA	0.146
P47129	0.9482	2	0.8803	3	0.7443	2	0.6861	2	NA	0.99
Q08902	0.1534	1	0.0039	0	0	0	0.0466	0	NA	0.2006
P23180	0.2	0	0.0194	0	0	0	0.0581	0	NA	0.1632
P42884	0.2846	1	0.0372	0	0	0	0.0559	0	NA	0.1511
Q01976	0.2424	0	0.0476	0	0	0	0.026	0	NA	0.1574

P81450	0.5763	1	0	0	0	0	0.2203	0	NA	0.1246
P33314	0.3487	2	0.0942	1	0.0888	1	0.1014	1	NA	0.2899
P43132	0.3208	2	0.1426	0	0	0	0.1386	0	NA	0.3218
P36012	0.6463	1	0.4716	1	0.4934	1	0.5109	1	NA	0.7768
P41735	0.2876	1	0.1502	0	0	0	0.1288	0	NA	0.131
P47187	0.1175	0	0	0	0	0	0.0627	0	NA	0.1153
P32891	0.247	1	0.0596	0	0	0	0.0937	0	NA	0.3996
P38836	0.114	0	0.0186	0	0	0	0.0349	0	NA	0.1303
P36022	0.1518	6	0.0083	1	0	0	0.0108	1	NA	0.1138
P38125	0.2692	2	0.1608	2	0.1906	2	0.1818	1	NA	0.2604
P27637	0.8561	4	0.8096	6	0.7983	6	0.7602	6	NA	0.9998
Q99177	0.2757	0	0.0557	0	0	0	0.0938	0	NA	0.205
O13558	0.2451	0	0	0	0	0	0.2255	0	NA	0.1837
P21306	0.3387	0	0.1452	0	0	0	0.3387	0	NA	0.6143
P43583	0.1867	3	0.0471	1	0.0392	1	0.0462	1	NA	0.234
P36076	0.6445	2	0.6007	2	0.5464	5	0.5219	5	NA	0.9918
P53197	0.47	1	0.3127	3	0.258	2	0.2774	2	NA	0.6233
P00424	0.4248	1	0.0719	0	0	0	0.1503	0	NA	0.2253
P10174	0.1833	0	0	0	0	0	0.15	0	NA	0.0975
Q12395	0.1301	0	0.0409	0	0	0	0.0632	0	NA	0.1105
P40526	0.0751	0	0	0	0	0	0.0079	0	NA	0.1085
P53255	0.2643	1	0.0828	0	0	0	0.0848	0	NA	0.1505
Q05610	0.7562	2	0.5233	1	0.5644	2	0.5479	2	NA	0.9216
P23501	0.1683	0	0.0025	0	0	0	0.0248	0	NA	0.1362
P32892	0.6051	5	0.379	4	0.383	3	0.3763	3	NA	0.8084
P15436	0.2242	1	0.0729	2	0.0611	1	0.0547	1	NA	0.1922
Q05521	0.173	1	0.0138	0	0	0	0.0761	0	NA	0.1417
P48510	0.8284	1	0.6702	5	0.5147	2	0.3914	1	NA	0.9912
P32469	0.25	1	0.0533	0	0	0	0.0667	0	NA	0.1614
Q12476	0.8924	2	0.564	2	0.5378	2	0.5203	2	NA	0.964
Q01574	0.209	1	0.0813	1	0.0617	1	0.0743	1	NA	0.2153
P18238	0.0879	0	0.0065	0	0	0	0.0358	0	NA	0.132
Q12001	0.1066	0	0	0	0	0	0.0257	0	NA	0.119
P25641	0.3135	1	0.0788	0	0	0	0.1192	0	NA	0.3155
Q02887	0.4435	3	0.1653	1	0.1835	1	0.2298	1	NA	0.6618
P48016	0.1974	1	0.0388	0	0	0	0.0487	0	NA	0.273
P00830	0.184	1	0.0528	0	0	0	0.0489	0	NA	0.1889
Q12468	0.5318	2	0.2	1	0	0	0.1818	1	NA	0.4789
Q02770	0.515	1	0.2691	1	0.2757	1	0.2525	1	NA	0.4785
Q03772	1	1	1	1	0.8571	1	0.8457	1	NA	0.9795
P00163	0.0571	0	0	0	0	0	0	0	NA	0.1068
P26343	0.6394	1	0.2751	1	0	0	0.2714	0	TRU	0.6073
P46680	0.2537	1	0.0098	0	0	0	0.0341	0	NA	0.1925
P38285	0.3461	2	0.1475	1	0.1075	1	0.1421	1	NA	0.2494
Q08683	0.1825	0	0.0073	0	0	0	0.0292	0	NA	0.1613
P38090	0.2383	1	0.0235	0	0	0	0.0436	0	NA	0.1628
Q08269	0.5704	4	0.4796	2	0.4692	3	0.4692	4	NA	0.9409
P43549	0.5387	2	0.4675	4	0.3591	3	0.37	3	NA	0.9329
P46993	0.3206	1	0	0	0	0	0.0766	0	NA	0.2484
P53855	0.299	5	0.1212	0	0.1011	1	0.1244	0	NA	0.6948
P39986	0.2469	3	0.0387	0	0	0	0.0354	0	NA	0.1361
Q08409	0.1671	1	0.0201	0	0	0	0.0402	0	NA	0.2738

P38995	0.0837	0	0	0	0	0	0.009	0	NA	0.1194
Q05791	0.2667	2	0.1333	0	0	0	0.1139	0	NA	0.1464
P30283	0.3632	1	0.2529	1	0.223	1	0.1977	1	NA	0.2374
P38845	0.8065	2	0.7656	1	0.7656	3	0.7204	3	NA	0.9932
P46963	0.5507	3	0.0878	0	0	0	0.1014	0	NA	0.3139
Q03220	0.5031	2	0.2375	0	0.1812	0	0.1844	0	NA	0.4081
P0CH63	0.2844	1	0.0356	0	0	0	0.0711	0	NA	0.1433
P06773	0.1795	0	0	0	0	0	0.0128	0	NA	0.1532
P15365	0.2247	2	0.0331	0	0	0	0.0405	0	NA	0.156
Q08650	0.1962	1	0.0431	0	0.0742	1	0.0885	1	NA	0.1638
P25337	0.5096	2	0.0701	0	0	0	0.1656	0	NA	0.2052
P06115	0.2491	1	0.0747	0	0	0	0.0516	0	NA	0.1778
P14724	0.9919	1	0.9919	1	0.8871	1	0.75	2	NA	0.9771
P32457	0.525	3	0.3	2	0.3154	3	0.2904	2	NA	0.8386
P06243	0.2959	2	0.0868	0	0	0	0.1065	0	NA	0.1823
Q12127	0.8722	2	0.203	0	0.218	0	0.2406	0	NA	0.5435
P38170	0.6167	6	0.3873	3	0.3117	3	0.3316	3	NA	0.969
P53344	0.1732	0	0	0	0	0	0.0577	0	NA	0.146
P49956	0.3252	3	0.0999	0	0	0	0.1228	0	NA	0.2956
P36034	0.1179	0	0.0049	0	0	0	0.0516	0	NA	0.129
P69851	0.2361	0	0.2083	0	0	0	0.25	0	NA	0.1875
Q12248	0.3723	0	0.266	0	0	0	0.3404	0	NA	0.6367
P25618	0.1322	2	0.0241	0	0	0	0.0241	0	NA	0.2178
Q08300	0.2865	0	0.1111	0	0	0	0.0819	0	NA	0.2304
P53923	0.2617	1	0.0041	0	0	0	0.0548	0	NA	0.1282
P46947	0.7857	1	0.6955	2	0.688	2	0.6466	2	NA	0.9619
P07262	0.174	0	0.0066	0	0	0	0.0264	0	NA	0.1894
P16661	0.0913	0	0	0	0	0	0.0089	0	NA	0.1311
P32448	0.7543	5	0.3752	1	0.2743	1	0.2648	1	NA	0.4841
P38084	0.2315	1	0.0903	0	0.1051	1	0.11	1	NA	0.2848
P40074	0.1339	1	0	0	0	0	0.0357	0	NA	0.1496
P38313	0.5679	2	0.2377	0	0	0	0.2006	0	NA	0.325
P38265	0.0857	0	0	0	0	0	0	0	NA	0.1218
P53600	0.3175	0	0.0529	0	0	0	0.0794	0	NA	0.1697
P27680	0.1282	0	0.0128	0	0	0	0.016	0	NA	0.1214
P00420	0.0558	0	0.0037	0	0	0	0	0	NA	0.114
Q04935	0.5951	2	0.278	0	0.3463	1	0.3463	1	NA	0.2293
Q04632	0.5008	2	0.3097	1	0.2586	1	0.2817	1	NA	0.3724
Q04201	0.5556	1	0.6239	1	0.547	1	0.5385	1	NA	0.5687
Q08977	0.5662	3	0.2287	1	0.1869	1	0.1887	1	NA	0.6346
P43595	0.0594	0	0.005	0	0	0	0	0	NA	0.1269
P38928	0.5152	3	0.3366	4	0.2734	3	0.3001	3	NA	0.9466
Q12359	0.1673	1	0.0691	0	0	0	0.08	0	NA	0.1711
P53727	0.0789	0	0	0	0	0	0	0	NA	0.1031
P38360	0.4153	3	0.0609	0	0	0	0.0732	0	NA	0.4724
P53194	0.472	1	0.144	0	0	0	0.256	0	NA	0.8553
Q07992	0.304	0	0	0	0	0	0.152	0	NA	0.4037
P17106	0.9117	2	0.8348	1	0.7892	2	0.7635	1	TRU	0.9973
P48237	0.2488	1	0.0602	0	0.0637	1	0.0741	1	NA	0.2097
P24869	0.4868	1	0.3788	2	0.3259	2	0.2912	2	NA	0.6131
Q01649	0.5354	3	0.0993	1	0.0606	1	0.0606	1	NA	0.1309
Q12145	0.776	4	0.3912	0	0.2555	0	0.2681	0	NA	0.9139

Q06538	0.179	2	0.0256	0	0	0	0.0537	0	NA	0.1928
P47977	0.8175	2	0.3614	1	0.3193	1	0.3649	1	NA	0.9037
P07143	0.3948	2	0.1877	1	0.1909	1	0.1909	1	NA	0.5489
P38865	0.3016	1	0.0265	0	0	0	0.1217	0	NA	0.1789
Q03375	1	1	0.9926	2	0.8222	1	0.8148	1	NA	0.9921
P20447	0.3518	2	0.1606	1	0.1491	1	0.1549	1	NA	0.2693
P36120	0.3369	2	0.2318	1	0.1968	2	0.2008	1	NA	0.2614
P32330	0.4343	5	0.1995	2	0.1984	2	0.2512	2	NA	0.9287
Q12471	0.2771	1	0.0353	0	0	0	0.0605	0	NA	0.2331
P19414	0.2018	1	0.0925	1	0	0	0.09	1	NA	0.2163
P40433	0.5828	3	0.4232	3	0.3579	3	0.3857	4	NA	0.8596
P29589	0.3956	1	0.0659	0	0	0	0.2143	0	NA	0.8502
P47127	0.2716	2	0.033	0	0	0	0.0533	0	NA	0.1397
P40053	0.1946	0	0.0351	0	0	0	0.0271	0	NA	0.1588
P25694	0.2862	2	0.1964	2	0.1437	2	0.1497	2	NA	0.4324
P32656	0.2888	1	0.1681	0	0	0	0.0905	0	NA	0.2225
P33313	0.5584	2	0.174	1	0.1662	1	0.1688	1	NA	0.2893
P49017	0.355	1	0.0619	0	0	0	0.0619	0	NA	0.2054
P47976	0.7662	2	0.3169	1	0.2492	1	0.3231	1	NA	0.7361
P53088	0.1978	0	0.0056	0	0	0	0.0696	0	NA	0.1317
P38818	0.2205	0	0.0034	0	0	0	0.0085	0	NA	0.1329
P28240	0.2298	1	0.0682	0	0	0	0.088	0	NA	0.4223
P43547	0.2406	0	0	0	0	0	0.066	0	NA	0.1184
P53178	0.1634	0	0	0	0	0	0.0347	0	NA	0.1618
P29311	0.6554	3	0.206	1	0.1685	1	0.1873	1	NA	0.4081
Q12064	0.6632	2	0.0052	0	0	0	0.1295	0	NA	0.8956
P07253	0.4444	1	0.0556	0	0	0	0.0988	0	NA	0.1396
P38907	0.1965	1	0.0175	0	0	0	0.0502	0	NA	0.1192
P43634	0.3951	1	0.1744	2	0.1867	2	0.1806	2	TRU	0.4665
P29465	0.3648	3	0.1442	1	0.1365	2	0.1502	1	NA	0.761
Q12453	0.3482	1	0.2339	2	0.2181	3	0.2208	3	NA	0.3583
P53202	0.164	2	0.0108	0	0	0	0.0228	0	NA	0.128
P08678	0.42	2	0.3198	3	0.3006	7	0.2897	7	NA	0.9418
P53899	0.5328	1	0.1642	0	0	0	0.2518	0	NA	0.8695
Q12046	0.4897	1	0.1858	0	0	0	0.1947	0	NA	0.4792
P28319	0.7657	1	0.3431	1	0	0	0.3682	1	NA	0.9697
P15700	0.3333	1	0.0343	0	0	0	0.1078	0	NA	0.2509
P50112	0.097	0	0.041	0	0	0	0.0261	0	NA	0.154
P25587	0.4609	1	0.2656	0	0	0	0.168	0	NA	0.2043
P38139	0.1093	0	0.0053	0	0	0	0	0	NA	0.1306
P04173	0.1951	0	0.0302	0	0	0	0.033	0	TRU	0.2247
P33311	0.3014	2	0.1332	1	0.1475	1	0.1488	1	NA	0.2286
P32419	0.2187	1	0.0233	0	0	0	0.0204	0	NA	0.1507
P10962	0.6046	1	0.4248	1	0.402	1	0.4216	1	NA	0.8451
P53048	0.2825	2	0.086	0	0.1412	2	0.1575	2	NA	0.2633
P38295	0.2151	1	0.051	0	0	0	0.0643	0	NA	0.171
P53745	0.1707	0	0	0	0	0	0.0069	0	NA	0.1371
Q03825	0.8522	2	0.7916	2	0.7111	6	0.6214	5	TRU	0.9985
P36084	0.4858	1	0.3529	1	0.3359	1	0.334	1	NA	0.5434
Q3E7Y6	1	1	0.4384	0	0	0	0.4521	0	NA	0.1495
Q02642	0.5987	2	0.3822	0	0.4777	2	0.4522	2	NA	0.7943
P53136	0.2829	2	0.041	0	0	0	0.095	0	NA	0.23

P43611	0.1941	1	0.0922	1	0.1098	1	0.1314	1	NA	0.2086
P12695	0.3817	2	0.2884	2	0.249	2	0.2448	2	NA	0.643
P35845	0.4301	4	0.2862	4	0.25	3	0.2391	3	NA	0.7523
P36147	0.3655	1	0	0	0	0	0.0457	0	NA	0.1315
Q07987	0.1452	0	0	0	0	0	0.0484	0	NA	0.0992
Q04182	0.2217	2	0.1046	2	0.106	3	0.1099	3	NA	0.5061
P29468	0.2588	1	0.081	1	0.0915	1	0.1021	1	NA	0.3077
P38264	0.3404	1	0.1596	0	0.1915	1	0.1968	1	NA	0.2207
P42841	0.2538	2	0.1484	0	0.1634	1	0.1742	2	NA	0.1832
P34217	0.7964	3	0.6931	4	0.6392	4	0.5928	5	NA	0.9991
P53203	0.355	2	0.1234	0	0.1147	0	0.1558	1	NA	0.2782
P00560	0.1899	1	0.0457	0	0	0	0.0577	0	NA	0.1557
P33333	0.1452	1	0.0924	0	0.0957	0	0.0957	0	NA	0.13
P32598	0.1282	0	0.0288	0	0	0	0.0577	0	NA	0.1137
Q05778	0.2826	1	0.0725	0	0	0	0.1413	0	NA	0.3062
P38089	0.2188	0	0.0433	0	0	0	0.0789	0	NA	0.186
P34227	0.1954	0	0.0077	0	0	0	0.0268	0	NA	0.1238
P33203	0.4237	3	0.1544	1	0	0	0.0926	0	NA	0.1679
P10363	0.2249	0	0.0636	0	0	0	0.0758	0	NA	0.1317
P42073	0.7167	3	0.364	1	0.2627	1	0.3096	1	NA	0.8032
Q12270	0.145	1	0	0	0	0	0.0954	0	NA	0.2197
Q08003	0.595	1	0.1	0	0	0	0.145	0	NA	0.5984
P32445	0.4	0	0.1407	0	0	0	0.1556	0	NA	0.1459
P36526	0.3288	0	0.1507	0	0	0	0.1918	0	NA	0.3632
P53724	0.5036	1	0.295	0	0.3309	1	0.3525	1	NA	0.2639
P11745	0.2875	1	0.1106	1	0.14	1	0.14	1	NA	0.1743
Q12213	0.2705	1	0.1189	0	0.1066	0	0.1148	0	NA	0.1389
P22354	0.5179	2	0.1692	0	0	0	0.1179	0	NA	0.2157
Q07887	0.095	0	0	0	0	0	0	0	NA	0.0972
P25045	0.2419	1	0.0125	0	0	0	0.0448	0	NA	0.1441
Q3E772	1	1	1	1	1	1	1	1	NA	0.9938
P39534	0.1111	0	0	0	0	0	0.0833	0	NA	0.1138
Q08777	0.2514	1	0.1459	1	0.1862	2	0.1843	2	NA	0.2192
Q04472	0.3892	3	0.0878	1	0.1018	1	0.1038	1	NA	0.2572
P43638	0.628	5	0.5365	5	0.4657	5	0.4542	7	NA	0.997
P38920	0.3056	1	0.1261	1	0.1131	1	0.1157	1	NA	0.2082
P53141	0.2483	0	0	0	0	0	0.0537	0	NA	0.1426
P22148	0.5681	1	0.4791	1	0.4529	1	0.466	1	TRU	0.9255
P39016	0.5611	2	0.4354	5	0.3434	3	0.2689	1	NA	0.986
P53077	0.2033	0	0	0	0	0	0.0813	0	NA	0.1429
P10566	0.1783	0	0.0446	0	0	0	0.0541	0	NA	0.2123
Q12163	0.5127	1	0.5297	1	0	0	0.3136	0	NA	0.4764
P40165	0.1098	0	0	0	0	0	0.0244	0	NA	0.1751
Q92316	0.2442	0	0	0	0	0	0	0	NA	0.101
P48439	0.1343	0	0	0	0	0	0	0	NA	0.1269
P05150	0.1598	0	0.0089	0	0	0	0.0414	0	NA	0.1467
Q04430	0.2125	1	0.0381	0	0	0	0.0518	0	NA	0.3214
Q12511	0.3794	2	0.1923	0	0.1399	1	0.1556	0	NA	0.5327
P38075	0.4693	1	0.1009	0	0	0	0.0614	0	NA	0.1832
Q00618	0.1927	0	0	0	0	0	0.0398	0	NA	0.1183
Q12036	0.1561	0	0	0	0	0	0	0	NA	0.1269
P19881	0.141	0	0	0	0	0	0.0321	0	NA	0.1266

P23287	0.3834	3	0.1212	1	0.1085	1	0.1175	1	NA	0.2926
P52290	0.1838	1	0.0021	0	0	0	0.0171	0	NA	0.1443
P38624	0.1535	0	0	0	0	0	0.0326	0	NA	0.1255
P40302	0.1111	0	0	0	0	0	0.0385	0	NA	0.1243
P42946	0.1305	1	0	0	0	0	0	0	NA	0.2046
Q00246	0.4502	2	0.1787	0	0.2131	1	0.2371	1	NA	0.1911
P38754	0.1449	0	0	0	0	0	0.0725	0	NA	0.1328
P02406	0.4161	1	0.302	1	0.2752	1	0.2752	1	NA	0.2492
P0CX27	1	1	0.1792	0	0	0	0.3868	0	NA	0.4323
P51998	0.3322	1	0.2413	1	0.2063	1	0.2378	1	NA	0.4751
O14467	0.8411	1	0.8477	2	0.7351	2	0.7351	2	TRU	0.6743
Q12171	0.1897	0	0.0289	0	0	0	0.0096	0	NA	0.1469
P17505	0.2665	1	0.0928	0	0	0	0.0389	0	NA	0.1853
Q04341	0.4793	1	0	0	0	0	0.0496	0	NA	0.1239
P40002	0.7988	2	0.7042	5	0.5901	5	0.536	4	NA	0.9993
Q06211	0.1528	1	0.0149	0	0	0	0.0149	0	NA	0.1836
P47164	0.2128	1	0.0532	0	0	0	0.0548	0	NA	0.1807
P32906	0.1785	0	0	0	0	0	0.0219	0	NA	0.4345
P29952	0.3706	1	0.1399	0	0	0	0.1119	0	NA	0.3671
P47083	0.8078	2	0.6138	5	0.5211	3	0.5177	3	NA	0.8848
P40364	0.3968	1	0.2222	0	0	0	0.2183	0	NA	0.2306
P15424	0.3434	3	0.1536	2	0.1551	2	0.1687	2	NA	0.3034
P48563	0.1919	3	0.0446	0	0.0581	1	0.0636	1	NA	0.2971
P47123	0.211	0	0.1193	0	0	0	0.0642	0	NA	0.2117
P38626	0.1373	0	0.0035	0	0	0	0.0176	0	NA	0.1657
P30771	0.3059	5	0.0721	0	0.068	1	0.0762	0	NA	0.3247
P40360	0.2997	2	0.0174	0	0	0	0.0453	0	NA	0.2054
P53617	0.5565	3	0.4643	2	0.44	2	0.4417	2	NA	0.9758
P53927	0.5955	2	0.4364	1	0.3727	1	0.4227	1	NA	0.8306
P06169	0.0906	0	0.0124	0	0	0	0.0213	0	NA	0.125
P40038	0.6119	2	0.5524	2	0.4952	4	0.4476	2	NA	0.9533
P53632	0.4675	3	0.3579	4	0.3545	2	0.3442	3	NA	0.8246
P24867	0.3513	1	0.0573	0	0	0	0.0824	0	NA	0.2235
P24004	0.2167	1	0.0374	0	0	0	0.0556	0	NA	0.2083
P39718	0.3278	0	0.1778	0	0	0	0.2111	0	NA	0.3586
Q03289	0.1925	0	0.0027	0	0	0	0.0642	0	NA	0.2102
P39685	0.2169	3	0.0553	1	0.0681	1	0.08	1	NA	0.2415
P31382	0.1647	1	0.1041	0	0.0791	1	0.0896	1	NA	0.3199
P23594	0.2439	1	0.2033	1	0.2114	1	0.2249	1	NA	0.2093
P38336	0.448	1	0.0108	0	0	0	0.0323	0	NA	0.1349
P47033	0.8138	1	0.3927	5	0.3723	5	0.4745	6	NA	1
P25502	0.4372	4	0.2441	2	0.1716	1	0.1859	3	TRU	0.4553
Q07800	0.6066	1	0.5386	1	0.5504	1	0.5504	1	NA	0.9763
P04161	0.1589	0	0	0	0	0	0	0	NA	0.1043
Q9ZZX9	0.125	0	0	0	0	0	0	0	NA	0.1584
Q12223	0.2773	0	0.021	0	0	0	0.0966	0	NA	0.1906
P38212	0.7559	1	0.493	1	0.446	1	0.385	0	NA	0.6418
Q03530	0.1206	0	0.0159	0	0	0	0.0476	0	NA	0.1463
P32611	0.4249	2	0.2494	1	0.1959	1	0.2341	1	NA	0.4599
Q04598	1	1	0.0381	0	0	0	0.1429	0	NA	0.2663
P22353	0.3992	1	0.1008	0	0	0	0.1218	0	NA	0.2511
P46946	0.6319	3	0.3942	0	0.2087	0	0.2348	0	NA	0.4468

P47103	0.3232	2	0.0025	0	0	0	0.0585	0	NA	0.2499
P53854	1	1	0.6536	1	0.6927	1	0.6034	1	NA	0.9134
P54861	0.317	2	0.1242	1	0.1096	1	0.1057	1	NA	0.4025
Q3E808	0.1875	0	0	0	0	0	0.25	0	NA	0.2021
P40059	0.9119	4	0.8284	5	0.6776	4	0.7075	7	NA	0.9999
P46957	0.2259	1	0.0637	0	0	0	0.1047	0	NA	0.1761
P50090	0.5896	5	0.2914	2	0.1995	2	0.2358	1	NA	0.9396
P32350	0.4735	3	0.2429	2	0.1737	1	0.1872	1	NA	0.7956
P33399	0.4945	1	0.2727	1	0.2655	1	0.2727	1	NA	0.4253
P42838	0.2367	1	0.0894	1	0.1087	1	0.1304	1	NA	0.2464
Q3E827	1	1	1	1	1	1	1	1	NA	0.6567
P53094	0.6059	4	0.4055	6	0.3867	4	0.3887	5	NA	0.9974
P38174	0.3064	1	0.2209	1	0.1473	1	0.152	1	NA	0.2806
P32259	0.1889	1	0.0349	0	0.0359	0	0.04	0	NA	0.4416
Q01662	0.2739	1	0.1085	0	0	0	0.093	0	NA	0.2274
P39943	0.8426	3	0.6548	3	0.3858	2	0.5051	2	TRU	0.9766
P26188	0.25	0	0	0	0	0	0.0585	0	NA	0.1333
P32389	0.8586	3	0.692	2	0.6756	3	0.6696	3	TRU	0.9964
P25338	0.0977	0	0	0	0	0	0	0	NA	0.0942
P40965	0.2392	2	0.0285	0	0	0	0.0513	0	NA	0.4465
P52920	0.2622	1	0.1037	0	0	0	0.1006	0	NA	0.2605
P40897	0.1502	1	0.0676	0	0.0763	1	0.0701	0	NA	0.1127
P54964	0.2825	1	0.1822	1	0.1636	1	0.1487	1	NA	0.2707
Q03558	0.2075	1	0.1075	0	0	0	0.1275	0	NA	0.5495
P41816	0.1525	0	0.0375	0	0	0	0.05	0	NA	0.2941
P25646	0.19	0	0.0566	0	0	0	0.0769	0	NA	0.2336
P46988	0.5413	0	0.0092	0	0	0	0.0917	0	NA	0.1646
P36093	0.571	3	0.3962	1	0.2514	1	0.2705	1	TRU	0.4688
P18898	0.1941	0	0.0053	0	0	0	0.0293	0	NA	0.2134
P32634	0.6429	4	0.4621	7	0.4113	7	0.4273	8	NA	0.9987
Q04119	0.3383	3	0.1261	0	0.1098	0	0.1424	0	NA	0.5082
P33297	0.5323	2	0.1751	0	0	0	0.106	0	NA	0.3546
Q06624	0.2651	1	0.0288	0	0	0	0.0432	0	NA	0.1242
P40525	0.5868	0	0.4215	0	0	0	0.2727	0	NA	0.1575
P0C2H9	0.1947	0	0	0	0	0	0.1504	0	NA	0.1487
Q02685	0.4274	1	0.1328	0	0	0	0.2158	0	NA	0.2975
Q03195	0.2319	2	0.0197	0	0	0	0.0378	0	NA	0.1555
Q07915	0.6784	1	0.2161	0	0.2563	1	0.2563	1	NA	0.2565
P36528	0.3879	1	0.1708	0	0.1886	1	0.2242	1	NA	0.3888
P05737	0.2787	1	0.0943	0	0.127	0	0.1352	0	NA	0.1478
Q12250	0.3011	1	0.0045	0	0	0	0.0382	0	NA	0.1644
P43603	0.5425	1	0.4009	1	0.4183	1	0.4466	1	NA	0.9639
P53163	0.8557	2	0.2062	0	0	0	0.2474	0	NA	0.8117
P40549	0.2159	0	0.0254	0	0	0	0.0286	0	NA	0.1863
P32559	0.2643	2	0.0057	0	0	0	0.0247	0	NA	0.2479
P40050	0.2733	2	0.0596	0	0	0	0.0814	0	NA	0.2001
P53583	0.1273	0	0	0	0	0	0.0148	0	NA	0.1309
P08593	0.3172	1	0.1455	0	0	0	0.1082	0	NA	0.162
P47007	0.3403	1	0.055	0	0	0	0.0864	0	NA	0.2423
Q03920	0.0724	0	0.009	0	0	0	0	0	NA	0.1029
P38205	0.2427	3	0.1506	2	0.1287	1	0.136	1	NA	0.3551
P08964	0.6696	7	0.1421	0	0	0	0.0617	1	NA	0.7294

P53883	0.7568	3	0.5484	3	0.5261	3	0.5633	3	NA	0.9804
P06102	0.7404	3	0.512	1	0.4234	3	0.4067	3	NA	0.9613
P41543	0.1239	0	0.0147	0	0	0	0.0168	0	NA	0.1418
P25693	0.474	1	0.1494	0	0	0	0.1883	1	NA	0.4189
Q06608	0.4	1	0.2	0	0	0	0.15	0	NA	0.2472
P33400	0.8032	5	0.3984	2	0.3584	2	0.3792	2	TRU	0.9986
Q12462	0.1186	0	0.0042	0	0	0	0.0636	0	NA	0.1235
Q05568	0.3383	1	0.0148	0	0	0	0.0475	0	NA	0.1682
P50085	0.2613	2	0.0323	0	0	0	0.071	0	NA	0.1557
P38244	0.2613	3	0.1045	1	0.0861	1	0.0953	1	NA	0.3262
Q12412	0.2171	1	0.0835	1	0.1076	1	0.102	1	NA	0.3425
P26570	0.5939	2	0.5477	1	0.5506	1	0.5462	3	NA	0.9854
P00635	0.0707	0	0.0043	0	0	0	0.0107	0	NA	0.117
Q04049	0.3212	3	0.0538	0	0.106	1	0.1187	1	NA	0.2071
P20095	0.3082	2	0.1963	1	0.1553	1	0.1667	1	NA	0.2489
Q03338	0.6908	2	0.4264	4	0.3475	2	0.3113	2	NA	0.7277
P22216	0.4032	2	0.1851	1	0.151	2	0.1583	2	NA	0.5332
P35196	0.2308	0	0.0315	0	0	0	0.0944	0	NA	0.1381
P38206	0.1237	0	0.0105	0	0	0	0.0383	0	NA	0.1125
P43565	0.574	5	0.3859	7	0.3689	6	0.3718	6	NA	0.9973
P38741	0.6999	3	0.5666	5	0.5161	4	0.5049	4	NA	0.9984
P32862	0.6436	7	0.506	5	0.4803	7	0.4778	6	TRU	0.9983
P05740	0.4565	1	0.1576	0	0	0	0.2011	0	NA	0.2866
P52893	0.3328	1	0.098	0	0	0	0.0693	0	NA	0.4092
P38013	0.2102	0	0	0	0	0	0.0568	0	NA	0.1173
P36000	0.2603	2	0.0523	0	0	0	0.0427	0	NA	0.2107
P38077	0.3441	1	0.0225	0	0	0	0.045	0	NA	0.2819
P02293	0.6183	1	0.3588	1	0.4122	1	0.4351	1	NA	0.9286
P25333	0.5804	4	0.4229	2	0.3449	2	0.3615	3	NA	0.845
Q07653	0.9799	2	0.999	1	1	1	1	1	NA	0.9998
P06633	0.2136	0	0.0682	0	0	0	0.1	0	NA	0.1367
P33734	0.1649	0	0.0199	0	0	0	0.0163	0	NA	0.1864
Q06674	0.1498	0	0.0048	0	0	0	0.029	0	NA	0.1426
P40495	0.1321	0	0.0135	0	0	0	0.0458	0	NA	0.1756
Q08750	0.2046	1	0.023	0	0	0	0.0292	0	NA	0.151
P52919	0.9718	1	0.6458	3	0.4232	2	0.5204	2	NA	0.7517
P52891	0.1722	1	0.0055	0	0	0	0.0165	0	NA	0.1367
P38826	0.4736	1	0.3678	1	0.3655	1	0.3839	1	NA	0.2768
Q12451	0.509	4	0.2814	4	0.2276	2	0.2393	2	NA	0.9614
P21957	0.802	5	0.4777	1	0.4629	3	0.4282	2	TRU	0.9691
P10834	0.2833	1	0.0478	0	0	0	0.0614	0	NA	0.1434
P25584	0.0833	0	0	0	0	0	0	0	NA	0.1187
P40005	0.4324	1	0.018	0	0	0	0.1081	0	NA	0.1507
P40473	0.8946	2	0.7835	4	0.6809	3	0.6239	3	NA	0.9924
Q12008	0.254	1	0.0772	0	0	0	0.0965	0	NA	0.3745
P38693	0.1156	0	0.0043	0	0	0	0.0107	0	NA	0.1205
Q12146	0.3866	1	0	0	0	0	0.0825	0	NA	0.307
P36081	0.3495	2	0.0944	0	0	0	0.1582	1	NA	0.1632
Q12221	0.5898	5	0.4558	4	0.3609	5	0.3516	3	NA	0.9861
Q9ZZV8	1	1	0.8824	1	0.9804	1	0.9804	1	NA	0.9939
Q00816	0.8373	5	0.8107	9	0.7239	7	0.6963	8	NA	0.9991
P30775	0.4383	2	0.247	1	0.1864	1	0.2131	1	NA	0.3844

P38622	0.3496	2	0.0586	0	0	0	0.0879	0	NA	0.1922
P38127	0.2175	1	0.0265	0	0	0	0.0265	0	NA	0.1777
P0CX53	0.3939	0	0.1212	0	0	0	0.1576	0	NA	0.1869
Q06678	0.3406	1	0.0245	0	0	0	0.0654	0	NA	0.1755
P14063	0.5191	1	0.1298	0	0	0	0.2977	0	NA	0.2288
P53140	0.1858	1	0.0867	0	0	0	0.0851	0	NA	0.2004
P05318	0.5	1	0.2547	0	0.4151	1	0.4245	1	NA	0.8581
P25367	1	1	0.8519	1	0.8519	1	0.8198	1	NA	0.9997
P35178	0.3309	1	0.1511	0	0.1906	0	0.1978	0	NA	0.375
P25628	0.2869	2	0.1361	1	0.1033	1	0.1115	1	NA	0.2311
P22936	0.3924	1	0.1499	1	0.1717	1	0.1744	1	NA	0.2225
P39524	0.2487	2	0.1166	2	0.0952	2	0.0841	2	NA	0.1912
Q04052	0.4821	3	0.2242	3	0.1579	1	0.1874	2	TRU	0.4368
Q06628	0.7154	1	0.6084	4	0.5637	3	0.5163	5	NA	0.9929
P81449	0.5104	1	0.0104	0	0	0	0.0938	0	NA	0.1627
Q12373	0.5481	2	0.4753	1	0.4649	2	0.4675	2	NA	0.9122
P00498	0.0774	0	0.0034	0	0	0	0.0135	0	NA	0.1171
Q02888	0.5769	1	0.0769	0	0	0	0.1374	0	NA	0.6041
P38431	0.4469	2	0.1654	1	0.2074	1	0.2123	1	NA	0.5597
P40325	0.5707	1	0.4394	1	0.4596	1	0.4596	1	NA	0.8154
P54839	0.2037	1	0.0489	0	0	0	0.0407	0	NA	0.151
P47034	0.1679	0	0	0	0	0	0.0992	0	NA	0.118
P40559	0.2389	2	0.0391	0	0.0687	1	0.0899	1	NA	0.1601
Q02821	0.3432	1	0.2048	1	0.1771	1	0.1661	1	NA	0.3638
Q05533	0.1233	0	0.0171	0	0	0	0.0616	0	NA	0.139
Q07532	0.7819	3	0.5042	3	0.5156	3	0.5014	3	NA	0.9562
P38702	0.1317	0	0.0532	0	0	0	0.056	0	NA	0.1065
P35728	0.6599	2	0.5465	2	0.3243	2	0.3605	2	NA	0.5606
P25588	0.9471	6	0.687	8	0.4845	6	0.4845	5	NA	0.9986
P53604	0.8667	2	0.8095	1	0.7429	1	0.7381	1	NA	0.9965
Q03723	0.0693	0	0	0	0	0	0	0	NA	0.1222
P0CE86	0.1341	0	0.0061	0	0	0	0.061	0	NA	0.1005
P53756	0.141	1	0.0083	0	0	0	0.0195	0	NA	0.1799
Q3E770	0.1833	0	0	0	0	0	0.025	0	NA	0.0875
P53010	0.1785	2	0.0179	0	0	0	0.0206	0	NA	0.263
Q12033	0.2844	3	0.0287	0	0	0	0.0318	0	NA	0.1315
P53238	0.5015	1	0.4299	1	0.3493	1	0.3254	1	NA	0.2198
P38744	0.2083	0	0	0	0	0	0.0667	0	NA	0.1899
Q08580	0.3351	1	0.0718	0	0	0	0.1303	0	NA	0.1454
P40961	0.331	2	0.0906	0	0	0	0.1045	0	NA	0.172
Q12245	0.2635	0	0	0	0	0	0.0405	0	NA	0.1364
P07272	0.3673	4	0.1239	0	0.0885	0	0.1162	0	TRU	0.3749
P35999	0.1995	1	0.0013	0	0	0	0.0117	0	NA	0.172
P87284	0.1091	0	0	0	0	0	0	0	NA	0.0898
P32524	0.4	2	0.2143	0	0.2393	1	0.2464	1	NA	0.3165
P23724	0.2075	0	0.0332	0	0	0	0.0996	0	NA	0.164
P19735	0.3048	1	0.1924	2	0.1624	1	0.158	1	NA	0.2047
P38953	0.3621	2	0.1084	0	0.0887	0	0.1108	0	NA	0.1866
P28272	0.0892	0	0.0287	0	0	0	0.0446	0	NA	0.1592
P03872	0.6351	2	0.3243	1	0.2838	2	0.3041	2	NA	0.431
Q08742	0.2349	0	0.0336	0	0	0	0.1208	0	NA	0.2117
P26755	0.459	1	0.1066	0	0	0	0.1066	0	NA	0.2582

P33312	0.1844	0	0.0861	0	0	0	0.1066	0	NA	0.2061
Q12504	0.2611	2	0.0364	0	0	0	0.0789	0	NA	0.1276
P40065	0.5828	2	0.2881	0	0.3146	1	0.3179	1	NA	0.4965
P0CW41	0.3243	2	0.0543	0	0	0	0.0696	0	NA	0.2805
P40957	0.8238	1	0.3471	2	0.1816	1	0.1762	1	NA	0.5305
P38112	0.4994	4	0.2484	1	0.1966	2	0.2083	2	NA	0.6876
P32266	0.3553	2	0.1158	1	0.1078	1	0.1078	1	NA	0.474
P10659	0.1545	0	0.0366	0	0	0	0.0524	0	NA	0.3278
P19358	0.1589	0	0.0182	0	0	0	0.0625	0	NA	0.3481
P32491	0.3557	2	0.2194	1	0.2016	1	0.2174	1	NA	0.3968
Q03081	0.7458	2	0.2825	0	0	0	0.1751	0	TRU	0.4116
P38069	0.2982	3	0.0218	0	0	0	0.0519	0	NA	0.2191
P53311	0.2123	0	0.0137	0	0	0	0.1096	0	NA	0.1502
P30952	0.1516	1	0.0343	0	0	0	0.0325	0	NA	0.2057
P38857	0.1783	0	0.031	0	0	0	0.1085	0	NA	0.1195
Q08818	0.2139	1	0.0376	0	0	0	0.0491	0	NA	0.1257
P25847	0.2541	1	0.0187	0	0	0	0.0187	0	NA	0.1438
Q06568	0.9365	1	0.3122	0	0.1958	0	0.2063	0	NA	0.4827
P46955	0.5223	2	0.2077	0	0.1869	0	0.2107	1	NA	0.8535
Q92317	0.5342	1	0.1781	0	0.3082	0	0.3562	0	NA	0.6744
Q12207	0.2948	0	0	0	0	0	0.1387	0	NA	0.2015
P34077	0.2586	2	0.0441	0	0	0	0.0632	0	NA	0.3105
P39705	0.9536	1	0.692	3	0.6197	3	0.6067	4	NA	0.9704
P40064	0.2185	2	0.0352	1	0.0532	1	0.0611	1	NA	0.3471
P39952	0.3781	2	0.0746	0	0	0	0.1318	0	NA	0.2729
P26263	0.1243	0	0.0213	0	0	0	0.0337	0	NA	0.1405
P10963	0.1821	0	0.0619	0	0	0	0.0656	0	NA	0.4273
P25644	0.5741	2	0.4774	3	0.4334	3	0.4045	4	NA	0.9708
P40960	0.394	1	0.2852	2	0.2289	2	0.1745	1	NA	0.302
P50091	0.8125	1	0.3576	0	0	0	0.2257	0	NA	0.61
P06738	0.1829	3	0.0466	0	0.041	0	0.0455	0	NA	0.1644
P32838	0.2364	1	0.0516	0	0	0	0.0897	0	NA	0.1847
P07283	0.1457	0	0.0276	0	0	0	0.0236	0	NA	0.1388
P15938	0.3361	3	0.1569	1	0.1531	2	0.1522	2	NA	0.2432
P53131	0.2308	2	0.1551	1	0.1421	1	0.1499	1	NA	0.2142
P33334	0.2296	4	0.0899	1	0.0651	2	0.0738	2	NA	0.3806
P23638	0.2403	0	0.0543	0	0	0	0.093	0	NA	0.1346
P53174	0.27	1	0.1392	0	0.2278	1	0.2447	1	NA	0.4295
P09232	0.4646	2	0.3024	1	0.2472	1	0.2535	1	NA	0.7219
Q05911	0.166	0	0	0	0	0	0.027	0	NA	0.1595
P25339	0.616	2	0.5372	5	0.3694	4	0.357	3	NA	0.9882
P10355	0.1811	0	0.0118	0	0	0	0.0354	0	NA	0.1529
P43606	0.3201	2	0.1608	2	0.1785	2	0.2021	2	NA	0.742
P19541	0.5874	1	0.4058	2	0.4036	2	0.3363	2	TRU	0.9726
P23796	0.2612	1	0.0078	0	0	0	0.0448	0	NA	0.1623
P12687	0.5822	4	0.0647	0	0	0	0.0943	0	NA	0.2829
P53196	0.1559	0	0.012	0	0	0	0.0192	0	NA	0.121
P20433	0.7511	1	0.4977	1	0.4072	2	0.4344	2	NA	0.5621
P39516	0.5072	1	0.3696	0	0.3333	0	0.3333	0	NA	0.2927
P07703	0.1761	0	0.0418	0	0	0	0.0746	0	NA	0.2241
Q04225	0.4305	2	0.3836	1	0.2524	2	0.2603	1	NA	0.4728
P15891	0.7618	1	0.7534	1	0.7399	1	0.7179	2	NA	0.9952

Q12482	0.2561	2	0.0299	0	0	0	0.0388	0	NA	0.2297
P53730	0.0526	0	0	0	0	0	0.0163	0	NA	0.1046
Q12342	0.3116	1	0.1548	1	0.1894	2	0.2016	2	NA	0.5676
Q06820	0.6891	3	0.4715	2	0.3938	3	0.3782	2	NA	0.9816
P47822	0.5143	1	0.1214	0	0	0	0.1214	0	NA	0.1765
P24279	0.3955	3	0.2729	2	0.2472	2	0.2719	2	NA	0.955
P50945	0.1197	0	0	0	0	0	0.0684	0	NA	0.1064
P38341	0.1604	0	0.0094	0	0	0	0.0849	0	NA	0.1045
P08465	0.3066	1	0.1276	1	0.144	1	0.1811	1	NA	0.5674
P53159	0.5142	1	0.1292	0	0	0	0.1318	0	NA	0.3897
Q03455	0.2403	2	0.076	1	0.0994	1	0.1229	1	NA	0.4708
Q03677	0.1955	0	0.0223	0	0	0	0.0168	0	NA	0.1194
P32500	0.258	1	0.0855	0	0	0	0.1038	0	NA	0.2674
P40460	0.6918	4	0.1751	1	0.178	1	0.178	1	NA	0.4958
P38795	0.098	0	0.0112	0	0	0	0.0126	0	NA	0.1254
Q02197	0.191	0	0	0	0	0	0.015	0	NA	0.1434
Q07896	0.4374	2	0.2896	1	0.264	1	0.2941	1	NA	0.3581
P38325	0.3209	1	0.1716	0	0.2015	0	0.2164	0	NA	0.1554
P25366	0.3094	2	0.058	0	0	0	0.1133	0	NA	0.2055
P50946	0.3319	1	0.2605	1	0.2353	1	0.2227	1	NA	0.3957
P06776	0.1407	0	0.0152	0	0	0	0.0152	0	NA	0.1261
P32567	0.681	3	0.5325	5	0.4292	4	0.4292	4	NA	0.9871
P05066	0.2389	2	0.023	0	0	0	0.0584	0	NA	0.2164
P34221	0.4722	1	0.2051	2	0.2436	2	0.2286	2	NA	0.4494
P41812	0.4034	2	0.1851	1	0.1383	0	0.1657	1	NA	0.5618
P07274	0.0476	0	0	0	0	0	0	0	NA	0.1504
P32523	0.334	0	0.0577	0	0	0	0.0775	0	NA	0.2488
Q12211	0.3971	3	0.2592	2	0.25	2	0.2518	2	NA	0.3706
P32606	0.4725	3	0.1988	0	0	0	0.1562	0	NA	0.4296
Q9ZZW2	0	0	0	0	0	0	0	0	NA	0.1932
P03962	0.1536	0	0.0037	0	0	0	0.0637	0	NA	0.1395
P49723	0.2087	1	0.0029	0	0	0	0.0899	0	NA	0.1205
P05317	0.2212	1	0.0865	0	0.141	1	0.141	1	NA	0.2611
Q04599	0.4105	1	0.1158	0	0	0	0.1333	0	NA	0.4234
P36519	0.3767	1	0.1199	0	0	0	0.137	0	NA	0.3991
P43588	0.3824	1	0.1144	0	0	0	0.1634	0	NA	0.2622
Q06103	0.31	1	0.0303	0	0	0	0.042	0	NA	0.133
P04051	0.1719	2	0.0438	0	0	0	0.0418	0	NA	0.3909
P40571	0.3819	0	0.25	0	0	0	0.25	0	NA	0.3228
Q03691	0.2812	1	0.1602	0	0.1445	1	0.1484	0	NA	0.3317
P32316	0.1578	0	0.0875	0	0	0	0.076	0	NA	0.1664
P47019	0.8514	1	0.5086	1	0.4286	1	0.4571	1	NA	0.5884
P47771	0.1304	1	0.0296	0	0	0	0.0119	0	NA	0.1396
P40350	0.1317	0	0.006	0	0	0	0.006	0	NA	0.1664
P38971	0.1588	1	0.0716	0	0.075	0	0.075	0	NA	0.1826
Q12674	0.349	6	0.1612	2	0.1232	1	0.1492	2	NA	0.5317
Q12527	0.4542	6	0.1222	0	0.0891	0	0.0993	0	NA	0.5998
P38696	0.1068	0	0	0	0	0	0.0286	0	NA	0.1387
P38110	0.1328	3	0.0057	0	0	0	0.009	0	NA	0.1216
P36062	0.3801	3	0.3483	3	0.3092	4	0.3078	3	NA	0.8717
P38176	0.1481	0	0.0305	0	0	0	0.0675	0	NA	0.2198
P48524	0.5318	7	0.293	2	0.2664	2	0.2848	4	NA	0.9689

Q03783	0.2947	0	0	0	0	0	0	0	NA	0.2173
Q06338	0.4064	2	0.1837	1	0.2297	1	0.258	1	NA	0.4294
Q07660	0.2667	5	0.0631	0	0.1164	0	0.1191	0	NA	0.3461
P12709	0.1661	0	0.0108	0	0	0	0.0271	0	NA	0.1892
P53746	0.1586	1	0.0626	1	0.0542	1	0.0584	1	NA	0.155
P38229	0.8279	4	0.4194	5	0.3787	3	0.3834	2	NA	0.9839
Q03833	0.7092	2	0.5403	5	0.3803	5	0.3949	4	TRU	0.9935
P38138	0.1394	1	0.0147	0	0	0	0.0115	0	NA	0.1884
Q03786	0.1658	0	0.0363	0	0	0	0.0466	0	NA	0.1481
P41546	0.8487	2	0.5378	2	0.3866	2	0.4202	2	TRU	0.7361
P06775	0.2206	1	0.0912	1	0	0	0.0962	1	NA	0.3694
Q08109	0.314	2	0.2269	0	0.0853	1	0.1216	1	NA	0.2375
P29295	0.4312	1	0.3279	1	0.3401	1	0.3421	1	NA	0.4093
P32874	0.1447	3	0.0084	0	0	0	0.0207	0	NA	0.2588
P09435	0.4607	4	0.2773	2	0.1402	1	0.1418	1	NA	0.4887
P39533	0.251	1	0.0976	1	0	0	0.0646	1	NA	0.1869
Q08361	0.4266	1	0.049	0	0	0	0.1049	0	NA	0.3081
Q04371	0.1915	1	0.0213	0	0	0	0.0511	0	NA	0.1623
P13090	0.1421	1	0.0277	0	0	0	0.0498	0	NA	0.1175
P32453	0.3742	1	0.1415	0	0	0	0.1258	0	NA	0.3244
P38891	0.1603	1	0.0611	0	0	0	0.0763	0	NA	0.1902
O94143	0.3922	0	0	0	0	0	0.098	0	NA	0.1567
P38636	0.2192	0	0	0	0	0	0.1781	0	NA	0.1181
Q05029	0.2251	2	0.047	0	0.0552	0	0.0691	0	NA	0.4148
P53858	0.9047	2	0.6827	7	0.5392	4	0.5179	4	NA	0.9976
P47818	0.3758	1	0.1646	0	0.2298	1	0.2547	1	NA	0.804
P43610	0.3904	4	0.2075	1	0.2134	2	0.204	2	NA	0.3421
Q07821	0.548	1	0.264	0	0.24	0	0.264	1	NA	0.6928
P25583	0.2657	1	0.1373	1	0.1164	0	0.1194	0	TRU	0.2327
P47156	0.1456	1	0.0082	0	0	0	0.0398	0	NA	0.1715
P33550	0.1553	1	0.0118	0	0	0	0.0329	0	NA	0.1312
P50107	0.2791	0	0.0828	0	0	0	0.0675	0	NA	0.2804
Q02796	1	1	0.7801	1	0.7289	1	0.741	1	NA	0.9991
P47084	0.2389	1	0.0019	0	0	0	0.0173	0	NA	0.1199
Q03104	0.4366	3	0.0039	0	0	0	0.0117	0	NA	0.2646
P12945	0.2881	2	0.0609	1	0.0691	1	0.0726	1	NA	0.1978
P40078	0.6475	1	0.387	1	0.3218	1	0.341	1	NA	0.7633
P07991	0.0684	0	0	0	0	0	0	0	NA	0.1861
Q02803	0.2295	0	0	0	0	0	0.0103	0	NA	0.1653
Q3E833	0.3636	0	0.0341	0	0	0	0.1364	0	NA	0.1638
Q08966	0.7033	2	0.4776	4	0.3963	2	0.3902	2	NA	0.5534
Q12447	0.2094	0	0	0	0	0	0	0	NA	0.1739
P53343	0.1833	0	0	0	0	0	0.025	0	NA	0.0927
P40585	0.1613	0	0	0	0	0	0.0403	0	NA	0.0997
P35994	0.1545	0	0	0	0	0	0.0325	0	NA	0.102
Q04264	0.224	3	0.0948	1	0.0908	1	0.0885	1	NA	0.221
P80667	0.5518	2	0.2254	1	0.2306	1	0.2668	1	NA	0.952
Q12144	0.0366	0	0	0	0	0	0	0	NA	0.1009
P52867	0.109	0	0.0215	0	0	0	0.035	0	NA	0.1658
P21372	0.543	4	0.2509	3	0.2603	2	0.2909	2	NA	0.5962
P39006	0.29	1	0.052	0	0	0	0.056	0	NA	0.2533
P31374	0.5317	6	0.323	5	0.281	4	0.2861	3	NA	0.9686

P27616	0.2451	1	0.1013	0	0	0	0.1242	0	NA	0.1417
P47148	0.1519	1	0.0742	0	0	0	0.0883	0	NA	0.1745
Q9ZZX8	0.1698	0	0	0	0	0	0.0755	0	NA	0.0951
P38270	0.3169	2	0.0378	0	0	0	0.061	0	NA	0.1555
P39981	0.1979	1	0.0417	0	0	0	0.0917	1	NA	0.2079
P38822	0.5419	5	0.357	1	0.188	1	0.2196	1	NA	0.5319
P53890	0.8125	3	0.7321	3	0.6205	3	0.596	3	NA	0.9899
P43571	0.137	0	0.0097	0	0	0	0.0107	0	NA	0.1348
P32873	0.6605	3	0.4681	6	0.3892	7	0.3785	6	NA	0.979
P28495	0.1828	0	0	0	0	0	0.0448	0	NA	0.117
P38695	0.2804	3	0.1081	1	0.1284	1	0.1419	1	NA	0.3266
P53901	0.687	1	0.5526	1	0.5355	1	0.4866	1	NA	0.9801
A0A023PXB	0.1961	0	0	0	0	0	0	0	NA	0.1228
P40026	0.3664	2	0.181	1	0.1767	1	0.1961	1	NA	0.2587
P53086	0.405	4	0.2137	1	0.1578	1	0.1727	1	NA	0.423
P47055	0.2603	1	0.0731	0	0	0	0.0594	0	NA	0.1148
P53281	0.7386	1	0.5436	2	0.5104	1	0.556	1	NA	0.9937
Q12296	0.5099	2	0.4235	1	0.4278	2	0.4108	3	NA	0.9231
P25491	0.6161	3	0.1222	0	0.1296	1	0.1467	1	NA	0.6859
P53035	0.8979	2	0.6675	3	0.5236	3	0.5209	3	TRU	0.9945
P30665	0.3955	4	0.2111	1	0.1929	1	0.2036	1	NA	0.5491
P34072	0.8425	3	0.8031	3	0.6901	5	0.6524	5	NA	0.9987
P36044	0.41	4	0.1231	0	0.1265	1	0.1528	1	NA	0.221
P39107	0.2532	1	0.0076	0	0	0	0.0203	0	NA	0.1343
P46982	0.2526	0	0.0119	0	0	0	0.0375	0	NA	0.1448
P53129	0.3696	1	0.0994	0	0	0	0.0978	0	NA	0.3219
P43563	0.3728	1	0.2857	1	0.3206	1	0.3171	1	NA	0.3422
P38694	0.1475	0	0.0109	0	0	0	0.0217	0	NA	0.1625
Q08925	0.3464	2	0.2794	2	0.2026	2	0.1928	1	NA	0.3589
Q06504	0.3128	1	0.0513	0	0	0	0.1231	0	NA	0.1532
P47077	0.3258	3	0.1021	1	0.1171	2	0.1246	2	NA	0.2655
P49687	0.4753	2	0.2923	4	0.268	5	0.2802	4	NA	0.9591
P38302	0.7143	2	0.6214	0	0	0	0.3143	0	NA	0.271
P38340	0.2716	0	0.0819	0	0	0	0.1293	0	NA	0.1486
P31378	0.2381	2	0.0376	0	0	0	0.0602	0	NA	0.1827
Q08202	0.4268	1	0.1179	0	0	0	0.2154	1	TRU	0.5953
P54784	0.3928	3	0.2035	1	0.2024	2	0.2079	1	NA	0.2751
P38219	0.2132	0	0.0279	0	0	0	0.033	0	NA	0.1506
P40345	0.2375	1	0.1437	1	0.112	1	0.115	1	NA	0.3474
Q12477	0.3454	1	0.1217	0	0	0	0.0625	0	NA	0.163
Q02785	0.1886	2	0.047	1	0.0443	1	0.0443	1	NA	0.2528
P0CE87	0.1341	0	0.0061	0	0	0	0.061	0	NA	0.1005
P40091	0.7905	3	0.5357	3	0.4024	2	0.4262	2	NA	0.9431
P12383	0.3961	3	0.2107	2	0.2182	3	0.2285	3	TRU	0.76
P52553	0.8509	1	0.2544	0	0	0	0.1579	0	NA	0.2535
Q04370	0.2481	1	0.0526	0	0	0	0.1153	0	NA	0.4106
P42934	0.191	2	0.0435	0	0.0672	1	0.083	1	NA	0.3158
Q04364	0.2588	1	0.0292	0	0	0	0.0759	0	NA	0.146
Q12252	0.3098	2	0.1433	1	0.1483	1	0.1594	1	NA	0.7382
Q06494	0.2319	0	0.0145	0	0	0	0.0319	0	NA	0.1638
Q07951	0.3073	0	0.0782	0	0	0	0.1341	0	NA	0.3684
P32903	0.225	0	0	0	0	0	0	0	NA	0.0248

Q06892	0.2802	1	0.0845	0	0	0	0.1208	0	NA	0.2868
Q07807	0.6507	2	0.4403	2	0.3754	3	0.3595	3	NA	0.98
P06839	0.1967	3	0.0643	0	0.0707	1	0.063	1	NA	0.2083
P32781	0.3563	0	0	0	0	0	0.2184	0	NA	0.2501
P04076	0.2009	0	0.0173	0	0	0	0.0518	0	NA	0.2231
P53755	0.4601	3	0.2127	0	0.1779	1	0.1943	1	NA	0.5964
Q08732	0.643	2	0.6166	4	0.5441	3	0.5336	3	NA	0.9864
P53688	0.2811	1	0.1162	1	0.0838	0	0.0946	0	NA	0.1447
P50079	0.3348	1	0.096	0	0	0	0.1496	0	NA	0.2989
P53685	0.161	1	0.0398	0	0	0	0.0775	0	NA	0.2028
P39730	0.507	2	0.4202	2	0.4012	1	0.4002	2	NA	0.6578
P39522	0.2308	1	0.0701	0	0	0	0.041	0	NA	0.1516
Q08630	0.1731	0	0	0	0	0	0.125	0	NA	0.1691
P47046	0.7518	2	0.5195	6	0.444	5	0.382	3	NA	0.9806
Q12280	0.3064	5	0.0515	0	0.0548	0	0.0756	0	NA	0.2469
P36007	0.1472	0	0.0153	0	0	0	0.0491	0	NA	0.1667
P32585	0.3779	1	0.1629	1	0.1792	1	0.1824	1	NA	0.4892
Q08268	0.1178	1	0.0339	0	0	0	0.0539	0	NA	0.1232
Q06630	0.2257	0	0.0752	0	0	0	0.146	0	NA	0.3642
Q12525	0.1327	0	0.0216	0	0	0	0.037	0	NA	0.1158
Q03218	0.3314	2	0.1431	0	0.1176	0	0.1196	0	NA	0.3494
P35724	0.6202	4	0.4923	5	0.4665	4	0.4427	6	NA	0.9891
P40029	0.2446	0	0.0489	0	0	0	0.1957	0	NA	0.4376
P33748	0.9702	1	0.8111	5	0.6207	4	0.6222	4	TRU	0.9983
P21339	0.7194	4	0.5708	5	0.5286	5	0.4591	7	NA	0.994
P34246	0.2885	1	0.0952	0	0	0	0.1176	0	NA	0.2452
Q06489	0.146	0	0.0195	0	0	0	0.0268	0	NA	0.1421
Q12200	0.1111	0	0	0	0	0	0.0094	0	NA	0.1337
P53164	0.0755	0	0	0	0	0	0	0	NA	0.1206
P46970	0.124	1	0.02	0	0	0	0.0372	0	NA	0.2032
Q04121	0.2717	2	0.128	1	0.1359	1	0.1327	1	NA	0.2987
P39720	0.3496	4	0.1547	1	0.1442	2	0.148	2	TRU	0.4745
P0CE93	0.1833	0	0	0	0	0	0.025	0	NA	0.0875
P37304	0.5831	1	0.4916	1	0.4783	3	0.4506	5	NA	0.9159
P39545	0.4	0	0	0	0	0	0.2364	0	NA	0.0878
P28795	0.4218	2	0.034	0	0	0	0.0975	0	NA	0.2177
Q03880	0.3934	1	0.3033	1	0.4016	1	0.418	1	NA	0.3871
P38291	0.4357	1	0.2286	0	0.15	0	0.2071	0	NA	0.2629
P32379	0.3231	1	0.0731	0	0	0	0.1154	0	NA	0.1888
Q12417	0.255	1	0.0466	0	0	0	0.0776	0	NA	0.1799
P53037	0.4148	5	0.2074	3	0.174	3	0.1837	3	NA	0.6889
Q12459	0.9556	2	0.6203	4	0.4828	3	0.5659	4	NA	1
P47029	0.718	4	0.5172	4	0.4646	3	0.4446	4	NA	0.9966
Q05955	0.1866	0	0.0041	0	0	0	0.0507	0	NA	0.1507
P38065	0.2137	2	0.042	0	0	0	0.0732	0	NA	0.2941
P49089	0.1329	0	0.0122	0	0	0	0.028	0	NA	0.1798
P22135	0.1938	0	0.0523	0	0	0	0.0708	0	NA	0.1189
P53062	0.2183	1	0.0051	0	0	0	0.132	0	NA	0.1132
P41815	0.2401	1	0.0778	0	0	0	0.0911	0	NA	0.2995
P47170	0.3239	4	0.1446	2	0.1269	1	0.1294	1	NA	0.7097
P13185	0.6269	4	0.5677	4	0.5075	4	0.5009	6	NA	0.9894
Q06346	0.1946	0	0	0	0	0	0.0271	0	NA	0.1373

Q04781	0.1287	1	0.0019	0	0	0	0.0147	0	NA	0.1489
P03879	0.1097	1	0.0063	0	0	0	0.0235	0	NA	0.1223
P32490	0.5079	2	0.3268	2	0.2972	2	0.2874	2	NA	0.6159
Q07684	0.5022	2	0.3799	2	0.3734	2	0.3384	2	NA	0.8694
P40577	0.7962	2	0.6386	3	0.5299	3	0.4212	2	NA	0.9698
Q02046	0.1437	0	0	0	0	0	0	0	NA	0.1081
P25293	0.6619	4	0.3717	2	0.3285	2	0.3333	2	NA	0.5106
P39744	0.4803	2	0.2549	4	0.2831	3	0.2775	4	NA	0.4781
P11632	0.7419	2	0.7204	0	0.6344	1	0.6022	0	NA	0.533
Q06144	0.3333	1	0.1759	0	0.2824	1	0.3056	1	NA	0.1508
P46964	0.1769	0	0	0	0	0	0.1231	0	NA	0.0998
P39081	0.4984	2	0.2173	2	0.1917	1	0.2045	1	NA	0.6377
P33200	0.2869	4	0.0994	1	0.1096	1	0.1424	0	TRU	0.3788
P27801	0.1405	0	0	0	0	0	0.0098	0	NA	0.1249
P40187	0.5539	4	0.4219	3	0.2472	1	0.2546	1	NA	0.547
Q03178	0.6598	1	0.1672	0	0	0	0.0557	0	NA	0.2554
Q04004	0.4739	2	0.1652	0	0	0	0.187	0	NA	0.224
P53833	0.1128	0	0	0	0	0	0.0769	0	NA	0.1235
P36110	0.541	1	0.3769	1	0.3404	1	0.3404	1	NA	0.9612
P21242	0.3542	1	0.2153	1	0.1806	1	0.2049	1	NA	0.2277
P22141	0.1919	0	0.0051	0	0	0	0.0303	0	NA	0.1519
P39551	0.245	1	0.0705	0	0.1376	1	0.1711	1	NA	0.3212
Q12488	0.1587	0	0	0	0	0	0	0	NA	0.1089
Q12335	0.202	0	0.0303	0	0	0	0.0758	0	NA	0.2377
P38876	0.2316	0	0	0	0	0	0.0947	0	NA	0.2304
P25635	0.2026	2	0.104	1	0.1138	1	0.1268	1	NA	0.2592
P38972	0.204	4	0.0515	0	0	0	0.0457	0	NA	0.5835
P40086	0.2222	0	0	0	0	0	0.0288	0	NA	0.1285
P53959	0.3611	3	0.1549	2	0.1335	2	0.143	2	NA	0.3389
P53053	0.0868	0	0	0	0	0	0	0	NA	0.1022
P69850	0.7234	1	0.3191	0	0	0	0.4043	0	NA	0.5628
Q03957	0.4205	2	0.3693	2	0.3617	2	0.358	2	NA	0.4943
P24783	0.3297	2	0.2729	2	0.2418	2	0.2418	2	NA	0.372
P25615	0.2646	2	0.0601	1	0.0825	1	0.0842	1	NA	0.1776
Q06053	0.3189	3	0.2036	3	0.1931	3	0.1976	3	NA	0.392
Q8TGJ3	0.1111	0	0	0	0	0	0	0	NA	0.1027
P39002	0.1398	1	0.0418	0	0.0461	0	0.0548	0	NA	0.1278
P36016	0.3167	2	0.076	1	0.0579	1	0.059	1	NA	0.1813
P07702	0.1911	1	0.0302	0	0	0	0.0374	0	NA	0.4796
P38851	0.2207	2	0	0	0	0	0.0041	0	NA	0.1782
P38999	0.139	0	0	0	0	0	0.0157	0	NA	0.1451
Q07923	0.1361	0	0	0	0	0	0	0	NA	0.1408
Q06406	0.3372	0	0.0349	0	0	0	0.1744	0	NA	0.1149
P47093	0.4037	1	0	0	0	0	0.0917	0	NA	0.1318
P53237	0.1777	0	0	0	0	0	0	0	NA	0.1165
P38304	0.4709	1	0.1614	0	0	0	0.1883	0	NA	0.5029
Q01846	0.3345	5	0.0639	0	0	0	0.0736	0	NA	0.2794
P36013	0.2571	1	0.1375	0	0.1001	1	0.1271	1	NA	0.1843
P41948	0.2124	1	0.0982	1	0.1303	1	0.1283	1	NA	0.1566
P34162	0.1286	0	0.0095	0	0	0	0.0476	0	NA	0.2579
P41821	0.1569	0	0.0164	0	0	0	0.0493	0	NA	0.2119
P40457	0.8946	7	0.2335	3	0.0989	2	0.1144	1	NA	0.6914

P40573	0.9786	2	0.4599	0	0	0	0.2246	0	TRU	0.4827
Q07980	0.4288	3	0.2518	2	0.1669	1	0.1698	0	NA	0.5252
P40562	0.3535	3	0.1299	0	0.1168	1	0.141	1	NA	0.2576
Q06106	0.5479	5	0.3777	4	0.3551	4	0.3303	2	NA	0.8343
P36010	0.1242	0	0	0	0	0	0.0458	0	NA	0.1375
Q12460	0.3274	2	0.1667	1	0.1508	1	0.1409	1	NA	0.1923
P32494	0.7821	4	0.6382	4	0.5456	4	0.5171	3	NA	0.9838
Q12066	0.4277	1	0.219	1	0.2376	1	0.2376	1	NA	0.3611
P04147	0.5338	3	0.2946	1	0.201	2	0.1872	1	NA	0.5726
P00950	0.3684	1	0.0405	0	0	0	0.1255	0	NA	0.1826
Q03760	0.2216	0	0	0	0	0	0.0419	0	NA	0.1341
P53218	0.2975	0	0	0	0	0	0.0443	0	NA	0.136
P32264	0.1963	0	0.0304	0	0	0	0.0514	0	NA	0.2979
P40968	0.3692	1	0.2484	1	0.211	1	0.2308	1	NA	0.397
P49704	0.5283	2	0.2611	2	0.1964	1	0.1721	0	NA	0.5905
P20457	0.2992	1	0.0379	0	0	0	0.053	0	NA	0.1565
Q08641	0.6131	1	0.5541	1	0.5143	2	0.4889	2	NA	0.9095
P33309	0.2798	1	0.013	0	0	0	0.0337	0	NA	0.1321
P09624	0.1423	0	0.01	0	0	0	0.006	0	NA	0.1198
Q05902	0.1712	1	0.0258	0	0	0	0.0485	0	NA	0.2003
Q03016	0.5972	7	0.4224	5	0.3707	4	0.3856	6	NA	0.9947
P53154	0.1411	1	0.0089	0	0	0	0.0464	0	NA	0.188
Q04806	0.1448	0	0.0656	0	0	0	0.0984	0	NA	0.1608
P48239	0.1517	0	0	0	0	0	0.0421	0	NA	0.1899
P41921	0.1573	0	0.0062	0	0	0	0.0248	0	NA	0.1326
Q05580	0.5618	4	0.3631	2	0.3271	2	0.3709	2	NA	0.9846
P49775	0.3981	1	0.267	1	0	0	0.1893	0	NA	0.215
Q04178	0.3167	1	0.1041	0	0	0	0.1131	0	NA	0.1769
P0CY07	0.4229	2	0.0114	0	0	0	0.04	0	NA	0.111
P40037	0.1473	0	0	0	0	0	0	0	NA	0.1106
P34167	0.7385	2	0.7477	1	0.6651	2	0.6628	2	NA	0.9898
P32481	0.2865	2	0.1689	1	0.1727	1	0.1765	1	NA	0.2328
P23301	0.4968	1	0.2611	0	0	0	0.2229	0	NA	0.6954
P28834	0.1806	1	0.05	0	0	0	0.0583	0	NA	0.2187
P25626	0.2899	1	0	0	0	0	0.071	0	NA	0.1394
P41939	0.1383	0	0.0243	0	0	0	0.0413	0	NA	0.1197
P39003	0.1912	1	0.0298	0	0	0	0.0825	0	NA	0.1289
Q12271	0.3514	1	0.2141	1	0.2096	1	0.2033	1	NA	0.4552
P32488	0.8994	2	0.6036	3	0.6361	3	0.6095	2	NA	0.9941
P40504	0.1567	0	0	0	0	0	0.0135	0	NA	0.1315
Q03579	0.2267	0	0.0133	0	0	0	0.0867	0	NA	0.1527
P40484	0.3758	1	0.414	1	0	0	0.3376	1	NA	0.4107
P32829	0.5173	3	0.315	3	0.2991	2	0.3006	2	NA	0.5592
P38172	0.1667	0	0.0222	0	0	0	0.0111	0	NA	0.1766
P07266	0.0882	0	0	0	0	0	0	0	NA	0.131
Q03079	0.401	1	0.0048	0	0	0	0.0097	0	NA	0.1176
P43619	0.1797	0	0	0	0	0	0.0203	0	NA	0.125
P40215	0.1696	0	0.0321	0	0	0	0.0339	0	NA	0.1185
P53914	0.3475	4	0.1231	1	0.0909	1	0.1051	1	NA	0.3285
Q03429	0.4878	1	0.5285	1	0	0	0.4553	0	NA	0.5028
Q12514	0.7714	2	0.4375	1	0.3714	1	0.3625	1	NA	0.7961
Q12143	0.2593	0	0.1435	0	0	0	0.1481	0	NA	0.3521

Q12311	0.2914	2	0.0668	0	0	0	0.0695	0	NA	0.392
Q05541	0.4158	2	0.0891	0	0	0	0.1221	0	NA	0.5049
P43124	0.5498	4	0.4104	3	0.3433	3	0.3582	2	NA	0.7996
P13259	0.5118	2	0.4292	2	0.4387	2	0.4387	2	NA	0.7843
P17967	0.205	1	0.046	0	0.0556	0	0.0594	0	NA	0.1272
P53900	0.6279	0	0.1395	0	0	0	0.1318	0	NA	0.1443
Q07418	0.9094	2	0.8216	2	0.6667	3	0.6023	3	NA	0.9792
P39108	0.0773	0	0.0107	0	0	0	0.0293	0	NA	0.2492
Q05637	0.4904	1	0.0096	0	0	0	0.2212	0	NA	0.1537
P25578	0.1497	0	0.0038	0	0	0	0.0173	0	NA	0.148
Q08108	0.1924	1	0.0335	0	0	0	0.0583	0	NA	0.3856
P33775	0.202	1	0.0563	0	0	0	0.0722	0	NA	0.2075
Q99216	0.4964	1	0.3978	2	0.3358	1	0.3686	1	NA	0.461
Q02256	0.4011	1	0.0467	0	0	0	0.1264	0	NA	0.1932
P40508	0.7	3	0.4781	2	0.4594	1	0.4562	1	NA	0.9101
P06777	0.3755	4	0.1645	2	0.1318	2	0.1445	2	NA	0.295
P40474	0.1753	1	0.0129	0	0	0	0.0683	0	NA	0.1624
Q04779	0.5965	2	0.3056	2	0.1871	1	0.2325	1	NA	0.7131
P31373	0.165	1	0.0635	0	0	0	0.0838	0	NA	0.125
P19158	0.2553	5	0.1082	3	0.1117	2	0.1205	3	NA	0.885
P36115	0.787	2	0.7236	2	0.6878	2	0.6602	4	NA	0.9986
P17119	0.6612	4	0.1838	1	0.1152	1	0.1276	1	NA	0.3355
P32895	0.2904	1	0.1148	0	0	0	0.0749	0	NA	0.1701
P06242	0.1144	0	0.0294	0	0	0	0.0654	0	NA	0.1145
P32487	0.1866	1	0.1195	1	0	0	0.0687	0	NA	0.1541
Q07508	0.41	0	0.092	0	0	0	0.1149	0	NA	0.2617
P03873	0.1868	0	0	0	0	0	0.0165	0	NA	0.1061
P38880	0.1813	1	0.0138	0	0	0	0.0432	0	NA	0.1901
P38782	0.4339	1	0.322	1	0.278	1	0.3017	1	NA	0.7939
P53390	0.1309	1	0.09	1	0.1002	1	0.1022	1	NA	0.2134
P36112	0.6444	2	0.0907	0	0	0	0.0463	0	NA	0.2319
P05694	0.1773	1	0.0065	0	0	0	0.0169	0	NA	0.1365
P53157	0.2154	0	0.0615	0	0	0	0.1462	0	NA	0.1262
P40567	0.3333	0	0.1622	0	0	0	0.2613	0	NA	0.1016
P08536	0.1155	0	0.0235	0	0	0	0.0157	0	NA	0.1319
P28737	0.2845	1	0.0249	0	0	0	0.0829	0	NA	0.2017
Q03834	0.3333	1	0.2319	1	0.2391	1	0.2383	2	NA	0.389
P38335	0.5865	5	0.5288	1	0.4352	3	0.4395	3	NA	0.9785
P47095	0.1393	0	0.0123	0	0	0	0.0656	0	NA	0.1288
P40959	0.3659	1	0.2133	2	0.1722	1	0.18	1	NA	0.2897
Q12493	0.6723	2	0.0672	0	0	0	0.0714	0	NA	0.2333
P32860	0.2852	2	0.0547	0	0	0	0.082	0	NA	0.1271
P08466	0.3678	0	0.0942	0	0	0	0.1003	0	NA	0.3092
P36161	0.1858	1	0.0086	0	0	0	0.0147	0	NA	0.1382
Q06810	0.9167	2	0.5694	2	0.5722	2	0.6111	2	NA	0.9952
Q03028	0.0935	0	0	0	0	0	0	0	NA	0.112
Q02201	0.4085	4	0.154	0	0.1585	1	0.1674	1	NA	0.3426
P38151	0.4092	2	0.1913	1	0.1792	1	0.1792	1	NA	0.2746
P53297	0.8269	4	0.6302	6	0.5014	4	0.4626	4	NA	0.9995
P53248	0.056	0	0	0	0	0	0	0	NA	0.1202
P11491	0.2809	1	0.1166	0	0.0936	0	0.1095	0	NA	0.6478
P19736	0.4547	3	0.1547	0	0.1679	1	0.2	0	NA	0.3643

P31115	0.2014	1	0.1109	0	0	0	0.1267	0	NA	0.174
P32588	0.5276	3	0.5298	3	0.4923	3	0.457	3	NA	0.9796
Q06835	0.1495	0	0	0	0	0	0.0935	0	NA	0.1045
P38623	0.5525	4	0.4049	3	0.4082	2	0.4148	3	NA	0.9147
P21827	0.2427	1	0.1722	1	0.1598	1	0.1701	1	NA	0.273
Q99188	0.5987	1	0.3398	1	0.3366	1	0.3851	1	NA	0.9154
P09938	0.2682	1	0.1429	0	0	0	0.1504	0	NA	0.1306
Q07505	0.1282	0	0.011	0	0	0	0	0	NA	0.1351
P38073	0.4548	5	0.2111	1	0.1774	1	0.1904	2	NA	0.5583
Q05040	0.3786	2	0.2562	2	0.1855	1	0.1683	0	NA	0.2637
P39993	0.2961	4	0.0706	2	0.0651	1	0.0692	1	NA	0.2507
Q04080	0.1067	1	0.0243	0	0	0	0.0637	0	NA	0.1157
Q12438	0.5455	2	0.1472	0	0	0	0.1818	0	NA	0.3462
P37291	0.1386	0	0.0107	0	0	0	0.0171	0	NA	0.1358
P39996	0.4006	1	0.2018	1	0.2433	1	0.27	1	NA	0.6581
P14064	0.9404	2	0.6661	3	0.5181	2	0.5379	2	TRU	0.9677
P40079	0.8768	2	0.6415	2	0.5406	2	0.535	2	NA	0.928
P25369	0.4011	2	0.2797	1	0.2938	1	0.3249	1	NA	0.8795
Q12230	0.607	3	0.3548	2	0.3167	2	0.3284	2	NA	0.6597
P38203	0.1474	0	0.0316	0	0	0	0.0842	0	NA	0.106
P07866	0.6564	4	0.3951	4	0.2787	3	0.2892	3	NA	0.9828
P33308	0.9463	2	0.5034	1	0.3893	1	0.3893	1	NA	0.5121
P23493	0.9971	2	0.5723	2	0.6077	2	0.6431	2	NA	0.9975
P40081	0.1629	0	0	0	0	0	0.0449	0	NA	0.1441
P25573	0.3813	3	0.2254	1	0.2014	1	0.2326	1	NA	0.8683
Q12374	0.2062	1	0	0	0	0	0.013	0	NA	0.123
P25353	0.2992	1	0.1456	1	0.155	2	0.1469	2	NA	0.7726
P32495	0.641	2	0.1795	0	0	0	0.3526	0	NA	0.6407
P34909	0.598	3	0.4702	3	0.3918	4	0.3629	2	TRU	0.9653
Q06162	0.3203	0	0.0131	0	0	0	0	0	NA	0.1684
Q99380	0.3611	0	0	0	0	0	0.3611	0	NA	0.0248
P33767	0.1744	0	0.0023	0	0	0	0.0395	0	NA	0.1507
P32833	0.5694	2	0.3952	1	0.3629	1	0.3806	1	NA	0.6669
P40459	0.0971	0	0.0032	0	0	0	0.0032	0	NA	0.1314
P40335	0.4802	3	0.1847	0	0.1398	0	0.1715	0	NA	0.7057
Q03306	0.569	3	0.441	3	0.3608	2	0.3675	2	NA	0.8963
Q06216	0.5849	3	0.216	1	0.1898	1	0.2346	2	NA	0.6906
Q06644	0.1163	0	0.003	0	0	0	0	0	NA	0.1339
P37012	0.2004	1	0.0615	0	0	0	0.0773	0	NA	0.3292
P46971	0.1719	1	0.0197	0	0.0367	0	0.0381	0	NA	0.231
Q12017	0.6818	2	0.3427	1	0	0	0.3182	0	NA	0.5865
Q07799	0.185	1	0.0361	0	0	0	0.0391	0	NA	0.1466
P33310	0.1554	1	0.0173	0	0	0	0.0561	0	NA	0.1332
P53051	0.3005	1	0.0475	0	0	0	0.0713	0	NA	0.3046
P53083	0.6688	2	0.2636	1	0.2331	1	0.2549	1	NA	0.4851
P24719	0.1348	1	0	0	0	0	0.0181	0	NA	0.1141
P40578	0.5247	6	0.3765	4	0.2722	4	0.2489	4	TRU	0.8428
Q08176	0.4779	1	0.0354	0	0	0	0.2301	0	NA	0.1189
P38286	0.1527	0	0	0	0	0	0.0259	0	NA	0.1445
Q12083	0.1636	0	0.0014	0	0	0	0.0196	0	NA	0.1291
P39731	0.6228	3	0.0761	0	0	0	0.1107	0	NA	0.2271
P0CX80	1	1	0	0	0	0	0.5082	0	NA	0.4827

P38734	0.0824	0	0	0	0	0	0	0	NA	0.1161
Q12387	0.1884	0	0	0	0	0	0.0101	0	NA	0.1362
P49954	0.2577	0	0.0069	0	0	0	0.0653	0	NA	0.1544
P19262	0.4968	3	0.3305	1	0.2397	1	0.2484	1	NA	0.3861
P54790	0.1916	2	0.026	0	0	0	0.0373	0	NA	0.1562
P38881	0.567	1	0.4268	2	0.4704	2	0.4237	1	NA	0.4776
Q12255	0.3478	1	0.087	0	0.1107	0	0.0988	0	NA	0.2902
P32896	0.507	2	0.3459	2	0.2984	2	0.2854	2	TRU	0.8192
Q06149	0.1912	2	0.0328	0	0.0314	0	0.0399	0	TRU	0.1762
P40975	0.2558	0	0	0	0	0	0	0	NA	0.0248
P28005	0.289	1	0.0462	0	0	0	0.0694	0	NA	0.1287
P21264	0.1068	0	0.021	0	0	0	0.0315	0	NA	0.141
P50109	0.8078	3	0.7622	2	0.7032	2	0.6965	4	NA	0.9992
P38227	0.3498	2	0.1916	1	0.1509	1	0.1582	1	NA	0.2062
P00128	0.1969	0	0	0	0	0	0.1102	0	NA	0.1032
P22289	0.2273	0	0	0	0	0	0.197	0	NA	0.1488
P06779	0.4018	1	0.2513	2	0.2496	3	0.246	3	NA	0.3232
P25301	0.2761	1	0.037	0	0	0	0.0587	0	NA	0.1619
P38086	0.4259	4	0.1555	1	0.0856	1	0.1284	1	NA	0.391
P33323	0.3846	1	0.011	0	0	0	0.0934	0	NA	0.156
P41817	0.7712	2	0.5294	1	0.3497	1	0.3464	1	TRU	0.9447
Q12091	0.2961	1	0.1711	0	0	0	0.2632	0	NA	0.3218
P36108	0.8664	3	0.569	1	0.2026	1	0.2371	1	NA	0.3926
P21182	0.1919	1	0.0354	0	0	0	0.0303	0	NA	0.1324
P32462	0.1073	0	0	0	0	0	0.0137	0	NA	0.1043
P09201	0.1466	0	0.0287	0	0	0	0.0603	0	NA	0.1792
P16151	0.1077	0	0	0	0	0	0	0	NA	0.1082
Q04991	0.1429	1	0	0	0	0	0.0635	0	NA	0.1178
P53260	0.2678	0	0.0383	0	0	0	0.0383	0	NA	0.1963
Q12209	0.2172	1	0.1108	1	0.0948	1	0.1224	1	NA	0.2093
P41818	1	1	0.9476	1	0.9214	3	0.8297	2	NA	0.9649
Q03330	0.3235	1	0.1731	1	0.1595	1	0.1549	1	NA	0.1892
P15442	0.358	5	0.0892	2	0.0772	1	0.0989	1	NA	0.5607
P41911	0.1614	1	0.0432	0	0	0	0.0409	0	NA	0.1352
P53839	0.1714	0	0.06	0	0	0	0.04	0	NA	0.2001
Q03554	0.0507	0	0	0	0	0	0.0217	0	NA	0.1304
P39982	0.1176	0	0	0	0	0	0	0	NA	0.1328
P07172	0.2026	1	0.039	0	0	0	0.0545	0	NA	0.1356
P51979	0.3572	3	0.1466	2	0.1247	2	0.1424	2	NA	0.2506
Q99181	0.1784	0	0	0	0	0	0	0	NA	0.1218
P04806	0.2351	0	0.0804	0	0	0	0.0639	0	NA	0.2296
P38922	0.5242	2	0.4581	1	0.3811	1	0.4053	1	NA	0.8389
P43581	0.1777	1	0	0	0	0	0.1044	0	NA	0.2562
P38800	0.6513	7	0.51	6	0.4483	9	0.4394	8	NA	0.9941
Q04087	0.8876	1	0.6484	3	0.366	2	0.4236	1	NA	0.7893
P31379	0.1231	0	0.0308	0	0	0	0	0	NA	0.1499
P53944	0.1338	0	0	0	0	0	0.0382	0	NA	0.1216
P53214	0.8748	2	0.2505	1	0.2632	1	0.6243	3	NA	1
P53919	0.813	2	0.7947	2	0.7683	3	0.7642	3	NA	0.9975
Q08208	0.7298	3	0.5054	4	0.4793	3	0.4771	3	NA	0.9596
P32831	0.6905	5	0.5253	4	0.4271	3	0.4107	4	NA	0.982
P40447	0.1357	0	0	0	0	0	0.0251	0	NA	0.1838

P47035	0.8999	1	0.8528	4	0.8251	2	0.8234	3	NA	0.9999
P53224	0.3423	1	0.1667	0	0.2883	1	0.3153	1	NA	0.3212
P36023	0.2364	2	0.0649	1	0.0684	1	0.0753	1	TRU	0.1914
P43575	0.2131	0	0	0	0	0	0.0164	0	NA	0.1078
P38351	0.5685	4	0.3348	1	0.2989	1	0.3124	1	NA	0.5273
P39937	0.1583	0	0.0116	0	0	0	0.027	0	NA	0.1557
P17157	0.259	1	0.0557	0	0	0	0.0787	0	NA	0.2528
P25625	0.0672	0	0	0	0	0	0	0	NA	0.097
Q12236	0.5939	5	0.4801	5	0.3922	3	0.4126	6	NA	0.9312
P32800	0.1218	0	0	0	0	0	0.0849	0	NA	0.1178
Q06169	0.4971	2	0.2964	2	0.2753	1	0.2906	1	NA	0.6017
Q08959	0.0436	0	0	0	0	0	0	0	NA	0.1093
P35182	0.1174	0	0.0214	0	0	0	0.032	0	NA	0.1273
P40164	0.331	3	0.1643	0	0.1294	0	0.1422	0	NA	0.4652
P23639	0.196	0	0.012	0	0	0	0.04	0	NA	0.1659
P30657	0.0752	0	0.015	0	0	0	0.0602	0	NA	0.1547
P53549	0.3638	1	0.1945	1	0.1579	1	0.1556	1	NA	0.3233
Q12461	0.3492	0	0.0475	0	0	0	0.0726	0	NA	0.1869
P13298	0.1991	0	0	0	0	0	0.031	0	NA	0.1355
Q99359	0.4946	3	0.2658	3	0.2164	1	0.2133	1	NA	0.4481
Q03713	0.5346	1	0.0692	0	0	0	0.2075	0	NA	0.2854
P01119	0.4919	1	0.3981	1	0.4207	1	0.4498	1	NA	0.5839
P53972	0.2592	1	0.0367	0	0	0	0.0367	0	NA	0.1757
P38615	0.1892	0	0.0243	0	0	0	0.0622	0	NA	0.1374
P0CX45	0.3543	1	0.2874	1	0	0	0.2244	0	NA	0.4613
P0CX41	0.2409	0	0.0146	0	0	0	0.0949	0	NA	0.1579
P0CX42	0.2409	0	0.0146	0	0	0	0.0949	0	NA	0.1579
P0CX87	1	0	1	0	1	0	1	0	NA	0.0248
P49167	0.4359	0	0.0128	0	0	0	0.1154	0	NA	0.1364
P36534	0.5084	2	0.1582	0	0	0	0.1347	0	NA	0.4469
P32786	0.3893	2	0.1767	1	0.1689	2	0.1711	2	NA	0.4167
P54999	0.1977	0	0.0349	0	0	0	0.2093	0	NA	0.1305
P39526	0.1559	3	0.0079	0	0	0	0.0258	0	NA	0.2146
Q06005	0.189	0	0.0579	0	0	0	0.061	0	NA	0.1351
Q9ZZW7	0.0851	0	0	0	0	0	0	0	NA	0.1102
P18409	0.4178	2	0.0365	0	0	0	0.0223	0	NA	0.1505
P53338	0.2495	1	0.0169	0	0	0	0.0465	0	NA	0.1502
P53885	0.7678	1	0.6339	1	0.5738	1	0.5765	1	NA	0.9697
P20484	0.2995	2	0.0628	0	0	0	0.1618	0	NA	0.1861
Q06580	0.5276	0	0.135	0	0	0	0.1227	0	NA	0.2778
P25336	0.2583	2	0.1012	1	0.1041	1	0.1169	2	NA	0.236
P38236	0.8634	2	0.5273	2	0.3852	1	0.3415	1	NA	0.8718
P28273	0.1882	0	0.042	0	0	0	0.0412	0	NA	0.4758
P32612	0.1833	0	0	0	0	0	0.025	0	NA	0.093
P22434	0.2304	0	0.0325	0	0	0	0.0163	0	NA	0.168
P0CE92	0.1833	0	0	0	0	0	0.025	0	NA	0.0875
P38698	0.2217	1	0	0	0	0	0	0	NA	0.1326
P36069	0.1254	0	0.0102	0	0	0	0.0373	0	NA	0.1369
P53633	0.3182	1	0	0	0	0	0	0	NA	0.1366
P53167	0.1027	0	0	0	0	0	0.0162	0	NA	0.1279
P21304	0.4514	3	0.3542	3	0.3073	2	0.3108	3	NA	0.7763
P14291	0.6143	4	0.4051	3	0.2745	2	0.2914	2	NA	0.3854

P40160	0.4635	2	0.2376	1	0.2518	1	0.28	1	NA	0.4128
P29539	0.4509	3	0.3549	7	0.249	7	0.2427	7	NA	0.5634
Q00453	0.8199	1	0.5498	2	0.3412	1	0.3365	1	TRU	0.9953
P41805	0.2534	0	0.009	0	0	0	0.0633	0	NA	0.1209
Q03942	0.0814	0	0	0	0	0	0.0125	0	NA	0.1077
O13563	0.4231	1	0.1603	0	0.2179	0	0.2179	0	NA	0.3701
P27999	0.6475	1	0.041	0	0	0	0.2377	0	NA	0.1679
P06367	0.5182	1	0.3869	0	0.3358	0	0.3285	0	NA	0.2417
P17890	0.5378	2	0.3904	1	0.3705	1	0.4104	1	NA	0.9845
Q04712	0.4655	2	0.217	2	0.2249	2	0.2327	2	NA	0.4249
P02407	0.4853	1	0.0735	0	0	0	0.1691	0	NA	0.251
P0C0T4	0.5556	1	0.1389	0	0	0	0.3611	1	NA	0.1405
Q06164	0.337	5	0.1657	2	0.1149	1	0.1217	1	NA	0.4393
Q99257	0.4107	3	0.1402	1	0.1035	1	0.1052	1	NA	0.2623
P40469	0.1095	0	0.0078	0	0	0	0.0155	0	NA	0.1748
P16523	0.2185	0	0.037	0	0	0	0.0778	0	NA	0.1785
P52918	0.1511	1	0.0343	0	0.0531	0	0.0564	0	NA	0.1684
P07884	0.2009	0	0.028	0	0	0	0.0467	0	NA	0.1459
P37293	0.4375	3	0.1354	0	0.1215	0	0.1771	0	NA	0.2721
Q99271	0.5645	2	0.5056	2	0.5096	3	0.4954	3	NA	0.9703
Q12408	0.1445	0	0.0173	0	0	0	0.0289	0	NA	0.1636
P25655	0.2391	4	0.0655	2	0.0617	2	0.0674	1	NA	0.3558
Q99207	0.6519	2	0.4926	7	0.3963	4	0.3654	3	NA	0.671
P38122	0.0833	0	0	0	0	0	0.0288	0	NA	0.1117
P38725	0.175	0	0	0	0	0	0.0417	0	NA	0.0933
P05375	0.0922	0	0	0	0	0	0	0	NA	0.0963
Q04383	0.6833	3	0.3033	2	0.238	1	0.2457	1	NA	0.8221
Q05788	0.254	1	0.0643	0	0	0	0.0643	0	NA	0.6865
Q04636	0.404	2	0.2174	1	0.1848	1	0.1957	1	NA	0.2729
Q12161	0.7783	2	0.5788	2	0.5788	2	0.5887	2	NA	0.9799
P06838	0.5429	1	0.4524	2	0.4	1	0.3714	2	NA	0.6852
P38344	0.5852	3	0.3384	1	0.3308	2	0.3206	1	NA	0.734
P53721	0.4509	2	0.0357	0	0	0	0.1071	0	NA	0.3582
Q03792	0.1183	0	0.0096	0	0	0	0.0165	0	NA	0.1832
P0CX44	0.4931	2	0.0369	0	0	0	0.0783	0	NA	0.2885
P38222	0.3986	3	0.096	1	0.1014	1	0.1087	1	NA	0.3396
P0CX43	0.4931	2	0.0369	0	0	0	0.0783	0	NA	0.2885
P05745	0.79	2	0.25	0	0	0	0.26	0	NA	0.1722
P14120	0.2095	0	0	0	0	0	0.1238	0	NA	0.1392
P0CX28	1	1	0.1792	0	0	0	0.3868	0	NA	0.4323
P0CX84	0.8833	2	0.0583	0	0	0	0.2167	0	NA	0.3644
P53236	0.472	3	0.3588	4	0.3147	4	0.292	3	NA	0.9511
P45978	0.7994	1	0.8195	1	0.6905	2	0.6705	3	NA	0.9981
P53047	0.1767	0	0	0	0	0	0.041	0	NA	0.127
Q08490	0.9254	2	0.8	1	0.722	2	0.7034	3	NA	0.9984
P51534	0.2053	2	0.0177	0	0	0	0.0469	0	NA	0.1946
P40486	0.3826	2	0.1223	0	0	0	0.0986	0	NA	0.2126
Q3E785	0.2532	0	0.038	0	0	0	0.1899	0	NA	0.1117
P32855	0.3296	4	0.0939	1	0.0939	1	0.108	1	NA	0.3571
P34234	0.4118	2	0.2353	0	0	0	0.183	0	NA	0.3913
P47017	0.5	1	0.1686	0	0.1919	0	0.2151	0	NA	0.3397
P0CW40	0.3243	2	0.0543	0	0	0	0.0696	0	NA	0.2805

P22133	0.1671	0	0.0212	0	0	0	0.0398	0	NA	0.2147
Q12019	0.2487	5	0.1269	1	0.1248	1	0.1291	1	NA	0.3579
Q08689	0.1591	0	0	0	0	0	0	0	NA	0.1246
Q05777	0.8858	2	0.4685	2	0.4094	2	0.437	2	NA	0.9976
P38996	0.7594	4	0.7506	3	0.6521	3	0.6397	4	NA	0.9987
P42939	0.3267	0	0.1089	0	0	0	0.2178	0	NA	0.1714
Q06512	0.1649	0	0.0036	0	0	0	0.0326	0	NA	0.1264
P14743	0.2593	1	0.0593	0	0	0	0.0703	0	NA	0.1461
P40477	0.6479	4	0.4541	4	0.5021	2	0.4822	5	NA	0.9998
P38837	0.2096	1	0.0859	0	0.0893	0	0.0962	0	NA	0.1779
O94084	0.4316	1	0.0842	0	0	0	0.1754	0	NA	0.9969
Q12375	0.1062	0	0	0	0	0	0	0	NA	0.1307
P35729	0.0858	1	0.0193	0	0	0	0.0347	0	NA	0.1138
Q04734	0.2343	0	0	0	0	0	0.0377	0	NA	0.1318
P0CE88	0.175	0	0	0	0	0	0.025	0	NA	0.0884
P0CE90	0.1417	0	0	0	0	0	0.025	0	NA	0.0929
P25360	0.273	2	0.1083	2	0.1073	1	0.1105	1	NA	0.5747
Q12057	0.6454	1	0.4929	2	0.4574	2	0.4291	2	NA	0.705
P40035	0.1233	0	0.0033	0	0	0	0	0	NA	0.1026
P38969	0.2033	0	0.0236	0	0	0	0.0851	0	NA	0.3084
P05030	0.2407	2	0.1187	1	0.1078	1	0.11	1	NA	0.38
P38148	0.1735	1	0.0434	0	0	0	0.0558	0	NA	0.2389
P21243	0.1548	0	0.0317	0	0	0	0.0516	0	NA	0.1658
Q08931	0.8271	1	0.6692	1	0.7895	1	0.782	1	NA	0.8985
Q08647	0.3462	3	0.1612	3	0.1302	2	0.1287	1	NA	0.2822
P53294	0.1213	0	0.0173	0	0	0	0.0223	0	NA	0.1306
P47116	0.6039	2	0.5477	3	0.533	3	0.5183	3	NA	0.994
Q02772	0.6667	1	0.1111	0	0	0	0.2593	0	NA	0.1932
P47104	0.2093	3	0.1002	1	0.1024	1	0.1186	2	NA	0.159
Q03778	0.0596	0	0	0	0	0	0.0183	0	NA	0.1198
P26754	0.4139	2	0.2344	0	0.2381	1	0.2747	1	NA	0.8728
P38066	0.2696	1	0.1768	1	0.1768	1	0.1826	1	NA	0.3423
P40043	0.5776	1	0.1615	0	0	0	0.1429	0	NA	0.6091
Q12690	0.4623	1	0.2563	0	0	0	0.1859	0	NA	0.1881
P39975	0.1385	0	0	0	0	0	0.039	0	NA	0.1343
Q04847	0.0814	0	0	0	0	0	0.0181	0	NA	0.1055
P40156	0.7056	3	0.3505	0	0	0	0.1729	0	NA	0.4384
Q01855	0.4296	1	0.0775	0	0	0	0.1549	0	NA	0.5691
P34218	0.4152	3	0.2587	2	0.2238	2	0.2431	2	NA	0.6719
Q08985	0.1138	0	0	0	0	0	0.0215	0	NA	0.1175
P19955	0.2195	0	0	0	0	0	0.1057	0	NA	0.2886
Q12108	0.2928	1	0.0864	0	0	0	0.0758	0	NA	0.3326
P39954	0.0757	0	0	0	0	0	0.0334	0	NA	0.1114
P22579	0.5273	5	0.4212	7	0.4069	8	0.3861	7	NA	0.9928
Q08887	0.9224	2	0.6209	4	0.5668	5	0.5758	4	TRU	0.9873
P39683	0.2424	0	0.042	0	0	0	0.0513	0	NA	0.1221
P53204	0.2734	2	0.2152	2	0.2228	2	0.2152	2	NA	0.2547
P32336	0.6063	2	0.4301	4	0.3631	2	0.3631	2	NA	0.9254
P53718	0.9237	1	0.49	2	0	0	0.5462	2	NA	0.9889
Q12454	0.3304	2	0.0134	0	0	0	0.0938	0	NA	0.1637
P47054	0.1491	1	0.0131	0	0	0	0.0208	0	NA	0.2027
Q02199	0.7945	2	0.5403	3	0.4682	2	0.4788	2	NA	0.9997

P16387	0.3024	1	0.1024	0	0	0	0.0833	0	NA	0.2727
P21375	0.2854	0	0.0878	0	0	0	0.0639	0	NA	0.2883
P53259	0.1532	0	0.0029	0	0	0	0.0491	0	NA	0.1707
P38787	0.1266	0	0	0	0	0	0.0422	0	NA	0.127
Q12370	0.1452	0	0	0	0	0	0.0484	0	NA	0.099
Q08322	0.1417	0	0	0	0	0	0.025	0	NA	0.0919
P38921	0.1444	0	0	0	0	0	0.0352	0	NA	0.1553
P39684	0.4108	3	0.1047	0	0.1522	1	0.1669	1	NA	0.3994
Q03407	0.4413	3	0.3316	3	0.3211	3	0.3094	2	NA	0.7422
Q03370	0.4206	2	0.2545	2	0.2473	2	0.2509	2	NA	0.6034
P53184	0.2454	0	0.0463	0	0	0	0.0926	0	NA	0.1642
P53043	0.1754	0	0.037	0	0	0	0.0487	0	NA	0.2417
P36002	0.4849	2	0.3293	3	0.2825	2	0.281	2	NA	0.9523
P15380	0.2201	1	0.1515	1	0.1722	1	0.1691	1	NA	0.3579
Q35811	0.1522	0	0	0	0	0	0	0	NA	0.0248
P10862	0.7762	2	0.4045	1	0.3552	1	0.3634	2	NA	0.9648
Q04231	0.2429	1	0	0	0	0	0.0395	0	NA	0.1203
Q08096	0.0954	0	0	0	0	0	0.0409	0	NA	0.2387
Q04418	0.5604	2	0.3326	2	0.2369	2	0.2551	2	NA	0.5106
P40339	0.0526	0	0.0093	0	0	0	0.0279	0	NA	0.1037
P53893	0.3495	3	0.1405	1	0	0	0.0802	0	NA	0.3848
P05749	0.2479	0	0.1322	0	0	0	0.2149	0	NA	0.1169
P87262	0.5785	0	0.3884	0	0	0	0.2645	0	NA	0.1535
P10664	0.4006	3	0.221	1	0	0	0.2044	0	NA	0.3544
P23250	0.7592	4	0.4717	2	0.4079	2	0.4201	2	NA	0.9648
P46669	0.5184	3	0.408	1	0.3712	2	0.362	2	NA	0.8092
P53046	0.3221	2	0.1896	3	0.187	3	0.1853	3	NA	0.6373
P42847	0.341	1	0.0092	0	0	0	0.1152	0	NA	0.1376
P03882	0.1957	0	0	0	0	0	0	0	NA	0.1195
Q04491	0.1145	0	0.0707	0	0	0	0.0673	0	NA	0.1434
P28791	0.4465	3	0.0496	0	0	0	0.1462	0	NA	0.7253
P39997	0.1156	0	0.0041	0	0	0	0.0345	0	NA	0.1914
P40991	0.4838	2	0.3706	1	0.3689	2	0.3414	1	NA	0.2522
P53081	0.0729	0	0	0	0	0	0	0	NA	0.1117
P40007	0.8831	2	0.5152	2	0.4372	2	0.4632	2	NA	0.7621
P53317	0.8827	2	0.5612	1	0.6429	1	0.6735	1	NA	0.8275
P53253	0.563	4	0.1709	2	0.1912	1	0.2083	2	NA	0.8433
P38830	0.4466	3	0.2568	1	0.2057	2	0.2057	2	TRU	0.3516
P32499	0.8681	2	0.7389	7	0.7903	3	0.7861	5	NA	0.9999
P33895	0.5144	3	0.0377	0	0	0	0.0488	0	NA	0.156
Q02629	0.8415	2	0.7518	6	0.684	6	0.682	6	NA	1
Q08485	0.1856	1	0.0234	0	0	0	0.0686	0	NA	0.1668
P32332	0.1049	0	0.037	0	0	0	0	0	NA	0.1214
P40219	0.5203	1	0.0405	0	0	0	0.3041	1	NA	0.2646
P53949	0.1878	0	0.0152	0	0	0	0.1218	0	NA	0.1831
P54791	0.2533	2	0.0113	0	0	0	0.034	0	NA	0.1451
P33401	0.1509	0	0.0561	0	0	0	0.0474	0	NA	0.3272
P23595	0.244	1	0.2175	1	0.2069	1	0.2414	1	NA	0.2843
P38193	0.6417	1	0.5556	1	0.5624	1	0.517	2	NA	0.9467
P39994	0.1321	0	0.0036	0	0	0	0.0196	0	NA	0.1777
Q9ZZW3	0.14	0	0	0	0	0	0	0	NA	0.1847
P40475	0.1901	1	0.0835	1	0.1101	1	0.1226	1	NA	0.2166

P43122	0.1916	1	0	0	0	0	0.0221	0	NA	0.1318
P25454	0.2725	1	0.1925	1	0.2	1	0.205	1	NA	0.3259
Q08904	0.2839	3	0.1209	1	0	0	0.1044	0	TRU	0.134
Q12196	0.4174	2	0.1591	1	0.219	1	0.2211	1	NA	0.3021
Q00245	0.316	1	0.1169	0	0.1688	0	0.2251	0	NA	0.5485
P43556	0.2101	1	0.0392	0	0.0476	0	0.0504	0	NA	0.1772
Q3E757	0.4138	1	0.1207	0	0	0	0.1494	0	NA	0.1579
P14126	0.2739	1	0.0568	0	0	0	0.0491	0	NA	0.2208
P17076	0.3672	2	0.25	1	0.2227	1	0.2656	1	NA	0.3841
P46969	0.1765	0	0.042	0	0	0	0.1008	0	NA	0.1392
Q08745	0.2571	0	0.1333	0	0	0	0.219	0	NA	0.1825
P20434	0.3395	1	0.0419	0	0	0	0.0605	0	NA	0.1561
P53117	0.2926	1	0.0193	0	0	0	0.09	0	NA	0.2111
P0CX56	0.2671	0	0.0685	0	0	0	0.1027	0	NA	0.125
P40090	0.0946	0	0	0	0	0	0	0	NA	0.1212
P43612	0.4182	4	0.3493	4	0.3503	5	0.3283	5	NA	0.9424
P53305	0.3545	1	0.3	0	0.2727	0	0.3091	0	NA	0.2
P38272	0.9294	2	0.5976	3	0.4282	3	0.4329	2	NA	0.9679
P53075	0.4731	4	0.1941	0	0.1369	1	0.175	1	NA	0.2407
Q03782	0.3374	2	0.2317	2	0.2317	2	0.2297	2	NA	0.3043
P40511	0.2062	2	0	0	0	0	0	0	NA	0.2308
P32583	0.9433	2	0.9458	2	0.8966	1	0.9113	1	NA	0.9997
Q08920	0.6394	2	0.2212	1	0.2837	1	0.2981	1	NA	0.5566
Q03503	0.0511	0	0	0	0	0	0	0	NA	0.1035
Q07623	0.76	2	0.7467	2	0.5911	2	0.5822	2	NA	0.8989
Q01560	0.7923	2	0.6812	2	0.6449	2	0.6473	3	NA	0.9987
P38861	0.2761	1	0.1429	1	0.1274	1	0.1178	0	NA	0.1969
Q08972	0.408	6	0.1572	2	0.1522	3	0.1472	2	NA	0.4709
P0CE68	0.1782	2	0.0501	1	0.0608	1	0.0714	2	NA	0.2894
P53898	0.3645	1	0.0535	0	0	0	0.0502	0	NA	0.1615
Q99297	0.0814	0	0.0033	0	0	0	0	0	NA	0.1587
Q07521	0.5556	1	0	0	0	0	0.213	0	NA	0.5123
Q06833	0.4545	1	0.3468	2	0.3312	2	0.3286	2	NA	0.7529
P38126	0.2766	1	0.0408	0	0	0	0.0621	0	NA	0.1963
P53124	0.5173	2	0.2402	1	0.2009	1	0.2102	1	NA	0.59
P38155	0.1833	0	0	0	0	0	0.025	0	NA	0.0878
P08018	0.6287	1	0.4775	3	0.4431	2	0.4401	3	NA	0.982
P53860	0.3447	1	0.0655	0	0	0	0.1197	0	NA	0.4158
P53252	0.5782	3	0.3746	2	0.3451	2	0.3451	2	NA	0.7028
P53872	0.8627	1	0.7255	1	0.7304	1	0.7794	1	NA	0.9966
Q08215	0.3446	2	0.0627	0	0	0	0.1279	0	NA	0.2845
Q12006	0.2698	1	0.0979	0	0	0	0.0714	0	NA	0.2713
P40454	0.2417	1	0.1603	1	0.1934	1	0.1934	1	NA	0.3446
P29461	0.4493	3	0.176	1	0.1587	1	0.1747	0	NA	0.3551
P31244	0.2759	1	0.2177	1	0.1987	1	0.2051	1	NA	0.2026
Q05672	0.8206	1	0.6127	2	0.5449	2	0.5536	3	NA	0.9957
Q08237	0.6644	2	0.2561	1	0.218	1	0.2284	1	NA	0.4663
Q12305	0.3381	0	0.0432	0	0	0	0.1223	0	NA	0.4066
Q99258	0.2548	1	0.0481	0	0	0	0.0625	0	NA	0.3158
Q12194	0.5094	3	0.0772	0	0	0	0.1253	0	NA	0.6693
P0CX49	0.3495	1	0.3387	0	0	0	0.3011	1	NA	0.3992
P0CX24	0.2209	0	0	0	0	0	0.064	0	NA	0.186

P38061	0.5538	1	0.2923	1	0	0	0.3	0	NA	0.1586
P0CX26	0.2391	0	0	0	0	0	0.1196	0	NA	0.1358
P05319	0.7264	1	0.3302	0	0.434	1	0.4057	1	NA	0.8693
P53881	0.3722	1	0.0939	0	0.1553	1	0.1618	1	NA	0.28
P0CX47	0.3333	1	0.0577	0	0	0	0.141	0	NA	0.1736
P48589	0.2448	0	0	0	0	0	0.0629	0	NA	0.1928
Q06109	0.2238	0	0.0036	0	0	0	0.0505	0	NA	0.1338
P38766	0.3389	1	0.2337	3	0.1812	2	0.2282	2	NA	0.6821
P33335	0.0737	0	0	0	0	0	0	0	NA	0.1148
Q12443	0.5522	1	0.5191	1	0.4809	2	0.4835	2	NA	0.9393
P25294	0.8068	2	0.5312	2	0.4148	1	0.4176	0	NA	0.9952
P52870	0.5366	1	0.3902	1	0.5244	1	0.4756	1	NA	0.7906
P32432	0.8272	4	0.7687	5	0.631	3	0.59	4	TRU	0.9991
P40510	0.2367	1	0.049	0	0	0	0.0597	0	NA	0.2881
P34250	0.9708	3	0.8737	4	0.7306	4	0.7253	6	NA	0.9995
P21801	0.1842	0	0.0038	0	0	0	0.0188	0	NA	0.1227
Q08230	0.4136	1	0	0	0	0	0.0926	0	NA	0.1856
P32844	0.2584	2	0.0099	0	0	0	0.0236	0	NA	0.1289
P38072	0.2625	0	0.0365	0	0	0	0.0731	0	NA	0.2342
Q12420	0.9625	4	0.8313	8	0.5486	4	0.569	4	NA	0.9863
P23615	0.4039	4	0.2136	2	0.1992	1	0.2012	1	NA	0.4959
P32377	0.3409	1	0.0657	0	0	0	0.0783	0	NA	0.2147
Q08213	0.0826	0	0.0028	0	0	0	0.0138	0	NA	0.1126
P50874	0.119	0	0.023	0	0	0	0.048	0	NA	0.1778
A0A023PZI1	1	1	0.2735	0	0.3761	1	0.4188	1	NA	0.948
Q04958	0.4163	8	0.218	4	0.212	3	0.2239	4	NA	0.9851
P40530	0.1345	0	0.0305	0	0	0	0.0533	0	NA	0.2546
P16467	0.1048	0	0.0071	0	0	0	0.0195	0	NA	0.1178
P53896	0.1364	0	0	0	0	0	0	0	NA	0.1218
P33760	0.1097	0	0.0049	0	0	0	0.0204	0	NA	0.2424
P07271	0.5262	3	0.2759	2	0.2398	3	0.2305	2	NA	0.7859
P32452	0.1317	0	0.006	0	0	0	0.024	0	NA	0.1648
P47032	0.5886	1	0.2742	1	0.2207	1	0.2642	1	NA	0.9881
P23394	0.2959	1	0.1803	1	0.1633	1	0.182	1	NA	0.1839
P25451	0.1268	0	0	0	0	0	0	0	NA	0.1053
P54885	0.1557	0	0.0219	0	0	0	0.0285	0	NA	0.1374
P28708	0.444	1	0.278	2	0.2008	1	0.2259	1	NA	0.3972
P54113	0.0897	0	0.0017	0	0	0	0.0102	0	NA	0.1255
Q06891	0.0608	0	0	0	0	0	0.0442	0	NA	0.1105
P38927	0.2286	1	0.0408	0	0	0	0.0653	0	NA	0.1408
P32841	0.6542	2	0.5093	2	0.2897	1	0.3131	1	NA	0.4975
P53879	0.5468	2	0.4532	1	0.435	1	0.4562	2	NA	0.88
P26784	0.2965	1	0	0	0	0	0.0352	0	NA	0.1621
P0CX50	0.3495	1	0.3387	0	0	0	0.3011	1	NA	0.3992
P56628	0.2295	0	0.0656	0	0	0	0.1803	0	NA	0.1084
P0CX85	0.8833	2	0.0583	0	0	0	0.2167	0	NA	0.3644
P36533	0.4286	0	0	0	0	0	0.2429	0	NA	0.1612
P43620	0.5861	2	0.5045	2	0.4637	2	0.4607	2	NA	0.9503
P05739	0.5	1	0.0966	0	0.1136	0	0.142	0	NA	0.1654
P38064	0.3405	1	0.0474	0	0	0	0.125	0	NA	0.1788
P0CX48	0.3333	1	0.0577	0	0	0	0.141	0	NA	0.1736
Q05636	0.1541	0	0.0197	0	0	0	0.0525	0	NA	0.1633

P43607	0.4118	2	0.346	1	0.3253	1	0.3322	1	NA	0.5813
P38701	0.6942	1	0.2397	0	0	0	0.2727	0	NA	0.1941
P36080	0.977	1	0.8387	4	0.8779	3	0.8341	3	NA	0.986
P0CX37	0.5551	1	0.2669	0	0	0	0.1695	0	NA	0.2486
P22470	0.8721	3	0.8541	4	0.7574	4	0.7525	6	NA	0.9995
O94742	0.7528	1	0.7978	0	0	0	0.3596	0	NA	0.2164
P22211	0.6747	3	0.5165	6	0.5152	6	0.5063	5	NA	0.9999
Q03435	0.8522	3	0.4532	1	0.3793	1	0.3695	1	NA	0.8634
P53920	0.1194	1	0.0241	0	0.0512	1	0.0612	1	NA	0.2645
P53935	0.8556	4	0.6226	8	0.5282	5	0.5137	6	NA	0.998
P14907	0.9417	2	0.8117	2	0.7837	1	0.7801	2	NA	1
Q03125	0.6883	1	0.2987	0	0	0	0.2338	0	TRU	0.4909
P32263	0.2657	1	0.014	0	0	0	0	0	NA	0.1364
P38755	0.3501	2	0.0984	0	0.0961	0	0.1167	0	NA	0.2878
P40186	0.4316	1	0.2351	0	0.214	1	0.2877	1	NA	0.6937
P25610	0.1532	0	0	0	0	0	0.0403	0	NA	0.1001
P53179	0.4539	2	0.2841	3	0.2509	2	0.2749	2	NA	0.8136
P32609	0.3961	3	0.0816	0	0	0	0.1146	0	NA	0.2398
P17442	0.2547	3	0.0713	1	0.0671	1	0.0764	1	NA	0.4723
P39104	0.4597	4	0.2842	2	0.2205	2	0.2289	2	NA	0.6221
P53191	0.8346	3	0.6913	3	0.6189	3	0.6047	4	NA	0.9982
P16861	0.1996	1	0.0405	0	0	0	0.0334	0	NA	0.2835
P38848	0.475	2	0.1416	1	0.1744	1	0.1658	1	NA	0.6865
P32945	0.4736	2	0.2732	1	0.2714	2	0.3406	3	NA	0.8385
P40012	0.2059	1	0.0612	0	0	0	0.0594	0	NA	0.2722
P40506	0.1753	0	0.0767	0	0	0	0.1096	0	NA	0.1977
P24384	0.3703	5	0.1729	3	0.1031	1	0.1319	1	NA	0.4421
P50086	0.1447	0	0	0	0	0	0	0	NA	0.1349
Q00723	0.2562	0	0.095	0	0.1405	0	0.157	0	NA	0.2118
Q04705	0.1761	0	0	0	0	0	0.0426	0	NA	0.1348
Q12310	0.485	2	0.1431	0	0	0	0.1345	0	NA	0.4805
P40494	0.6222	2	0.4506	2	0.2975	2	0.3198	2	NA	0.7413
Q12069	0.2403	1	0.1234	1	0	0	0.1126	1	NA	0.262
Q12355	0.5068	2	0.0766	0	0	0	0.1824	1	NA	0.437
P07244	0.091	0	0.015	0	0	0	0.0187	0	NA	0.2094
P07257	0.1359	0	0	0	0	0	0.019	0	NA	0.1252
P28519	0.8032	4	0.5256	1	0	0	0.434	1	NA	0.9103
P38883	0.2805	1	0.1533	1	0.0957	1	0.1127	1	NA	0.2272
P46990	0.4565	1	0.1848	0	0	0	0.2011	0	NA	0.2915
P29453	0.3672	2	0.2266	1	0.1992	1	0.2383	1	NA	0.3148
P36523	0.2688	0	0.0158	0	0	0	0.0672	0	NA	0.2564
Q04740	0.5345	2	0.1351	1	0.1494	1	0.1983	1	NA	0.5616
P38961	0.5434	2	0.2653	1	0.2934	1	0.2934	1	NA	0.3444
Q04487	0.199	1	0	0	0	0	0.0204	0	NA	0.1521
Q12334	0.7578	2	0.6368	2	0.4933	2	0.5112	2	NA	0.7264
P36047	0.4349	2	0.0592	0	0	0	0.0917	0	NA	0.1383
Q04964	1	1	0.7596	0	0.7692	0	0.7212	0	NA	0.9938
P38282	1	1	0.8832	2	0.6117	2	0.6426	2	NA	0.8617
Q08204	0.4684	6	0.0906	0	0	0	0.0476	0	NA	0.2653
Q12516	0.4096	2	0.2265	1	0.1236	1	0.1785	0	NA	0.6737
Q03175	0.2478	1	0.0029	0	0	0	0.0292	0	NA	0.1247
D6VTK4	0.3341	1	0.1578	1	0.1717	1	0.1856	1	NA	0.1579

Q04439	0.4003	5	0.2543	3	0.2305	2	0.224	2	NA	0.5729
P38879	0.569	1	0.3506	1	0.3506	1	0.3736	1	NA	0.2824
Q02795	0.1573	0	0.0035	0	0	0	0.0105	0	NA	0.1743
Q08952	0.5165	2	0.2125	1	0.2161	1	0.2271	1	NA	0.7146
P53170	0.1609	0	0	0	0	0	0.0183	0	NA	0.1334
Q12524	0.2824	1	0	0	0	0	0	0	NA	0.1623
Q12515	0.9119	2	0.8576	4	0.7661	3	0.7356	2	NA	0.8747
P38768	0.4041	1	0.1744	0	0.1773	1	0.1686	1	NA	0.3405
Q99373	0.8656	2	0.419	1	0.3123	1	0.3874	1	NA	0.7676
P38855	0.6466	2	0.2438	0	0.1555	0	0.1555	0	NA	0.3724
P46999	0.5122	1	0.115	0	0	0	0.108	0	NA	0.3057
P40025	0.1776	0	0.0312	0	0	0	0.0623	0	NA	0.1072
P33329	0.593	1	0.5465	1	0.5606	1	0.569	1	NA	0.9966
P24031	0.0771	0	0.0043	0	0	0	0.0064	0	NA	0.1272
P40347	0.236	0	0.0062	0	0	0	0	0	NA	0.1272
P53691	0.3342	1	0.0863	0	0	0	0.1159	0	NA	0.3009
P38796	0.2425	1	0.11	0	0.15	1	0.14	1	NA	0.1632
Q04373	0.3399	1	0.1905	1	0.189	1	0.2027	1	NA	0.2173
Q12318	0.0868	0	0	0	0	0	0.0207	0	NA	0.1092
P00127	0.6735	1	0.8571	1	0.5986	1	0.551	1	NA	0.8747
P25560	0.2553	0	0.0266	0	0	0	0.1702	0	NA	0.2675
P0C2H8	0.1947	0	0	0	0	0	0.1504	0	NA	0.1507
P49626	0.3978	3	0.2099	1	0	0	0.1934	0	NA	0.3575
P41056	0.271	0	0.0187	0	0	0	0.0561	0	NA	0.1371
P36525	0.314	1	0.062	0	0	0	0.0504	0	NA	0.225
Q05635	0.4514	2	0.0971	0	0	0	0.0914	0	NA	0.255
P38886	0.3694	1	0.3246	1	0.2649	1	0.2649	1	NA	0.4944
Q02555	0.3376	3	0.1847	1	0.1614	1	0.1635	1	NA	0.2706
Q04062	0.1883	1	0.0102	0	0	0	0.0509	0	NA	0.1547
P32349	0.4205	5	0.1453	0	0.1453	1	0.1376	0	NA	0.2731
P40042	0.0378	0	0	0	0	0	0.0108	0	NA	0.0957
Q12167	0.3068	1	0.0082	0	0	0	0.0356	0	NA	0.1659
Q07979	0.4004	3	0.2112	1	0.1554	1	0.1633	1	NA	0.349
Q12330	0.2553	0	0.0319	0	0	0	0.1809	0	NA	0.2366
P25303	0.5809	3	0.1592	0	0	0	0.1671	0	NA	0.5006
P32915	0.0708	0	0	0	0	0	0.0167	0	NA	0.1379
Q12529	0.1126	0	0	0	0	0	0.0295	0	NA	0.1042
Q01589	0.9408	1	0.6953	2	0.5473	1	0.574	1	NA	0.9939
P23833	0.2305	0	0.061	0	0	0	0.1017	0	NA	0.1983
P38164	0.2842	4	0.1262	0	0	0	0.1156	0	NA	0.7508
P53304	0.1218	0	0.0128	0	0	0	0.0085	0	NA	0.1111
Q05676	0.2838	0	0.1486	0	0	0	0.2568	0	NA	0.1107
Q06217	0.2818	0	0	0	0	0	0.1182	0	NA	0.1189
P37254	0.1487	0	0.014	0	0	0	0.0203	0	NA	0.1274
P38113	0.0627	0	0.0085	0	0	0	0	0	NA	0.1111
Q12107	0.4755	2	0.1925	0	0	0	0.1434	0	NA	0.3059
P53946	0.4728	3	0.2132	2	0.1815	1	0.1801	1	NA	0.4564
Q06410	0.2374	0	0.0096	0	0	0	0.0312	0	NA	0.1256
Q04489	0.1676	1	0.0419	0	0	0	0.0457	0	NA	0.145
P0CX79	0.1713	0	0.0193	0	0	0	0.0387	0	NA	0.3916
P53104	0.4281	2	0.1918	1	0.1929	1	0.175	1	NA	0.5689
P38348	0.1146	0	0.0021	0	0	0	0.0458	0	NA	0.1242

P09932	0.2201	0	0.0392	0	0	0	0.0461	0	NA	0.1662
P25619	0.244	1	0.1205	1	0.1506	1	0.1566	1	NA	0.2955
P15108	0.3716	4	0.1418	2	0.1362	2	0.1475	2	NA	0.3615
P40441	0.1007	0	0	0	0	0	0.0241	0	NA	0.1191
Q08561	0.7672	2	0.7845	2	1	1	0.9397	2	NA	0.9926
Q12345	0.484	2	0.332	1	0.292	1	0.312	1	NA	0.8644
Q12106	0.2682	1	0.0762	0	0.1159	1	0.1225	1	NA	0.2389
Q12321	0.47	3	0.2968	2	0.2403	1	0.235	1	NA	0.5543
P32570	0.2645	0	0.0744	0	0	0	0	0	NA	0.273
P36032	0.1268	0	0.0233	0	0	0	0.0465	0	NA	0.229
P36060	0.2583	0	0.0397	0	0	0	0.0364	0	NA	0.1798
P50108	0.2519	2	0.0458	0	0	0	0.117	0	NA	0.2957
P46151	0.2572	1	0.0959	0	0.0837	0	0.1081	0	NA	0.4208
P34232	0.3315	1	0.1793	0	0	0	0.2228	0	NA	0.3105
Q06389	0.1684	0	0	0	0	0	0.0579	0	NA	0.1346
Q08229	0.9261	1	0.7984	4	0.6986	4	0.6786	5	NA	0.9955
Q12366	0.1562	0	0.0142	0	0	0	0.0597	0	NA	0.1297
P16603	0.1679	0	0.0116	0	0	0	0.0362	0	NA	0.2768
P36006	0.4017	4	0.2736	2	0.2524	2	0.25	2	NA	0.7013
Q00402	0.6689	15	0.167	5	0.1197	3	0.1179	3	NA	0.906
P33331	0.096	0	0.008	0	0	0	0	0	NA	0.1302
Q08692	0.5108	1	0.1727	0	0	0	0.2302	0	NA	0.6925
P25580	0.1226	0	0.0216	0	0	0	0.0361	0	NA	0.1548
P40550	0.1354	2	0.0305	0	0	0	0.0439	0	NA	0.2468
P50947	0.5333	1	0.4697	1	0.4606	1	0.4606	1	TRU	0.9756
Q12326	0.2739	1	0.1617	0	0	0	0.1518	0	NA	0.3061
Q07930	0.3039	0	0.0539	0	0	0	0.0784	0	NA	0.1589
P19657	0.2439	2	0.1468	1	0.1309	1	0.1352	1	NA	0.4285
P47140	0.1743	0	0.0275	0	0	0	0.0703	0	NA	0.1595
P47036	0.6449	1	0.2029	0	0.4203	1	0.4275	1	NA	0.7197
Q12226	0.2321	0	0.0464	0	0	0	0.0485	0	NA	0.172
Q00582	0.2903	1	0.029	0	0	0	0.1129	0	NA	0.2369
P36113	0.1797	1	0.0254	0	0	0	0.0581	0	NA	0.13
P34244	0.7819	6	0.5711	8	0.4111	8	0.4341	8	NA	0.9973
P53631	0.195	2	0.039	0	0.0904	0	0.1259	0	NA	0.1458
P32899	0.1967	0	0.0273	0	0	0	0.071	0	NA	0.1358
P39567	0.1216	0	0.0124	0	0	0	0.0149	0	NA	0.2012
P39520	0.8664	3	0.835	9	0.7318	4	0.7161	6	TRU	0.9992
P22023	0.1597	1	0.03	0	0.0286	1	0.0344	1	NA	0.1767
Q04066	0.1188	0	0.0383	0	0	0	0.0268	0	NA	0.1089
P43560	0.5386	3	0.3843	3	0.3338	3	0.3412	3	NA	0.7039
P41800	0.5117	3	0.1056	0	0	0	0.1526	0	NA	0.8292
P34165	0.5556	0	0.4444	0	0	0	0.6389	0	NA	0.0248
P32333	0.2833	5	0.1296	2	0.1012	3	0.1071	2	NA	0.5191
P53166	0.3387	3	0.1355	1	0.1141	1	0.1497	1	NA	0.2156
Q02820	0.3774	0	0	0	0	0	0.1698	0	NA	0.281
Q8TGN9	0.319	0	0.0613	0	0	0	0.1411	0	NA	0.1825
Q03735	0.5944	5	0.3977	4	0.3095	3	0.3042	4	NA	0.9644
P20676	0.9944	2	0.8727	12	0.8309	9	0.8067	10	NA	1
Q03790	0.8063	2	0.4842	1	0.4611	2	0.5263	2	NA	0.9801
P40368	0.3128	3	0.0154	0	0	0	0.0421	0	NA	0.136
P38632	0.412	1	0.1199	0	0	0	0.1311	0	NA	0.1997

Q99220	0.2675	1	0.1347	1	0.1107	1	0.1089	1	NA	0.3398
P87286	0.4931	1	0.0069	0	0	0	0.0903	0	NA	0.2046
Q07362	0.9838	1	0.8216	1	0.773	1	0.827	2	NA	0.9962
P33751	0.1322	0	0.0124	0	0	0	0.0579	0	NA	0.131
P46956	0.1833	1	0.0675	0	0.1093	1	0.1222	1	NA	0.1465
Q12746	0.125	0	0	0	0	0	0.0641	0	NA	0.1227
P52960	0.3273	3	0.1195	1	0.1074	1	0.1064	1	TRU	0.2445
P35056	0.4951	1	0.3938	3	0.2925	4	0.2516	1	NA	0.4962
P47190	0.1102	0	0.004	0	0	0	0.0199	0	NA	0.1661
Q06597	0.1077	0	0	0	0	0	0.0615	0	NA	0.1321
Q04749	0.5845	1	0.4601	1	0.385	2	0.4085	2	NA	0.8836
P30902	0.6724	2	0.0517	0	0	0	0.0575	0	NA	0.1455
P40581	0.2883	0	0	0	0	0	0.0368	0	NA	0.1154
P46973	0.3354	0	0.0671	0	0	0	0.1463	0	NA	0.2732
P32465	0.1649	1	0.086	1	0.0947	1	0.1088	1	NA	0.1671
P39935	0.7532	5	0.7153	4	0.6765	3	0.6691	4	NA	0.9983
P13902	0.755	2	0.2119	0	0.3709	1	0.3841	0	TRU	0.5695
P53125	0.462	6	0.2563	1	0.2358	4	0.246	4	NA	0.8495
Q05979	0.1611	1	0.0044	0	0	0	0.0442	0	NA	0.2184
P35191	0.5401	4	0.1644	0	0	0	0.1605	0	NA	0.7907
P47074	0.6155	4	0.3029	2	0.2563	2	0.2699	2	NA	0.5937
O60200	0.3721	0	0.0814	0	0	0	0.2907	0	NA	0.4985
Q02205	0.8696	2	0.7772	2	0.587	1	0.5109	1	NA	0.9424
Q01159	0.2375	1	0.122	1	0.1264	1	0.1329	1	NA	0.3089
P41940	0.0859	0	0	0	0	0	0.0166	0	NA	0.1437
Q06390	0.4869	1	0.2124	1	0	0	0.0588	0	NA	0.181
P38849	0.1179	0	0.0133	0	0	0	0.0494	0	NA	0.2071
P23500	0.125	0	0	0	0	0	0.0263	0	NA	0.1219
Q12181	0.1573	0	0.0257	0	0	0	0.0273	0	NA	0.261
P23059	0.2614	0	0	0	0	0	0.0114	0	NA	0.1749
Q12499	0.499	2	0.2505	1	0.2309	1	0.2348	1	NA	0.2214
P37838	0.6219	5	0.3445	3	0.3212	3	0.3285	3	NA	0.7277
P33755	0.2983	1	0.0431	0	0	0	0.0448	0	NA	0.4729
P47149	0.4279	2	0.1095	0	0	0	0.0896	0	NA	0.1371
P25382	0.1922	1	0.0816	0	0	0	0.0777	0	NA	0.1901
Q12457	0.5825	3	0.2842	2	0.2474	2	0.2579	2	NA	0.753
P38181	0.2397	3	0.0786	2	0.0686	1	0.0759	1	NA	0.6325
Q12202	0.2901	3	0.1077	0	0.1036	0	0.1146	0	NA	0.2557
P36102	0.4713	2	0.2077	1	0	0	0.0957	1	NA	0.4552
P07270	0.8365	2	0.8013	3	0.6795	3	0.6731	3	TRU	0.9956
P38361	0.1551	1	0.0383	0	0	0	0.0679	1	NA	0.1441
Q04493	0.5215	2	0.1227	0	0.1779	0	0.1779	0	NA	0.1261
P53903	0.6899	1	0.3333	0	0.6357	1	0.6202	1	NA	0.984
P38292	0.155	0	0.0194	0	0	0	0.0315	0	NA	0.1351
Q06449	0.6512	1	0.5349	1	0.5116	1	0.5581	1	NA	0.9817
Q03262	0.1527	1	0.045	0	0	0	0.0611	0	NA	0.2235
Q08957	0.6082	4	0.2572	0	0	0	0.2308	0	TRU	0.6183
P0CD90	0.349	1	0.0537	0	0	0	0.2416	1	NA	0.1769
P00856	0.1667	0	0	0	0	0	0	0	NA	0.183
P13517	0.5296	1	0.0627	0	0	0	0.0871	0	NA	0.3385
P43568	0.125	0	0.0353	0	0	0	0.0462	0	NA	0.196
P39708	0.1707	0	0.0066	0	0	0	0.0263	0	NA	0.1765

P38844	0.7385	1	0.2277	0	0.28	1	0.5385	3	NA	0.9998
P38692	0.7269	3	0.612	5	0.4787	5	0.4676	5	NA	0.9985
P08638	0.5282	2	0.2562	2	0.2088	3	0.1896	1	TRU	0.612
P27705	1	1	0.9425	6	0.8492	4	0.7857	6	TRU	0.999
Q02455	0.9392	5	0.4208	5	0.2229	2	0.2389	1	NA	0.7463
Q12198	0.1965	0	0.0122	0	0	0	0.047	0	NA	0.1448
Q99189	0.0947	0	0	0	0	0	0	0	NA	0.1285
P19524	0.3189	4	0.1201	2	0	0	0.0642	1	NA	0.5875
P33420	0.4873	4	0.0219	0	0	0	0.023	0	NA	0.1354
Q03210	0.3683	2	0.2752	2	0.2436	2	0.2535	2	NA	0.84
P38769	0.1571	1	0.0238	0	0	0	0.0476	0	NA	0.1165
P46673	0.1855	0	0.0175	0	0	0	0.0269	0	NA	0.1175
P32770	0.6064	3	0.3978	3	0.2837	3	0.2712	3	NA	0.9201
P53915	0.15	0	0	0	0	0	0.0208	0	NA	0.1314
P20967	0.2535	2	0.0493	0	0	0	0.0385	0	NA	0.2541
P43558	0.2924	1	0.1329	0	0	0	0.1661	0	NA	0.2893
Q06651	0.4965	1	0.2832	1	0.2797	2	0.3112	1	NA	0.7857
P21595	0.0716	0	0.0102	0	0	0	0.0143	0	NA	0.1216
Q01454	0.3722	3	0.1036	0	0	0	0.1046	0	NA	0.3792
P47027	0.4188	2	0.2186	2	0.2094	2	0.2225	2	NA	0.6862
Q03921	0.1643	1	0.0171	0	0	0	0.0294	0	NA	0.27
P36009	0.2503	2	0.0939	1	0.0776	1	0.083	1	NA	0.2817
P07807	0.3886	1	0.0142	0	0	0	0.0237	0	NA	0.1443
Q12125	0.0994	0	0	0	0	0	0	0	NA	0.1627
P40582	0.2137	0	0	0	0	0	0.0427	0	NA	0.1247
Q04005	0.7415	2	0.3844	0	0.3673	1	0.3265	1	NA	0.6695
P41318	0.1881	0	0.1287	0	0	0	0.0396	0	NA	0.1867
P40260	0.1951	1	0.1098	1	0.1138	1	0.122	1	NA	0.1549
Q12205	0.351	4	0.0895	0	0.0554	1	0.0907	1	NA	0.3122
Q12404	0.2893	1	0.0472	0	0	0	0.0629	0	NA	0.2199
P39552	0.2692	1	0.094	0	0	0	0.1667	0	NA	0.1689
P38590	0.6564	2	0.454	3	0.362	2	0.409	2	NA	0.925
P13712	0.263	1	0.0498	0	0	0	0.064	0	NA	0.2497
P32633	0.4388	1	0.1295	0	0.4101	1	0.4245	1	NA	0.8168
P47047	0.3318	4	0.1612	1	0.1333	2	0.1295	2	NA	0.3137
P23503	0.3014	1	0.112	0	0	0	0.0937	0	NA	0.3429
Q00539	0.5468	4	0.1511	0	0	0	0.1434	1	NA	0.5671
P47016	0.2606	0	0.0554	0	0	0	0.0749	0	NA	0.2654
P38757	0.5583	2	0.1794	1	0.2466	2	0.3072	2	NA	0.9437
Q06287	0.2103	1	0.0714	0	0	0	0.0675	0	NA	0.1321
Q99210	0.1681	0	0.0043	0	0	0	0.0474	0	NA	0.1354
P15873	0.1395	0	0	0	0	0	0	0	NA	0.1169
P48565	0.272	2	0.137	1	0.1764	1	0.1839	1	NA	0.3626
P31383	0.126	1	0.0535	1	0.052	1	0.0567	1	NA	0.2254
P52923	0.1376	0	0	0	0	0	0.0265	0	NA	0.1893
P87275	0.3504	1	0.0146	0	0	0	0.1752	0	NA	0.1266
P47182	0.2708	1	0.0069	0	0	0	0.0417	0	NA	0.133
P07246	0.136	0	0.0107	0	0	0	0.024	0	NA	0.1401
P38720	0.1534	0	0.0184	0	0	0	0.0491	0	NA	0.2291
P33204	0.4269	1	0.0643	0	0	0	0.0877	0	NA	0.1556
Q08280	0.1911	1	0.0382	0	0	0	0.0885	0	NA	0.2169
P47040	0.098	0	0	0	0	0	0.0221	0	NA	0.147

Q03758	0.5152	4	0.3163	2	0.2174	1	0.237	1	NA	0.7855
P12630	0.356	2	0.184	1	0.1567	2	0.1567	2	NA	0.7559
P48445	0.1507	0	0.0275	0	0	0	0.029	0	NA	0.2123
P53958	0.8561	1	0.8384	3	0.7096	4	0.6742	4	NA	0.9983
P53082	0.275	0	0.0167	0	0	0	0.0417	0	NA	0.1156
P53687	0.3177	2	0.1969	2	0.2103	2	0.2103	2	NA	0.3022
P38753	0.5752	2	0.4403	1	0.3562	1	0.3496	1	NA	0.8599
P39924	0.1933	2	0.0106	0	0	0	0.1188	0	NA	0.1464
Q12522	0.2408	0	0.0612	0	0	0	0.0612	0	NA	0.1406
Q08416	0.4522	0	0.0127	0	0	0	0.0955	0	NA	0.1294
P47010	0.1077	0	0	0	0	0	0	0	NA	0.1188
P53843	0.5872	1	0.3221	2	0.3725	1	0.3859	1	NA	0.5939
Q02733	0.0762	0	0.01	0	0	0	0	0	NA	0.1153
P53863	0.6627	2	0.4017	2	0.3458	1	0.3576	1	NA	0.7518
P06245	0.2816	1	0.0763	0	0	0	0.1105	0	NA	0.2042
P40309	0.1661	2	0.0309	0	0	0	0.0596	0	NA	0.3093
P00572	0.1898	0	0.0046	0	0	0	0.037	0	NA	0.129
P22517	0.2685	1	0.1186	0	0.094	0	0.1029	0	NA	0.2391
P07264	0.2169	1	0.1361	1	0.1181	1	0.1284	1	NA	0.3041
P39946	0.1397	0	0.0182	0	0	0	0.0344	0	NA	0.2022
Q12502	0.6614	5	0.5244	4	0.3985	2	0.434	2	NA	0.9987
Q10740	0.1461	0	0.0253	0	0	0	0.0328	0	NA	0.1448
P40089	0.2581	0	0.0753	0	0	0	0.2258	0	NA	0.3131
Q06436	0.5164	4	0.3358	3	0.303	2	0.3075	2	NA	0.8617
Q99316	0.4079	2	0.0217	0	0	0	0.0542	0	NA	0.1179
P38191	0.3696	1	0	0	0	0	0	0	NA	0.1495
Q05648	0.3792	2	0.1063	0	0	0	0.1087	0	NA	0.1914
O75012	0.4737	0	0.0842	0	0	0	0.1684	0	NA	0.1256
Q12093	0.1127	0	0.0528	0	0	0	0.0576	0	NA	0.1846
P11633	0.6667	2	0.798	1	0.6768	2	0.6667	2	NA	0.993
Q02892	0.4992	2	0.2442	2	0.1901	2	0.1793	1	NA	0.3988
Q06178	0.3741	1	0.2943	2	0.3217	2	0.2968	2	NA	0.2087
P36129	0.3803	1	0	0	0	0	0	0	NA	0.1338
P16547	0.9466	2	0.4885	1	0.4148	1	0.3766	2	NA	0.8779
Q06668	0.4904	3	0.0552	0	0.0573	0	0.0552	0	NA	0.216
P36163	0.2	0	0	0	0	0	0.0174	0	NA	0.1616
P50076	0.0667	0	0.0095	0	0	0	0	0	NA	0.1254
P39945	0.1767	1	0.0326	0	0	0	0.0442	0	NA	0.1414
P39988	0.1186	0	0	0	0	0	0	0	NA	0.1014
Q12180	0.4466	3	0.1942	3	0.1631	2	0.1515	0	TRU	0.6756
P22943	1	1	0.9817	1	0.9725	1	0.8716	2	NA	0.9795
P31539	0.3579	3	0.1608	1	0.1035	1	0.1167	1	NA	0.3691
P22202	0.4315	2	0.2352	1	0.1308	1	0.1137	1	NA	0.4066
P40885	0.1993	1	0.0705	0	0.0794	1	0.1041	1	NA	0.1688
P12683	0.1888	2	0.0417	0	0.0522	0	0.0569	0	NA	0.2386
P19211	0.5414	1	0.242	0	0	0	0.2102	0	NA	0.5622
P47031	0.3174	2	0.0834	0	0	0	0.1026	0	NA	0.5208
P40051	0.2035	1	0.002	0	0	0	0.0274	0	NA	0.1978
P41734	0.0924	0	0	0	0	0	0	0	NA	0.1055
P26364	0.4711	2	0.0222	0	0	0	0.0756	0	NA	0.166
P47119	0.1371	0	0	0	0	0	0.0355	0	NA	0.1242
P36224	0.7265	3	0.1957	1	0.193	1	0.1957	1	NA	0.3294

P36146	0.3426	2	0.0837	0	0.1016	0	0.1116	0	NA	0.3505
Q12246	0.383	4	0.1859	1	0.1603	1	0.1907	1	NA	0.5115
P53145	0.5047	5	0.2562	2	0.1656	1	0.1938	1	NA	0.7196
P53114	0.2959	3	0.0972	1	0.091	1	0.121	1	NA	0.4109
Q05930	0.092	0	0.01	0	0	0	0.0334	0	NA	0.1445
Q12176	0.5454	5	0.4332	3	0.3883	2	0.399	3	NA	0.9099
Q08179	0.5236	3	0.2845	2	0.2845	3	0.2967	3	NA	0.5793
P38300	0.3094	1	0.0576	0	0	0	0.1043	0	NA	0.1731
P19263	0.2643	3	0.1118	1	0.1063	1	0.11	1	NA	0.4538
P53379	0.3775	3	0.0705	0	0	0	0.0973	1	NA	0.7034
Q12467	0.3907	2	0.1093	0	0	0	0.1349	0	NA	0.3561
P50873	0.2934	2	0.1517	1	0	0	0.0838	0	NA	0.1748
Q06815	0.5171	2	0.189	0	0.2546	0	0.273	0	NA	0.8069
P40314	0.5839	1	0.4564	0	0	0	0.2349	0	NA	0.1247
Q08444	0.5054	2	0.4248	2	0.342	1	0.3355	1	NA	0.5625
Q08287	0.5517	2	0.4194	1	0.3347	1	0.3161	1	NA	0.4179
P27476	0.7512	3	0.7826	3	0.7271	3	0.7246	3	NA	0.9997
P38082	0.7	1	0.3591	0	0	0	0.2045	0	TRU	0.3168
P38271	0.4299	2	0.1707	0	0.2744	1	0.2744	1	NA	0.5454
P38713	0.5412	7	0.2781	3	0.253	3	0.2871	3	NA	0.9806
P16451	0.4976	2	0.1171	0	0	0	0.0634	0	NA	0.3594
Q01802	0.153	0	0.0067	0	0	0	0.0222	0	NA	0.1346
Q9ZZX0	0.1299	0	0	0	0	0	0	0	NA	0.1218
Q04728	0.0998	0	0	0	0	0	0.0227	0	NA	0.138
P32317	0.1984	1	0.0196	0	0	0	0.053	0	NA	0.1374
Q08271	0.2739	2	0.0679	0	0	0	0.0977	0	NA	0.2702
Q99369	0.2707	1	0.0752	0	0	0	0.109	0	NA	0.1347
Q03002	0.674	2	0.4555	6	0.4069	4	0.3977	4	NA	0.9958
Q12263	0.7137	2	0.3897	4	0.331	4	0.3345	3	NA	0.982
P25569	0.2604	2	0.0255	0	0	0	0.0658	0	NA	0.2937
P47011	0.25	1	0.1026	0	0.1263	1	0.1263	1	NA	0.1831
P38736	0.6368	1	0.13	0	0	0	0.1211	0	NA	0.1534
P53258	0.2768	2	0.0895	0	0.0821	1	0.1042	1	NA	0.1916
Q04322	0.5792	3	0.3278	1	0.2958	1	0.2944	2	NA	0.5497
P46943	0.1395	1	0.014	0	0	0	0.0171	0	NA	0.1153
P00815	0.2028	1	0.0163	0	0	0	0.0375	0	NA	0.1409
P53187	0.5	1	0.1495	0	0	0	0.1075	0	NA	0.1434
P38250	0.4419	2	0.3457	2	0.3541	3	0.3277	3	NA	0.8441
Q04213	0.5621	3	0.3895	2	0.4	2	0.4126	2	NA	0.9047
P53877	0.2505	1	0.0829	0	0	0	0.0541	0	NA	0.1912
P38853	0.7216	3	0.4493	3	0.3668	4	0.3488	3	NA	0.9111
Q12166	0.1507	0	0.0132	0	0	0	0.0315	0	NA	0.2445
P24720	0.2157	1	0.0256	0	0	0	0.0407	0	NA	0.1523
Q08970	0.3368	2	0.2025	2	0.2004	2	0.1983	2	NA	0.3286
Q04922	0.5828	2	0.357	0	0	0	0.2882	0	NA	0.7937
P32435	0.2083	0	0.0167	0	0	0	0	0	NA	0.1244
P47085	0.2515	0	0.0178	0	0	0	0.0592	0	NA	0.2312
P0CD99	0.3218	3	0.0591	0	0.1084	0	0.1051	0	NA	0.1488
P0CX81	1	1	0	0	0	0	0.5082	0	NA	0.4827
P27929	0.4259	2	0.0679	0	0	0	0.0329	0	NA	0.2133
Q12080	0.9077	4	0.556	1	0.4637	1	0.4879	1	NA	0.9268
Q08962	0.1768	0	0.0055	0	0	0	0.0884	0	NA	0.3233

P06100	0.3822	0	0	0	0	0	0.0314	0	NA	0.3355
P40010	0.5558	3	0.2865	2	0.1962	1	0.1904	1	NA	0.5179
Q02630	0.8652	2	0.7664	8	0.6765	5	0.6739	5	NA	1
Q05166	0.6989	3	0.6364	2	0.4811	2	0.517	1	NA	0.9674
P52593	0.1656	1	0.0121	0	0	0	0.0218	0	NA	0.1277
P09457	0.3774	1	0.0094	0	0	0	0.0755	0	NA	0.1225
Q08622	0.3076	2	0.0306	0	0	0	0.0683	0	NA	0.1928
P38715	0.1407	0	0	0	0	0	0.0428	0	NA	0.2781
Q05584	0.2409	1	0.062	0	0	0	0.0657	0	NA	0.1328
P48813	0.3047	1	0.2051	2	0.1976	2	0.1659	2	NA	0.5484
P04912	0.3636	0	0.2121	0	0	0	0.2727	0	NA	0.7389
P25364	0.6259	4	0.4592	3	0.3067	3	0.3387	3	TRU	0.8651
P53686	0.2577	1	0.1485	1	0.1597	1	0.1541	1	NA	0.3402
Q02959	0.396	1	0.2626	2	0.2224	2	0.2339	2	NA	0.4619
P39936	0.7265	4	0.6652	4	0.5635	4	0.5656	5	NA	0.9864
P07342	0.2198	1	0.0859	0	0.0713	0	0.0786	0	NA	0.1643
P53892	0.7151	3	0.4473	1	0.2906	1	0.2764	1	NA	0.6026
P38710	0.2034	0	0	0	0	0	0.0271	0	NA	0.1453
P46997	0.6449	3	0.4374	3	0.3671	2	0.3499	2	NA	0.8679
P47135	0.6994	3	0.5005	6	0.4079	6	0.374	3	NA	0.9992
P00724	0.0921	0	0.0056	0	0	0	0.0056	0	NA	0.2743
P35688	0.2773	1	0.0718	1	0	0	0.0826	1	NA	0.2468
P40513	0.3947	0	0.1278	0	0	0	0.0639	0	NA	0.1786
P36046	0.8437	3	0.7916	2	0.737	2	0.7345	2	NA	0.996
Q02889	0.4159	0	0.177	0	0	0	0.2566	0	NA	0.2865
P53152	0.4745	0	0.1971	0	0	0	0.2263	0	NA	0.2595
P23748	0.6841	2	0.435	2	0.4458	2	0.4838	3	NA	0.9859
P25270	0.2913	1	0.0947	0	0	0	0.1019	0	NA	0.1807
P32505	0.699	3	0.4876	2	0.3886	2	0.3752	3	NA	0.8144
Q03264	0.4	2	0.266	2	0.2194	2	0.233	2	NA	0.5631
P36052	0.2736	2	0.0299	0	0	0	0.0995	0	NA	0.2621
P48837	0.7172	2	0.5823	1	0.536	1	0.5416	1	NA	0.9999
Q08214	0.1947	1	0.05	0	0	0	0.0474	0	NA	0.1271
Q2V2Q1	1	1	0.8938	2	0.7788	1	0.8407	1	TRU	0.5774
P53918	0.3611	3	0.1649	0	0	0	0.1308	0	NA	0.708
P15646	0.4098	1	0.3394	1	0.2905	1	0.3058	1	NA	0.5136
Q12119	0.1019	1	0.0038	0	0	0	0.0094	0	NA	0.1757
Q04739	0.5995	2	0.6475	1	0.5372	1	0.5156	2	NA	0.9329
P28007	0.5951	2	0.4878	1	0.5707	2	0.5707	2	NA	0.9965
P40099	0.2796	1	0.0474	0	0	0	0.0616	0	NA	0.2288
P11710	0.6621	4	0.291	2	0	0	0.2246	1	NA	0.6852
P14742	0.1688	1	0.0391	0	0	0	0.0432	0	NA	0.1395
P03069	0.9893	2	0.4342	1	0.2811	1	0.3238	1	TRU	0.4903
Q00055	0.1407	1	0.0051	0	0	0	0.0742	0	NA	0.1905
P40060	0.504	1	0.28	0	0	0	0.168	0	NA	0.1948
Q03694	0.6262	4	0.3571	1	0.3286	2	0.3667	2	NA	0.9399
P30606	0.2299	2	0.1166	1	0.1494	1	0.1544	1	NA	0.316
P11927	0.8938	1	0.6559	4	0.4642	3	0.4411	3	NA	0.8933
P39005	0.4203	2	0.1159	0	0	0	0.1594	0	NA	0.2693
Q08448	0.0933	0	0	0	0	0	0.02	0	NA	0.1139
Q92328	0.2583	1	0.1476	1	0.1845	1	0.203	1	NA	0.212
Q07376	0.1379	0	0.0103	0	0	0	0.0514	0	NA	0.2452

P39106	0.2152	0	0.0472	0	0	0	0.0486	0	NA	0.2347
P32179	0.2521	0	0.056	0	0	0	0.0756	0	NA	0.2147
P39692	0.1623	1	0.0039	0	0	0	0.0155	0	NA	0.1403
P0CE00	0.3272	4	0.0598	0	0.1262	0	0.1246	0	NA	0.1817
Q03162	0.4	3	0.1435	1	0.1226	1	0.1419	1	NA	0.3301
P32340	0.2047	1	0.0292	0	0	0	0.0351	0	NA	0.2421
Q04751	0.2105	0	0.0246	0	0	0	0.0737	0	NA	0.235
P25566	0.1369	0	0.0179	0	0	0	0.0655	0	NA	0.1636
P38798	0.4555	3	0.2011	1	0.1919	1	0.191	1	NA	0.3158
P53939	0.8354	3	0.5725	2	0.4152	2	0.4373	2	NA	0.9548
Q06148	0.4327	1	0.2018	1	0.2427	1	0.2602	1	NA	0.1904
P38747	0.6906	1	0.456	2	0.4951	1	0.4919	1	NA	0.6875
P40039	0.1326	1	0.0341	0	0	0	0.0436	0	NA	0.1114
P13045	0.075	0	0.0154	0	0	0	0.0365	0	NA	0.1554
Q08886	0.3634	4	0.146	2	0.1572	2	0.175	2	NA	0.5446
Q06201	0.4734	2	0.25	0	0.234	0	0.2713	1	NA	0.6044
P02294	0.5954	1	0.3206	1	0.4122	1	0.4351	1	NA	0.9219
P28817	0.228	0	0.016	0	0	0	0.022	0	NA	0.1498
P17709	0.252	1	0.032	0	0	0	0.046	0	NA	0.3033
P40985	0.2141	2	0.0751	0	0	0	0.1244	1	NA	0.3913
P38074	0.2443	1	0.0747	0	0	0	0.1149	0	NA	0.1508
P21190	0.8306	2	0.4	2	0.2944	1	0.2778	1	TRU	0.784
P38697	0.1893	1	0.0249	0	0	0	0.0421	0	NA	0.217
P40565	0.3514	0	0.0068	0	0	0	0.1081	0	NA	0.1599
Q06098	0.3228	1	0.0406	0	0	0	0.0564	0	NA	0.2999
Q6Q560	0.2447	0	0.0426	0	0	0	0.1383	0	NA	0.1048
P52489	0.1304	0	0.0534	0	0	0	0.0395	0	NA	0.1396
Q04081	0.125	0	0	0	0	0	0.0152	0	NA	0.1499
P53150	0.6746	1	0.342	2	0.2874	1	0.3135	2	NA	0.3888
P40970	0.1907	1	0.0089	0	0	0	0.0766	0	NA	0.1844
P57743	0.3146	0	0.0449	0	0	0	0.1685	0	NA	0.1051
P36051	0.0783	0	0.0065	0	0	0	0.0174	0	NA	0.2552
P53091	0.4071	4	0.2576	4	0.2311	3	0.2419	3	NA	0.865
P43594	1	1	0.1235	0	0	0	0.2588	0	NA	0.565
P34166	0.5789	0	0.4737	0	0	0	0.6579	0	NA	0.0248
P40990	0.2365	1	0	0	0	0	0.0399	0	NA	0.1263
P36157	0.9972	1	0.6777	1	0.3691	0	0.3967	0	NA	0.9001
P25846	0.245	3	0.0271	0	0	0	0.0448	0	NA	0.1445
Q12317	0.3598	2	0.061	0	0	0	0.0935	0	NA	0.2135
Q04149	0.3861	3	0.1772	1	0.0965	1	0.1282	0	NA	0.1711
P40096	0.5352	1	0.5423	1	0.4577	1	0.4648	1	NA	0.7127
P36118	0.9037	3	0.4689	1	0.2671	1	0.3075	1	NA	0.7298
Q12032	0.6703	2	0	0	0	0	0.027	0	NA	0.1168
P43636	0.0557	0	0	0	0	0	0	0	NA	0.1041
P00812	0.2222	1	0.015	0	0	0	0.039	0	NA	0.2574
Q06485	0.3553	1	0.1421	0	0.1675	1	0.1777	1	NA	0.3378
P40471	0.1818	0	0.0471	0	0	0	0.064	0	NA	0.1137
P11353	0.2652	0	0.1585	0	0	0	0.0945	0	NA	0.2556
P28789	0.1407	0	0	0	0	0	0.0398	0	NA	0.13
P54862	0.1799	1	0.0617	0	0.0794	1	0.0988	1	NA	0.2021
P23585	0.1719	1	0.0092	0	0	0	0.0795	1	NA	0.2204
P39734	0.7936	3	0.5246	3	0.4905	3	0.5095	5	NA	0.9683

P28241	0.2222	0	0.0488	0	0	0	0.065	0	NA	0.2909
P38217	0.1373	1	0.037	0	0.049	1	0.0545	1	NA	0.1788
P25605	0.3819	2	0.165	0	0	0	0.1715	0	NA	0.2337
P06168	0.1797	1	0.0608	0	0	0	0.0506	0	NA	0.1488
P32617	0.5964	1	0.3916	1	0.2771	1	0.3072	1	NA	0.2713
Q08045	0.5649	1	0.0382	0	0	0	0.1298	0	NA	0.2981
P13134	0.4189	3	0.1646	1	0.1572	1	0.156	1	NA	0.7221
P0CX09	0.1076	0	0.0319	0	0	0	0.0259	0	NA	0.1892
P38888	0.1131	0	0.0188	0	0	0	0.0201	0	NA	0.145
P01149	0.3394	0	0.0424	0	0	0	0	0	NA	0.2405
P18408	0.2452	0	0.0651	0	0	0	0.0805	0	NA	0.1302
P53059	0.0932	0	0	0	0	0	0.0251	0	NA	0.1148
P53176	0.2778	1	0.0641	0	0	0	0.1496	0	NA	0.2015
P32335	0.211	1	0.0734	0	0.0711	0	0.0826	0	NA	0.2026
Q05473	0.3535	1	0.0318	0	0	0	0.0446	0	NA	0.199
Q12175	0.1598	1	0.0111	0	0	0	0.0377	0	NA	0.2002
P53070	0.0972	0	0.012	0	0	0	0.0224	0	NA	0.1389
P25374	0.2072	1	0.0905	0	0	0	0.0744	0	NA	0.4979
P47096	0.0904	0	0.0169	0	0	0	0.0621	0	NA	0.1427
P42837	0.2469	1	0.0432	0	0	0	0.0603	0	NA	0.2494
P38631	0.201	4	0.0965	1	0.088	1	0.1002	1	NA	0.2826
P08417	0.1721	0	0.0697	0	0	0	0.0758	0	NA	0.1527
P08524	0.1875	0	0	0	0	0	0.0284	0	NA	0.1355
P32791	0.1603	1	0.0131	0	0	0	0.0306	0	NA	0.2085
Q06625	0.1888	0	0.0221	0	0	0	0.0202	0	NA	0.3387
P25346	0.2143	2	0.0463	0	0	0	0.0579	0	NA	0.1116
P25370	0.258	2	0.0883	1	0.0989	1	0.1148	1	NA	0.2477
P18852	0.6455	1	0.2727	0	0	0	0.2727	0	NA	0.1925
P36148	0.2813	1	0.1023	1	0.1332	1	0.1655	1	NA	0.3269
P53849	0.6601	1	0	0	0	0	0.0588	0	NA	0.1556
Q01766	0.7381	1	0.4354	1	0.2857	1	0.2891	1	NA	0.7493
Q07729	0.1616	1	0.0511	0	0	0	0.0532	0	NA	0.1371
Q06214	0.4736	3	0.1687	1	0.2093	1	0.2297	2	NA	0.3489
P38144	0.4446	7	0.1816	1	0.1479	2	0.1692	2	NA	0.6331
P27810	0.1399	1	0	0	0	0	0.056	0	NA	0.1798
P15454	0.4064	0	0.0642	0	0	0	0.1658	0	NA	0.1709
P20485	0.3625	3	0.1495	1	0.1186	1	0.1564	1	NA	0.5064
P53153	0.1569	0	0.0255	0	0	0	0.0693	0	NA	0.1655
P49367	0.2237	1	0.0361	0	0	0	0.0317	0	NA	0.2895
Q12405	0.2196	0	0.0698	0	0	0	0.1008	0	NA	0.2359
Q08218	0.2219	1	0.0449	0	0.1489	1	0.1404	1	NA	0.2149
P38878	0.4209	3	0.0827	0	0.1043	0	0.1187	0	NA	0.3089
Q06567	0.1476	0	0.007	0	0	0	0.0334	0	NA	0.1314
P47025	0.493	4	0.1695	0	0.1218	1	0.1387	1	NA	0.3861
P32787	0.368	1	0.1078	0	0	0	0.1822	0	NA	0.4175
P38257	0.5818	3	0.2836	1	0.1751	1	0.1968	1	NA	0.3332
P36027	0.7979	2	0.25	0	0.2899	2	0.6011	2	NA	0.9999
P36066	0.3218	1	0.0022	0	0	0	0.0216	0	NA	0.1304
P43623	0.0412	0	0.0029	0	0	0	0.0294	0	NA	0.1506
P11914	0.251	1	0.056	0	0	0	0.0519	0	NA	0.1952
Q01926	0.2872	2	0	0	0	0	0.0234	0	NA	0.134
P14908	0.0938	0	0	0	0	0	0.0264	0	NA	0.132

P10849	0.3682	1	0.1136	0	0	0	0.05	0	NA	0.1876
P47018	0.7029	3	0.5356	3	0.4937	2	0.5042	2	NA	0.981
P39923	0.4228	3	0.1545	1	0.2016	3	0.2358	3	NA	0.7158
P48234	0.454	1	0.3197	1	0.2942	1	0.3055	1	NA	0.3183
P12904	0.0745	0	0.0155	0	0	0	0.0404	0	NA	0.121
P32794	0.1808	1	0.0731	0	0.0577	0	0.0769	0	NA	0.3991
P0CX78	0.1713	0	0.0193	0	0	0	0.0387	0	NA	0.3916
P35183	0.2145	1	0.0047	0	0	0	0.0233	0	NA	0.1519
P38724	0.1258	1	0.0661	1	0.0694	1	0.0774	1	NA	0.1138
P25300	0.095	0	0.0016	0	0	0	0.0187	0	NA	0.1078
P47136	0.7319	5	0.6068	8	0.3898	5	0.3849	5	NA	0.9792
P40969	0.2171	2	0.0181	0	0	0	0.051	0	NA	0.1302
Q06632	0.2093	3	0.0766	1	0.0634	1	0.0641	0	NA	0.3664
O13297	0.592	2	0.4481	1	0.4117	1	0.4044	1	NA	0.7947
P53298	0.7414	3	0.4729	3	0.3547	3	0.3448	2	NA	0.8463
Q07533	0.4373	2	0.226	1	0.2328	1	0.2407	1	NA	0.8721
P53008	0.1236	0	0.0252	0	0	0	0.018	0	NA	0.1625
P38226	0.1688	0	0.0101	0	0	0	0.0504	0	NA	0.1791
P24309	0.2025	1	0.0963	0	0.0988	1	0.1111	1	NA	0.2575
Q04089	0.4038	3	0.3522	2	0.3402	2	0.3488	2	NA	0.8399
Q12051	0.191	0	0.003	0	0	0	0.0358	0	NA	0.1825
P43577	0.1132	0	0	0	0	0	0	0	NA	0.1183
P38068	0.4187	1	0.266	0	0	0	0.2167	0	NA	0.2391
Q08484	0.5181	3	0.3925	3	0.3956	1	0.4176	3	NA	0.9835
P38873	0.3648	5	0.115	1	0.1008	1	0.106	1	NA	0.539
P07277	0.2144	0	0	0	0	0	0.0293	0	NA	0.1409
P00549	0.148	0	0.056	0	0	0	0.07	0	NA	0.1784
P23292	0.5513	2	0.4524	2	0.4579	2	0.4689	2	NA	0.9829
P06208	0.2633	1	0.0372	0	0	0	0.0743	0	NA	0.2868
P32047	0.9845	3	0.7168	5	0.6504	5	0.6372	5	NA	0.9991
P53102	0.4247	1	0.0091	0	0	0	0.0685	0	NA	0.1942
P15807	0.0985	0	0.0036	0	0	0	0.0365	0	NA	0.115
P46985	0.1564	0	0.0166	0	0	0	0.0782	0	NA	0.3691
P33749	1	1	0.6952	5	0.4873	3	0.5063	3	TRU	0.9977
P54785	0.9286	3	0.7837	3	0.6816	5	0.649	5	TRU	0.9986
Q07349	0.2833	1	0.0024	0	0	0	0.0238	0	NA	0.1402
P33201	0.2627	0	0.0805	0	0	0	0.0975	0	NA	0.1462
P35198	0.5751	2	0.388	1	0.3788	1	0.388	1	NA	0.9681
P53128	0.1383	0	0.0233	0	0	0	0.0317	0	NA	0.1805
Q07500	0.1872	0	0.011	0	0	0	0.0514	0	NA	0.1277
P34160	0.1185	1	0.0116	0	0	0	0.0383	0	NA	0.1713
P40451	0.6225	2	0.2353	0	0	0	0.2304	0	NA	0.7397
P50082	0.2951	1	0.1383	0	0.1265	0	0.1501	1	NA	0.3539
P07347	0.3193	1	0.1723	0	0	0	0.1303	0	NA	0.1602
P06174	0.2036	1	0.0436	0	0	0	0.0836	0	NA	0.1182
P39979	0.1491	0	0.0248	0	0	0	0.0807	0	NA	0.1083
Q05549	0.2358	1	0.0279	0	0	0	0.0325	0	NA	0.1414
P38274	0.2745	3	0.0846	0	0	0	0.0641	0	NA	0.1932
P36078	0.1897	0	0	0	0	0	0.0776	0	NA	0.1095
P47042	0.4648	4	0.099	0	0	0	0.1244	0	NA	0.6677
P32467	0.1997	1	0.0972	1	0.1059	1	0.1198	1	NA	0.1718
P40884	0.2151	0	0.0138	0	0	0	0.0448	0	NA	0.2218

Q06505	0.6707	2	0.5683	3	0.5512	2	0.5537	2	NA	0.9363
Q08773	0.442	6	0.2241	3	0.1929	2	0.2036	2	NA	0.6485
Q07551	0.1635	0	0	0	0	0	0.0321	0	NA	0.1996
P40015	0.1488	0	0.0545	0	0	0	0.0922	0	NA	0.1601
P34253	0.3195	2	0.0383	0	0	0	0.0831	0	NA	0.3294
P30624	0.1671	1	0.0486	0	0	0	0.0443	0	NA	0.1435
Q12494	0.6771	6	0.5086	9	0.4143	5	0.3933	6	NA	0.9884
Q08001	0.5007	3	0.2006	1	0	0	0.1804	0	NA	0.7575
P47051	0.176	1	0.0098	0	0	0	0.0318	0	NA	0.1656
P42834	0.3288	1	0.1301	0	0	0	0.1164	0	NA	0.2656
P53206	0.112	0	0.0051	0	0	0	0.0229	0	NA	0.1655
Q12285	0.7311	2	0.2689	1	0.2453	1	0.2547	1	NA	0.6536
P41910	0.5924	2	0.4127	1	0.438	1	0.4456	1	NA	0.9137
Q99278	0.487	1	0.0087	0	0	0	0.0957	0	NA	0.1303
P29496	0.3226	3	0.0903	0	0	0	0.1239	0	NA	0.6851
P23060	0.1377	0	0.011	0	0	0	0.0358	0	NA	0.1223
Q12411	0.7537	3	0.3432	2	0.3268	2	0.3284	3	NA	0.9666
P23641	0.1576	0	0.0032	0	0	0	0	0	NA	0.1623
Q08550	0.8319	4	0.2586	1	0.1293	1	0.1487	1	NA	0.2374
P53123	0.2094	0	0.0125	0	0	0	0.0406	0	NA	0.1278
P0CY08	0.5905	1	0.0762	0	0	0	0.0667	0	NA	0.2307
P48564	0.4561	2	0.2233	2	0.2118	1	0.1889	1	NA	0.7152
P0CY06	0.4229	2	0.0114	0	0	0	0.04	0	NA	0.111
P53320	0.2131	0	0.0219	0	0	0	0.0628	0	NA	0.1469
P48240	0.328	1	0.228	1	0.244	1	0.256	1	NA	0.5677
P32347	0.0773	0	0.011	0	0	0	0.0414	0	NA	0.1098
P33750	0.4499	3	0.2536	1	0.2086	1	0.2229	1	NA	0.361
P36008	0.2184	1	0.0898	1	0.1262	1	0.1286	1	NA	0.2224
P47144	0.3021	1	0.0993	0	0	0	0.1145	0	NA	0.291
P38728	0.2765	0	0.1118	0	0	0	0.1824	0	NA	0.3169
P36049	0.8899	2	0.8501	4	0.6698	2	0.6674	2	NA	0.9884
P46671	0.6422	2	0.1275	0	0	0	0.1422	0	NA	0.4085
P41277	0.192	0	0.02	0	0	0	0.036	0	NA	0.1463
P53306	0.1264	0	0	0	0	0	0.0099	0	NA	0.1073
P36125	0.1941	1	0.0806	0	0.1319	1	0.1319	1	NA	0.1824
P38625	0.1029	0	0.0019	0	0	0	0.0286	0	NA	0.1318
P06244	0.2494	1	0.2191	1	0.2343	1	0.2242	1	NA	0.4195
Q03361	0.7374	5	0.5388	5	0.5514	4	0.5651	6	NA	0.9991
Q12265	0.379	2	0.1895	0	0	0	0.1895	0	NA	0.8186
P14681	0.2228	1	0.0136	0	0	0	0	0	NA	0.1682
P38689	0.1187	0	0.0031	0	0	0	0.0312	0	NA	0.1195
P09620	0.4348	3	0.2894	2	0.2785	2	0.2689	2	NA	0.4231
P32489	0.7748	2	0.1036	0	0	0	0.0811	0	NA	0.1921
Q12124	0.8979	1	0.7981	1	0.7007	2	0.6845	2	NA	0.9952
P32354	0.6042	3	0.38	3	0.2539	2	0.2627	2	NA	0.423
Q3E798	0.4943	1	0.0115	0	0	0	0.1264	0	NA	0.1075
P38162	0.5102	2	0.1276	0	0	0	0.1276	0	NA	0.2214
P50087	0.1631	0	0.0172	0	0	0	0.0386	0	NA	0.1335
Q04533	0.2265	1	0.1156	1	0.1171	1	0.0971	1	NA	0.2464
Q12041	0.6178	2	0.4869	1	0.3979	1	0.3874	1	TRU	0.7066
P47169	0.2635	2	0.0631	0	0	0	0.0548	0	NA	0.2539
P21826	0.1787	0	0.0379	0	0	0	0.0433	0	NA	0.2353

P53045	0.1036	0	0.068	0	0.0939	0	0.1003	0	NA	0.1535
Q08471	0.9666	3	0.8283	6	0.6455	6	0.5898	5	NA	0.9986
P32334	0.8752	3	0.4342	3	0.4556	3	0.5406	4	NA	1
P54199	0.5916	3	0.4699	3	0.3246	3	0.3115	3	NA	0.9244
P50276	0.1603	1	0.0035	0	0	0	0.0505	0	NA	0.1184
P32357	0.2958	2	0.0845	0	0	0	0.1127	0	NA	0.2822
P18962	0.1553	0	0.0183	0	0	0	0.0391	0	NA	0.1445
Q05031	0.1266	0	0.048	0	0	0	0.0546	0	NA	0.1869
P38121	0.3986	2	0.2383	1	0.2113	1	0.2227	2	NA	0.5805
Q3E705	0.7554	2	0.206	1	0.2618	1	0.2918	1	NA	0.8195
Q05775	0.8604	2	0.6226	2	0.5585	3	0.566	3	NA	0.9931
Q03764	0.2978	2	0.073	0	0	0	0.0843	0	NA	0.3801
P43613	0.4203	2	0.0949	0	0.1559	1	0.1627	1	NA	0.1719
Q12284	0.2806	0	0.0255	0	0	0	0.0663	0	NA	0.1448
P32474	0.1857	1	0.0348	0	0	0	0.0348	0	NA	0.1333
P38137	0.1878	0	0.0681	0	0	0	0.0792	0	NA	0.3832
P32352	0.1351	0	0	0	0	0	0	0	NA	0.1089
P08640	0.8581	1	0.7162	5	0.5838	2	0.6781	1	NA	1
P40464	0.164	0	0.0064	0	0	0	0.0289	0	NA	0.1469
P22146	0.3417	2	0.0859	1	0.0948	1	0.1449	1	NA	0.8482
Q07540	0.3046	0	0.0402	0	0	0	0	0	NA	0.1167
Q05670	0.3486	2	0.0502	0	0	0	0.0591	0	NA	0.1778
Q99341	0.3789	1	0.0435	0	0	0	0.1118	0	NA	0.2417
P39958	0.1929	0	0.0266	0	0	0	0.0599	0	NA	0.1938
Q12154	0.2429	1	0.0734	0	0	0	0.0706	0	NA	0.2253
P38211	0.0647	0	0	0	0	0	0	0	NA	0.1064
P37303	0.1447	0	0.0026	0	0	0	0.0233	0	NA	0.1363
P48566	0.4423	2	0.297	2	0.2512	1	0.3096	1	NA	0.7027
P40358	0.3829	1	0.1814	2	0.1535	2	0.1426	2	NA	0.3215
P36005	0.2379	1	0.0115	0	0	0	0.0577	0	NA	0.2326
P39518	0.0927	0	0	0	0	0	0.0134	0	NA	0.187
Q08601	0.4792	1	0.3681	1	0.3588	1	0.3565	2	NA	0.8477
P38156	0.316	3	0.1124	1	0.1743	2	0.1743	2	NA	0.3289
Q03667	0.8462	1	0.641	2	0.6667	2	0.6474	2	NA	0.9913
P38994	0.5558	1	0.4904	3	0.4146	2	0.3967	3	NA	0.7495
P38860	0.3185	2	0.1197	0	0	0	0.0869	0	NA	0.4643
P05085	0.408	3	0.1966	1	0	0	0.1761	1	TRU	0.785
P53933	0.4549	3	0.414	3	0.2641	3	0.2675	3	NA	0.7723
Q04549	0.0951	0	0	0	0	0	0.0141	0	NA	0.109
P53309	0.669	2	0.5106	3	0.3486	2	0.2764	1	NA	0.8921
P50275	0.6136	6	0.296	2	0.2192	3	0.2384	2	NA	0.6101
P08566	0.1209	0	0.0214	0	0	0	0.0195	0	NA	0.1723
Q12691	0.2539	4	0.0412	0	0.0587	1	0.0687	0	NA	0.3306
P38263	0.4475	3	0.2431	1	0.2403	1	0.2486	1	NA	0.3915
P40956	0.7121	2	0.2828	1	0.3207	2	0.3106	2	TRU	0.9364
P32477	0.1755	1	0.0295	0	0	0	0.0428	0	NA	0.1601
P42944	0.8584	3	0.6443	4	0.4846	4	0.5136	3	TRU	0.9884
P32479	0.3238	2	0.1714	1	0.1321	2	0.144	1	TRU	0.6439
Q05787	0.2449	2	0.1116	1	0	0	0.108	1	NA	0.45
Q12060	0.4734	3	0.3033	1	0.2561	2	0.2418	2	NA	0.7579
P10591	0.4081	2	0.2508	2	0.1199	1	0.1215	1	NA	0.4502
P32337	0.1148	1	0.0404	0	0	0	0.0266	0	NA	0.1181

P21954	0.1472	0	0.0257	0	0	0	0.035	0	NA	0.1458
P40886	0.2004	1	0.0598	0	0.0756	1	0.0896	1	NA	0.1697
P43579	0.5694	3	0.409	2	0.3873	2	0.396	2	NA	0.6326
Q08118	0.5222	3	0.1638	0	0	0	0.0802	0	NA	0.1712
Q99312	0.18	0	0.0133	0	0	0	0.0311	0	NA	0.1376
P38063	0.184	0	0.0123	0	0	0	0.0337	0	NA	0.1338
Q12446	0.8136	1	0.7757	1	0.7836	1	0.7852	1	NA	0.9993
P42951	0.3871	3	0.2405	3	0.2125	3	0.201	2	NA	0.5709
Q02783	0.2591	1	0.0024	0	0	0	0.0218	0	NA	0.1504
P35192	0.5228	2	0.307	2	0.2854	1	0.2974	1	TRU	0.3436
P38132	0.2923	4	0.1077	0	0.0982	1	0.1077	1	NA	0.2812
P32783	0.3922	1	0.2202	2	0.1353	1	0.1628	1	NA	0.3564
P39014	0.2859	2	0.2109	2	0.2188	2	0.2313	2	NA	0.4292
P40850	0.2904	3	0.0976	0	0.0928	1	0.112	1	NA	0.5032
P53725	1	1	0.6344	1	0.8978	2	0.8441	1	NA	0.9903
P10507	0.2424	1	0.0411	0	0	0	0.0455	0	NA	0.1421
P35719	0.7489	3	0.0183	0	0	0	0.0868	0	NA	0.1613
P47069	0.5073	6	0.2537	1	0.2889	2	0.2625	2	NA	0.8576
Q03151	0.1798	0	0.0381	0	0	0	0.0599	0	NA	0.1632
Q08923	0.83	2	0.7589	2	0.6798	1	0.6838	2	NA	0.995
Q04603	0.6224	1	0.5051	1	0.449	1	0.449	1	NA	0.6495
P38195	0.7237	2	0.6187	2	0.6109	3	0.607	3	NA	0.9393
Q04406	0.4545	1	0.0535	0	0	0	0.0642	0	NA	0.1692
Q06706	0.2305	3	0.0578	0	0.0638	1	0.0808	1	NA	0.1892
P53200	0.3387	1	0.0484	0	0	0	0.0565	0	NA	0.1464
P53970	0.1016	0	0	0	0	0	0.0244	0	NA	0.1922
P62651	0.1912	0	0	0	0	0	0	0	NA	0.1498
P27882	0.5661	1	0.2646	0	0	0	0.2381	0	NA	0.5142
P39727	0.2506	1	0.0843	0	0	0	0.094	0	NA	0.2251
P46949	0.8972	3	0.8984	2	0.7846	2	0.7907	2	NA	0.9981
Q07928	0.3404	0	0.0142	0	0	0	0.078	0	TRU	0.1319
P36033	0.097	0	0.0211	0	0	0	0.0253	0	NA	0.1186
Q06636	0.1872	1	0.0639	0	0	0	0.137	0	NA	0.1125
P53973	0.2181	2	0.0552	0	0.068	1	0.0765	1	NA	0.2203
Q03532	0.2198	1	0.1267	1	0.1069	1	0.1149	1	NA	0.3205
P39984	0.2743	1	0.1721	0	0	0	0.1072	0	NA	0.4118
Q03281	0.5762	1	0.4118	1	0.3484	1	0.3484	1	NA	0.8488
P32480	0.2423	2	0.1154	2	0.1131	1	0.128	1	TRU	0.2241
Q12122	0.1727	2	0.0773	0	0.0932	0	0.1114	0	NA	0.1525
Q04638	0.1099	0	0	0	0	0	0.0237	0	NA	0.1007
P21374	0.4936	1	0.1362	0	0	0	0.2255	0	NA	0.4087
P38169	0.1696	0	0.0043	0	0	0	0.0152	0	NA	0.1152
P38691	0.7376	3	0.62	7	0.551	7	0.4947	8	NA	0.9978
P21965	0.192	1	0.0427	0	0	0	0.064	0	NA	0.1298
P43564	0.5536	4	0.4464	6	0.4119	5	0.4063	5	NA	0.8319
P28321	0.1565	0	0.0703	0	0	0	0.1022	0	NA	0.2617
Q96VH5	0.3814	0	0.2371	0	0.3711	1	0.4021	1	NA	0.5838
Q06324	0.9857	2	0.6375	1	0.3992	1	0.4257	1	NA	0.9824
Q05812	0.9162	3	0.8887	7	0.8159	7	0.7692	7	NA	0.9998
Q12117	0.2031	1	0.0938	1	0.1375	1	0.1344	1	NA	0.175
P53869	0.2442	0	0	0	0	0	0.1512	0	NA	0.112
Q07938	0.1246	0	0.0356	0	0	0	0.0178	0	NA	0.1365

Q02866	0.5931	4	0.3088	2	0.2859	2	0.3186	2	NA	0.9569
P28003	0.6877	2	0.3874	0	0.3438	2	0.3148	0	NA	0.9699
P35197	0.6222	2	0.5227	3	0.4489	2	0.4347	2	NA	0.8211
P53961	0.1485	0	0.0131	0	0	0	0.0655	0	NA	0.1119
P30777	0.0893	0	0	0	0	0	0.0373	0	NA	0.1196
P08539	0.3263	2	0.0869	0	0.1059	1	0.1208	1	NA	0.2277
P32784	0.3399	2	0.1858	3	0.1884	2	0.191	2	NA	0.2706
P06774	0.9019	2	0.8642	3	0.8302	3	0.7396	3	TRU	0.9989
Q08220	0.2464	1	0.0163	0	0	0	0.0489	0	NA	0.2797
Q96VH4	0.1658	0	0.0881	0	0	0	0.0725	0	NA	0.1622
Q12276	0.614	6	0.4326	5	0.4061	3	0.4133	4	NA	0.9868
P32589	0.404	3	0.1732	0	0.1082	1	0.114	1	NA	0.2884
Q05827	0.7458	1	0	0	0	0	0.3729	0	NA	0.7221
P32464	0.218	1	0.0702	0	0	0	0.1253	1	NA	0.2015
P32466	0.1446	1	0.0705	1	0.0847	1	0.0741	1	NA	0.2243
Q6Q5K6	0.3088	0	0.1324	0	0	0	0.2794	0	NA	0.2613
P0CY09	0.5905	1	0.0762	0	0	0	0.0667	0	NA	0.2307
P47155	0.3153	1	0.1724	1	0.2069	1	0.202	1	NA	0.2036
P39004	0.1912	1	0.0298	0	0	0	0.0825	0	NA	0.1289
Q03824	0.3163	2	0.0099	0	0	0	0.0397	0	NA	0.1857
Q12056	0.141	0	0	0	0	0	0.109	0	NA	0.1758
Q12055	0.2589	0	0.1168	0	0.1472	0	0.1574	0	NA	0.2757
P01094	1	1	0.9853	1	1	1	0.8971	1	NA	0.747
P07278	0.5096	1	0.2981	1	0.2933	2	0.2957	1	NA	0.5053
P53158	0.8486	2	0.4954	1	0.4862	1	0.4633	1	NA	0.9749
Q08979	0.5161	4	0.3518	3	0.3564	3	0.3656	3	NA	0.8589
P36035	0.2127	1	0.1266	0	0.1364	1	0.1315	1	NA	0.2249
P34239	0.4143	2	0.1944	2	0.1848	2	0.2283	2	NA	0.895
P22134	0.2635	1	0	0	0	0	0.0338	0	NA	0.123
P19659	0.8307	6	0.6846	7	0.5347	6	0.5208	6	NA	0.9966
P25046	0.6545	2	0.5455	2	0.4682	1	0.4455	1	NA	0.91
P22855	0.181	2	0.024	0	0	0	0.0295	0	NA	0.1922
Q03829	0.3288	2	0.1793	1	0.2011	1	0.2201	1	NA	0.3653
P11746	0.8462	2	0.8287	1	0.7657	1	0.7517	1	TRU	0.9942
P38760	0.4492	2	0.1002	1	0.0653	1	0.0744	1	NA	0.27
P33441	0.6862	3	0.3776	1	0.375	1	0.3827	1	NA	0.5765
P32492	0.2182	4	0.0421	0	0	0	0.0394	0	NA	0.3124
P53742	0.3951	3	0.1914	0	0	0	0.144	0	NA	0.2633
Q07913	0.1071	0	0	0	0	0	0.0327	0	NA	0.1108
Q03718	0.1061	0	0	0	0	0	0.0072	0	NA	0.1328
P36139	0.5406	1	0.212	0	0	0	0.2226	0	NA	0.8017
P39535	0.2645	2	0.0465	0	0	0	0.0613	0	NA	0.2165
P07269	0.6476	4	0.6208	5	0.4973	3	0.517	4	TRU	0.9849
P40155	0.2915	0	0.0402	0	0	0	0.1357	0	NA	0.3756
Q03180	0.6831	1	0.1969	1	0	0	0.1046	0	NA	0.3539
P39966	0.4289	1	0.2414	2	0.2392	2	0.2328	2	NA	0.4654
P38797	0.2711	1	0.0292	0	0	0	0.0729	0	NA	0.2702
Q01939	0.1506	0	0.0247	0	0	0	0.0444	0	NA	0.1789
P30620	0.3964	3	0.1679	1	0.112	1	0.1573	1	NA	0.4528
P07390	0.2352	1	0.0429	0	0	0	0.0368	0	NA	0.1662
P40100	0.1914	0	0	0	0	0	0.0825	0	NA	0.1822
P08468	0.1675	0	0.0025	0	0	0	0.0225	0	NA	0.1585

P32901	0.2712	2	0.1015	0	0.1215	1	0.1215	0	NA	0.25
P30402	0.2467	0	0.0441	0	0	0	0.1145	0	NA	0.2897
P32628	0.7739	3	0.598	2	0.4221	2	0.4121	2	NA	0.9453
Q12021	0.3893	2	0.1561	0	0	0	0.1067	0	NA	0.3228
Q00578	0.4009	4	0.2183	1	0.1696	1	0.1922	1	NA	0.5707
P53331	0.2839	1	0.094	0	0	0	0.1175	0	NA	0.2597
P03871	0.2547	1	0.0777	0	0	0	0.0751	0	NA	0.1533
P21538	0.7543	4	0.6593	3	0.6333	4	0.6	5	TRU	0.9986
P38330	0.3447	3	0.2845	3	0.2257	3	0.2257	3	NA	0.5427
P25298	0.2408	2	0.096	1	0	0	0.1241	0	NA	0.2887
Q02204	0.5038	2	0.2008	0	0.2008	1	0.2045	0	NA	0.5683
P02400	0.6727	1	0.3091	1	0.4182	1	0.4182	1	NA	0.7721
P36520	0.413	2	0.1273	1	0.1242	0	0.1491	1	NA	0.2823
P46784	0.2571	0	0.1333	0	0	0	0.219	0	NA	0.1885
P25042	0.8804	1	0.8125	4	0.5788	2	0.4891	2	TRU	0.992
Q04031	0.8809	2	0.5447	1	0.4085	1	0.4213	1	NA	0.6632
Q12212	0.1495	0	0.0113	0	0	0	0.0193	0	NA	0.1279
P33330	0.1797	0	0.0506	0	0	0	0.0557	0	NA	0.1502
P40073	0.4905	1	0.3324	1	0.3433	1	0.2943	1	NA	0.6571
P20134	0.8799	3	0.7389	6	0.624	6	0.5927	6	TRU	0.9997
P17065	0.7181	4	0.4427	1	0.3729	1	0.4045	1	NA	0.9876
P40014	0.638	1	0.267	0	0.1991	0	0.1855	1	NA	0.6322
P53172	0.4231	2	0.4061	2	0.3776	2	0.3757	2	NA	0.6772
Q02775	0.9555	3	0.6545	3	0.4241	2	0.4634	2	NA	0.8316
Q12038	0.2178	2	0.0765	1	0.0852	2	0.0968	2	NA	0.3443
Q05515	0.3285	2	0.2391	2	0.2017	2	0.2287	2	NA	0.425
P42845	0.9595	1	0.9667	3	0.85	2	0.8119	2	TRU	0.9944
P47821	0.1424	0	0	0	0	0	0.065	0	NA	0.2777
P32867	0.6483	2	0.2	0	0	0	0.0931	0	NA	0.1752
P38742	0.6082	5	0.4215	4	0.3656	3	0.3778	4	NA	0.9675
Q6Q547	0.7586	1	0.2759	0	0	0	0.5862	0	NA	0.804
Q12129	0.3028	1	0.055	0	0	0	0.0642	0	NA	0.1313
P36003	0.6121	5	0.3772	3	0.3772	3	0.3922	4	NA	0.9988
P36059	0.2671	1	0.0653	0	0.0831	0	0.1365	0	NA	0.1841
P40354	0.256	2	0.0656	0	0	0	0.0875	0	NA	0.1813
P49686	0.8907	1	0.8	3	0.693	3	0.7047	3	NA	1
P38738	0.3947	4	0.1384	0	0.1605	0	0.2135	0	NA	0.9661
Q03266	0.2219	0	0.0027	0	0	0	0.0455	0	NA	0.1441
P33302	0.2032	3	0.0748	0	0.0794	2	0.0841	2	NA	0.5003
P51533	0.2334	4	0.0889	1	0.1029	2	0.1132	3	NA	0.5717
P25362	0.0837	0	0	0	0	0	0.0233	0	NA	0.1378
P27514	0.2964	3	0.1387	2	0.1253	2	0.1286	2	NA	0.5434
P12868	0.2293	3	0.0175	0	0	0	0.0311	0	NA	0.2111
P16862	0.2367	2	0.0751	0	0.0647	0	0.0803	0	NA	0.4944
P39008	0.5289	2	0.2887	2	0.3349	1	0.2679	2	NA	0.533
P40303	0.3071	1	0.1654	0	0.1142	0	0.1142	0	NA	0.2668
Q03776	0.1636	0	0	0	0	0	0.0239	0	NA	0.111
P40359	0.2066	0	0.0563	0	0	0	0.1455	0	NA	0.3047
P07275	0.1252	0	0.0157	0	0	0	0.0209	0	NA	0.1541
P09368	0.1933	1	0.0021	0	0	0	0.0336	0	NA	0.188
P47065	0.3401	1	0.0375	0	0	0	0.0634	0	NA	0.263
P53335	0.7085	2	0.6199	1	0.4945	1	0.524	1	NA	0.9145

P32327	0.1542	2	0.0263	0	0	0	0.0263	0	NA	0.1314
Q12465	0.1959	1	0.0344	0	0.0213	0	0.0369	0	NA	0.4218
Q06407	0.7166	2	0.5738	5	0.557	5	0.5342	6	NA	0.9987
Q06108	0.7396	6	0.4543	4	0.362	4	0.4229	3	NA	0.9993
P06781	0.0833	0	0	0	0	0	0.0208	0	NA	0.1021
Q07844	0.4002	4	0.2091	2	0.2091	1	0.2103	1	NA	0.751
Q12487	0.3497	1	0.0736	0	0	0	0.0982	0	NA	0.1377
Q12508	0.3753	1	0.0214	0	0	0	0.0119	0	NA	0.1313
P40858	0.3851	1	0.1677	0	0	0	0.2174	0	NA	0.2824
Q08932	0.7008	5	0.5591	3	0.4514	3	0.4147	3	NA	0.9557
P47150	0.3846	1	0.1538	0	0	0	0.1377	0	NA	0.2378
P03877	0.1735	0	0.0096	0	0	0	0.0145	0	NA	0.1768
P38262	0.2243	1	0.0785	1	0.086	1	0.0953	1	NA	0.2967
P25365	0.6441	2	0.3521	3	0.2113	2	0.2507	2	NA	0.9968
P33754	0.301	0	0	0	0	0	0.0728	0	NA	0.1733
Q08968	0.1541	0	0.0291	0	0	0	0.0305	0	NA	0.1143
P40505	0.8869	4	0.5749	2	0.5596	2	0.5291	2	NA	0.9451
P33421	0.3636	1	0	0	0	0	0	0	NA	0.2152
Q12020	0.4311	2	0.2526	1	0.2449	2	0.2628	2	NA	0.6223
P32900	0.9087	4	0.8243	8	0.6172	4	0.5368	4	NA	0.9969
P38163	0.1614	2	0.0683	0	0.0525	0	0.0515	1	NA	0.2464
P36169	0.8676	1	0.6507	4	0.4451	2	0.4704	2	NA	0.9376
P35210	0.5665	6	0.2689	2	0.2458	2	0.2847	2	TRU	0.984
P09937	0.284	1	0.1834	1	0.1834	1	0.1834	1	NA	0.14
P35177	0.6276	7	0.3881	5	0.3514	5	0.3483	5	NA	0.9733
P38637	0.4702	2	0.2633	1	0.2853	1	0.3197	1	NA	0.6039
P31755	0.3917	3	0.1187	0	0	0	0.1021	0	NA	0.577
Q06593	0.1163	1	0	0	0	0	0.0137	0	NA	0.137
P0CE91	0.1417	0	0	0	0	0	0.025	0	NA	0.0929
P53427	0.1667	0	0	0	0	0	0.025	0	NA	0.0883
P20052	0.2901	1	0.1741	1	0.1843	1	0.1945	1	NA	0.2517
P32854	0.4514	1	0.125	0	0	0	0.1354	0	NA	0.123
P25297	0.1891	1	0.0477	0	0.0596	0	0.063	0	NA	0.2031
P20133	0.1446	0	0	0	0	0	0.0492	0	NA	0.182
P48363	0.397	0	0.0704	0	0	0	0.1457	0	NA	0.3519
P39105	0.2289	1	0	0	0	0	0.0377	0	NA	0.6116
P20604	0.1543	0	0.0032	0	0	0	0.0579	0	NA	0.1243
P25043	0.1494	0	0.0345	0	0	0	0.092	0	NA	0.2111
P39682	0.0859	0	0.0048	0	0	0	0.0207	0	NA	0.1149
Q01329	0.4955	4	0.2025	1	0.1809	1	0.2344	1	NA	0.9463
P40555	0.2909	0	0.0636	0	0	0	0.0818	0	NA	0.2318
P04046	0.1627	0	0.0137	0	0	0	0.0059	0	NA	0.1764
Q08273	0.314	1	0.2149	0	0.2562	1	0.2727	1	NA	0.1992
P53221	0.5354	1	0.0866	0	0	0	0.2283	0	NA	0.2556
P0CX25	0.2391	0	0	0	0	0	0.1196	0	NA	0.1358
P36532	0.381	1	0.0571	0	0	0	0.2	0	NA	0.1324
P45818	0.3103	2	0.2163	2	0.2234	2	0.2287	1	NA	0.2328
Q3E792	0.5556	1	0.1574	0	0	0	0.3704	1	NA	0.159
P53292	0.4145	1	0.2261	0	0	0	0.1739	0	NA	0.5894
P53165	0.8326	4	0.8539	4	0.7428	5	0.7215	4	NA	0.9994
P06700	0.2758	1	0.1352	1	0.1352	1	0.1441	1	NA	0.3
P42933	0.185	0	0.0027	0	0	0	0.0241	0	NA	0.1372

P27692	0.5833	3	0.4948	2	0.4346	2	0.445	2	NA	0.9738
P32909	0.727	2	0.5243	3	0.4351	1	0.4608	2	NA	0.9857
P53127	0.3592	5	0.1119	1	0.087	1	0.0991	1	NA	0.4207
Q06315	0.7164	3	0.538	5	0.4542	5	0.4717	5	NA	0.9968
P38247	0.3704	1	0.0617	0	0	0	0.1111	0	NA	0.3058
P01098	0.7674	1	0.5465	1	0	0	0.5698	0	NA	0.4607
Q02890	0.2782	1	0.1102	1	0.135	1	0.1405	1	NA	0.3679
P33299	0.3854	2	0.3662	2	0.2141	1	0.2206	1	NA	0.366
P40534	0.2759	3	0	0	0	0	0.0046	0	NA	0.1234
P53835	0.1785	0	0.0045	0	0	0	0.0363	0	NA	0.2311
P32849	0.4474	6	0.1506	0	0.1104	0	0.1343	1	NA	0.5524
P53295	0.337	1	0.0353	0	0	0	0.0489	0	NA	0.2917
P11938	0.6445	5	0.5526	4	0.4196	4	0.4135	5	TRU	0.9838
P38251	0.1525	0	0.0113	0	0	0	0.0141	0	NA	0.1592
P0CH08	0.5	1	0.1719	0	0	0	0.1875	0	NA	0.1452
Q03305	0.2476	1	0.1262	0	0.1286	0	0.1286	0	NA	0.233
P40993	0.6061	1	0.3636	1	0.4141	1	0.4596	1	NA	0.8171
Q12277	0.2566	0	0.0038	0	0	0	0.0604	0	NA	0.2002
P40011	0.1197	0	0	0	0	0	0.047	0	NA	0.1272
P13433	0.3257	3	0.0489	0	0	0	0.0718	1	NA	0.5096
Q04172	0.5548	3	0.1732	0	0	0	0.1711	0	NA	0.4194
P53965	0.3381	2	0.1708	0	0.1495	0	0.1601	0	NA	0.2935
P38717	0.214	3	0.0146	0	0	0	0.0374	0	NA	0.1769
P09007	0.8643	1	0.4299	0	0.4344	1	0.4887	1	NA	0.8059
P56508	0.2911	0	0	0	0	0	0.2405	0	NA	0.1685
P53616	0.4286	2	0.2214	1	0.2286	1	0.2976	1	NA	0.9528
P38688	0.3047	1	0.1297	1	0.1359	1	0.1437	1	NA	0.19
Q08217	0.3742	3	0.1017	0	0	0	0.069	0	NA	0.449
P32857	0.1683	1	0.065	1	0.0841	1	0.0803	1	NA	0.1604
P38009	0.1503	1	0.0034	0	0	0	0.0118	0	NA	0.1465
Q06665	0.2182	1	0.0347	0	0.0751	1	0.0766	1	NA	0.186
P32863	0.3619	3	0.2272	2	0.1403	1	0.167	1	NA	0.3757
P38232	0.5503	2	0.287	0	0.2604	0	0.3166	1	NA	0.5774
P25332	0.1171	0	0.015	0	0	0	0.024	0	NA	0.1286
A0A0B7P22	0.2143	0	0	0	0	0	0.3214	0	NA	0.0248
Q03956	0.2251	0	0.037	0	0	0	0.0855	0	NA	0.1427
P38630	0.4332	3	0.2648	2	0.2253	2	0.2416	2	NA	0.4686
P40348	0.1643	0	0.0085	0	0	0	0.034	0	NA	0.1463
P22336	0.2029	1	0.1111	1	0	0	0.1063	0	NA	0.3037
P0CX54	0.3939	0	0.1212	0	0	0	0.1576	0	NA	0.1869
P0C2H6	0.4853	1	0.0735	0	0	0	0.0441	0	NA	0.137
P04449	0.7613	1	0.6452	1	0.6323	1	0.6387	1	NA	0.7853
P22276	0.1488	0	0.0722	1	0	0	0.0661	0	NA	0.2143
P35718	0.2547	1	0.1462	1	0.1509	1	0.1698	1	NA	0.155
Q05022	0.3343	6	0.1382	5	0.144	5	0.1423	5	NA	0.5894
Q99314	0.2016	1	0.0766	0	0.1129	0	0.129	0	NA	0.1427
Q08963	0.437	1	0.1597	0	0	0	0.1723	0	NA	0.2462
P46995	0.4079	3	0.2469	2	0.1787	2	0.1924	2	NA	0.3994
Q02774	0.2095	1	0.1333	0	0.1714	1	0.1857	1	NA	0.1815
Q01590	0.6412	2	0.3971	2	0.4324	2	0.3735	1	NA	0.9251
P16658	0.3687	1	0.0928	0	0	0	0.0955	0	NA	0.1793
P32908	0.622	4	0.0939	0	0	0	0.0204	0	NA	0.3588

P47052	0.1924	1	0.0694	0	0	0	0.0552	0	NA	0.1976
P40092	0.9054	1	0.2365	0	0	0	0.2027	0	NA	0.6809
P34226	0.579	3	0.4339	2	0.3894	2	0.3678	3	NA	0.8087
Q08581	0.9683	2	0.5225	4	0.3009	4	0.2984	2	NA	0.5808
Q03088	0.68	1	0.5358	1	0.5067	3	0.4545	4	NA	0.9935
P38788	0.2398	2	0.0836	0	0.0892	0	0.0985	0	NA	0.1765
P38714	0.14	0	0.0016	0	0	0	0.0093	0	NA	0.1249
P40328	0.5902	3	0.3448	2	0.317	2	0.3554	2	NA	0.9245
P38085	0.2779	2	0.1147	1	0.147	1	0.1664	1	NA	0.6465
Q08579	0.1903	1	0.0367	0	0.0902	1	0.0885	1	NA	0.1382
Q05027	0.3439	1	0.1847	0	0.2293	0	0.2293	0	NA	0.8245
P36040	0.1236	0	0	0	0	0	0	0	NA	0.1318
P38208	0.391	0	0	0	0	0	0	0	NA	0.0964
P32345	0.0552	0	0	0	0	0	0.0162	0	NA	0.1044
P49960	0.3874	3	0.1149	0	0.1104	1	0.1126	1	NA	0.2498
P33411	0.4422	1	0.243	1	0.2869	1	0.2948	1	NA	0.2746
Q07350	0.5677	2	0.2068	0	0	0	0.1805	0	NA	0.7756
Q12428	0.157	0	0.0058	0	0	0	0	0	NA	0.1354
P30656	0.2091	0	0.0244	0	0	0	0.0627	0	NA	0.2128
P25044	0.1522	0	0.0299	0	0	0	0.0507	0	NA	0.1656
P07276	0.6411	4	0.3773	5	0.2958	2	0.2823	0	NA	0.891
P32641	0.4385	3	0.1851	1	0.1988	1	0.214	2	NA	0.447
P25299	0.4831	2	0.2432	1	0.2635	1	0.2838	1	NA	0.5919
Q12495	0.7475	4	0.533	4	0.4406	3	0.4802	3	NA	0.9232
P32529	0.424	1	0	0	0	0	0.048	0	NA	0.1089
P38712	0.3473	2	0.2315	2	0.2116	1	0.1936	1	NA	0.2839
Q08162	0.2547	3	0.047	0	0.043	1	0.0629	1	NA	0.1997
P40992	0.2607	1	0.0409	0	0	0	0.0292	0	NA	0.1659
Q3E811	0.2742	0	0	0	0	0	0.1452	0	NA	0.1791
P0CX36	0.2337	0	0.0421	0	0	0	0.0881	0	NA	0.1483
Q04693	0.2292	4	0.0632	0	0.0595	0	0.0639	0	NA	0.5724
P40018	0.7602	1	0.5765	1	0.5561	1	0.5612	1	NA	0.9592
Q12242	0.4322	4	0.2429	1	0.1759	2	0.1714	2	NA	0.3965
P10662	0.3956	2	0.1308	0	0	0	0.0872	0	NA	0.2993
Q03516	0.2392	2	0.0661	1	0.0609	1	0.0714	1	NA	0.3685
Q04477	0.7418	1	0.1408	0	0	0	0.1127	0	NA	0.2264
Q03148	0.3603	1	0.0034	0	0	0	0.037	0	NA	0.1516
P23201	0.8138	4	0.7121	6	0.6248	8	0.6201	11	NA	0.9996
P38359	0.2084	2	0.0501	0	0	0	0.0675	0	NA	0.1381
P38353	0.0449	0	0	0	0	0	0	0	NA	0.1386
P47079	0.2007	0	0.0528	0	0	0	0.0599	0	NA	0.153
P53851	0.2867	2	0.0829	1	0.0924	1	0.1043	1	NA	0.2101
Q05021	0.7186	2	0.5644	2	0.5847	2	0.578	3	NA	0.987
P27796	0.2878	2	0.0552	0	0	0	0.1247	0	NA	0.3333
P27654	0.5143	1	0.3048	1	0.3381	1	0.3429	1	NA	0.9673
P32521	0.8466	5	0.7365	6	0.673	5	0.673	6	NA	0.9999
P53261	0.4942	2	0.3983	3	0.3868	2	0.3901	2	NA	0.745
P53112	0.8182	3	0.4194	2	0.3138	1	0.261	1	NA	0.7667
P14747	0.3162	3	0.2334	1	0.1474	2	0.144	2	NA	0.266
Q12164	0.1792	0	0.0538	0	0	0	0.0968	0	NA	0.1132
Q12445	0.6555	2	0.3378	1	0.291	1	0.311	1	NA	0.6332
Q07949	0.5466	1	0.5189	1	0.4836	1	0.4181	1	NA	0.7026

P38958	0.5225	1	0	0	0	0	0.1081	0	NA	0.1247
Q9ZZX7	0.0625	0	0	0	0	0	0	0	NA	0.1498
P11154	0.1477	2	0.0229	0	0	0	0.017	0	NA	0.1278
P50861	0.1775	0	0.0178	0	0	0	0.1006	0	NA	0.1366
Q12300	0.2674	2	0.2084	3	0.1599	2	0.1691	2	NA	0.2849
Q12672	0.4562	1	0.1187	0	0	0	0.1875	0	NA	0.203
P0CX46	0.3543	1	0.2874	1	0	0	0.2244	0	NA	0.4613
P0CX86	1	0	1	0	1	0	1	0	NA	0.0248
P25348	0.5628	1	0.235	0	0	0	0.2186	0	NA	0.2586
P35996	0.0652	0	0.0217	0	0	0	0.0362	0	NA	0.1907
P39956	0.6231	2	0.4246	5	0.304	2	0.3229	1	TRU	0.9814
Q02773	0.2246	2	0.084	1	0.0557	1	0.0507	1	NA	0.1654
P50106	0.7299	1	0.5401	0	0.4307	1	0.4088	1	NA	0.5452
P0C0V8	0.2759	0	0.046	0	0	0	0.1724	0	NA	0.1271
P39939	0.605	2	0.4286	0	0.4706	1	0.4706	1	NA	0.7699
Q12745	0.2412	1	0.0155	0	0	0	0.0254	0	NA	0.1253
P50088	0.4632	1	0.0737	0	0	0	0.1368	0	NA	0.1166
P53251	0.9619	1	0.6238	1	0.3095	0	0.3095	0	NA	0.6779
Q06091	0.3486	1	0.0857	0	0	0	0.1029	0	NA	0.3064
Q01684	0.356	1	0.089	0	0	0	0.1152	0	NA	0.1459
P53101	0.2065	0	0.0516	0	0	0	0.0602	0	NA	0.1433
P53201	0.7143	4	0.2941	1	0.2374	1	0.229	1	NA	0.5728
P20053	0.372	2	0.0796	0	0	0	0.0409	0	NA	0.2961
P25375	0.2893	2	0.0042	0	0	0	0.0098	0	NA	0.1567
P39927	0.7059	2	0.4847	2	0	0	0.2965	1	NA	0.8658
P41909	0.1966	2	0.0126	0	0	0	0.0241	0	NA	0.1345
P48581	0.2893	1	0.1746	1	0.1771	1	0.1945	1	NA	0.1775
P20051	0.1374	0	0.022	0	0	0	0.0357	0	NA	0.1396
Q03446	0.6619	1	0.4762	2	0.4238	1	0.4667	1	NA	0.9766
P39531	0.2179	1	0.0595	0	0	0	0.0643	0	NA	0.1973
P25611	0.2764	2	0.0036	0	0	0	0.0325	0	TRU	0.2312
P01120	0.5466	1	0.4658	1	0.4752	1	0.472	1	NA	0.9446
P21672	0.1979	1	0.0633	1	0.0725	1	0.0829	1	NA	0.3315
Q08961	0.1835	0	0.0017	0	0	0	0.0206	0	NA	0.1363
P05743	0.5354	1	0.0866	0	0	0	0.2362	0	NA	0.2556
P0C2H7	0.5294	1	0.0956	0	0	0	0.0882	0	NA	0.1352
Q02753	0.4625	1	0.125	0	0	0	0.175	0	NA	0.1897
P24000	0.7613	1	0.6452	1	0.6323	1	0.6387	1	NA	0.744
P0CH09	0.5	1	0.1719	0	0	0	0.1875	0	NA	0.1452
P49166	1	1	0.6818	0	0	0	0.6023	0	NA	0.6492
Q12530	0.2836	1	0.01	0	0	0	0.1244	0	NA	0.1177
P32385	0.6145	2	0.1526	0	0	0	0.1406	0	NA	0.2454
P22138	0.1845	2	0.049	0	0	0	0.049	0	NA	0.2479
P47076	0.5342	1	0.2112	0	0.2795	0	0.3043	0	NA	0.8482
P20436	0.2808	1	0.089	0	0	0	0.1781	0	NA	0.1206
P23248	0.3451	1	0.0824	0	0	0	0.1255	0	NA	0.1502
Q03430	0.7341	2	0.4792	2	0.3546	1	0.3906	1	NA	0.4315
P39743	0.5913	3	0.251	0	0.2407	0	0.2448	0	NA	0.9459
P36056	0.3662	4	0.1497	0	0	0	0.1067	0	NA	0.3322
P38175	0.9492	1	0.2203	0	0	0	0.1921	0	NA	0.406
P51862	0.5118	5	0.3142	2	0.3024	3	0.323	4	NA	0.9919
P11978	0.8211	6	0.5898	3	0.4521	7	0.4772	9	NA	0.9965

Q05567	0.1477	1	0.0272	0	0	0	0.056	0	NA	0.1642
Q08873	0.35	1	0.11	0	0.205	0	0.245	1	NA	0.5211
P33303	0.1615	0	0.0311	0	0	0	0.0745	0	NA	0.2023
P53009	0.3744	2	0.1866	1	0.1766	1	0.2152	1	NA	0.7726
P53334	0.4171	1	0.2047	1	0	0	0.1995	1	NA	0.551
P18759	0.1702	1	0.0462	0	0	0	0.0356	0	NA	0.1314
Q03144	0.1116	0	0.0179	0	0	0	0.0536	0	NA	0.112
P34252	0.8962	2	0.6468	3	0.5519	2	0.5784	3	NA	0.9511
Q04902	0.1013	0	0	0	0	0	0.0295	0	NA	0.1197
P53937	0.1888	0	0.1259	0	0	0	0.042	0	NA	0.1025
P39007	0.0961	1	0.0265	0	0	0	0.0432	0	NA	0.1126
Q12325	0.2867	3	0.1221	0	0.0974	0	0.1422	0	NA	0.2243
Q06563	0.1675	0	0	0	0	0	0.0254	0	NA	0.0969
P32774	0.3934	1	0.0902	0	0	0	0.2295	0	NA	0.2693
P39078	0.2973	1	0.0455	0	0	0	0.0473	0	NA	0.1725
Q08422	1	1	0.5733	2	0.6467	2	0.6333	2	NA	0.8799
P38197	0.2218	0	0.0195	0	0	0	0.0584	0	NA	0.2084
P28004	0.5699	2	0.5567	2	0.2427	1	0.2533	1	NA	0.5886
P14242	0.4021	2	0.1695	1	0.1203	1	0.1397	1	NA	0.4994
P48567	0.4342	3	0.2308	0	0.1911	0	0.206	1	NA	0.3633
Q06244	0.1024	0	0.0472	0	0	0	0.063	0	NA	0.1397
Q9ZZW1	0.3134	1	0.0149	0	0	0	0	0	NA	0.3531
P37299	0.3506	0	0.1558	0	0	0	0.4026	0	NA	0.6742
P48743	0.5031	2	0.349	4	0.3292	4	0.3218	4	TRU	0.866
P38145	0.1471	0	0.0336	0	0	0	0.0588	0	NA	0.1656
P0CX83	0.5661	2	0.3757	1	0.3915	1	0.381	1	NA	0.7658
P40212	0.4824	1	0.2111	0	0	0	0.1558	0	NA	0.2266
P05738	0.1047	0	0	0	0	0	0.0471	0	NA	0.1334
P0CX29	0.5034	1	0.1655	0	0	0	0.2276	0	NA	0.2554
P0CX40	0.545	2	0.355	1	0.2	1	0.24	1	NA	0.2544
P53330	0.9427	1	0.6752	1	0.586	1	0.6369	1	NA	0.8386
P40204	0.2987	0	0.013	0	0	0	0.2078	0	NA	0.1861
Q12136	0.9279	2	0.7607	5	0.6852	4	0.677	5	NA	0.9783
P43589	0.2254	0	0.0424	0	0	0	0.0469	0	NA	0.2479
Q12314	0.2441	0	0.0094	0	0	0	0.0235	0	NA	0.1989
P38810	0.2637	1	0.1421	2	0	0	0.1206	1	NA	0.4028
P39723	0.7106	4	0.2717	2	0.2347	2	0.2444	2	NA	0.4906
P32580	0.2157	1	0.0122	0	0	0	0.0543	0	NA	0.2976
P32591	0.7285	4	0.5176	2	0.4739	2	0.4812	2	NA	0.9961
P40342	0.1644	0	0	0	0	0	0.0274	0	NA	0.1488
P40528	0.3636	4	0.0632	0	0.1086	0	0.133	0	NA	0.3416
P09733	0.2148	0	0.0179	0	0	0	0.0492	0	NA	0.241
P48606	1	1	0.0849	0	0	0	0.1698	0	NA	0.3913
Q12030	0.6165	2	0.4806	1	0.4417	1	0.4417	1	NA	0.5711
Q03640	0.4188	5	0.2408	3	0.2181	3	0.2272	3	NA	0.7935
Q02939	0.2476	1	0.0702	0	0.0799	0	0.0994	1	NA	0.1571
P89886	0.326	1	0	0	0	0	0.0939	0	NA	0.223
P33298	0.2967	0	0.0397	0	0	0	0.035	0	NA	0.2398
P40327	0.4828	2	0.1808	1	0.1465	1	0.1693	1	NA	0.2802
P50896	0.6266	4	0.4994	4	0.4209	3	0.4281	4	NA	0.9553
P32864	0.2338	3	0.0779	0	0	0	0.0663	0	NA	0.1733
Q9ZZW4	1	1	0.0862	0	0	0	0.2759	0	NA	0.4392

P80210	0.1663	1	0.0485	0	0	0	0.0716	0	NA	0.2
P40893	0.1414	0	0.0253	0	0	0	0.0707	0	NA	0.1455
P39083	0.7071	2	0.5313	5	0.4201	3	0.4201	4	NA	0.9436
P05747	1	1	1	1	1	1	0.9322	1	NA	0.8723
P26321	0.431	2	0.0539	0	0	0	0.1347	0	NA	0.1822
Q12532	0.5568	4	0.3083	3	0.2457	2	0.2688	2	NA	0.8184
Q05468	0.473	1	0.2642	1	0.2531	1	0.26	1	NA	0.469
P38902	0.2	0	0	0	0	0	0.075	0	NA	0.1179
P35997	0.3902	0	0.0488	0	0	0	0.2317	0	NA	0.1204
P0CX30	0.5034	1	0.1655	0	0	0	0.2276	0	NA	0.2554
Q02608	0.1405	0	0	0	0	0	0.0579	0	NA	0.1277
P40963	0.1805	1	0.0473	0	0.1006	1	0.1095	1	NA	0.1868
P53078	0.1321	0	0.0179	0	0	0	0.0179	0	NA	0.1155
P53148	0.6925	4	0.4744	4	0.3926	4	0.3544	4	NA	0.9179
P35208	0.4484	3	0.4141	3	0.2656	3	0.2547	2	NA	0.5003
P15624	0.2252	2	0.0202	0	0	0	0.0235	0	NA	0.1456
P07806	0.2554	2	0.0815	0	0	0	0.0833	0	NA	0.253
P48231	0.2156	2	0.0586	0	0.0552	1	0.0637	1	NA	0.261
P53319	0.1077	0	0.0122	0	0	0	0.0366	0	NA	0.2225
P03876	0.2471	1	0.0234	0	0	0	0.0328	0	NA	0.3259
P53109	0.2544	1	0.093	0	0.1123	1	0.1211	1	NA	0.198
P21147	0.2333	1	0.049	0	0	0	0.0863	0	NA	0.1412
P16550	0.3676	1	0.1651	1	0.1651	1	0.1682	0	NA	0.6418
P38115	0.1366	0	0.0087	0	0	0	0.0465	0	NA	0.1888
P35734	0.7568	1	0.7397	2	0.7021	2	0.6473	2	NA	0.9929
P32907	0.1809	1	0.1064	0	0.1206	1	0.1348	1	NA	0.1677
Q7LHD1	0.9853	2	0.2941	1	0.3235	1	0.3309	1	NA	0.7544
P25642	0.0753	0	0	0	0	0	0.0205	0	NA	0.1
Q04329	0.4488	1	0.189	0	0	0	0.1024	0	NA	0.1427
P26798	0.7796	1	0.7138	2	0	0	0.3882	1	TRU	0.976
Q07843	0.287	0	0	0	0	0	0.0435	0	NA	0.1337
P18963	0.2545	5	0.0883	3	0.0902	3	0.0973	3	NA	0.9224
Q03020	0.1879	0	0.0182	0	0	0	0.0788	0	NA	0.2052
Q04772	0.3433	1	0.1592	0	0	0	0.1891	0	NA	0.2179
Q12072	0.5837	4	0.3596	3	0.2894	3	0.2919	3	NA	0.9376
Q12425	0.427	1	0.0162	0	0	0	0.0595	0	NA	0.1523
Q04431	0.4753	2	0.0857	0	0	0	0.0779	0	NA	0.3489
Q92325	0.2136	1	0.097	1	0.1015	2	0.0879	0	NA	0.2299
P34078	0.8121	2	0.6112	1	0.5248	2	0.4536	2	NA	0.8087
Q06213	0.3949	0	0.2803	0	0	0	0.2166	0	NA	0.2714
Q02574	0.3924	2	0.1962	0	0	0	0.1456	0	NA	0.7327
Q02891	0.1732	1	0.0329	0	0	0	0.0461	0	NA	0.1371
P47050	0.2363	2	0.0297	0	0	0	0.0653	0	NA	0.2246
P18899	1	1	0.9907	1	1	1	1	1	NA	0.9981
P31385	0.4988	1	0.4469	2	0.4593	1	0.437	1	NA	0.6722
Q08949	0.4673	2	0.3317	3	0.2042	1	0.232	2	NA	0.6088
P25559	0.1658	0	0.0026	0	0	0	0.0447	0	NA	0.1345
P32460	0.2746	1	0.0164	0	0	0	0	0	NA	0.1199
Q06143	0.1074	0	0.0235	0	0	0	0	0	NA	0.11
Q99234	0.3279	2	0.2585	1	0.2456	1	0.2439	1	NA	0.3732
P54858	0.7903	3	0.4839	2	0.4677	2	0.4484	2	NA	0.8738
P53095	0.1472	0	0	0	0	0	0.014	0	NA	0.1162

P53978	0.1877	2	0.0852	1	0.0795	1	0.092	1	NA	0.1563
P39112	0.1765	1	0.0392	0	0	0	0.0392	0	NA	0.1581
P14284	0.2493	5	0.0406	0	0	0	0.0512	0	NA	0.1909
P53847	0.2931	2	0.1154	1	0.1074	2	0.1101	1	NA	0.2399
P38246	0.2458	1	0.0395	0	0	0	0.0904	0	NA	0.142
Q03212	0.5291	2	0.2855	2	0.2618	2	0.2564	2	NA	0.6446
Q12429	0.1781	1	0.0526	0	0	0	0.0336	0	NA	0.1384
P39540	0.071	0	0	0	0	0	0	0	NA	0.1036
Q05785	0.8581	3	0.7308	3	0.6966	3	0.6705	3	NA	0.9979
P23337	0.1751	1	0.0805	0	0.0466	0	0.0607	0	NA	0.1292
P28742	0.5986	8	0.1881	2	0.1503	2	0.162	2	NA	0.4208
P42846	0.8816	4	0.6616	5	0.6734	5	0.6142	6	NA	0.9812
Q02799	0.8505	2	0.2658	0	0.196	0	0.1894	0	NA	0.8552
Q04396	0.1934	0	0	0	0	0	0	0	NA	0.1969
P40070	0.6578	1	0.615	1	0.5775	1	0.5882	1	NA	0.9943
P38158	0.2277	1	0.0257	0	0	0	0.0651	0	NA	0.191
P29467	0.1397	0	0.0196	0	0	0	0.0466	0	NA	0.1331
P38157	0.1709	0	0.0043	0	0	0	0.0513	0	TRU	0.1456
Q12343	0.5423	2	0.2958	1	0.3063	1	0.3169	1	NA	0.589
P40185	0.3034	0	0.0276	0	0	0	0.069	0	NA	0.1514
Q12372	0.1973	1	0	0	0	0	0.0292	0	NA	0.3016
P40535	0.9319	4	0.77	5	0.6405	5	0.5571	4	TRU	0.9927
Q00955	0.1818	4	0.0479	0	0	0	0.0412	0	NA	0.2444
Q12184	0.3488	1	0.0872	0	0	0	0.0814	0	NA	0.1943
P14540	0.3092	0	0.0891	0	0	0	0.0947	0	NA	0.3121
P54115	0.226	0	0.06	0	0	0	0.034	0	NA	0.143
Q12433	0.811	3	0.682	3	0.5636	3	0.5265	3	NA	0.9958
Q04601	0.1426	0	0.0276	0	0	0	0.0353	0	NA	0.1341
P53886	0.2929	5	0.0789	1	0.0847	2	0.0938	2	NA	0.4986
P53940	0.5606	4	0.1875	1	0.197	1	0.2197	1	NA	0.7099
Q04212	0.194	0	0	0	0	0	0.0567	0	NA	0.1097
P47064	0.2371	0	0.0155	0	0	0	0.0412	0	NA	0.1962
Q12509	0.1986	1	0.0388	0	0	0	0.0799	0	NA	0.1311
P53290	0.1349	0	0.0176	0	0	0	0.085	0	NA	0.121
Q06629	0.5445	4	0.2374	0	0.1053	0	0.1766	1	NA	0.8108
Q99383	0.7903	2	0.7341	3	0.6142	3	0.6124	3	NA	0.9983
P53389	0.1843	1	0.0188	0	0	0	0.058	0	NA	0.1216
P40481	0.1777	1	0.0531	0	0	0	0.0663	0	NA	0.233
P50095	0.1912	1	0.021	0	0	0	0.0363	0	NA	0.2629
P53219	0.1754	0	0.0175	0	0	0	0.0526	0	NA	0.2736
P38912	0.5817	2	0.4967	1	0.4967	2	0.4902	2	NA	0.5973
P53193	0.5924	1	0.2065	0	0	0	0.1576	0	NA	0.4246
P33417	0.9263	2	0.8224	3	0.7806	3	0.7085	4	TRU	0.998
P32526	0.4938	2	0.2624	2	0.2096	1	0.2484	2	NA	0.6116
P47145	0.0427	0	0	0	0	0	0	0	NA	0.1018
Q06508	0.26	1	0.0067	0	0	0	0.0567	0	NA	0.1164
P39678	0.6927	2	0.4286	1	0.3049	1	0.3241	2	TRU	0.8125
P53341	0.226	1	0.0257	0	0	0	0.0651	0	NA	0.1809
P38986	0.1575	0	0	0	0	0	0.0262	0	NA	0.1945
P46682	0.2979	1	0.1273	1	0.1224	2	0.1446	2	NA	0.3585
P53722	0.2379	0	0.0088	0	0	0	0.0881	0	NA	0.1366
P10823	0.4187	1	0.3029	1	0.2873	1	0.2829	1	NA	0.5485

P05373	0.1784	1	0.0146	0	0	0	0.038	0	NA	0.1412
Q01448	0.8768	2	0.8752	4	0.7536	4	0.7328	5	NA	0.999
P32581	0.3969	3	0.2186	0	0.1426	0	0.1442	0	NA	0.634
P53897	0.9405	2	0.7381	2	0.7024	2	0.6488	2	NA	0.9916
P53115	0.546	6	0.36	3	0.2915	5	0.2921	5	NA	0.9609
Q04746	0.2381	1	0	0	0	0	0	0	NA	0.1145
P43614	0.05	0	0	0	0	0	0	0	NA	0.1031
P25586	0.4715	2	0.1614	0	0.1867	1	0.1994	1	NA	0.3084
P53905	0.4609	1	0.3043	0	0.2957	0	0.2957	0	NA	0.5386
P38633	0.1969	0	0.1339	0	0	0	0.1575	0	NA	0.1105
P29469	0.4113	3	0.2938	3	0.2316	3	0.235	3	NA	0.3486
P40356	0.733	1	0.6826	1	0.534	2	0.5416	2	NA	0.997
P53050	0.7917	1	0.5811	3	0.4342	3	0.4276	3	TRU	0.9754
Q12031	0.2904	2	0.007	0	0	0	0.0522	0	NA	0.2622
P22149	0.7783	7	0.5841	3	0.458	2	0.4493	3	TRU	0.993
Q99299	0.8193	6	0.6741	4	0.3984	3	0.434	3	NA	0.9653
Q07747	0.2766	1	0.0608	0	0	0	0.0881	0	NA	0.1558
Q12354	0.1586	0	0	0	0	0	0	0	NA	0.1246
Q06321	0.4032	4	0.1778	3	0.172	3	0.1778	3	NA	0.71
Q06834	0.1632	0	0.059	0	0	0	0.0625	0	NA	0.1201
P46983	0.6177	2	0.2014	0	0.1843	0	0.2491	0	NA	0.7538
P15801	0.264	3	0.0813	2	0.0678	2	0.0694	2	NA	0.1811
P43572	0.6779	6	0.3642	4	0.2608	3	0.268	3	NA	0.8368
Q08651	0.1788	0	0.0394	0	0	0	0.0879	0	NA	0.1195
P53871	0.2129	0	0.0644	0	0	0	0.0532	0	NA	0.1519
P21268	0.4205	2	0.1181	1	0.0892	1	0.0916	1	NA	0.462
P38140	0.4159	3	0.2401	1	0.1626	2	0.1796	2	NA	0.4932
P18414	0.0776	0	0	0	0	0	0	0	NA	0.0949
P38604	0.1505	1	0.0369	0	0	0	0.0492	0	NA	0.1836
P38748	0.4735	2	0.2017	2	0.188	2	0.1846	2	NA	0.4126
Q06179	0.2359	10	0.0232	0	0	0	0.0346	0	NA	0.185
P17064	0.1407	1	0.015	0	0	0	0.0244	0	NA	0.1993
P48362	0.2766	2	0.1041	1	0.1066	1	0.1066	1	NA	0.2471
Q02786	0.4752	1	0	0	0	0	0	0	NA	0.1794
P43586	0.8627	2	0.5931	2	0.6324	2	0.6324	2	NA	0.7612
P36775	0.3857	4	0.2851	4	0.218	3	0.2215	4	NA	0.6557
P53846	0.2963	0	0.0556	0	0	0	0.0707	0	NA	0.1722
P40958	0.199	0	0.0153	0	0	0	0.0663	0	NA	0.1487
Q08278	0.5495	2	0.2477	0	0.2928	0	0.2568	0	NA	0.5291
P32569	0.5269	4	0.2751	2	0.2416	2	0.2402	2	NA	0.7111
P10869	0.186	0	0.0531	0	0	0	0.0664	0	NA	0.2572
P31380	0.5482	8	0.4041	5	0.3395	4	0.3386	4	NA	0.778
P38297	0.2901	2	0.1041	2	0.0994	2	0.1076	1	NA	0.2188
P47102	0.2081	3	0.0582	1	0.0582	1	0.0717	2	NA	0.2778
P18851	0.3664	2	0.0638	0	0	0	0.0638	0	NA	0.3075
Q04082	0.1357	0	0	0	0	0	0	0	NA	0.1108
P32836	0.2409	1	0.0909	0	0	0	0.1227	0	NA	0.1853
Q12341	0.2246	1	0.0027	0	0	0	0.0428	0	NA	0.1183
P09950	0.2755	2	0.0566	0	0	0	0.0529	0	NA	0.291
Q04458	0.109	0	0.0019	0	0	0	0.0188	0	NA	0.1515
P33416	0.307	1	0.0493	0	0	0	0.0308	0	NA	0.1945
P42833	0.1278	0	0.0093	0	0	0	0.0444	0	NA	0.119

P19807	0.1829	2	0.0249	0	0	0	0.0764	0	NA	0.2386
P10081	0.0937	0	0.0127	0	0	0	0.0304	0	NA	0.1082
P40154	0.9812	1	0.9563	1	0.9563	1	0.9688	1	NA	0.9968
Q12442	0.1199	1	0.0063	0	0	0	0.0946	0	NA	0.1345
P0CT04	0.48	0	0.1867	0	0	0	0.1867	0	NA	0.1264
P24583	0.5248	6	0.3215	3	0.265	2	0.2867	2	NA	0.959
P38439	0.7716	3	0.7478	2	0.6724	2	0.6487	2	NA	0.9818
P32875	0.3527	1	0.2101	1	0	0	0.1643	0	NA	0.2444
P38703	0.2555	1	0.0462	0	0	0	0.1217	0	NA	0.1968
P25579	0.9811	3	0.7221	6	0.4906	4	0.5489	3	NA	0.9918
P39970	0.7628	2	0.5092	2	0.272	0	0.2311	0	TRU	0.7053
P25613	0.2014	1	0.1343	0	0.1413	1	0.1625	1	NA	0.1908
P54783	0.1901	0	0.0228	0	0	0	0.0837	0	NA	0.2727
P39976	0.1331	1	0	0	0	0	0.0161	0	NA	0.1358
O13529	0.1788	0	0	0	0	0	0.0596	0	NA	0.1106
P38248	0.5128	2	0.1002	0	0.1189	1	0.1515	1	NA	0.5336
Q03018	0.2141	4	0.0252	0	0	0	0.0325	0	NA	0.1495
P38312	0.0845	0	0	0	0	0	0	0	NA	0.1303
P40207	0.27	1	0	0	0	0	0.0253	0	NA	0.1528
P25659	0.748	2	0.62	2	0.528	2	0.512	1	NA	0.9885
P43574	0.798	2	0.5137	3	0.4216	3	0.4255	2	TRU	0.9971
Q12247	1	1	0.8278	1	0.6755	1	0.7219	1	NA	0.9079
Q12361	0.4776	3	0.308	1	0.2778	1	0.2643	1	NA	0.8303
P36014	0.2096	0	0	0	0	0	0.0898	0	NA	0.0983
P25373	0.2727	0	0.0273	0	0	0	0.0727	0	NA	0.1084
P17695	0.2657	0	0	0	0	0	0.0559	0	NA	0.1461
P47122	0.439	1	0.2597	1	0.2909	1	0.2805	1	NA	0.6167
Q04399	0.1793	0	0.0395	0	0	0	0.0099	0	NA	0.2144
Q12096	0.1466	0	0.0102	0	0	0	0.0183	0	NA	0.1258
P36156	0.2297	0	0	0	0	0	0.0622	0	NA	0.1483
P04911	0.3636	0	0.2121	0	0	0	0.2727	0	NA	0.7485
P38199	0.4593	3	0.2493	0	0.2651	1	0.2677	1	NA	0.6093
P42950	0.5065	4	0.3058	2	0.1893	0	0.2006	1	TRU	0.6934
Q12390	0.1416	0	0	0	0	0	0.0515	0	NA	0.1249
P47171	0.2561	3	0.0789	2	0.085	2	0.085	2	NA	0.4437
Q04432	0.2278	1	0	0	0	0	0.0422	0	NA	0.1738
Q12329	0.7307	3	0.6347	2	0.6213	2	0.6133	3	NA	0.9897
Q05164	0.8056	3	0.334	1	0.2875	1	0.4012	1	NA	0.9999
P20050	0.3835	2	0.1074	0	0	0	0.1025	0	NA	0.2342
Q07959	0.361	2	0.0792	0	0	0	0.0755	0	NA	0.1348
P40006	0.2622	0	0.0356	0	0	0	0.1333	0	NA	0.1919
P38991	0.3733	1	0.218	1	0.2207	1	0.2153	1	NA	0.2429
P32767	0.111	0	0.0009	0	0	0	0.0065	0	NA	0.1217
P39962	0.4866	1	0.3187	1	0.292	1	0.2863	1	NA	0.4539
P47114	0.4527	2	0.2495	1	0.1831	1	0.1992	1	NA	0.1811
P36004	0.5456	2	0.3757	3	0.3702	2	0.3605	3	NA	0.961
P38998	0.1877	0	0.0375	0	0	0	0.0402	0	NA	0.1948
P43601	0.388	3	0.154	0	0.19	2	0.208	2	NA	0.4631
P14904	0.2821	2	0.0856	0	0	0	0.1245	1	NA	0.3089
P32785	0.1696	1	0.0698	0	0	0	0.0599	0	NA	0.2836
Q02207	0.1411	1	0.0178	0	0	0	0.03	0	NA	0.2803
Q05498	0.254	1	0.1005	0	0	0	0.1164	0	NA	0.1122

Q04952	0.1748	3	0.0185	0	0	0	0.0431	0	NA	0.2088
P61830	0.4706	1	0.3456	1	0.3676	1	0.375	1	NA	0.1827
Q05931	0.3212	2	0.0807	0	0	0	0.0365	0	NA	0.2057
P53096	0.208	1	0.0619	0	0	0	0.0774	0	NA	0.238
P47175	0.1201	0	0.0056	0	0	0	0	0	TRU	0.1324
P12684	0.156	1	0.0411	0	0.0364	1	0.045	1	NA	0.1737
P07260	0.3286	1	0.1127	0	0	0	0.169	0	NA	0.2941
Q06704	0.9451	2	0.5005	4	0.2799	2	0.2755	1	NA	0.8984
P11986	0.1238	0	0.0225	0	0	0	0.0206	0	NA	0.1494
Q12479	0.2179	0	0	0	0	0	0	0	NA	0.1018
Q6B0V8	0.1176	0	0	0	0	0	0	0	NA	0.1681
Q04934	0.7241	4	0.4636	2	0.415	1	0.4194	2	NA	0.9897
P38954	0.2087	1	0.1063	0	0.1404	1	0.1651	1	NA	0.2782
Q04437	0.4436	3	0.1049	0	0.0986	0	0.1304	0	NA	0.6707
P25341	0.6014	3	0.3222	2	0.2806	3	0.2889	3	NA	0.8228
P17423	0.2073	0	0.1176	0	0	0	0.1289	0	NA	0.2092
P23291	0.5279	2	0.4033	3	0.3848	1	0.3885	1	NA	0.721
P47912	0.1354	1	0.0245	0	0	0	0.0432	0	NA	0.1929
P40971	0.4544	2	0.2823	3	0.2823	3	0.2835	3	TRU	0.7246
P50113	0.0699	0	0	0	0	0	0.0294	0	NA	0.1087
P28496	0.256	1	0.1172	0	0.1292	0	0.1531	0	NA	0.1664
P38832	0.5346	1	0.195	0	0	0	0.2264	0	NA	0.2608
P37302	0.2086	0	0.0801	0	0	0	0.0279	0	NA	0.1537
Q03687	0.2	1	0.0864	0	0.163	1	0.158	1	NA	0.1537
P46989	0.3506	1	0.0959	0	0.1476	0	0.2066	1	NA	0.5713
P01097	1	1	0.5765	0	0	0	0.6	0	NA	0.8896
Q12676	0.0656	0	0	0	0	0	0.0187	0	NA	0.1171
P31381	0.2553	2	0.1044	1	0.1489	1	0.1644	1	NA	0.4476
P48236	0.2917	2	0.0347	0	0.1435	0	0.1435	0	NA	0.2317
Q12344	0.6812	2	0.5168	2	0.4295	1	0.4217	1	NA	0.8174
Q06623	0.4061	2	0.171	0	0.0519	0	0.0824	0	NA	0.2648
P43570	0.2113	0	0	0	0	0	0.0523	0	NA	0.1153
Q04429	0.8487	3	0.4563	2	0.4326	3	0.4255	2	NA	0.8642
P38635	0.1612	0	0.0537	0	0	0	0.0507	0	NA	0.1193
P38790	0.075	0	0	0	0	0	0.0143	0	NA	0.1166
P47185	0.1781	2	0.0317	0	0.1164	1	0.1252	1	NA	0.2036
Q12134	0.2099	1	0.0741	0	0	0	0.1152	0	NA	0.1571
P04807	0.1811	0	0.0967	0	0	0	0.072	0	NA	0.1942
P32351	0.4913	1	0.3266	2	0.3699	2	0.3728	2	NA	0.3772
P40069	0.1995	2	0.0422	0	0	0	0.0252	0	NA	0.1914
P53982	0.1738	0	0.0286	0	0	0	0.069	0	NA	0.1482
Q12385	0.1396	0	0.0254	0	0	0	0.0279	0	NA	0.1065
P43598	0.9846	1	0.8923	2	0.8	2	0.7744	2	NA	0.9856
P28239	0.2161	1	0.0581	0	0	0	0.0742	0	NA	0.127
P28743	0.6246	5	0.4079	3	0.3456	2	0.3782	2	NA	0.9838
P22209	0.3494	3	0.0552	0	0	0	0.0782	0	NA	0.1743
P32486	0.4194	1	0.3139	1	0.3319	3	0.3208	3	NA	0.7562
P38852	0.5441	2	0.3912	1	0.2912	1	0.3088	1	NA	0.5698
Q08558	0.2657	1	0.0074	0	0	0	0.048	0	NA	0.1435
P36037	0.1818	1	0.028	0	0	0	0.0336	0	NA	0.1672
Q03214	0.1722	2	0.0376	0	0.0439	0	0.0524	1	NA	0.2299
Q06200	0.3705	1	0.2188	1	0.2366	1	0.2478	1	NA	0.7224

P39009	0.271	2	0.1306	0	0.1092	0	0.1442	1	NA	0.259
P00925	0.1968	0	0.0343	0	0	0	0.0412	0	NA	0.2101
Q99382	0.3204	1	0.1326	0	0.2541	1	0.2818	1	NA	0.334
Q02908	0.1813	0	0.0287	0	0	0	0.0359	0	NA	0.1356
P35731	0.1691	0	0.0144	0	0	0	0	0	NA	0.1897
P38993	0.1572	1	0.0503	0	0	0	0.055	0	NA	0.1472
P38124	0.1825	1	0.0912	0	0.1332	1	0.1515	1	NA	0.2201
Q03655	0.3244	1	0.0763	1	0.084	1	0.1164	1	NA	0.6738
P40367	0.0867	0	0.0096	0	0	0	0.0217	0	NA	0.195
Q07845	0.2658	2	0.0728	0	0.0981	1	0.1076	1	NA	0.23
Q12680	0.1925	3	0.0587	0	0	0	0.0434	0	NA	0.5023
P54854	0.1764	2	0.0317	0	0.1146	1	0.1235	1	NA	0.2036
P47124	0.2525	1	0.0303	0	0	0	0.048	0	NA	0.1839
Q03973	0.7033	2	0.4553	1	0.4715	1	0.4593	1	NA	0.7767
Q02196	0.2079	0	0.005	0	0	0	0.0347	0	NA	0.1585
P38131	0.2414	1	0.0323	0	0	0	0.0474	0	NA	0.158
P53966	0.1398	0	0.0172	0	0	0	0.0077	0	NA	0.1261
P38130	0.0965	0	0.005	0	0	0	0.0371	0	NA	0.1372
P17260	0.6038	1	0.2396	1	0.2268	1	0.2588	1	NA	0.9068
P38210	0.5111	2	0.1611	0	0	0	0.2778	0	NA	0.7876
P38801	0.7554	2	0.4022	1	0.4293	1	0.4239	1	NA	0.5068
P38828	0.4011	0	0.0588	0	0	0	0.0802	0	NA	0.1601
P38872	0.4008	1	0.1519	0	0	0	0.1055	0	NA	0.1764
Q12440	0.1536	1	0.0223	0	0	0	0.027	0	NA	0.1376
Q05123	0.3619	2	0.0728	0	0	0	0.0749	0	NA	0.1807
P19097	0.3466	7	0.1208	1	0.0768	1	0.0806	1	NA	0.9303
Q08421	0.4782	3	0.2112	1	0.1529	1	0.1723	1	NA	0.2497
P38225	0.1405	1	0.009	0	0	0	0.006	0	NA	0.119
P19658	0.3018	2	0.0337	0	0	0	0.0449	0	NA	0.2624
P38231	0.3029	0	0.0057	0	0	0	0.0629	0	NA	0.1627
P0CF35	0.1271	0	0.0805	0	0	0	0.089	0	NA	0.1193
P29704	0.1396	1	0	0	0	0	0.0135	0	NA	0.1098
P39676	0.0752	0	0	0	0	0	0.0301	0	NA	0.1234
Q12035	0.871	2	0.5253	1	0.3641	1	0.3779	1	NA	0.7348
P00360	0.1566	0	0.0753	0	0	0	0.0843	0	NA	0.195
P32775	0.1832	0	0.0057	0	0	0	0.0185	0	NA	0.2027
P51601	0.3457	1	0.2099	0	0.2016	0	0.2099	1	NA	0.203
P47088	0.0844	0	0.005	0	0	0	0	0	NA	0.1105
P0CE11	0.0924	0	0	0	0	0	0	0	NA	0.1199
Q02516	0.5868	1	0.5496	1	0.5413	1	0.5248	1	TRU	0.9767
P32339	0.1167	0	0.0158	0	0	0	0	0	NA	0.1094
P69852	0.5072	0	0.058	0	0	0	0.2029	0	NA	0.132
P17629	0.3684	2	0.1689	1	0.1662	1	0.1875	1	NA	0.4044
Q12039	0.2153	0	0.0028	0	0	0	0.0227	0	NA	0.1586
P41833	0.3133	2	0.1117	1	0.1067	1	0.1033	0	NA	0.4049
P50942	0.3753	2	0.2367	1	0.2434	2	0.2511	2	NA	0.6628
P36132	0.1062	0	0.0181	0	0	0	0.0337	0	NA	0.147
O13555	0.848	1	0.472	0	0	0	0.352	0	NA	0.1655
P40034	0.2744	1	0.0691	0	0	0	0.0793	0	NA	0.1598
P27809	0.2104	1	0.0769	0	0.0882	0	0.0928	0	NA	0.1882
Q01919	0.6075	2	0.5813	3	0.4863	3	0.4913	3	NA	0.9897
P35844	0.3986	2	0.0507	0	0	0	0.0737	0	NA	0.6027

P25389	0.6201	4	0.3905	4	0.325	3	0.3433	3	NA	0.9155
Q06147	0.4818	3	0.2591	1	0.2344	2	0.2562	3	NA	0.7477
P40312	0.3417	0	0.1	0	0	0	0.125	0	NA	0.1675
P53830	0.4912	2	0.2316	1	0	0	0.2281	0	NA	0.3868
P22204	0.3969	2	0.1434	1	0.1364	1	0.1818	0	NA	0.4675
P0CH64	0.2844	1	0.0356	0	0	0	0.0711	0	NA	0.1433
P43550	0.2876	1	0.0423	0	0	0	0.0575	0	NA	0.3519
P54838	0.2534	2	0.0103	0	0	0	0.0377	0	NA	0.2458
Q02710	0.7647	1	0.3476	0	0.4118	0	0.4866	1	NA	0.9339
Q04949	0.1859	0	0	0	0	0	0.0288	0	NA	0.1182
P0CX11	0.1373	0	0.0275	0	0	0	0.0572	0	NA	0.2164
P41912	0.3207	1	0.1392	0	0	0	0.1983	0	NA	0.3279
P42222	0.1396	0	0.0275	0	0	0	0.0526	0	NA	0.2131
P25087	0.2898	2	0.0705	0	0	0	0.1201	0	NA	0.1424
P38224	0.1577	0	0	0	0	0	0.0369	0	NA	0.17
P53739	0.7032	3	0.6047	3	0.5252	2	0.5129	3	NA	0.9977
Q12029	0.2171	0	0.0612	0	0	0	0.0673	0	NA	0.1849
P32621	0.2413	2	0.0734	0	0	0	0.0579	0	NA	0.1657
P38785	0.879	2	0.6815	2	0.4873	2	0.4873	1	NA	0.9762
Q06104	0.3265	1	0.1088	0	0	0	0.1701	0	NA	0.5376
P53171	0.2892	1	0.0488	0	0	0	0.1289	0	NA	0.2598
Q01722	0.7884	2	0.6629	3	0.5393	3	0.5375	2	TRU	0.9984
Q04839	0.9628	1	0.9202	1	0.9043	1	0.8457	1	NA	0.9925
Q05905	0.209	0	0.0205	0	0	0	0.0205	0	NA	0.1708
P39987	0.3339	1	0.205	2	0	0	0.1025	0	NA	0.2077
P32590	0.3535	3	0.1371	1	0.1025	1	0.1111	1	NA	0.2939
Q03213	0.8095	3	0.6871	3	0.5508	3	0.5396	4	TRU	0.9979
P38284	0.8863	1	0.4941	2	0.549	2	0.5765	2	NA	0.9798
Q03419	0.0949	0	0.0063	0	0	0	0.057	0	NA	0.1447
P32361	0.3525	4	0.0843	1	0.078	1	0.104	1	NA	0.3799
P38803	0.3653	1	0.1527	1	0.1407	1	0.1677	1	NA	0.2595
P07170	0.3063	0	0.2432	0	0	0	0.1396	0	NA	0.1466
P32645	0.5543	2	0.1536	0	0.1648	0	0.1648	0	NA	0.1841
P38620	0.1321	0	0.0031	0	0	0	0	0	NA	0.1206
Q04377	0.324	1	0.1794	1	0	0	0.1165	1	NA	0.3132
P34730	0.696	3	0.2381	1	0.1941	1	0.2015	1	NA	0.4615
P53753	0.4808	1	0.1182	0	0	0	0.1415	1	NA	0.9338
Q03103	0.2238	1	0.0231	0	0	0	0.032	0	NA	0.1218
P38913	0.1634	0	0.0261	0	0	0	0.0261	0	NA	0.1817
P52911	0.1708	1	0	0	0	0	0.0516	0	NA	0.192
P42940	0.3027	1	0.0498	0	0	0	0.0651	0	NA	0.1287
P38289	0.2752	1	0.0444	0	0	0	0.0444	0	NA	0.2006
Q04660	0.4994	5	0.3197	2	0.2305	1	0.2429	1	NA	0.6393
Q12497	1	1	0.9892	1	0.9892	1	0.9355	1	NA	0.9871
P38310	0.3333	2	0.1247	0	0.2022	1	0.2172	1	NA	0.5748
P00358	0.1657	0	0.0693	0	0	0	0.0693	0	NA	0.1197
P48015	0.0925	0	0.0075	0	0	0	0.015	0	NA	0.1293
P39732	0.4895	2	0.3487	1	0.3039	1	0.3092	1	NA	0.6093
Q06648	0.953	2	0.6658	3	0.6554	3	0.6397	3	NA	0.9847
P40036	0.6971	2	0.5438	5	0.4015	1	0.3796	1	NA	0.9271
P40056	0.4842	1	0.3649	1	0.4105	1	0.3965	1	NA	0.6866
P32190	0.1678	1	0.0437	0	0	0	0.0592	1	NA	0.3456

Q12692	0.3507	1	0.1716	0	0.2687	1	0.2761	1	NA	0.196
P47026	0.102	0	0	0	0	0	0.0143	0	NA	0.1178
P02309	0.4175	1	0.165	0	0.2233	0	0.2524	0	NA	0.433
P40545	0.1226	0	0.0115	0	0	0	0.0268	0	NA	0.1074
Q6Q546	0.1096	0	0	0	0	0	0.0548	0	NA	0.1051
P28627	0.1368	0	0	0	0	0	0.0421	0	NA	0.1259
P46958	0.4883	1	0.3262	2	0.3241	2	0.3412	2	NA	0.8816
Q06142	0.18	1	0.0058	0	0	0	0.0116	0	NA	0.1376
P40499	0.0937	0	0	0	0	0	0	0	NA	0.1239
Q08227	0.1589	1	0.0026	0	0	0	0.013	0	NA	0.1386
P40206	0.3558	1	0.1298	0	0.149	1	0.1346	0	NA	0.159
P07250	0.4113	1	0.1239	1	0.1296	1	0.1803	1	NA	0.5176
P14832	0.3642	0	0.0432	0	0	0	0.0556	0	NA	0.2945
P25603	0.5077	1	0.3923	0	0.3462	0	0.3923	1	NA	0.532
P0CX08	0.1076	0	0.0319	0	0	0	0.0259	0	NA	0.1892
P53080	1	1	1	1	0.9714	1	0.9429	1	NA	0.9939
Q12452	0.0807	0	0	0	0	0	0.0288	0	NA	0.1057
P40020	0.9441	3	0.8116	10	0.7021	7	0.6598	8	NA	0.9992
P41813	0.7135	2	0.6206	4	0.5139	3	0.4548	4	TRU	0.9985
Q06205	0.648	2	0.699	2	0.6327	2	0.6046	2	NA	0.983
P08431	0.2295	0	0.0519	0	0	0	0.0656	0	NA	0.174
P36088	0.1444	0	0	0	0	0	0.0611	0	NA	0.1435
P40088	0.2203	1	0.0421	0	0	0	0.0767	0	NA	0.1679
Q12315	0.4758	2	0.3216	3	0.2528	1	0.2639	1	NA	0.5725
P43535	0.3936	4	0.0199	0	0	0	0.0293	0	NA	0.3528
P39717	0.3966	6	0.1432	0	0.1364	0	0.1648	1	NA	0.901
Q12068	0.1842	0	0.0205	0	0	0	0.0468	0	NA	0.1725
P32806	0.2948	2	0.0611	0	0.0808	0	0.1048	0	NA	0.2886
P40857	0.106	0	0	0	0	0	0	0	NA	0.0999
P0CE41	0.6578	10	0.4421	8	0.3815	6	0.3862	8	TRU	1
P40480	0.7008	4	0.5605	4	0.5162	4	0.53	5	NA	0.9972
P53941	0.3586	1	0.1241	0	0	0	0.1621	0	NA	0.2898
P25038	0.1849	1	0.0296	0	0	0	0.0266	0	NA	0.1371
P47125	0.2009	1	0.0552	0	0	0	0.0751	0	NA	0.2851
P30605	0.1627	1	0.0856	1	0.0805	1	0.0976	1	NA	0.1223
Q12358	0.2233	0	0.0971	0	0	0	0.1214	0	NA	0.4666
P32807	0.2542	0	0.0282	0	0	0	0.0731	0	NA	0.1998
P54070	0.2623	2	0.0269	0	0	0	0.0336	0	NA	0.1599
Q07648	0.2333	0	0	0	0	0	0.0467	0	NA	0.1216
P40487	0.1341	1	0.0776	0	0.0965	1	0.0941	0	NA	0.1659
Q04067	0.6423	2	0.3942	1	0.2956	1	0.3139	1	NA	0.7203
P32501	0.2907	2	0.0955	0	0	0	0.0871	0	NA	0.3217
Q03096	0.1381	0	0.0166	0	0	0	0.0663	0	NA	0.1085
Q06340	0.6667	2	0.4978	1	0.4145	1	0.4518	2	NA	0.8898
Q04651	0.1477	0	0	0	0	0	0.0795	0	NA	0.1384
P40098	0.2008	1	0.0779	0	0	0	0.0738	0	NA	0.13
P36141	0.0752	0	0	0	0	0	0.0677	0	NA	0.1256
Q08023	0.2075	0	0.0446	0	0	0	0.0429	0	NA	0.1332
P40068	0.8936	3	0.8273	6	0.6984	7	0.6884	7	NA	0.9995
P38777	0.214	0	0.0617	0	0	0	0.0617	0	NA	0.1196
P38143	0.2901	0	0	0	0	0	0.0617	0	NA	0.1115
P34243	0.2577	1	0.0351	0	0	0	0.0512	0	NA	0.1677

Q12398	0.7926	2	0.5691	3	0.4171	1	0.4309	1	TRU	0.9698
P15496	0.3368	1	0.1771	1	0.1979	1	0.1632	1	NA	0.3527
Q08295	0.3701	2	0.073	0	0	0	0.0764	0	NA	0.3306
P47056	0.2277	0	0	0	0	0	0.0089	0	NA	0.1136
P43615	0.27	1	0.0127	0	0	0	0.0338	0	NA	0.1105
P49435	0.0695	0	0	0	0	0	0	0	NA	0.1156
P54074	0.2356	1	0.0593	0	0.0689	0	0.0881	0	NA	0.1624
P13586	0.2621	2	0.0674	0	0	0	0.0874	0	NA	0.1898
P53743	0.7057	2	0.4462	2	0.4968	2	0.519	2	NA	0.9829
P40456	0.2791	2	0.1503	2	0.1413	2	0.1476	3	NA	0.2444
P03870	0.1749	0	0.0165	0	0	0	0.0544	0	NA	0.1282
P40492	0.3101	2	0.0678	0	0	0	0.1047	0	NA	0.4504
P53913	1	1	0.5145	0	0	0	0.4509	0	NA	0.7329
P14065	0.1314	0	0	0	0	0	0.0288	0	NA	0.1636
Q04233	0.708	3	0.6589	5	0.6163	5	0.6021	6	NA	0.9983
Q08726	0.2277	1	0.0432	0	0	0	0.0605	0	NA	0.1311
Q02784	0.16	0	0.1067	0	0	0	0	0	NA	0.0985
Q99393	0.2564	1	0.0801	0	0.1378	1	0.1442	1	NA	0.2636
Q08522	0.2124	0	0	0	0	0	0	0	NA	0.2218
Q06554	0.2976	5	0.0958	1	0.0874	1	0.1003	2	NA	0.3747
P00817	0.3206	1	0.101	0	0	0	0.115	0	NA	0.2148
Q06683	0.1219	0	0.0058	0	0	0	0.0189	0	NA	0.1171
Q06667	0.1246	0	0	0	0	0	0	0	NA	0.0961
Q02932	0.0911	0	0	0	0	0	0.0116	0	NA	0.1149
P40507	0.5444	2	0.325	1	0.3278	1	0.3528	1	NA	0.6216
P14164	0.7729	2	0.7291	2	0.6799	5	0.6224	6	TRU	0.9978
P40516	0.179	1	0.1167	0	0	0	0.1206	1	NA	0.2067
P22696	0.8118	1	0.3824	0	0.4176	1	0.4353	0	NA	0.8264
Q07872	0.6883	2	0.4656	1	0.4696	1	0.4494	1	NA	0.8352
P17214	0.2203	1	0.0758	0	0	0	0.113	0	NA	0.1584
Q03661	0.9505	7	0.8752	9	0.7226	7	0.7304	9	NA	0.9985
P40030	0.1284	0	0	0	0	0	0.0338	0	NA	0.1011
A0A023PZB	0.6508	1	0.1667	0	0	0	0.3333	0	NA	0.3806
Q07651	0.4498	1	0.1877	0	0.2492	1	0.2621	1	NA	0.5611
P32363	0.1327	0	0.0442	0	0	0	0.0642	0	NA	0.2486
P13434	0.5903	1	0.3194	1	0.3264	1	0.3472	1	TRU	0.4243
P48353	0.7902	1	0.2545	0	0.2902	1	0.308	1	NA	0.9219
P53119	0.2352	2	0.0538	1	0.067	1	0.0725	1	NA	0.2582
P20459	0.2796	1	0.0855	0	0.1086	0	0.1053	0	NA	0.132
P53067	0.1444	1	0.0139	0	0	0	0.0209	0	NA	0.1379
Q03036	0.3073	1	0.0447	0	0	0	0.1788	0	NA	0.1268
P43596	0.5006	5	0.3329	3	0.3126	3	0.3253	3	NA	0.6226
P05986	0.304	2	0.1658	0	0	0	0.1156	0	NA	0.2119
Q12350	0.4219	1	0.0565	0	0	0	0.0731	0	NA	0.3019
P13186	0.6347	5	0.544	6	0.4682	6	0.4534	6	NA	0.9968
P27466	0.3587	2	0.1973	1	0.2197	2	0.222	2	NA	0.6069
P36973	0.1436	0	0	0	0	0	0	0	NA	0.1045
P16639	0.0795	0	0.002	0	0	0	0.0119	0	NA	0.1441
Q06405	0.2673	0	0.0099	0	0	0	0.099	0	NA	0.2221
P22140	0.11	0	0	0	0	0	0.0256	0	NA	0.1284
P05453	0.4701	1	0.3839	3	0.3577	3	0.3562	3	NA	0.6312
P43557	0.4251	1	0.0097	0	0	0	0.0193	0	NA	0.1333

P04386	0.4336	3	0.1544	1	0.1112	0	0.1044	0	TRU	0.5
P37261	0.1244	0	0.0415	0	0	0	0	0	NA	0.1338
Q99339	0.25	0	0	0	0	0	0.1724	0	NA	0.2043
P25555	0.4941	3	0.4122	2	0.3162	1	0.3513	1	NA	0.4634
P38988	0.1433	0	0.0133	0	0	0	0	0	NA	0.1441
P49018	0.1825	0	0.0024	0	0	0	0.017	0	NA	0.1178
Q06336	0.6068	3	0.2819	1	0	0	0.167	0	NA	0.3129
Q04410	0.4113	1	0.2527	1	0.2581	1	0.2661	1	NA	0.304
Q03835	0.42	1	0.188	1	0.164	1	0.204	1	NA	0.5394
Q12753	0.9049	2	0.7305	5	0.5692	6	0.5346	5	TRU	0.9996
P53834	0.4379	1	0.0654	0	0	0	0.1569	0	NA	0.2578
Q04697	0.1886	1	0.0273	0	0	0	0.0943	0	NA	0.1601
P35843	0.4908	2	0.1106	0	0	0	0.0622	0	NA	0.6581
P19882	0.3164	2	0.0577	0	0	0	0.0629	0	NA	0.3081
Q08914	0.1013	0	0	0	0	0	0.0295	0	NA	0.1221
P0CS90	0.3654	1	0.2202	2	0.0765	1	0.0795	0	NA	0.222
P02829	0.3865	4	0.1425	2	0.1326	2	0.1481	2	NA	0.4226
P0CY13	0.4454	0	0.1513	0	0	0	0.2437	0	NA	0.2581
P40576	0.5417	1	0.1157	0	0	0	0.0324	0	NA	0.2626
Q9P305	0.8779	2	0.7099	1	0.6336	1	0.6183	1	NA	0.9488
Q06096	0.2718	4	0.0488	0	0	0	0.0859	0	NA	0.2758
Q04895	0.1402	1	0.0047	0	0	0	0.0346	0	NA	0.1682
P40318	0.2593	3	0.1463	1	0.1403	2	0.135	1	NA	0.2301
P38249	0.4741	4	0.2531	2	0.2033	2	0.2158	2	NA	0.5633
Q04409	0.146	0	0.016	0	0	0	0.022	0	NA	0.1526
Q08119	0.4552	3	0.2689	2	0.2479	1	0.2731	1	NA	0.4148
P53337	0.1935	1	0.0097	0	0	0	0.129	1	NA	0.1922
Q04461	0.7208	3	0.513	4	0.474	3	0.4589	4	NA	0.9803
P26793	0.445	2	0.1597	0	0.0812	0	0.1257	0	NA	0.1441
Q12418	0.5936	2	0.2729	0	0	0	0.2291	0	NA	0.6573
P40106	0.144	0	0.024	0	0	0	0.056	0	NA	0.1446
P38682	0.7809	1	0.57	1	0.5578	2	0.5375	2	NA	0.9811
Q03937	0.8951	2	0.6728	1	0.5679	1	0.5494	1	NA	0.9453
Q12520	0.233	1	0	0	0	0	0.0413	0	NA	0.1691
P50094	0.2176	2	0.0115	0	0	0	0.0344	0	NA	0.1364
P22580	0.2787	2	0	0	0	0	0.0146	0	NA	0.3671
P19146	0.105	0	0	0	0	0	0	0	NA	0.1058
P32789	0.5547	1	0.3254	1	0.3151	2	0.358	3	NA	0.9196
Q00381	0.1701	0	0	0	0	0	0	0	NA	0.1069
P38731	0.1292	1	0.0415	0	0.0654	1	0.0702	0	NA	0.1439
P17261	0.0885	0	0	0	0	0	0	0	NA	0.135
P40988	0.221	1	0.029	0	0	0	0.0634	0	NA	0.1514
Q08193	0.4298	2	0.2355	1	0.2417	1	0.2583	1	NA	0.8066
Q08908	0.1153	1	0.0159	0	0	0	0.0159	0	NA	0.1158
P38812	0.1514	0	0.0054	0	0	0	0.0541	0	NA	0.1158
P32642	0.3689	1	0.1189	0	0.1434	1	0.168	0	NA	0.3255
P38970	0.5977	2	0.5158	6	0.517	5	0.4784	7	NA	0.9928
P41809	0.8496	5	0.3213	3	0.2575	3	0.4639	6	NA	1
P15992	0.4486	1	0.2523	0	0.229	0	0.2664	0	NA	0.2218
Q06592	0.1282	0	0	0	0	0	0.0705	0	NA	0.0964
P10592	0.4069	2	0.2598	1	0.1142	1	0.108	1	NA	0.3616
P45820	0.1636	0	0	0	0	0	0	0	NA	0.1053

P10961	0.8103	4	0.7671	7	0.6831	6	0.6567	6	TRU	0.9976
P48570	0.1495	1	0.0748	0	0.0888	1	0.0911	1	NA	0.1264
Q04344	0.1646	0	0	0	0	0	0	0	NA	0.1378
Q99332	0.8173	3	0.392	1	0.2791	2	0.3439	1	NA	0.97
P46972	0.1299	0	0.0169	0	0	0	0.0621	0	NA	0.231
P0CY11	0.5714	1	0.1349	0	0	0	0.2063	0	NA	0.3063
P09064	0.6386	1	0.2351	0	0	0	0.4	1	NA	0.7937
Q3E7A9	0.5342	1	0.0548	0	0	0	0.3288	0	NA	0.2566
Q3E731	0.5306	1	0.5204	0	0.5204	0	0.5	0	NA	0.7092
Q01519	0.1807	0	0.0361	0	0	0	0.2771	0	NA	0.3276
Q07788	0.1253	0	0	0	0	0	0.0601	0	NA	0.112
P46962	0.195	0	0.0155	0	0	0	0.0774	0	NA	0.1197
P35732	0.9485	1	0.8076	4	0.832	3	0.8076	2	NA	0.9993
P89113	0.3115	0	0.0328	0	0	0	0.082	0	NA	0.2404
P06634	0.4354	3	0.3526	3	0.346	3	0.3427	3	NA	0.8991
P36041	0.9525	2	0.8639	4	0.8766	4	0.8418	6	NA	0.999
P21951	0.1787	5	0.0414	0	0	0	0.0392	0	NA	0.5106
P53208	0.1189	0	0.003	0	0	0	0.0335	0	NA	0.142
Q03466	0.4106	3	0.207	1	0.1505	1	0.1346	1	NA	0.7673
Q12396	0.3468	1	0.0383	0	0	0	0.0563	0	NA	0.1788
P32801	0.4562	2	0.2047	2	0.1969	1	0.2266	1	NA	0.7648
P53173	0.0797	0	0	0	0	0	0	0	NA	0.0959
P07149	0.1292	2	0.0161	0	0	0	0.0102	0	NA	0.1861
P45976	0.789	2	0.7737	3	0.5321	2	0.5688	2	NA	0.9658
P13181	0.223	1	0.0523	0	0.1098	1	0.1446	1	NA	0.2642
Q12347	0.2703	0	0.0523	0	0	0	0.0872	0	NA	0.1994
Q08992	0.1013	0	0	0	0	0	0.0295	0	NA	0.124
P32478	0.7627	1	0.3051	0	0	0	0.1235	0	NA	0.4693
P32485	0.1977	1	0.1149	1	0	0	0.069	0	NA	0.2104
Q12214	0.1574	0	0.0277	0	0	0	0.0191	0	NA	0.1768
Q12086	0.1977	1	0.0302	0	0	0	0.0512	0	NA	0.1194
P21623	0.1791	1	0.0187	0	0	0	0.0653	0	NA	0.1701
P39517	0.2648	2	0.2332	1	0.2134	1	0.2194	1	NA	0.1905
P43605	0.4342	2	0.2527	0	0	0	0.1922	0	NA	0.343
P42835	0.7301	4	0.1182	0	0	0	0.1595	1	NA	0.9923
P23776	0.125	0	0.0022	0	0	0	0.0201	0	NA	0.151
Q06551	0.1838	0	0.0167	0	0	0	0.0696	0	NA	0.1866
P38261	0.5963	3	0.3161	3	0.3094	3	0.3054	3	NA	0.7194
P53233	0.2168	0	0	0	0	0	0.0352	0	NA	0.1601
P40008	0.1472	0	0.013	0	0	0	0.0433	0	NA	0.1409
P38911	0.6521	2	0.7567	3	0.6764	3	0.6521	3	NA	0.9948
Q12473	0.118	1	0.0084	0	0	0	0.0028	0	NA	0.118
Q08905	0.0759	0	0.0309	0	0	0	0.0155	0	NA	0.1439
P39726	0.5412	2	0.1706	0	0	0	0.1176	0	NA	0.1359
P36173	0.1626	1	0.0016	0	0	0	0.0423	0	NA	0.1597
Q07830	0.1042	0	0.0069	0	0	0	0	0	NA	0.2062
P39722	0.1541	0	0.0181	0	0	0	0.0317	0	NA	0.1433
P37292	0.2531	1	0.0429	0	0	0	0.0286	0	NA	0.1712
P40531	0.5859	3	0.1963	0	0.1442	1	0.1748	1	NA	0.398
P27472	0.1716	1	0.0851	0	0.0553	0	0.0582	0	NA	0.1227
P16622	0.1858	1	0.0051	0	0	0	0.0458	0	NA	0.1491
Q9URQ5	0.7179	1	0.6154	0	0	0	0.4872	0	NA	0.8732

P40502	0.4841	1	0.0318	0	0	0	0.1083	0	NA	0.1571
P00331	0.1351	0	0.0086	0	0	0	0.0316	0	NA	0.2007
Q12013	0.1736	0	0.008	0	0	0	0.0374	0	NA	0.1707
P39925	0.3246	2	0.1261	0	0.0986	1	0.1196	1	NA	0.5564
Q00776	0.2926	2	0.0947	0	0.0863	0	0.12	0	NA	0.3757
P19880	0.8692	3	0.7169	5	0.5754	4	0.5846	4	TRU	0.9958
P22108	0.3938	1	0.1108	0	0	0	0.1631	0	NA	0.4227
Q8TGM5	0.5224	0	0	0	0	0	0.209	0	NA	0.1963
P16521	0.1868	1	0.067	1	0.0785	1	0.09	1	NA	0.2235
P39985	0.2847	1	0.1096	2	0.1086	1	0.1292	1	NA	0.4013
P32601	0.2098	0	0	0	0	0	0.0699	0	NA	0.1099
Q12168	0.2503	1	0.1399	1	0.1361	1	0.1399	1	NA	0.3409
P39704	0.2791	0	0	0	0	0	0.0372	0	NA	0.1798
P53917	0.4607	2	0.2298	1	0.2183	1	0.2162	1	NA	0.429
P43592	0.5701	2	0.2262	0	0	0	0.1719	0	NA	0.2735
Q07541	0.8054	1	0.1007	0	0	0	0.3758	0	NA	0.6711
Q06135	0.2306	2	0.027	0	0	0	0.0541	0	NA	0.3244
P18494	0.9164	3	0.789	7	0.6932	8	0.6932	7	TRU	0.9994
P32288	0.2892	1	0.0676	0	0	0	0.0784	0	NA	0.2145
Q02979	0.2805	4	0.1038	1	0.103	1	0.13	1	NA	0.3609
P36143	0.5633	1	0.5081	1	0.5097	1	0.4708	3	NA	0.9112
P53192	0.3745	1	0	0	0	0	0.017	0	NA	0.1278
P49095	0.1605	0	0.0058	0	0	0	0.0097	0	NA	0.2273
P38011	0.1034	0	0	0	0	0	0.0157	0	NA	0.1061
P46961	0.2	1	0.0071	0	0	0	0.0964	0	NA	0.2539
Q04924	0.3675	2	0.0598	0	0.0541	0	0.0598	0	NA	0.1852
P40107	0.1009	0	0.0059	0	0	0	0.0861	0	NA	0.1016
P21192	0.8519	4	0.7195	11	0.5221	4	0.4974	3	TRU	0.9969
Q04347	0.7938	2	0.5395	1	0.5645	1	0.5588	2	NA	0.9919
Q12191	0.915	2	0.8299	3	0.6364	1	0.6393	1	NA	0.9886
P53223	0.0962	0	0	0	0	0	0.0711	0	NA	0.1542
P21560	0.3313	2	0.0448	0	0	0	0.1522	0	NA	0.237
P41733	0.0482	0	0	0	0	0	0	0	NA	0.1056
P38779	0.4335	2	0.2074	0	0.2101	1	0.1995	1	NA	0.3423
P53280	0.2977	3	0.0865	1	0	0	0.1194	0	NA	0.4414
P14306	0.4566	1	0.1233	0	0	0	0.0685	0	NA	0.2588
P32798	0.3645	2	0.1777	0	0.2301	1	0.2278	0	NA	0.8466
P0CX13	0.1372	0	0	0	0	0	0.0422	0	NA	0.1168
P32898	0.2285	1	0.0233	0	0	0	0.0162	0	NA	0.1877
P53333	0.2946	1	0.1646	1	0.1612	1	0.1768	1	NA	0.2671
P00044	0.4128	0	0.0734	0	0	0	0.2202	0	NA	0.1949
P47089	0.5202	2	0.0303	0	0	0	0.0808	0	NA	0.1532
P36091	0.1203	0	0.0401	0	0	0	0.0423	0	NA	0.3008
P40087	0.2897	1	0.2196	1	0.1822	1	0.1893	1	NA	0.3598
P53550	0.7588	1	0.6649	5	0.5526	4	0.5464	4	NA	0.9933
P41819	0.2075	0	0.0566	0	0	0	0.0755	0	NA	0.2468
Q04623	0.2031	1	0	0	0	0	0.0177	0	NA	0.1631
Q3E840	0.3537	0	0.0488	0	0	0	0.2195	0	NA	0.108
P39712	0.8094	1	0.5696	4	0.2504	4	0.3593	4	NA	0.9999
Q08906	0.9281	1	0.2614	0	0	0	0.3725	0	NA	0.8044
P25621	0.1758	1	0.0723	1	0.1113	1	0.1309	1	NA	0.1593
Q12178	0.1962	0	0	0	0	0	0.0759	0	NA	0.2339

P04387	0.1425	0	0.0184	0	0	0	0.0391	0	TRU	0.1553
P25653	0.8869	5	0.1883	3	0.179	3	0.3878	8	NA	1
P17649	0.1911	1	0.0616	0	0	0	0.0658	0	NA	0.2733
P28006	0.7503	5	0.3392	2	0.2749	2	0.3405	3	NA	0.9705
Q08559	0.5581	1	0.0698	0	0	0	0.1395	0	NA	0.1464
P38783	0.5	1	0.0308	0	0	0	0.1385	0	NA	0.1172
P40208	0.4	2	0.1429	0	0	0	0.1341	0	NA	0.6215
P15315	0.7556	2	0.4044	1	0	0	0.2844	0	TRU	0.6623
P00045	0.6018	0	0.2124	0	0	0	0.3009	0	NA	0.2566
P35176	0.3111	0	0.1422	0	0	0	0.1156	0	NA	0.1692
P25719	0.2143	0	0.011	0	0	0	0.0495	0	NA	0.1434
Q06032	0.6722	1	0.5083	3	0.471	3	0.4606	2	NA	0.9829
Q06151	0.12	0	0.0114	0	0	0	0.0429	0	NA	0.1346
P24784	0.4376	3	0.3209	3	0.2836	2	0.3063	2	NA	0.7485
P24482	0.3367	3	0.1205	1	0.1205	1	0.1234	1	NA	0.5413
P53720	0.2188	1	0.1068	0	0.1016	0	0.1276	0	NA	0.2238
P48235	0.8578	1	0.3507	0	0.3412	0	0.3934	0	NA	0.8359
P32644	0.4398	2	0.2676	2	0.2596	2	0.2703	2	NA	0.4299
P43555	0.3258	1	0.0989	0	0	0	0.1079	0	NA	0.3814
P34216	0.8125	4	0.6879	5	0.5561	3	0.5134	3	NA	0.9984
Q12003	0.1841	1	0.0055	0	0	0	0.0165	0	NA	0.1653
P12754	0.4962	2	0.3149	2	0.2949	2	0.3088	2	NA	0.9637
P40319	0.1478	1	0	0	0	0	0.1188	1	NA	0.1638
P38332	0.0853	0	0.0026	0	0	0	0.031	0	NA	0.1307
Q12283	0.2583	2	0.05	0	0	0	0.0944	0	NA	0.161
Q04433	0.7917	4	0.7027	5	0.4905	1	0.5417	1	NA	0.9992
Q08907	0.7843	1	0.4853	2	0.5098	2	0.5196	2	NA	0.9995
P04397	0.0873	0	0.0014	0	0	0	0.0157	0	NA	0.1377
P04385	0.1117	0	0	0	0	0	0.0208	0	NA	0.1958
P37020	0.1104	0	0.0141	0	0	0	0.0141	0	NA	0.2313
P46984	1	1	0.9675	2	0.7724	1	0.7561	1	NA	0.5218
P32191	0.1695	0	0.0663	0	0	0	0.0647	0	NA	0.1898
P52910	0.1288	0	0.0264	0	0	0	0.0176	0	NA	0.2214
P40353	0.1276	0	0.0114	0	0	0	0	0	NA	0.1271
P47013	0.1809	1	0.0073	0	0	0	0.0978	1	NA	0.1274
P53759	0.1608	0	0.0355	0	0	0	0.0733	0	NA	0.1828
Q05958	0.6376	2	0.4889	7	0.4435	7	0.4091	5	TRU	0.9909
P53938	0.3716	1	0.1393	0	0.2268	1	0.2295	1	NA	0.2601
Q08299	0.1403	1	0.0182	0	0	0	0.0479	0	NA	0.1902
P02994	0.2489	0	0.0917	0	0	0	0.0808	0	NA	0.1989
P09032	0.391	4	0.1799	2	0.1747	2	0.2024	2	NA	0.3557
P40557	0.2354	2	0.0357	0	0	0	0.0285	0	NA	0.1374
Q12450	0.2415	1	0	0	0	0	0.0145	0	NA	0.1138
P53199	0.086	0	0.0287	0	0	0	0.0201	0	NA	0.1359
P39875	0.5313	1	0.3305	2	0.2365	1	0.2678	1	NA	0.5794
Q08991	0.1147	0	0.0187	0	0	0	0.0427	0	NA	0.1666
P40491	0.3677	1	0.1677	0	0	0	0.1161	0	NA	0.2222
P46998	0.3833	1	0.0167	0	0	0	0.0611	0	NA	0.1938
P53889	0.278	1	0.0579	0	0	0	0.0772	0	NA	0.1838
P53121	0.2905	1	0.2282	1	0.2369	1	0.2431	1	NA	0.2531
Q03557	0.2349	1	0.0129	0	0	0	0.0259	0	NA	0.3166
P39012	0.1515	1	0.0244	0	0	0	0.0423	0	NA	0.1792

P40569	0.4793	1	0.2314	0	0	0	0.2727	0	NA	0.5753
Q12393	0.1809	0	0	0	0	0	0.0205	0	NA	0.2149
P38301	0.275	1	0.0036	0	0	0	0.0357	0	NA	0.2261
Q08929	0.1691	1	0.0033	0	0	0	0.0476	0	NA	0.1861
P48365	0.2466	2	0.0523	1	0.0724	1	0.0885	1	NA	0.3022
P32835	0.2466	1	0.0822	0	0	0	0.1233	0	NA	0.1706
P53551	0.8372	2	0.4612	2	0.4845	2	0.5465	2	NA	0.8645
P08521	0.56	0	0	0	0	0	0.56	0	NA	0.0248
Q03771	0.3514	3	0.0801	0	0.0749	0	0.0904	0	NA	0.1542
Q08981	0.6794	3	0.4163	1	0.3397	1	0.378	1	NA	0.5773
P60010	0.1573	0	0.032	0	0	0	0.0427	0	NA	0.2158
P46367	0.1118	0	0.0096	0	0	0	0.0173	0	NA	0.1333
P39998	0.4955	2	0.3031	2	0.2704	2	0.2795	2	NA	0.5857
P53073	0.3158	1	0.1632	0	0.2	1	0.2263	1	NA	0.3042
Q03071	0.4848	0	0.0101	0	0	0	0.1414	0	NA	0.254
P38347	0.2053	1	0.0644	0	0	0	0.0597	0	NA	0.1378
P43616	0.2786	1	0	0	0	0	0.0249	0	NA	0.1464
Q06001	0.5126	3	0.3326	2	0.2866	2	0.341	3	NA	0.8123
P34756	0.5597	5	0.4267	11	0.3749	10	0.3586	10	NA	0.9998
P53279	0.2816	0	0	0	0	0	0.1437	0	NA	0.153
P20081	0.193	0	0.0351	0	0	0	0.114	0	NA	0.1915
P40466	0.4835	2	0.3264	2	0	0	0.281	1	TRU	0.5763
P18411	0.1717	0	0.0202	0	0	0	0.0758	0	NA	0.169
P38196	0.1753	1	0.0751	1	0.0595	1	0.0642	1	NA	0.1571
Q05015	0.0673	0	0.0224	0	0	0	0.0269	0	NA	0.1251
P40066	0.274	1	0.1726	0	0	0	0.1425	0	NA	0.5255
P24814	0.2485	2	0.1825	3	0.1529	3	0.1434	3	NA	0.2234
Q08685	0.0674	0	0.009	0	0	0	0.0404	0	NA	0.1155
P53968	0.9086	5	0.6917	7	0.587	4	0.5619	6	TRU	0.9743
Q3E7A4	0.6881	1	0.1193	0	0	0	0.1927	0	NA	0.1899
P07255	0.2542	0	0.0169	0	0	0	0.2203	0	NA	0.0993
P53239	0.2342	0	0	0	0	0	0.0348	0	NA	0.2236
Q05080	0.6413	2	0.4619	1	0.3827	1	0.3946	1	NA	0.6432
Q03063	0.9912	1	0.8119	2	0.7898	2	0.7633	3	TRU	0.9984
Q12382	0.2483	1	0.031	0	0.1138	1	0.1414	1	NA	0.1523
P53388	0.2385	2	0.1151	1	0.1414	1	0.1464	2	NA	0.3551
P40568	0.8976	4	0.6111	3	0.4826	2	0.4722	2	NA	0.9714
P47133	0.2363	1	0	0	0	0	0.0103	0	NA	0.1211
P05316	0.1532	1	0	0	0	0	0.0395	0	NA	0.1206
P25585	0.0263	0	0	0	0	0	0	0	NA	0.1092
P32805	0.689	3	0.4482	2	0.3545	1	0.3913	1	TRU	0.8312
P38817	0.6547	3	0.294	2	0.2171	1	0.2085	1	NA	0.7319
Q06543	0.1397	0	0.0551	0	0	0	0	0	NA	0.1498
P00431	0.3712	1	0.1191	0	0	0	0.133	0	NA	0.4568
O13547	0.7689	1	0.3739	1	0.4328	1	0.521	1	NA	0.9993
Q03690	0.3258	4	0.1206	1	0.1159	2	0.1167	3	NA	0.5689
P40987	0.0838	0	0	0	0	0	0.0069	0	NA	0.1161
Q12287	0.9855	1	0.1594	0	0	0	0.3913	0	NA	0.8134
P36075	0.4131	2	0.1196	0	0	0	0.1535	0	NA	0.3277
Q03652	0.3874	2	0.0758	0	0	0	0.0926	0	NA	0.3135
P40366	0.515	0	0.4611	0	0	0	0.3114	0	NA	0.3019
P47110	0.6943	1	0.6229	1	0.64	1	0.6429	1	NA	0.9917

P47138	0.2209	0	0.0058	0	0	0	0.064	0	NA	0.1586
P06103	0.2464	3	0.0092	0	0	0	0.0183	0	NA	0.1439
P40214	0.8772	3	0.5263	3	0.4211	3	0.4064	2	NA	0.8381
Q08967	0.2799	1	0.1639	3	0.1702	2	0.1513	2	NA	0.1632
Q12320	0.1439	0	0.014	0	0	0	0.014	0	NA	0.1177
P47177	0.1411	0	0.0149	0	0	0	0.0272	0	NA	0.1184
P43567	0.0779	0	0	0	0	0	0.0182	0	NA	0.1843
P43553	0.5921	6	0.4091	6	0.3462	3	0.3555	4	NA	0.9848
Q08951	0.3627	1	0.2361	3	0.2479	2	0.2489	3	NA	0.3606
P50273	0.1915	2	0.0073	0	0	0	0.0263	0	NA	0.1579
P47039	0.0946	0	0.018	0	0	0	0.018	0	NA	0.1248
P36122	0.1542	1	0.034	0	0.0588	1	0.0719	1	NA	0.2054
Q03373	0.8235	2	0.6223	2	0.4954	3	0.4458	1	NA	0.9683
Q08225	0.18	0	0.007	0	0	0	0.0323	0	NA	0.1358
Q05871	0.1821	0	0.0071	0	0	0	0.0821	0	NA	0.1209
P32768	0.8347	1	0.648	3	0.3149	3	0.4515	3	NA	1
P32472	0.0963	0	0	0	0	0	0	0	NA	0.1253
P40989	0.2227	4	0.1182	1	0.1108	1	0.1177	1	NA	0.3668
Q03768	0.6528	2	0.2792	0	0	0	0.2113	0	NA	0.4806
P07286	0.1116	0	0	0	0	0	0.0045	0	NA	0.1269
Q08969	1	1	1	1	1	1	1	1	NA	0.9948
Q05926	0.2477	0	0	0	0	0	0.055	0	NA	0.0985
P38523	0.7325	2	0.3158	1	0.2368	0	0.307	0	NA	0.4028
P32769	0.3682	2	0.1489	1	0.1571	1	0.1768	1	NA	0.32
Q12150	0.2346	5	0.0365	0	0	0	0.0548	1	NA	0.5904
P53079	0.3477	1	0.024	0	0	0	0.0552	0	NA	0.1883
P06106	0.1847	0	0.0473	0	0.0541	0	0.0653	0	NA	0.286
Q08496	0.1831	1	0	0	0	0	0.0178	0	NA	0.128
P53911	0.7694	2	0.7482	1	0.7482	1	0.7318	1	NA	0.9947
Q12403	0.3289	1	0.0978	0	0	0	0.1467	0	NA	0.2453
Q08822	0.1918	1	0.019	0	0	0	0.038	0	NA	0.1746
P38866	0.169	0	0.0023	0	0	0	0.0278	0	NA	0.1216
P53848	0.1311	0	0.0085	0	0	0	0.0158	0	NA	0.1509
P50264	0.0906	0	0.0118	0	0	0	0.0236	0	NA	0.1167
P19145	0.1661	1	0.0482	0	0.0548	0	0.0581	0	NA	0.1876
Q07825	0.2577	1	0.0654	1	0.0587	1	0.0708	1	NA	0.6581
Q12333	0.1323	1	0.0306	0	0	0	0.0742	0	NA	0.191
P33892	0.131	1	0.0056	0	0	0	0.0101	0	NA	0.1628
P25596	0.1642	1	0	0	0	0	0.0423	0	NA	0.1635
P53130	0.3571	0	0	0	0	0	0	0	NA	0.1399
P07261	0.5669	2	0.3783	2	0.372	3	0.3605	3	TRU	0.8578
P23293	0.5205	1	0.4475	1	0.4338	1	0.4429	1	NA	0.7913
P24870	0.3466	1	0.2834	2	0.281	2	0.2436	2	NA	0.2023
P40955	0.126	0	0.0134	0	0	0	0.0402	0	NA	0.1315
Q05583	0.0758	0	0	0	0	0	0.0152	0	NA	0.1161
P38273	0.3324	3	0.1151	1	0.1136	1	0.1406	1	NA	0.6711
Q06680	0.3353	2	0.1874	2	0.1768	3	0.1633	2	NA	0.3948
P53264	0.2315	1	0.0584	0	0.0652	0	0.0697	0	NA	0.136
Q05892	0.3971	1	0.0397	0	0	0	0.0722	0	NA	0.2294
O13525	0.2687	1	0.0478	0	0	0	0.0478	0	NA	0.1746
P53622	0.1848	2	0.0724	1	0	0	0.0541	1	NA	0.1684
P43639	0.3345	1	0.2086	1	0.2338	1	0.2302	1	NA	0.5457

P41902	1	1	0	0	0	0	0.2754	0	NA	0.2978
P41811	0.1496	1	0.1012	1	0.1046	1	0.1035	1	NA	0.1981
Q00873	0.4464	1	0.3571	1	0.2857	1	0.308	1	NA	0.2294
P53728	0.487	1	0.1883	0	0	0	0.1526	0	NA	0.5282
P43600	0.3578	1	0.0302	0	0	0	0.125	0	NA	0.2318
Q99321	0.5106	1	0.3777	0	0.2606	0	0.3138	0	NA	0.456
Q12084	0.1293	0	0.0259	0	0	0	0.0647	0	NA	0.1297
Q12389	0.5608	4	0.3638	5	0.3256	4	0.3146	4	NA	0.7426
P69771	0.9461	2	0.25	0	0	0	0.2402	0	NA	0.5792
P53924	0.5249	2	0.433	2	0.3889	3	0.341	2	NA	0.9637
P40564	0.6088	4	0.3773	2	0.2778	1	0.3125	1	NA	0.6195
P35195	0.3269	0	0.0481	0	0	0	0.1923	0	NA	0.3636
P33412	0.1486	0	0.0433	0	0	0	0.0774	0	NA	0.1363
P0CX10	0.1373	0	0.0275	0	0	0	0.0572	0	NA	0.2164
Q12434	0.4257	1	0.0842	0	0	0	0.104	0	NA	0.2147
D6W196	0.2632	1	0.0425	0	0	0	0.085	0	NA	0.2209
P18900	0.3573	2	0.1142	0	0	0	0.1268	0	NA	0.2456
P00410	0.0558	0	0	0	0	0	0	0	NA	0.1016
Q08054	0.7595	2	0.3754	1	0.3988	1	0.4692	2	NA	0.9809
P47130	0.1726	0	0.0071	0	0	0	0.0426	0	NA	0.1772
P38892	0.1821	0	0	0	0	0	0.0344	0	NA	0.1098
Q04930	0.6874	5	0.4797	2	0.4711	2	0.4668	3	TRU	0.9795
P53271	0.3244	0	0.0153	0	0	0	0.042	0	NA	0.1398
P89105	0.415	3	0.2071	1	0.1681	1	0.1699	1	NA	0.2074
P25334	0.2233	0	0.044	0	0	0	0.1006	0	NA	0.1563
P21705	0.6627	1	0.4627	1	0.3922	1	0.4118	1	TRU	0.8702
P53734	0.4213	3	0.2369	2	0.2226	2	0.2289	2	NA	0.2644
P13382	0.4285	5	0.2146	3	0.1614	3	0.1771	2	NA	0.3433
P36152	0.5833	1	0.2356	0	0	0	0.2816	1	NA	0.3534
Q99252	0.4192	1	0.2757	1	0.2692	1	0.2626	2	NA	0.5955
P42842	0.1881	1	0.0011	0	0	0	0.0166	0	NA	0.1356
P32626	0.1806	0	0.0132	0	0	0	0.0441	0	NA	0.207
P40566	0.1741	1	0.0668	0	0.0877	1	0.106	1	NA	0.3226
P47160	0.6814	1	0.5686	3	0.6005	2	0.6078	2	NA	0.9939
Q08649	0.3236	1	0.1551	1	0.1371	1	0.1438	1	NA	0.3546
P39521	0.7703	5	0.6752	5	0.5566	6	0.5491	6	TRU	0.995
Q03254	0.4303	3	0.2964	2	0.2746	2	0.2541	1	NA	0.5763
P40209	0.8107	2	0.6714	5	0.4304	3	0.3786	2	NA	0.9341
Q08236	0.7253	5	0.5306	4	0.466	4	0.4804	4	NA	0.9911
P29366	0.4701	3	0.2033	1	0.196	1	0.216	1	NA	0.7133
P35206	0.2341	1	0.0561	0	0	0	0.1195	0	NA	0.1982
P40465	0.2864	0	0.0376	0	0	0	0.0563	0	NA	0.1529
P00425	0.5563	1	0.0861	0	0	0	0.1788	0	NA	0.2913
P52868	0.2615	1	0.0742	0	0	0	0.0813	0	NA	0.2874
P38821	0.2265	1	0.0306	0	0	0	0.0265	0	NA	0.1966
P35497	0.1261	0	0.0084	0	0	0	0.0448	0	NA	0.1398
P38859	0.4054	4	0.2332	6	0.1518	4	0.1682	4	NA	0.4219
P36170	0.7921	3	0.2387	3	0.1814	3	0.29	3	NA	1
P23900	0.5247	3	0.4649	4	0.4185	5	0.4111	3	NA	0.9456
P41913	0.7931	2	0.6705	4	0.6398	4	0.6111	4	NA	0.998
P23797	0.1546	0	0.0164	0	0	0	0	0	NA	0.1388
P38875	0.1984	1	0.0213	0	0	0	0.0361	0	NA	0.2425

P50944	0.4011	2	0.2496	3	0.2062	4	0.2188	4	NA	0.7009
P53188	1	1	0.9417	2	0.6083	1	0.6417	1	NA	0.9352
P26309	0.4689	3	0.2148	0	0.2066	1	0.2213	1	NA	0.7711
Q06549	0.1479	0	0	0	0	0	0.0845	0	NA	0.1118
P40019	1	1	1	1	1	1	1	1	NA	0.9939
Q05790	0.2867	1	0.0403	0	0	0	0.0498	0	NA	0.3324
P15790	0.1855	1	0	0	0	0	0.0188	0	NA	0.1275
P21592	0.2121	1	0.0498	0	0	0	0.0758	0	NA	0.3195
P43542	0.1583	0	0	0	0	0	0.0607	0	NA	0.12
P38723	0.1522	0	0.0105	0	0	0	0.063	0	NA	0.1218
P32344	0.7297	1	0.6036	1	0.3874	1	0.4144	1	NA	0.1551
Q02554	0.6376	3	0.5023	3	0.4243	2	0.4427	2	NA	0.775
P38823	0.4543	2	0.2981	1	0.1755	1	0.1971	1	NA	0.4772
P38791	0.1499	0	0.0078	0	0	0	0.0491	0	NA	0.1738
P31116	0.1978	1	0	0	0	0	0.0056	0	NA	0.1422
P40546	0.9769	2	0.7514	1	0.5925	1	0.5607	1	NA	0.9627
Q06344	0.793	3	0.6672	4	0.664	4	0.6433	4	NA	0.9806
P24521	0.2106	1	0.0333	0	0	0	0.0466	0	NA	0.1931
P38767	0.2857	1	0.2065	2	0.2272	2	0.2134	1	NA	0.2183
P32353	0.126	0	0.0356	0	0	0	0.0521	0	NA	0.1061
P38894	0.7628	1	0.5293	3	0.4363	8	0.4633	7	NA	1
P39719	0.2554	1	0.1507	1	0.1571	1	0.175	1	NA	0.4478
P33893	0.207	0	0.0092	0	0	0	0.0259	0	NA	0.2498
P00359	0.1687	0	0.0663	0	0	0	0.0602	0	NA	0.1228
P00427	0.223	0	0	0	0	0	0.0743	0	NA	0.1401
P53859	0.4623	2	0.2397	1	0.1575	1	0.2021	1	NA	0.7933
P40509	0.1081	0	0.0034	0	0	0	0	0	NA	0.1396
P27697	0.2735	1	0.0599	0	0.0619	0	0.0778	1	NA	0.1707
P38824	1	1	0.6093	1	0.649	1	0.6424	1	NA	0.9935
P14922	0.6584	3	0.618	3	0.6056	4	0.5756	4	TRU	0.9986
Q12123	0.1841	0	0.0397	0	0.0765	0	0.0878	0	NA	0.1682
P20448	0.5571	3	0.3364	1	0.274	2	0.2688	1	NA	0.3765
Q12432	0.4788	2	0.3092	2	0.3491	1	0.3242	1	NA	0.8412
Q12025	0.361	1	0.1756	0	0	0	0.2098	0	NA	0.2095
P53198	0.2778	1	0	0	0	0	0	0	NA	0.1082
Q03898	0.9725	2	0.6186	3	0	0	0.3608	1	NA	0.5821
P32599	0.2492	0	0.1059	0	0	0	0.0405	0	NA	0.1327
P16892	0.1105	0	0.0652	0	0	0	0.0907	0	NA	0.1984
P32614	0.2234	0	0.066	0	0	0	0.0596	0	NA	0.2666
Q12156	0.5839	1	0.1007	0	0	0	0.1074	0	NA	0.1379
P40529	0.6477	1	0.4497	3	0.3826	1	0.4228	1	NA	0.8574
Q8TGM7	0.5738	0	0.5246	0	0.4754	0	0.5082	0	NA	0.7238
P40493	0.4048	2	0.1696	0	0	0	0.2292	0	NA	0.284
P38356	0.5919	3	0.3489	1	0.3489	1	0.3302	1	NA	0.7309
P38428	0.7931	2	0.4384	1	0.3448	1	0.33	1	NA	0.8696
Q04792	0.2684	2	0.1162	0	0.1077	0	0.1162	0	NA	0.4627
P20449	0.3174	1	0.1826	1	0.1452	1	0.1577	1	NA	0.132
Q04217	0.4404	3	0.2802	4	0.2755	3	0.277	3	NA	0.8461
Q06349	0.224	1	0.0611	0	0.0882	1	0.095	1	NA	0.2047
P47128	1	1	0.708	0	0.823	1	0.7611	1	NA	0.8867
P14020	0.1086	0	0.0037	0	0	0	0.0037	0	NA	0.122
P32461	0.2491	1	0.118	0	0	0	0.1273	0	NA	0.1546

P36039	0.3043	1	0.1225	0	0	0	0.17	0	NA	0.4405
P32803	0.2217	0	0	0	0	0	0.0542	0	NA	0.1876
P40540	0.0922	0	0	0	0	0	0	0	NA	0.1238
Q12431	0.0648	0	0	0	0	0	0	0	NA	0.0938
P53246	0.3233	4	0.0547	1	0.0581	1	0.0756	1	NA	0.2544
P14741	0.2262	0	0.0098	0	0	0	0.0328	0	NA	0.1318
P38260	0.231	1	0.0828	0	0	0	0.0724	0	NA	0.1287
P43561	0.1077	0	0.0338	0	0	0	0.0322	0	NA	0.1457
Q08645	0.1642	1	0.0146	0	0	0	0.0219	0	NA	0.172
P11412	0.2594	1	0.0574	0	0	0	0.095	0	NA	0.1689
P31787	0.4598	0	0.046	0	0	0	0.1724	0	NA	0.1811
P54114	0.1719	1	0.0277	0	0	0	0.0138	0	NA	0.1286
P40994	0.2295	0	0	0	0	0	0	0	NA	0.1314
Q02804	0.1515	0	0.0101	0	0	0	0.0455	0	NA	0.1056
P25296	0.44	1	0.0229	0	0	0	0.1086	0	NA	0.2313
P37267	0.7415	1	0.1429	0	0	0	0.2449	0	NA	0.2628
P15202	0.2155	0	0.1204	0	0	0	0.1029	0	NA	0.2709
P24871	0.3326	1	0.1891	0	0	0	0.0978	0	NA	0.1599
P38719	0.2436	1	0.1392	0	0.0974	1	0.1044	0	NA	0.3374
P27344	0.7413	1	0.4627	1	0.5423	1	0.5075	1	NA	0.9817
Q12743	0.3343	1	0.2199	1	0.2463	1	0.2639	1	NA	0.3237
Q04216	0.4298	2	0.038	0	0	0	0.0556	0	NA	0.1832
Q12015	0.4521	1	0.339	1	0.2808	1	0.2774	1	NA	0.8147
P38737	0.1783	4	0.0155	0	0	0	0.0327	0	NA	0.1834
P00924	0.1991	0	0.0229	0	0	0	0.032	0	NA	0.2064
P38819	0.3396	1	0.0519	0	0	0	0.066	0	NA	0.1646
P25340	0.1015	0	0.0169	0	0	0	0.0486	0	NA	0.1448
Q08913	0.1147	0	0.0187	0	0	0	0.0427	0	NA	0.1692
Q08911	0.1383	0	0.0878	0	0	0	0.0798	0	NA	0.1295
Q01217	0.2086	2	0.0197	0	0	0	0.0603	0	NA	0.4812
P53111	0.1787	0	0.0173	0	0	0	0.0634	0	NA	0.1354
Q03818	0.8067	2	0.3467	0	0	0	0.2467	0	NA	0.199
P32450	0.6702	1	0.3404	1	0	0	0.2553	0	NA	0.3495
Q01896	0.2475	3	0.0422	0	0.0568	1	0.0596	0	NA	0.3847
P53744	0.1052	0	0.0053	0	0	0	0.0232	0	NA	0.1393
P36149	0.1192	0	0	0	0	0	0.0725	0	NA	0.1241
P17891	0.9227	1	0.6438	2	0.5923	1	0.5966	1	NA	0.9691
P43635	0.1276	0	0.0041	0	0	0	0.0206	0	NA	0.1756
Q08387	0.2225	1	0.0201	0	0	0	0.0424	0	NA	0.2509
P33317	0.2449	0	0.0476	0	0	0	0.1973	0	NA	0.2603
Q03653	0.4361	5	0.1125	0	0.1036	0	0.1253	0	NA	0.2641
P25574	0.1513	0	0.0079	0	0	0	0.0092	0	NA	0.1883
P36168	0.3682	2	0.2603	3	0.246	4	0.2351	4	NA	0.5564
P54781	0.0892	0	0	0	0	0	0.0204	0	NA	0.148
P32476	0.0847	0	0	0	0	0	0.0121	0	NA	0.3305
P29703	0.1108	0	0	0	0	0	0.038	0	NA	0.1033
Q03034	0.1431	0	0.006	0	0	0	0.0338	0	NA	0.1574
P38934	0.8532	4	0.3638	0	0.2362	0	0.2234	1	NA	0.6585
P48582	0.4905	3	0.1517	2	0.1635	2	0.1588	2	NA	0.3336
P39101	0.6803	4	0.2839	2	0.2916	1	0.266	1	NA	0.8191
P09440	0.2031	2	0.0492	0	0	0	0.0308	0	NA	0.2259
P27614	0.2066	1	0.059	0	0.0573	0	0.0729	0	NA	0.2666

Q06697	0.402	3	0.2494	0	0.2672	0	0.2672	0	NA	0.638
P32458	0.4651	1	0.2145	1	0.1783	1	0.188	1	NA	0.4595
P35201	0.7322	2	0.5683	3	0.5865	2	0.5938	2	NA	0.9339
P37298	0.337	0	0.0608	0	0	0	0.1381	0	NA	0.219
P25453	0.2665	0	0.0269	0	0	0	0.0838	0	NA	0.3317
P38774	0.1423	0	0.0163	0	0	0	0.0325	0	NA	0.1246
P54005	0.3839	1	0.0685	0	0	0	0.0744	0	NA	0.1747
P39715	0.7311	2	0.3443	0	0.3962	1	0.4434	1	NA	0.9086
Q99247	0.4185	3	0.2894	3	0.2643	4	0.2661	4	NA	0.9398
P36053	0.7862	2	0.5586	1	0.5793	1	0.5448	1	NA	0.9487
P38149	0.2403	0	0.0034	0	0	0	0.0046	0	NA	0.3174
Q12518	0.7555	2	0.6608	5	0.6211	5	0.5705	3	NA	0.9661
P12385	0.1556	0	0.0069	0	0	0	0.0618	0	NA	0.1281
Q02883	0.1603	1	0.0085	0	0	0	0.0171	0	NA	0.1777
P22007	0.1323	0	0.007	0	0	0	0.0348	0	NA	0.15
O13329	0.4664	2	0.3251	1	0.2968	2	0.2951	2	NA	0.3915
P40515	0.1613	0	0	0	0	0	0	0	NA	0.1115
