# Supplementary material for: Intrinsically disordered signaling proteins: Essential hub players in the control of stress responses in Saccharomyces cerevisiae
Source: PLoS One. 2022 Mar 15;17(3):e0265422. doi: 10.1371/journal.pone.0265422 (PMC8923507; doi:10.1371/journal.pone.0265422)
Supplement: S2 Table — (PDF) [file pone.0265422.s013.pdf]

S2 Table. Clustering of GO categories from REVIGO, used for construct TreeMap of Figure 1.

Representative		Term_ID	Description	$\log_{10}$ *p*-value	uniqueness
positive regulation of transcription from RNA polymerase II promoter	1	GO:0045944	positive regulation of transcription from RNA polymerase II promoter	-7.3982	0.512
	2	GO:0045892	negative regulation of transcription DNA-templated	-6.7857	0.511
	3	GO:0051346	negative regulation of hydrolase activity	-5.1773	0.766
	4	GO:0006325	chromatin organization	-4.271	0.82
	5	GO:0035305	negative regulation of dephosphorylation	-4.1596	0.66
	6	GO:0032269	negative regulation of cellular protein metabolic process	-2.6157	0.578
	7	GO:0051248	negative regulation of protein metabolic process	-2.6157	0.598
	8	GO:0040008	regulation of growth	-2.5438	0.67
	9	GO:0051336	regulation of hydrolase activity	-2.4607	0.726
	10	GO:0044092	negative regulation of molecular function	-4.0037	0.746
	11	GO:0051128	regulation of cellular component organization	-2.2129	0.628
	12	GO:0006338	chromatin remodeling	-2.0331	0.84
	13	GO:0007118	budding cell apical bud growth	-1.8594	0.806
	14	GO:1902903	regulation of supramolecular fiber organization	-1.8342	0.591
	15	GO:0034063	stress granule assembly	-1.6396	0.819
	16	GO:0090066	regulation of anatomical structure size	-2.8023	0.766
	17	GO:0035303	regulation of dephosphorylation	-2.2051	0.682
	18	GO:0044087	regulation of cellular component biogenesis	-1.9866	0.66
	19	GO:0071824	protein-DNA complex subunit organization	-1.5978	0.831
	20	GO:0000469	cleavage involved in rRNA processing	-1.5662	0.73
	21	GO:0060962	regulation of ribosomal protein gene transcription from RNA polymerase II promoter	-1.5142	0.677
	22	GO:0032535	regulation of cellular component size	-2.8023	0.665
	23	GO:0043254	regulation of protein complex assembly	-2.1964	0.573
	24	GO:0051129	negative regulation of cellular component organization	-1.9079	0.555
	25	GO:0043620	regulation of DNA-templated transcription in response to stress	-1.5662	0.581
	26	GO:0051246	regulation of protein metabolic process	-1.4713	0.625
	27	GO:0065004	protein-DNA complex assembly	-1.4401	0.799
	28	GO:0065009	regulation of molecular function	-1.8669	0.738
	29	GO:0043618	regulation of transcription from RNA polymerase II promoter in response to stress	-1.5662	0.576
	30	GO:0032879	regulation of localization	-1.4605	0.705
Transposition, RNA-mediated	1	GO:0032197	Transposition, RNA-mediated	-8.2039	0.905
	2	GO:0032196	transposition	-8.2039	0.904
	3	GO:0030036	actin cytoskeleton organization	-4.2958	0.718
	4	GO:0007010	cytoskeleton organization	-2.2658	0.845
	5	GO:0030010	establishment of cell polarity	-2.1964	0.899
	6	GO:0000910	cytokinesis	-2.1964	0.853
	7	GO:0030029	actin filament-based process	-4.1933	0.903

	8	GO:0007163	establishment or maintenance of cell polarity	-2.0721	0.905
	9	GO:0044772	mitotic cell cycle phase transition	-1.6252	0.854
	10	GO:0044770	cell cycle phase transition	-1.5978	0.859
	11	GO:0097435	supramolecular fiber organization	-2.5276	0.85
	12	GO:0044396	actin cortical patch organization	-1.6346	0.873
	13	GO:0000281	mitotic cytokinesis	-1.3905	0.842
	14	GO:0000278	mitotic cell cycle	-1.378	0.851
	15	GO:0030865	cortical cytoskeleton organization	-1.3332	0.806
response to abiotic stimulus	1	GO:0009628	response to abiotic stimulus	-4.6331	0.892
	2	GO:0071214	cellular response to abiotic stimulus	-3.263	0.839
	3	GO:0009991	response to extracellular stimulus	-3.2234	0.847
	4	GO:0009605	response to external stimulus	-2.7575	0.895
	5	GO:0071229	cellular response to acid chemical	-2.3209	0.87
	6	GO:0007266	Rho protein signal transduction	-1.4605	0.677
	7	GO:0001101	response to acid chemical	-2.0331	0.897
	8	GO:0007165	signal transduction	-1.3664	0.571
NLS-bearing protein import into nucleus	1	GO:0006607	NLS-bearing protein import into nucleus	-4.0892	0.892
	2	GO:0006406	mRNA export from nucleus	-2.9441	0.824
	3	GO:0032880	regulation of protein localization	-1.7228	0.717
	4	GO:0034504	protein localization to nucleus	-1.3468	0.942
cell communication	1	GO:0007154	cell communication	-3.0324	0.949
growth	1	GO:0040007	growth	-2.8346	0.987
signaling	1	GO:0023052	signaling	-1.3498	0.988
