# Supplementary material for: Intrinsically disordered signaling proteins: Essential hub players in the control of stress responses in Saccharomyces cerevisiae
Source: PLoS One. 2022 Mar 15;17(3):e0265422. doi: 10.1371/journal.pone.0265422 (PMC8923507; doi:10.1371/journal.pone.0265422)
Supplement: S3 Table — (PDF) [file pone.0265422.s014.pdf]

S3 Table. Number of IDPs identified by each predictor and pLLPs index.

	VSL2		IUPred2		MobiDB		FuzPred	
Range	# Proteins	# TF	# Proteins	# TF	# Proteins	# TF	# Proteins	# TF
[0.0 - 0.1)	402	0	3833	13	3120	11	266	0
[0.1 - 0.2)	1504	5	921	17	1394	24	2702	10
[0.2 - 0.3)	1381	8	584	18	820	29	1027	5
[0.3 - 0.4)	941	12	417	19	514	31	584	9
[0.4 - 0.5)	738	15	301	27	353	24	318	10
[0.5 - 0.6)	544	20	267	20	239	22	274	9
[0.6 - 0.7)	417	25	152	18	134	14	219	11
[0.7 - 0.8)	314	29	99	20	72	11	208	14
[0.8 - 0.9)	234	32	92	15	41	4	302	11
[0.9 - 1.0]	246	24	55	3	34	0	857	91
