# Supplementary material for: Intrinsically disordered signaling proteins: Essential hub players in the control of stress responses in Saccharomyces cerevisiae
Source: PLoS One. 2022 Mar 15;17(3):e0265422. doi: 10.1371/journal.pone.0265422 (PMC8923507; doi:10.1371/journal.pone.0265422)
Supplement: S4 Table — (PDF) [file pone.0265422.s015.pdf]

**S4 Table. Number of IDRs of *S. cerevisiae* proteome according to MobiDB.**

| # of IDR | # proteins | # IDPs | # TFs | #IDTFs |
|---|---|---|---|---|
| 0 | 4289 | 49 | 29 | 0 |
| 1 | 1310 | 139 | 44 | 6 |
| 2 | 534 | 109 | 33 | 9 |
| 3 | 282 | 97 | 24 | 10 |
| 4 | 136 | 40 | 16 | 10 |
| 5 | 72 | 37 | 8 | 4 |
| 6 | 49 | 27 | 11 | 9 |
| 7 | 27 | 9 | 3 | 2 |
| 8 | 13 | 6 | 2 | 1 |
| 9 | 5 | 4 | 0 | 0 |
| 10 | 3 | 2 | 0 | 0 |
| 11 | 1 | 1 | 0 | 0 |
