# Supplementary material for: Intrinsically disordered signaling proteins: Essential hub players in the control of stress responses in Saccharomyces cerevisiae
Source: PLoS One. 2022 Mar 15;17(3):e0265422. doi: 10.1371/journal.pone.0265422 (PMC8923507; doi:10.1371/journal.pone.0265422)
Supplement: S5 Table — (PDF) [file pone.0265422.s016.pdf]

S5 Table. IDP KEGG enrichment using clusterProfiler adding the stress pathways.

ID	Description (# Proteins in the path)	GeneRatio VSL (323)	GeneRatio IUPred (112)	GeneRatio MobiDB(67)
sce04011	MAPK signaling pathway – yeast (114)	42	15	15
Ion Homeostasis (263)		66	17	8
Osmotic Stress (145)		40	13	10
sce04111	Cell cycle – yeast (130)	36	13	8
sce04130	SNARE interactions in vesicular transport (20)	12	NA	NA
Nutrient Adaptation (180)		43	24	14
Heat Shock (101)		7	9	7
Oxidative Stress (138)		33	12	8
sce04144	Endocytosis (78)	20	7	4
sce03022	Basal transcription factors (32)	10	4	2
sce04113	Meiosis – yeast (133)	26	11	7
sce03013	RNA transport (95)	18	8	5
sce03440	Homologous recombination (21)	6	3	1
sce04138	Autophagy – yeast (85)	16	4	2
sce04139	Mitophagy – yeast (40)	9	1	1
Pheromone Response (56)		11	5	4
sce03015	mRNA surveillance pathway (48)	9	3	1
sce03450	Non-homologous end-joining (10)	3	-	-
sce03010	Ribosome (187)	27	10	10
sce03040	Spliceosome (81)	13	6	2
sce03420	Nucleotide excision repair (37)	6	1	1
sce03020	RNA polymerase (30)	5	1	-
sce03018	RNA degradation (64)	9	3	1
sce03410	Base excision repair (18)	3	1	1
sce04141	Protein processing in endoplasmic reticulum (93)	11	3	1
sce03030	DNA replication (31)	3	1	1
sce00190	Oxidative phosphorylation (74)	7	2	1
sce04136	Autophagy – other (23)	2	1	-
sce04145	Phagosome (35)	3	-	-
sce04213	Longevity regulating pathway - multiple species (38)	3	2	1
sce04120	Ubiquitin mediated proteolysis (51)	4	2	1
sce03430	Mismatch repair (20)	1	1	1
sce03050	Proteasome (36)	2	1	-
sce03060	Protein export (22)	1	-	-
sce00770	Pantothenate and CoA biosynthesis (23)	1	1	-
sce04070	Phosphatidylinositol signaling system (23)	1	-	-
sce03008	Ribosome biogenesis in eukaryotes (90)	6	2	1
sce04146	Peroxisome (40)	2	1	1
sce00561	Glycerolipid metabolism (32)	1	-	-
sce00564	Glycerophospholipid metabolism (40)	1	-	-
sce01110	Biosynthesis of secondary metabolites (348)	1	-	-

The annotation universe was downloaded from the KEGG pathway database and those genes not included in this database were added (Kawakami et al., 2016). The total universe obtained was of 2697 genes/proteins. We made a Hypergeometric test with the number of proteins considered IDPs and with an annotation in the expanded dataset. The *p*-value was adjusted for multiple testing by Bonferroni-Hochberg. Symbol (-) means not described.
