# Supplementary material for: Intrinsically disordered signaling proteins: Essential hub players in the control of stress responses in Saccharomyces cerevisiae
Source: PLoS One. 2022 Mar 15;17(3):e0265422. doi: 10.1371/journal.pone.0265422 (PMC8923507; doi:10.1371/journal.pone.0265422)
Supplement: S6 Table — (PDF) [file pone.0265422.s017.pdf]

S6 Table. Distribution of IDTFs in stress response pathways.

Name	ENSEMBL_PRIMARY	Heat shock	Ion homeostasis	Nutrient adaptation	Osmotic stress	Oxidative stress	D_ratio_VSL2	D_ratio_MobiDB
**Rlm1**	YPL089C	+	+	-	+	+	0.8861	0.821
**Ume6**	YDR207C	-	+	+	-	-	0.9713	0.7943
**Mig1**	YGL035C	-	-	+	-	-	1	0.7857
**Dig1***	YPL049C	-	-	-	-	-	0.9912	0.7633
**Mcm1***	YMR043W	-	-	-	-	-	0.8462	0.7517
**Hap2**	YGL237C	-	-	+	-	-	0.9019	0.7396
**Azf1**	YOR113W	-	-	+	-	-	0.8446	0.721
**Gln3**	YER040W	-	-	+	+	+	0.9164	0.6932
**Pho4**	YFR034C	-	+	-	-	-	0.8365	0.6731
**Met4**	YNL103W	-	+	-	-	-	0.8586	0.6696
**Sko1**	YNL167C	-	-	+	+	-	0.9645	0.6615
**Hsf1**	YGL073W	+	-	+	-	+	0.8103	0.6567
**Sok2**	YMR016C	-	-	+	-	-	0.8548	0.6561
**Msn2**	YMR037C	+	+	+	+	+	0.9702	0.6222
**Sfl1***	YOR140W	-	-	-	-	-	0.8799	0.5927
**Sfp1**	YLR403W	-	-	+	+	+	0.8272	0.59
**Yap1**	YML007W	-	+	-	-	+	0.8692	0.5846
**Cyc8**	YBR112C	-	+	+	-	-	0.6584	0.5756
**Crz1**	YNL027W	+	+	-	-	+	0.9086	0.5619
**Hot1**	YMR172W	-	+	-	+	-	0.8095	0.5396
**Hap4**	YKL109W	-	-	+	-	-	0.9404	0.5379
**Haa1**	YPR008W	-	-	+	-	-	0.9049	0.5346
**Rtg1**	YOL067C	-	-	+	-	-	0.6215	0.5254
**Hap5**	YOR358W	-	-	+	-	-	0.5868	0.5248
**Msn4**	YKL062W	+	+	+	+	+	1	0.5063
**Sub1***	YMR039C	-	-	-	+	-	0.8836	0.75
**Mss11***	YMR164C	-	-	+	-	-	0.8522	0.5
**Flo11**	P08640	-	-	+	-	-	0.8581	0.6781
Rgt1	YKL038W	-	-	++	-	-	0.6436	0.4778
Ste12	YHR084W	-	-	-	-	-	0.7631	0.4666
Msn1	YOL116W	-	+	-	+	-	0.5681	0.466
Aft1	YGL071W	-	+	-	-	+	0.7783	0.4493
Rtg3	YBL103C	-	-	+	-	-	0.8704	0.4444
Swi4	YER111C	+	+	-	+	+	0.7164	0.4437
Gat1	YFL021W	-	-	+	+	+	0.798	0.4255
Stp2	YHR006W	-	-	+	-	-	0.7671	0.4159
Rap1	YNL216W	-	+	+	-	-	0.6445	0.4135
Skn7	YHR206W	-	+	-	+	+	0.5788	0.41
Dal81	YIR023W	-	-	+	-	-	0.5278	0.399
Upc2	YDR213W	-	-	-	+	-	0.6473	0.3888
Hap1	YLR256W	+	+	-	-	-	0.6578	0.3862
Smp1	YBR182C	-	+	-	+	-	0.7412	0.3805
Rim101	YHL027W	-	+	-	-	-	0.8032	0.3792
Zap1	YJL056C	-	+	-	-	-	0.7966	0.3648
Tup1	YCR084C	-	+	+	-	-	0.5694	0.3576
Hap3	YBL021C	-	-	+	-	-	0.5903	0.3472
Rds2	YPL133C	-	-	+	-	-	0.5874	0.3363
Rpn4	YDL020C	+	+	+	-	-	0.6742	0.3352
Yap5	YIR018W	-	+	-	-	-	0.8653	0.3347
Cat8	YMR280C	-	-	+	-	-	0.6148	0.3252
Mbp1	YDL056W	-	+	-	-	-	0.6927	0.3241
Gcn4	YEL009C	+	-	+	-	+	0.9893	0.3238
Tec1	YBR083W	-	-	+	-	-	0.6296	0.3004

Mac1	YMR021C	-	+	-	-	-	0.5228	0.2974
Stp1	YDR463W	-	-	+	-	-	0.7168	0.2948
Cup2	YGL166W	-	+	-	-	-	0.7556	0.2844
Ime1	YJR094C	-	-	+	-	-	0.8306	0.2778
Swi6	YLR182W	+	+	-	+	+	0.6015	0.2765
Cad1	YDR423C	-	+	-	+	+	0.6333	0.2714
Mga2	YIR033W	-	-	-	+	-	0.5247	0.2489
Nrg1	YDR043C	-	+	-	-	-	0.6883	0.2338
Aft2	YPL202C	-	+	-	-	-	0.6082	0.2308
War1	YML076C	-	+	-	-	-	0.5138	0.2214

*These proteins participate in others process not reported in Kawakami et al. (2016).

Bold names correspond to IDTFs classified by MobiDB and VSL, while the rest of IDTFs were classified only by VSL.
