# Supplementary material for: Intrinsically disordered signaling proteins: Essential hub players in the control of stress responses in Saccharomyces cerevisiae
Source: PLoS One. 2022 Mar 15;17(3):e0265422. doi: 10.1371/journal.pone.0265422 (PMC8923507; doi:10.1371/journal.pone.0265422)
Supplement: S7 Table — (PDF) [file pone.0265422.s018.pdf]

S7 Table: Yeast IDPs involved in osmotic stress response [a].

Protein	Molecular function	Functional elements located in IDRs	Ref
Msb2	Osmosensor of the Sho branch of this pathway, where Sho functions as a co-osmosensor, involved in the activation of the Hog1 MAPK.	Msb2 contains four IDRs, three located at its extracellular domain, one at the amino-end, and the other two are close by the region interacting with Opy2 that is necessary for the osmostress signal transduction. The remaining one is located at the cytoplasmic region, which associates with the scaffold protein Bem1 to recruit Ste20 to the plasma membrane.	[1]
Opy2	Osmosensor involved in the activation of the Hog1 MAPK.	Opy2 shows two IDRs, both localized to the cytoplasmic domain, which allow the interaction between the adaptor protein Ste50 and Ste11, targeting Ste11 to the plasma membrane, and leading to the activation of the Hog1 osmostress pathway.	[2]
Ste20	Ste20 MAPKs has osmotransducing function to modulate the nutrient adaptation and osmotic stress response.	Ste20 with five IDRs distributed throughout the protein. One of these IDRs overlap with the PXXP motifs, one binding to Bem1 and the other to the Sho1 osmosensor. The next IDR towards the amino terminus includes the Cdc42 binding site located in Ste20 CRIB (Cdc42- and Rac-Interactive Binding) domain.	[3]
Rtg1	Rtg1 is transcription factor involved in the regulation of the mitochondrial retrograde (RTG) pathway and also is essential for peroxi- some proliferation.	Rtg1 shows two IDRs, extending over its DBD and C-terminal domains.	[4]
Sub1	Sub1 protein has a gene-specific function in the recruitment of TBP, TFIIB, and RNAP II/III to promoters during osmotic stress. Also, Sub1 is recruited by Hot1, an IDP, to osmostress gene promoters.	Sub1 contains two IDRs covering most of the sequence, overlapping with phosphorylation sites.	[5,6]
Cyc8	Cyc8 also is part of a coactivator complex able to recruit SWI/SNF and SAGA complexes to promoters.<br>The Cyc8-Tup1 corepressor complex also associates to the ATF/CREB repressor Sko1, which by Hog1 phosphorylation converts this repressor complex into an activator of hyperosmotic stress responsive genes.	Cyc8 has four IDRs harboring putative phosphorylation sites.	[7–9]
Sko1	Sko1 is phosphorylated and converted into a transcriptional activator by Hog1 during hyperosmotic stress. Sko1-Cyc8-Tup1 complex pass from a repressing to an activating state, recruiting other proteins as SAGA histone acetylase complex and SWI/SNF nucleosome-remodeling complex to target promoters in vivo.	The recognized phosphorylation sites of Sko1 fall into two of its three IDRs. Disorder N-terminal region of Sko1 is important for the Sko1-Tup1 interaction, while sumoylation occurs toward C-terminal region, reducing the association of some TFs with chromatin.	[8,10,11]
Sic1	In response to stress conditions Hog1 phosphorylates Sic1 protein at T173, inhibiting Sic1 ubiquitination. Phosphorylated Sic1 cannot activate	Sic1 contains one IDR located at its N-terminal domain, which contain phosphorylation sites.	[2,12,13]

	Clb5,6/Cdc28, affecting DNA replication and delaying G1/S transition.		
Hot1	Hot1 is an inducer of a smaller subset of osmoresponsive genes (GPD1, STL1, GPP2, HGI1, HSP12, NQM1, CTT1, GRE1, SPI1 and DIA3 genes) and, under non-stress conditions it activates STL1.	Hot1 contains four IDR scattered throughout its sequence.	[5,14]
Msn2/ Msn4	Msn2 and Msn4 activate a large group of environmental stress responsive genes (ESR). but it is also implicated in the adjustments of growth (expansion and division), nutrient and metabolic status through complex transcriptional networks.	The IDR located in the N-terminal region contains the Msn2 transcriptional activating domain (AD), an essential region for its function and nuclear localization. IDRs towards its carboxy-terminus contain the NES domain, and sites susceptible to phosphorylation, which controls the Msn2 export from the nucleus to the cytosol.	[15,16]
Mcm1	Mcm1 protein belongs to *S. cerevisiae* MADS box family. Mcm1 regulates the metabolism in response to different arginine levels, and is essential for cell viability, controlling G_1 /S and G_2 /M cell cycle transitions, mating, osmotolerance, recombination, and minichromosome maintenance.	Mcm1 presents one IDR located at its the C terminal, which contains the polyglutamine and the acid domains important for its transcription activity and for the regulation of alpha mating-type specific genes.	[17,18]
Dig1	Dig1 is phosphorylated and its repressing activity is inhibited under cell stress condition.	Dig1 presents three IDRs located to its N- and C-terminal domains. These IDRs contain several phosphorylation sites.	[19–21]
Dot6	Dot6 is a component of the RPD3 histone deacetylase complex RPD3C(L) responsible for the deacetylation of lysine residues in the N-terminal region of the core histones.	Dot6 contains seven IDRs, two of them overlapping with phosphorylation sites.	[22]

[a] These proteins are highlighted in Figures 3 and 4 of the main text.

References

1. Yamamoto K, Tatebayashi K, Saito H. Binding of the Extracellular Eight-Cysteine Motif of Opy2 to the Putative Osmosensor Msb2 Is Essential for Activation of the Yeast High-Osmolarity Glycerol Pathway. Mol Cell Biol. 2016;36: 475–487. doi:10.1128/MCB.00853-15

2. Saito H, Posas F. Response to Hyperosmotic Stress. Genetics. 2012;192: 289–318. doi:10.1534/genetics.112.140863

3. Tanaka K, Tatebayashi K, Nishimura A, Yamamoto K, Yang H-Y, Saito H. Yeast Osmosensors Hkr1 and Msb2 Activate the Hog1 MAPK Cascade by Different Mechanisms. Sci Signal. 2014;7: ra21. doi:10.1126/scisignal.2004780

4. Jia Y, Rothermel B, Thornton J, Butow RA. A basic helix-loop-helix-leucine zipper transcription complex in yeast functions in a signaling pathway from mitochondria to the nucleus. Mol Cell Biol. 1997;17: 1110–1117. doi:10.1128/MCB.17.3.1110

5. Gomar-Alba M, del Olmo M. Hot1 factor recruits co-activator Sub1 and elongation complex Spt4/5 to osmostress genes. Biochem J. 2016;473: 3065–3079. doi:10.1042/BCJ20160463

6. Rosonina E, Willis IM, Manley JL. Sub1 Functions in Osmoregulation and in Transcription by both RNA Polymerases II and III. Mol Cell Biol. 2009;29: 2308–2321. doi:10.1128/MCB.01841-08

7. Wang S, Xing Z, Pascuzzi PE, Tran EJ. Metabolic Adaptation to Nutrients Involves Coregulation of Gene Expression by the RNA Helicase Dbp2 and the Cyc8 Corepressor in *Saccharomyces cerevisiae*. G3 GenesGenomesGenetics. 2017;7: 2235–2247. doi:10.1534/g3.117.041814

8. Proft M, Struhl K. Hog1 Kinase Converts the Sko1-Cyc8-Tup1 Repressor Complex into an Activator that Recruits SAGA and SWI/SNF in Response to Osmotic Stress. Mol Cell. 2002;9: 1307–1317. doi:10.1016/S1097-2765(02)00557-9

9. Tam J, van Werven FJ. Regulated repression governs the cell fate promoter controlling yeast meiosis. Nat Commun. 2020;11: 2271. doi:10.1038/s41467-020-16107-w

10. Pascual-Ahuir A, González-Cantó E, Juyoux P, Pable J, Poveda-Huertes D, Saiz-Balbastre S, et al. Dose dependent gene expression is dynamically modulated by the history, physiology and age of yeast cells. Biochim Biophys Acta BBA - Gene Regul Mech. 2019;1862: 457–471. doi:10.1016/j.bbagrm.2019.02.009

11. Sri Theivakadadcham VS, Bergey BG, Rosonina E. Sumoylation of DNA-bound transcription factor Sko1 prevents its association with nontarget promoters. Mendenhall EM, editor. PLOS Genet. 2019;15: e1007991. doi:10.1371/journal.pgen.1007991

12. Nishizawa M, Kawasumi M, Fujino M, Toh-e A. Phosphorylation of Sic1, a Cyclin-dependent Kinase (Cdk) Inhibitor, by Cdk Including Pho85 Kinase Is Required for Its Prompt Degradation. Yanagida M, editor. Mol Biol Cell. 1998;9: 2393–2405. doi:10.1091/mbc.9.9.2393

13. Verma R, Feldman RMR, Deshaies RJ. SICI Is Ubiquitinated In Vitro by a Pathway that Requires CDC4, CDC34, and Cyclin/CDK Activities. Mol Biol Cell. 1997;8: 11.

14. Rep M, Krantz M, Thevelein JM, Hohmann S. The Transcriptional Response of Saccharomyces cerevisiae to Osmotic Shock. J Biol Chem. 2000;275: 8290–8300. doi:10.1074/jbc.275.12.8290

15. Roetzer A, Gregori C, Jennings AM, Quintin J, Ferrandon D, Butler G, et al. Candida glabrata environmental stress response involves Saccharomyces cerevisiae Msn2/4 orthologous transcription factors. Mol Microbiol. 2008;69: 603–620. doi:10.1111/j.1365-2958.2008.06301.x

16. Sadeh A, Baran D, Volokh M, Aharoni A. Conserved Motifs in the Msn2-Activating Domain are Important for Msn2-Mediated Yeast Stress Response. J Cell Sci. 2012; 3333–3342. doi:10.1242/jcs.096446

17. Jamai A, Dubois E, Vershon AK, Messenguy F. Swapping Functional Specificity of a MADS Box Protein: Residues Required for Arg80 Regulation of Arginine Metabolism. Mol Cell Biol. 2002;22: 5741–5752. doi:10.1128/MCB.22.16.5741-5752.2002

18. Christ C, Tye BK. Functional domains of the yeast transcription/replication factor MCM1. Genes Dev. 1991;5: 751–763. doi:10.1101/gad.5.5.751

19. Bardwell L, Cook JG, Zhu-Shimoni JX, Voora D, Thorner J. Differential regulation of transcription: Repression by unactivated mitogen-activated protein kinase Kss1 requires the Dig1 and Dig2 proteins. Proc Natl Acad Sci. 1998;95: 15400–15405. doi:10.1073/pnas.95.26.15400

20. Chasman D, Ho Y, Berry DB, Nemec CM, MacGilvray ME, Hose J, et al. Pathway connectivity and signaling coordination in the yeast stress-activated signaling network. Mol Syst Biol. 2014;10: 759. doi:10.15252/msb.20145120

21. Blackwell E, Kim H-JN, Stone DE. The pheromone-induced nuclear accumulation of the Fus3 MAPK in yeast depends on its phosphorylation state and on Dig1 and Dig2. BMC Cell Biol. 2007;8: 44. doi:10.1186/1471-2121-8-44

22. Singer MS, Kahana A, Wolf AJ, Meisinger LL, Peterson SE, Goggin C, et al. Identification of High-Copy Disruptors of Telomeric Silencing in Saccharomyces cerevisiae. Genetics. 1998;150: 613–632. doi:10.1093/genetics/150.2.613
