# Supplementary material for: Intrinsically disordered signaling proteins: Essential hub players in the control of stress responses in Saccharomyces cerevisiae
Source: PLoS One. 2022 Mar 15;17(3):e0265422. doi: 10.1371/journal.pone.0265422 (PMC8923507; doi:10.1371/journal.pone.0265422)
Supplement: S8 Table — (PDF) [file pone.0265422.s019.pdf]

S8 Table: Yeast IDPs involved in heat shock stress response [a].

Protein	Molecular function	Functional elements located in IDRs	Ref
Hsf1	Hsf1 regulates protein homeostasis under basal conditions and it is necessary for recovery upon a brief exposure to extreme temperature.	Hsf1 presents IDRs throughout its sequence. Some IDRs overlap with the two AD domains, phosphorylation sites, with its DNA binding domain (DBD) and with an oligomerization region. The IDR localized at Hsf1 C-terminus self-assembles into highly ordered structures showing amylogenic properties.	[1–3]
Msn2/ Msn4	Msn2/4 is required for long-term survival at high temperatures.	Msn2 has four IDRs located at the N-terminal region. These regions contain the transcriptional activating domain (AD), an essential region for its function and nuclear localization and, towards its carboxy-terminus, the IDRs contain the NES domain, and sites susceptible to phosphorylation, which control the Msn2 export from the nucleus to the cytosol.	[4,5]
Rlm1	Rlm1 belongs to the MADS box family. It controls the expression of genes required for cell integrity, activated by hypotonic stress, heat shock, or impaired cell wall synthesis.	Rlm1 shows two large IDRs, one which completely overlaps with its AD and contains phosphorylation hot spots.	[6–8]
Crz1	Crz1 rapidly translocates to the nucleus and activates gene expression either in response to environmental stress or by exposure to high Ca^{2+} concentrations.	Crz1 contains six IDRs, one extending over its AD, and another partially overlapping with a nuclear export sequence and containing three phosphorylation sites.	[9,10]

[a] These proteins are highlighted in Figures 3 and 4 of the main text.

References

1. Morano KA, Grant CM, Moye-Rowley WS. The Response to Heat Shock and Oxidative Stress in *Saccharomyces cerevisiae*. Genetics. 2012;190: 1157–1195. doi:10.1534/genetics.111.128033

2. D. Westerheide S, Raynes R, Powell C, Xue B, N. Uversky V. HSF Transcription Factor Family, Heat Shock Response, and Protein Intrinsic Disorder. CPPS. 2012;13: 86–103. doi:10.2174/138920312799277956

3. Pujols J, Santos J, Pallarès I, Ventura S. The Disordered C-Terminus of Yeast Hsf1 Contains a Cryptic Low-Complexity Amyloidogenic Region. IJMS. 2018;19: 1384. doi:10.3390/ijms19051384

4. Sadeh A, Baran D, Volokh M, Aharoni A. Conserved Motifs in the Msn2-Activating Domain are Important for Msn2-Mediated Yeast Stress Response. Journal of Cell Science. 2012; 3333–3342. doi:10.1242/jcs.096446

5. Yamamoto N, Maeda Y, Ikeda A, Sakurai H. Regulation of Thermotolerance by Stress-Induced Transcription Factors in *Saccharomyces cerevisiae*. Eukaryot Cell. 2008;7: 783–790. doi:10.1128/EC.00029-08

6. Jung US, Sobering AK, Romeo MJ, Levin DE. Regulation of the yeast Rlm1 transcription factor by the Mpk1 cell wall integrity MAP kinase: Reporters for cell wall integrity signalling. Molecular Microbiology. 2002;46: 781–789. doi:10.1046/j.1365-2958.2002.03198.x

7. Staleva L, Hall A, Orlow SJ. Oxidative Stress Activates FUS1 and RLM1 Transcription in the Yeast Saccharomyces cerevisiae in an Oxidant- dependent Manner□D. Molecular Biology of the Cell. 2004;15: 9.

8. Jakobson CM, Jarosz DF. Molecular Origins of Complex Heritability in Natural Genotype-to-Phenotype Relationships. Cell Systems. 2019;8: 363–379. doi:10.1016/j.cels.2019.04.002

9. Boustany LM, Cyert MS. Calcineurin-dependent regulation of Crz1p nuclear export requires Msn5p and a conserved calcineurin docking site. Genes Dev. 2002;16: 608–619. doi:10.1101/gad.967602

10. Cyert MS. Calcineurin signaling in Saccharomyces cerevisiae: how yeast go crazy in response to stress. Biochemical and Biophysical Research Communications. 2003;311: 1143–1150. doi:10.1016/S0006-291X(03)01552-3
