# Supplementary material for: Intrinsically disordered signaling proteins: Essential hub players in the control of stress responses in Saccharomyces cerevisiae
Source: PLoS One. 2022 Mar 15;17(3):e0265422. doi: 10.1371/journal.pone.0265422 (PMC8923507; doi:10.1371/journal.pone.0265422)
Supplement: S9 Table — (PDF) [file pone.0265422.s020.pdf]

S9 Table: Yeast IDPs involved in nutrient adaptation [a].

Protein	Molecular function	Functional elements located in IDRs	Ref
Sfp1	Sfp1 is a TF controlling expression, processing, and localization of ribosomal proteins, through its phosphorylation and interaction with TORC1, which promotes Sfp1 translocation into the nucleus. Therefore, Sfp1 degradation leads to the repression of the genes involved in these processes.	Sfp1 contains four IDRs, most of them in the C-terminal region, where one IDR overlaps with its DBD and with a recognized AD.	[1]
Npr1	Npr1 is a protein kinase phosphorylated by TORC1, involved in the control of amino acid transport.	Npr1 presents five IDRs, overlapping with phosphorylation sites and with its catalytic domain.	[1]
Sip2	Sip2 is part of one of the Snf1 complex subunits and leads to Snf1 localization into the cytosol.	Sip2 contains three IDRs, the longest one located at its N-terminal region.	[2–4]
Gal83	Gal83 directs Snf1 to the nucleus, during glucose depletion.	Gal83 shows two IDRs, extending over its N-terminal domain.	[2,5]
Mks1	Mks1 acts as a negative regulator of Rtg1 to control amino acid biosynthesis.	Mks1 contains five IDR, two of them overlapping with phosphorylation sites.	[6]
Reg1	Reg1 is the regulatory subunit of Glc7 protein phosphatase 1, involved in Snf1 and Gpa1 dephosphorylation, depending on glucose availability.	Reg1 has eight predicted IDRs distributed throughout its sequence, some of them containing phosphorylation motifs.	[7,8]
Mig1	Mig1 is a transcriptional repressor inactivated by Snf1-mediated phosphorylation. Mig1 inactivation leads to the derepression of alternative carbon source metabolism and promotes filamentous growth under glucose deficiency.	Mig1 structural disorder is scattered in six IDRs located all over its length, from which those in the N-terminal region partially overlap with identified DBDs.	[9,10]
Cyc8	Cyc8 is a TF that in complex with Tup1 and Dbp2 represses the expression of various genes involved in the utilization of alternative carbon sources. Cyc8 also is part of a coactivator complex able to recruit SWI/SNF and SAGA complexes to promoters. The Cyc8-Tup1 corepressor complex also associate to the ATF/CREB repressor Sko1, which by Hog1 phosphorylation converts this repressor complex into an activator of hyperosmotic stress responsive genes.	Cyc8 has four IDRs harboring putative phosphorylation sites.	[10–12]
Sko1	Sko1 recruitment into the Cyc8-Tup1 repressor complex also controls gene expression in response to nutrient fluctuations but not to glucose.	The recognized phosphorylation sites of Sko1 fall into two of its three IDRs.	[11]
Azf1	Azf1 is a TF, member of the C2H2 zinc finger class, involved in the activation of genes associated to growth, carbon metabolism and cell wall integrity.	Azf1 contains six IDRs distributed throughout its sequence.	[13]
Gln3	Gln3 is an essential TF participating in the activation of genes involved in the Nitrogen Catabolite Repression (NCR) system. The activity of Gln3 is controlled mostly by phosphorylation, showing 146 putative phosphorylation sites. Its phosphorylation state dictates its nuclear localization, its response to TORC1 and nitrogen limitation, and its association to different proteins.	Gln3 contains seven IDR, three located at its carboxy terminal region (alfa-cap), implicated in its nuclear localization in response to TORC1.	[1,14]

Hsf1	Hsf1 its phosphorylated by Snf1. During nutrient adaptation, a subset of Hsf1 gene targets is controlled by glucose starvation depending on Snf1 and the Hsf1 carboxyl-terminal activation domain.	Hsf1 carboxyl-terminal activation domain, which overlaps with two of its six IDRs.	[9]
Sok2	Sok2 is a TF regulated by PKA through Yak1, and its activity impacts on *FLO11* gene expression.	Sok2 presents four IDRs scattered throughout their sequence.	[15]
Haa1	Haa1 is a TF regulated by PKA through Yak1, and its activity impacts on *FLO11* gene expression.	Haa1 presents five IDRs scattered throughout their sequence.	[15]
Flo11	Flo11 is a glycoprotein required for pseudohyphal and invasive growth, flocculation, and biofilm formation.	Flo11 presents one IDR that has not been associated with its known functions,	[15]
Dig1	Dig1 is negatively regulated by a signaling pathway sensing hyperosmotic conditions through the Msb2 and Sho1 sensors. It is a negative regulator of Ste12 TF, also an IDP.	Dig1 presents three IDRs located to its N- and C-terminal domains and containing several phosphorylation sites.	[16]
Mss11	Mss11 is involved in the control of filamentous growth and starch metabolism through the activation of Flo11.	Mss11 contains five IDRs.	[17]
Stb3 and Tod6	Stb3 and Tod6 are repressors acting on the promoters of genes encoding ribosomal proteins or involved in ribosome biogenesis. Under nutrient scarcity, these IDTFs inhibit the expression of a large number of genes involved in the growth process and its control for a successful adaptation to these hostile conditions.	Stb3 and Tod6 have three and five IDRs located to their N- and C-terminal domains respectively.	[18,19]
Ume6	Ume6 is a TF implied in coupling the control of meiosis with metabolic regulation triggered by nitrogen deprivation. Ume6 can act as repressor and as activator depending on its protein interactors. Its repression activity is exerted through its association to a histone deacetylase complex leading to an epigenetic repression.	Ume6 contains seven IDRs, three of them extending over protein binding regions and phosphorylation sites.	[20,21]
Hap2, 4, and 5	This Hap complex is highly conserved in eukaryotes. Subunits 2, 3, and 5 form the DNA binding domain, whereas Hap4 contains the Hap complex activation domain. The Hap complex functions as a positive and negative regulator and has been involved in the perception of oxidative stress, in the control of iron homeostasis, of nitrogen metabolism and of the balance between respiration and fermentation.	Hap2, 4, and 5 have a high number of disordered residues, distributed in one, three and five IDRs, respectively, some of them overlapping with protein binding or phosphorylation sites.	[22,23]

[a] These proteins are highlighted in Figures 3 and 5 of the main text.

References

1. Conrad M, Schothorst J, Kankipati HN, Van Zeebroeck G, Rubio-Texeira M, Thevelein JM. Nutrient sensing and signaling in the yeast Saccharomyces cerevisiae. FEMS Microbiol Rev. 2014;38: 254–299. doi:10.1111/1574-6976.12065

2. Jiang R, Carlson M. The Snf1 protein kinase and its activating subunit, Snf4, interact with distinct domains of the Sip1/Sip2/Gal83 component in the kinase complex. Mol Cell Biol. 1997;17: 2099–2106. doi:10.1128/MCB.17.4.2099

3. Lin SS, Manchester JK, Gordon JI. Sip2, an N-Myristoylated β Subunit of Snf1 Kinase, Regulates Aging in Saccharomyces cerevisiae by Affecting Cellular Histone Kinase Activity, Recombination at rDNA Loci, and Silencing. Journal of Biological Chemistry. 2003;278: 13390–13397. doi:10.1074/jbc.M212818200

4. Hedbacker K, Townley R, Carlson M. Cyclic AMP-Dependent Protein Kinase Regulates the Subcellular Localization of Snf1-Sip1 Protein Kinase. Mol Cell Biol. 2004;24: 1836–1843. doi:10.1128/MCB.24.5.1836-1843.2004

5. Hedbacker K, Carlson M. Regulation of the Nucleocytoplasmic Distribution of Snf1-Gal83 Protein Kinase. Eukaryot Cell. 2006;5: 1950–1956. doi:10.1128/EC.00256-06

6. Dilova I, Chen C-Y, Powers T. Mks1 in Concert with TOR Signaling Negatively Regulates RTG Target Gene Expression in S. cerevisiae. Current Biology. 2002;12: 389–395. doi:10.1016/S0960-9822(02)00677-2

7. Tu J, Carlson M. REG1 binds to protein phosphatase type 1 and regulates glucose repression in Saccharomyces cerevisiae. The EMBO Journal. 1995;14: 5939–5946. doi:10.1002/j.1460-2075.1995.tb00282.x

8. Castermans D, Somers I, Kriel J, Louwet W, Wera S, Versele M, et al. Glucose-induced posttranslational activation of protein phosphatases PP2A and PP1 in yeast. Cell Res. 2012;22: 1058–1077. doi:10.1038/cr.2012.20

9. Hahn J-S, Thiele DJ. Activation of the Saccharomyces cerevisiae Heat Shock Transcription Factor Under Glucose Starvation Conditions by Snf1 Protein Kinase. Journal of Biological Chemistry. 2004;279: 5169–5176. doi:10.1074/jbc.M311005200

10. Wang S, Xing Z, Pascuzzi PE, Tran EJ. Metabolic Adaptation to Nutrients Involves Coregulation of Gene Expression by the RNA Helicase Dbp2 and the Cyc8 Corepressor in *Saccharomyces cerevisiae*. G3 Genes|Genomes|Genetics. 2017;7: 2235–2247. doi:10.1534/g3.117.041814

11. Proft M, Struhl K. Hog1 Kinase Converts the Sko1-Cyc8-Tup1 Repressor Complex into an Activator that Recruits SAGA and SWI/SNF in Response to Osmotic Stress. Molecular Cell. 2002;9: 1307–1317. doi:10.1016/S1097-2765(02)00557-9

12. Tam J, van Werven FJ. Regulated repression governs the cell fate promoter controlling yeast meiosis. Nat Commun. 2020;11: 2271. doi:10.1038/s41467-020-16107-w

13. Slattery MG, Liko D, Heideman W. The Function and Properties of the Azf1 Transcriptional Regulator Change with Growth Conditions in *Saccharomyces cerevisiae*. Eukaryot Cell. 2006;5: 313–320. doi:10.1128/EC.5.2.313-320.2006

14. Feller A, Georis I, Tate JJ, Cooper TG, Dubois E. Alterations in the Ure2 αCap Domain Elicit Different GATA Factor Responses to Rapamycin Treatment and Nitrogen Limitation. Journal of Biological Chemistry. 2013;288: 1841–1855. doi:10.1074/jbc.M112.385054

15. Malcher M, Schladebeck S, Mösch H-U. The Yak1 Protein Kinase Lies at the Center of a Regulatory Cascade Affecting Adhesive Growth and Stress Resistance in *Saccharomyces cerevisiae*. Genetics. 2011;187: 717–730. doi:10.1534/genetics.110.125708

16. Bardwell L, Cook JG, Zhu-Shimoni JX, Voora D, Thorner J. Differential regulation of transcription: Repression by unactivated mitogen-activated protein kinase Kss1 requires the Dig1 and Dig2 proteins. Proceedings of the National Academy of Sciences. 1998;95: 15400–15405. doi:10.1073/pnas.95.26.15400

17. Gagiano M, Bester M, Van Dyk D, Franken J, Bauer FF, Pretorius IS. Mss11p is a transcription factor regulating pseudohyphal differentiation, invasive growth and starch metabolism in *Saccharomyces cerevisiae* in response to nutrient availability. Molecular Microbiology. 2003;47: 119–134. doi:10.1046/j.1365-2958.2003.03247.x

18. Lippman SI, Broach JR. Protein kinase A and TORC1 activate genes for ribosomal biogenesis by inactivating repressors encoded by Dot6 and its homolog Tod6. Proceedings of the National Academy of Sciences. 2009;106: 19928–19933. doi:10.1073/pnas.0907027106

19. Liko D, Conway MK, Grunwald DS, Heideman W. Stb3 Plays a Role in the Glucose-Induced Transition from Quiescence to Growth in *Saccharomyces cerevisiae*. Genetics. 2010;185: 797–810. doi:10.1534/genetics.110.116665

20. Williams RM, Primig M, Washburn BK, Winzeler EA, Bellis M, Sarrauste de Menthiere C, et al. The Ume6 regulon coordinates metabolic and meiotic gene expression in yeast. Proceedings of the National Academy of Sciences. 2002;99: 13431–13436. doi:10.1073/pnas.202495299

21. Zhang Y, Iratni R, Erdjument-Bromage H, Tempst P, Reinberg D. Histone Deacetylases and SAP18, a Novel Polypeptide, Are Components of a Human Sin3 Complex. Cell. 1997;89: 357–364. doi:10.1016/S0092-8674(00)80216-0

22. Mao Y, Chen C. The Hap Complex in Yeasts: Structure, Assembly Mode, and Gene Regulation. Front Microbiol. 2019;10: 1645. doi:10.3389/fmicb.2019.01645

23. Bolotin-Fukuhara M. Thirty years of the HAP2/3/4/5 complex. Biochimica et Biophysica Acta (BBA) - Gene Regulatory Mechanisms. 2017;1860: 543–559. doi:10.1016/j.bbagrm.2016.10.011
