# Supplementary material for: Intrinsically disordered signaling proteins: Essential hub players in the control of stress responses in Saccharomyces cerevisiae
Source: PLoS One. 2022 Mar 15;17(3):e0265422. doi: 10.1371/journal.pone.0265422 (PMC8923507; doi:10.1371/journal.pone.0265422)
Supplement: S10 Table — (PDF) [file pone.0265422.s021.pdf]

S10 Table: Yeast IDPs involved in oxidative stress response [a].

Protein	Molecular function	Functional elements located in IDRs	Ref
Yap1	Yap1 is translocated from the cytoplasm into the nucleus upon an oxidant condition, a relocation mediated by the cysteine rich domains. Yap1 transcriptional activity also is involved in response to a hyperoxidant environment produced by diamide or H_2O_2. Yap1 CRDs play a key role in the regulation of the thioredoxin encoding gene (*TRX2)*, which is required for peroxide resistance.	Yap1 presents four IDRs located at the N- and C-termini (n-CDR and c-CRD) and involved in the regulation of Yap1 nuclear localization and activity.	[1]
Msn2/ Msn4	Msn2/Msn4 proteins are important in oxidative stress response. They regulate the activity of Mot3 and Rox1, transcriptional repressors of hypoxic genes, among others	Msn2 has four IDRs located at the N-terminal region, which contains the transcriptional activating domain (AD), an essential region for its function and nuclear localization. Towards its carboxy-terminus, Msn2 contains its NES domain, and sites susceptible to phosphorylation, controlling its export from the nucleus to the cytosol.	[2–4]
Rlm1	Rlm1 belongs to the MADS box family. It controls the gene expression required to keep functioning the cell integrity pathway, activated by hypotonic stress, heat shock, or impaired cell wall synthesis.	Rlm1 shows two large IDRs, one which completely overlaps with its AD and contains hot spots for phosphorylation.	[5–7]
Crz1	Crz1p rapidly translocates to the nucleus and activates gene expression either in response to specific environmental stresses or by addition of Ca^{2+} to the media.	Crz1 contains six IDRs, one extending over its AD, and another partially overlapping a nuclear export sequence and containing three phosphorylation sites.	[8,9]
Gln3	Gln3 is a transcriptional regulator of nitrogen catabolism that affects the efficiency of translation termination	Gln3 contains seven IDRs distributed throughout its sequence.	[10,11]

[a] These proteins are highlighted into Figures 3 and 5 of the main text.

.

References

1. Bolotin-Fukuhara M. Thirty years of the HAP2/3/4/5 complex. Biochimica et Biophysica Acta (BBA) - Gene Regulatory Mechanisms. 2017;1860: 543–559. doi:10.1016/j.bbagrm.2016.10.011

2. Sadeh A, Baran D, Volokh M, Aharoni A. Conserved Motifs in the Msn2-Activating Domain are Important for Msn2-Mediated Yeast Stress Response. Journal of Cell Science. 2012; 3333–3342. doi:10.1242/jcs.096446

3. Yamamoto N, Maeda Y, Ikeda A, Sakurai H. Regulation of Thermotolerance by Stress-Induced Transcription Factors in *Saccharomyces cerevisiae*. Eukaryot Cell. 2008;7: 783–790. doi:10.1128/EC.00029-08

4. Martínez-Montañés F, Rienzo A, Poveda-Huertes D, Pascual-Ahuir A, Proft M. Activator and Repressor Functions of the Mot3 Transcription Factor in the Osmostress Response of Saccharomyces cerevisiae. Eukaryotic Cell. 2013;12: 636–647. doi:10.1128/EC.00037-13

5. Jung US, Sobering AK, Romeo MJ, Levin DE. Regulation of the yeast Rlm1 transcription factor by the Mpk1 cell wall integrity MAP kinase: Reporters for cell wall integrity signalling. Molecular Microbiology. 2002;46: 781–789. doi:10.1046/j.1365-2958.2002.03198.x

6. Staleva L, Hall A, Orlow SJ. Oxidative Stress Activates FUS1 and RLM1 Transcription in the Yeast Saccharomyces cerevisiae in an Oxidant- dependent Manner□D. Molecular Biology of the Cell. 2004;15: 9.

7. Jakobson CM, Jarosz DF. Molecular Origins of Complex Heritability in Natural Genotype-to-Phenotype Relationships. Cell Systems. 2019;8: 363–379. doi:10.1016/j.cels.2019.04.002

8. Boustany LM, Cyert MS. Calcineurin-dependent regulation of Crz1p nuclear export requires Msn5p and a conserved calcineurin docking site. Genes Dev. 2002;16: 608–619. doi:10.1101/gad.967602

9. Cyert MS. Calcineurin signaling in Saccharomyces cerevisiae: how yeast go crazy in response to stress. Biochemical and Biophysical Research Communications. 2003;311: 1143–1150. doi:10.1016/S0006-291X(03)01552-3

10. Rai R, Tate JJ, Shanmuganatham K, Howe MM, Nelson D, Cooper TG. Nuclear Gln3 Import Is Regulated by Nitrogen Catabolite Repression Whereas Export Is Specifically Regulated by Glutamine. Genetics. 2015;201: 989–1016. doi:10.1534/genetics.115.177725

11. Antonets KS, Belousov MV, Belousova ME, Nizhnikov AA. The Gln3 Transcriptional Regulator of Saccharomyces cerevisiae Manifests Prion-Like Properties upon Overproduction. Biochemistry Moscow. 2019;84: 441–451. doi:10.1134/S0006297919040126
