# Supplementary material for: Intrinsically disordered signaling proteins: Essential hub players in the control of stress responses in Saccharomyces cerevisiae
Source: PLoS One. 2022 Mar 15;17(3):e0265422. doi: 10.1371/journal.pone.0265422 (PMC8923507; doi:10.1371/journal.pone.0265422)
Supplement: S11 Table — (PDF) [file pone.0265422.s022.pdf]

S11 Table. Number of proteins in ranges of disorder ratio by stress pathways determined using four disorder predictors programs and FuzPred.

	"D" Ratio VSL2		"D" Ratio IUPred2A		"D" Ratio MobiDB-Lite		"D" Ratio MobiDB		FuzPred	
range	# Proteins	# TF	# Proteins	# TF	# Proteins	# TF	# Proteins	# TF	# Proteins	# TF
**Heat shock response**										
0.0 - 0.2	16	0	44	0	48	0	48	0	19	0
0.2 - 0.4	28	0	32	1	36	4	34	4	22	0
0.4 - 0.6	30	0	16	4	11	3	12	3	15	1
0.6 - 0.8	18	4	6	3	5	2	6	2	16	2
0.8 - 1.0	9	6	3	2	1	1	1	1	29	7
**Ion Homeostasis**										
0.0 - 0.2	58	0	136	0	146	4	128	0	78	0
0.2 - 0.4	80	0	64	7	73	10	84	15	51	1
0.4 - 0.6	59	5	46	13	36	10	43	10	22	1
0.6 - 0.8	42	14	12	6	6	4	6	3	6	6
0.8 - 1.0	24	10	5	3	2	1	2	1	87	21
**Nutrient Adaptation**										
0.0 - 0.2	36	0	85	1	91	2	87	2	42	0
0.2 - 0.4	53	0	43	4	46	8	51	9	39	1
0.4 - 0.6	48	7	28	12	27	11	28	12	16	2
0.6 - 0.8	20	9	15	10	13	8	14	9	19	3
0.8 - 1.0	23	16	9	5	3		0	0	64	26
**Osmotic Stress response**										
0.0 - 0.2	31	0	56	0	62	1	56	0	27	0
0.2 - 0.4	30	0	35	3	46	3	49	6	32	0
0.4 - 0.6	44	3	41	7	28	8	30	7	11	1
0.6 - 0.8	25	6	8	5	7	4	8	3	13	2
0.8 - 1.0	15	8	5	2	2	1	2	1	62	14
**Oxidative Stress response**										
0.0 - 0.2	33	0	71	0	78	1	73	0	46	0
0.2 - 0.4	40	0	31	2	31	2	34	3	29	0
0.4 - 0.6	32	1	24	5	21	7	23	8	11	1
0.6 - 0.8	20	5	7	6	6	4	6	3	13	2
0.8 - 1.0	13	9	5	2	2	1	2	1	39	12
